# *N*-Hydroxybenzimidazole as a structurally modifiable platform for *N*-oxyl radicals for direct C–H functionalization reactions†
†Electronic supplementary information (ESI) available: Experimental procedures, computational studies and characterization for all relevant compounds. See DOI: 10.1039/d0sc02134b


**DOI:** 10.1039/d0sc02134b

**Published:** 2020-05-18

**Authors:** Tomomi Yoshii, Saori Tsuzuki, Shunya Sakurai, Ryu Sakamoto, Julong Jiang, Miho Hatanaka, Akira Matsumoto, Keiji Maruoka

**Affiliations:** a Department of Chemistry , Graduate School of Science , Kyoto University , Sakyo , Kyoto 606-8502 , Japan . Email: maruoka.keiji.4w@kyoto-u.ac.jp; b Institute for Research Initiatives , Division for Research Strategy , Graduate School of Materials Science , Data Science Center , Nara Institute of Science and Technology , Ikoma , Nara 630-0192 , Japan; c PRESTO , Japan Science and Technology (JST) , 4-1-8 Honcho , Kawaguchi , Saitama 332-0012 , Japan; d Graduate School of Pharmaceutical Sciences , Kyoto University , Sakyo , Kyoto 606-8501 , Japan; e School of Chemical Engineering and Light Industry , Guangdong University of Technology , Guangzhou 510006 , China

## Abstract

Methods for direct functionalization of C–H bonds mediated by *N*-oxyl radicals constitute a powerful tool in modern organic synthesis. While several *N*-oxyl radicals have been developed to date, the lack of structural diversity for these species has hampered further progress in this field. Here we designed a novel class of *N*-oxyl radicals based on *N*-hydroxybenzimidazole, and applied them to the direct C–H functionalization reactions. The flexibly modifiable features of these structures enabled facile tuning of their catalytic performance. Moreover, with these organoradicals, we have developed a metal-free approach for the synthesis of acyl fluorides *via* direct C–H fluorination of aldehydes under mild conditions.

## Abstract 2

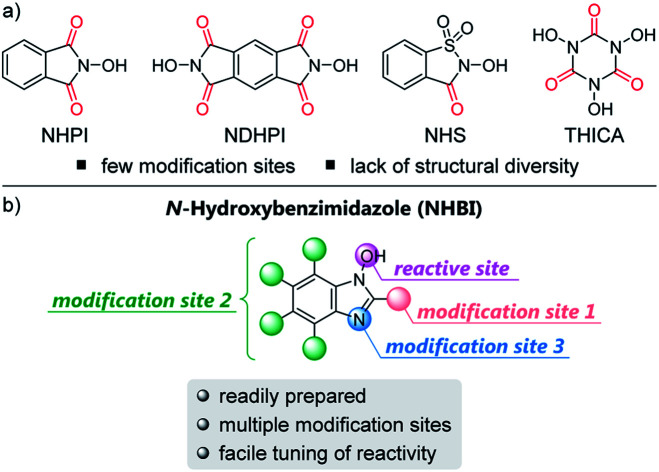
A novel class of *N*-oxy radicals based on flexibly modifiable *N*-hydroxybenzimidazole skeleton was designed and applied to C–H functionalization reactions.

## Introduction

**Fig. 1:**
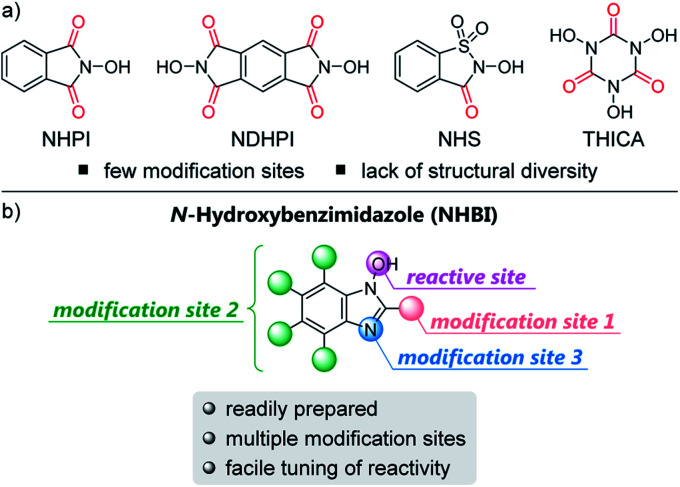
(a) Representative examples of *N*-hydroxy compounds. (b) Design of *N*-hydroxybenzimidazole as *N*-oxyl radical platform.

The direct functionalization of C–H bonds has been recognized as an innovative approach to realize step- and atom-economical synthesis of various functionalized molecules. Considerable research efforts have been devoted to the development of a variety of C–H functionalization reactions based on transition-metal-catalyzed or photoredox-promoted approaches.^1^,^2^ On the other hand, transition-metal-free and non-photolytic direct C–H functionalization reactions still remain a significant challenge,^3^ although they will provide new synthetic strategies with complementary reactivity and selectivity.^4^ In this context, *N*-oxyl radicals have attracted much attention as promising organoradical catalysts for these transformations.^5^ As represented by *N*-hydroxyphthalimide (NHPI), several *N*-hydroxy compounds have been used to generate active *N*-oxyl radicals *in situ*, which selectively abstract hydrogen atoms from C–H bonds of organic molecules. The resulting alkyl radicals react with various radical acceptors to afford the corresponding functionalized products.^6^ In order to improve the catalytic efficiency and stability of the key *N*-oxyl radicals, several *N*-hydroxy compounds as organoradical precursors have been designed based on the structure of NHPI and utilized in the same type of transformations (Fig. 1a).^7^ Most of the reported *N*-hydroxy compounds, however, have similar structures featuring carbonyl groups adjacent to hydroxylamine moiety as a reactive site. While these carbonyl groups significantly contribute to enhancing the catalytic performance of *N*-oxyl radicals,^8^ only a few sites are available for structural modification due to their existence. As a result, such a lack of structural diversity for these *N*-hydroxy compounds renders it difficult to alter the specific properties of the corresponding *N*-oxyl radicals such as bond dissociation energies (BDEs), a fundamental parameter to estimate their reactivities in the hydrogen atom transfer process. Accordingly, further progress in this field demands a completely novel design of *N*-oxyl radicals with structural diversity. Herein, we report the design and synthesis of novel organoradical species based on *N*-hydroxybenzimidazoles (NHBIs), which contain multiple modification sites (Fig. 1b), and demonstrate their distinct reactivities as organoradical catalysts and efficient radical initiators in the direct C–H functionalization reactions.

*N*-Hydroxybenzimidazoles, which are planar and stable heterocycles bearing an *N*-hydroxy moiety, have recently attracted interest in the field of biological and pharmaceutical sciences as anti-virulence or anti-cancer agents.^9^,^10^ However, they have rarely been used in synthetic organic chemistry and, to the best of our knowledge, studies on the potential of NHBIs to generate the corresponding *N*-oxyl radicals have not yet been conducted.^11^ In this context, we became interested in the potential of NHBIs as novel organoradical precursors based on the following features: (1) NHBIs can be readily prepared from 2-nitroaniline derivatives in a few steps (see the ESI†); (2) substituents can be easily introduced at both the aromatic ring and the 2-position of the benzimidazole moiety; (3) NHBIs contain additional modification sites such as the nitrogen atom at the 3-position of the benzimidazole moiety and the counteranion of the resulting benzimidazolium species, which may potentially be exploited for further functionalization.

## Results and discussion

**Fig. 2:**
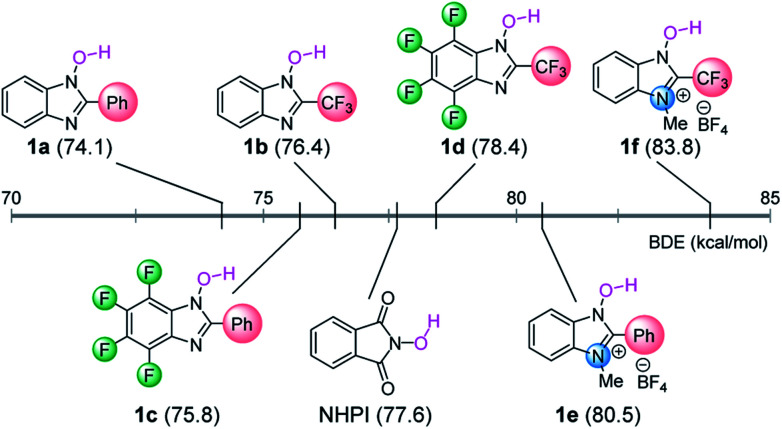
Calculated BDE values for O–H bond in **1** and NHPI. Calculations were performed at B3LYP-D3/6-311G(d,p) in SMD (MeCN) level of theory. For details, see the ESI.†

To test our hypothesis, we initially carried out density functional theory (DFT) calculations in order to estimate the BDE values of the O–H bonds in our designed NHBI derivatives (**1a–f**) (Fig. 2).^12^ As expected, the results of the DFT calculations revealed that the BDE values for **1** can be tuned within a wide range (∼10 kcal mol^–1^) by facile modifications such as the introduction of substituents at the aromatic ring or the 2-position of the imidazole moiety, or by *N*-alkylation at the 3-position. Moreover, some of synthesized NHBI derivatives have the similar or even higher BDEs compared to that of NHPI, which has been widely used as an efficient catalyst for several hydrogen atom abstraction reactions.^6^ Thus, these results indicate that *N*-oxyl radicals derived from NHBI derivatives potentially work as organoradical catalysts for direct C–H functionalization *via* hydrogen atom abstraction.

**Scheme 1:**
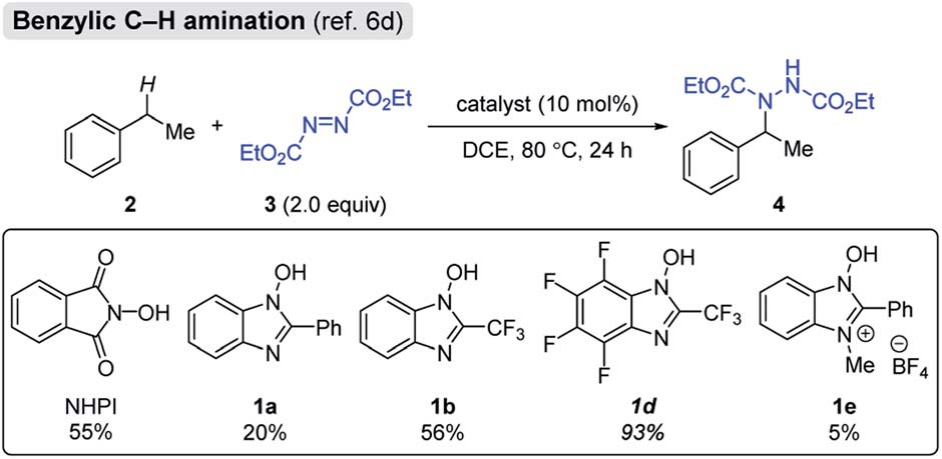
Evaluation of **1** as organoradical catalyst for benzylic C–H amination. Yields were determined by ^1^H NMR spectroscopy using 1,1,2,2-tetrachloroethane as an internal standard.

In order to evaluate the catalytic activities of NHBI derivatives, we attempted to apply them to the reported C–H functionalization reactions. Among several transformations catalyzed by *N*-oxyl radicals, we selected the benzylic C–H amination of ethylbenzene (**2**) as a model reaction, which was originally reported as the NHPI-catalyzed reaction (Scheme 1).6*d* After the reaction was run using diethyl azodicarboxylate (**3**) in the presence of 10 mol% of NHPI at 80 °C for 24 h, the desired aminated product **4** was obtained in moderate yield. On the other hand, yields of **4** for reactions using NHBI derivatives varied significantly, depending on their substituents. Notably, when **1d** was used under the same reaction conditions, the yield of **4** was much higher than the use of NHPI. On the contrary, cationic NHBI derivative **1e**, which has a highest value of BDE among examined *N*-hydroxy compounds, did not show remarkable catalytic activity for this transformation, probably due to difficult regeneration of active *N*-oxy radicals. These experimental results clearly indicate that our designed NHBI derivatives can be applied to direct C–H functionalization reactions.

**Scheme 2:**
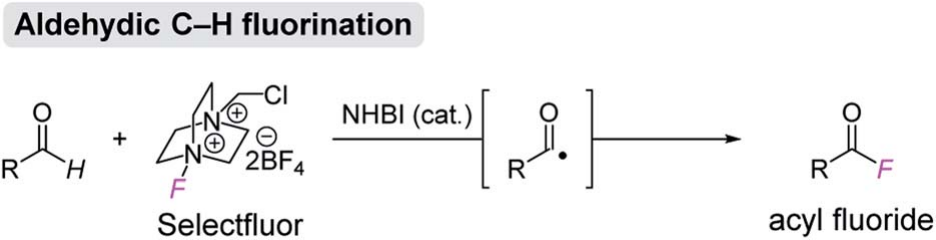
Aldehydic C–H fluorination reaction using NHBI.

Having confirmed the potential of NHBI derivatives as active organoradical precursors, we set about exploring their distinct reactivities for the development of direct C–H functionalization reactions. Our continuing concern about methods for generation and application of acyl radical species^13^,^14^ led us to investigate the direct functionalization of aldehydes *via* C(sp^2^)–H bond activation by NHBI derivatives. To our delight, we found that in the presence of catalytic amount of NHBI, the aldehydic C–H bond was directly converted into C–F bond by using Selectfluor as a fluorine atom transfer reagent, affording the corresponding acyl fluorides (Scheme 2). Acyl fluorides have recently attracted much attention in synthetic and biological chemistry owing to their unique reactivity, which is not observable in commonly employed acyl chlorides or acid anhydrides.^15^,^16^ While several synthetic methods for acyl fluorides have been reported, they are typically prepared by using toxic fluorination reagents and/or precious metal catalysts.^17^,^18^ Therefore, the development of their different synthetic approach with high practicability is in great demand.

Initially, the effect of NHBI derivatives was assessed in the reaction of 3-phenylpropanal (**5a**) with Selectfluor (Table 1). Although the reaction using 10 mol% of **1a** did not afford the desired acyl fluoride **6a** (entry 1), fluorine-substituted NHBIs **1b–d** afforded **6a** in moderate to good yields (entries 2–4). It is noteworthy that cationic NHBI derivative **1e**, which was not active for the aforementioned benzylic C–H amination reaction (Scheme 1), turned out to be effective for this C–H fluorination reaction (entry 5). During the optimization, we observed the formation of 3-phenylpropionic acid (**6a′**) as a side product, which could potentially be formed during the reaction of an acyl radical with oxygen.^19^ Thus, we carried out the reaction under an inert atmosphere of argon, which further improved the yield of **6a** with the generation of **6a′** sufficiently suppressed (entry 6). On the other hand, the introduction of trifluoromethyl group at the 2-position of benzimidazolium structure gave only a small amount of the product (entry 7). While the effect of counteranion for cationic NHBI derivative is almost negligible (entry 8), the importance of free hydroxylamine moiety of NHBI was confirmed by the reaction with *N*-benzyloxybenzimidazole **1h** (entry 9). Neither **6a** or **6a′** was obtained in the absence of NHBI derivative (entry 10). As a comparative study, NHPI and its derivatives were used for this transformation (entries 11–13). Although the reactions proceeded moderately in the presence of these *N*-hydroxy compounds, the yields of **6a** were much lower than the use of **1e**.

**Table 1:** Optimization of reaction conditions^*a*^ ^,^^*b*^

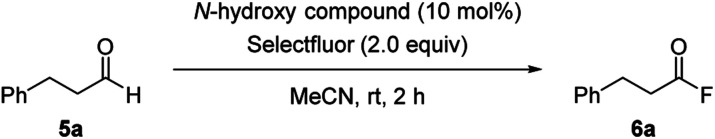				
Entry	*N*-Hydroxy compound	Yield of **6a** (%)	Yield of **6a′** (%)	Recovery of **5a** (%)
1	**1a**	N.D.	10	81
2	**1b**	28	21	45
3	**1c**	33	28	39
4	**1d**	61	16	8
5	**1e**	64	12	11
6^*c*^	**1e**	77	5	18
7^*c*^	**1f**	4	17	83
8^*c*^	**1g**	72	2	9
9^*c*^	**1h**	Trace	Trace	97
10^*c*^	—	N.D.	Trace	96
11^*c*^	NHPI	47	18	26
12^*c*^	4-Nitro-NHPI	19	36	15
13^*c*^	THICA	46	17	16
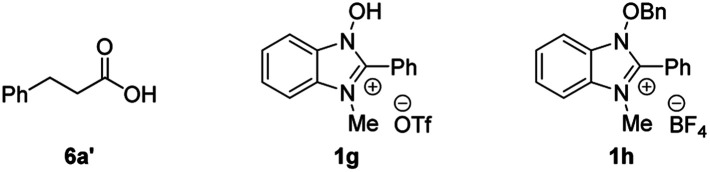				

^*a*^Unless otherwise specified, reactions were carried out in MeCN for 2 h in the presence of **5a** (0.20 mmol), Selectfluor (2.0 equiv.) and *N*-hydroxy compound (10 mol%).

^*b*^Yields of **6a** were determined by ^1^H NMR spectroscopy using benzotrifluoride as the internal standard.

^*c*^The reaction was conducted under an atmosphere of argon. N.D.: not detected.

With the optimal NHBI derivative in hand, the scope of aldehyde was then investigated (Table 2). While isolated yields of products were determined after one-pot conversion into benzyl amides **7** to avoid loss of acyl fluorides **6** due to their volatility and/or instability on silica-gel, several reactions could be scaled up to 2.0 mmol and the obtained acyl fluorides were successfully isolated after flash column chromatography in acceptable yields. The reactions of aliphatic aldehydes proceeded even at room temperature, affording the desired products in moderate to high yields. Desired products bearing benzylic C–H bond (**6a–7a**), acyclic or cyclic ether moieties (**7f** and **7i**) and nitrogen-containing functional groups (**6d–7d**, **6h–7h** and **7k**) were successfully obtained, highlighting the advantage of mild reaction conditions. Although pivalaldehyde gave the corresponding product **7j** in low yield due to competitive decarbonylation,18*d* functionalized aldehyde with two stereogenic centers provided the desired benzylamide **7k** without epimerization. Moreover, products from aryl aldehydes (**7l**, **6m–7m** and **6n–7n**) and *trans*-cinnamaldehyde (**6o–7o**) could also be obtained at increased reaction temperature.

**Table 2:** Scope of aldehydes

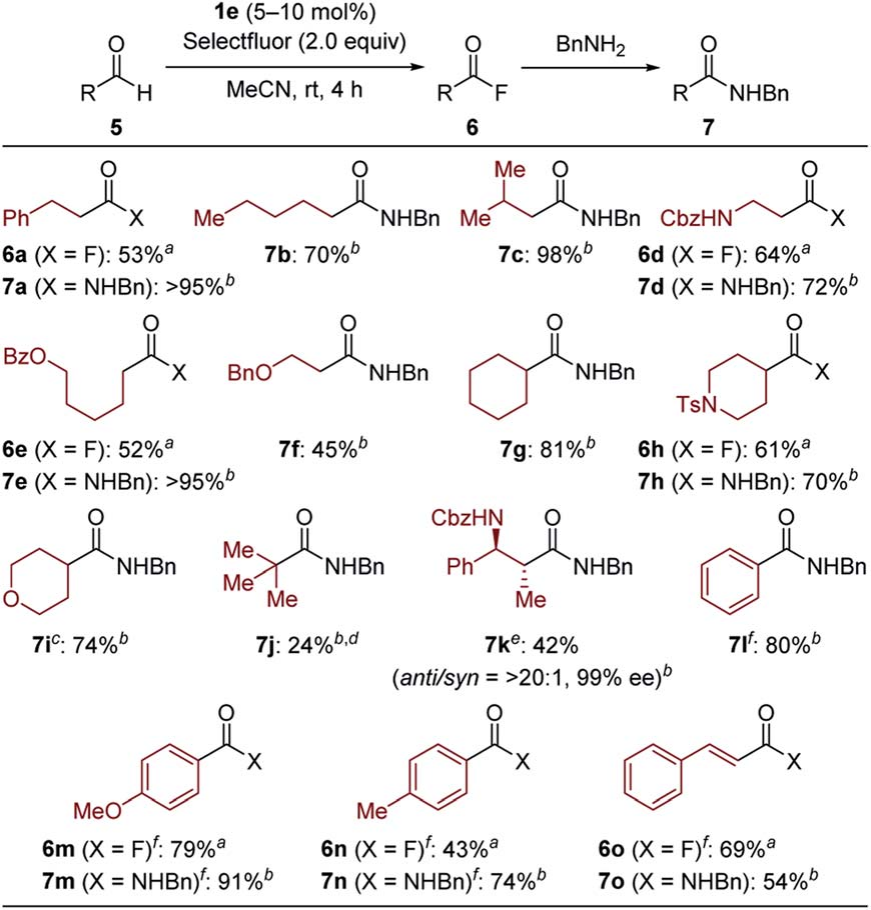

^*a*^The reaction was carried out in MeCN for 4 h in the presence of **5** (2.0 mmol), Selectfluor (2.0 equiv.) and **1e** (10 mol%). Isolated as acyl fluoride **6**.

^*b*^The reaction was carried out in MeCN for 4 h in the presence of **5** (0.20 mmol), Selectfluor (1.0 equiv.) and **1e** (5.0 mol%), followed by a treatment with benzylamine (2.0 equiv.) for 2 h. Isolated as benzylamide **7**.

^*c*^The reaction was conducted at 50 °C.

^*d*^NMR yield.

^*e*^The reaction was conducted in the presence of **1e** (10 mol%).

^*f*^The reaction was conducted at 80 °C.

**Scheme 3:**
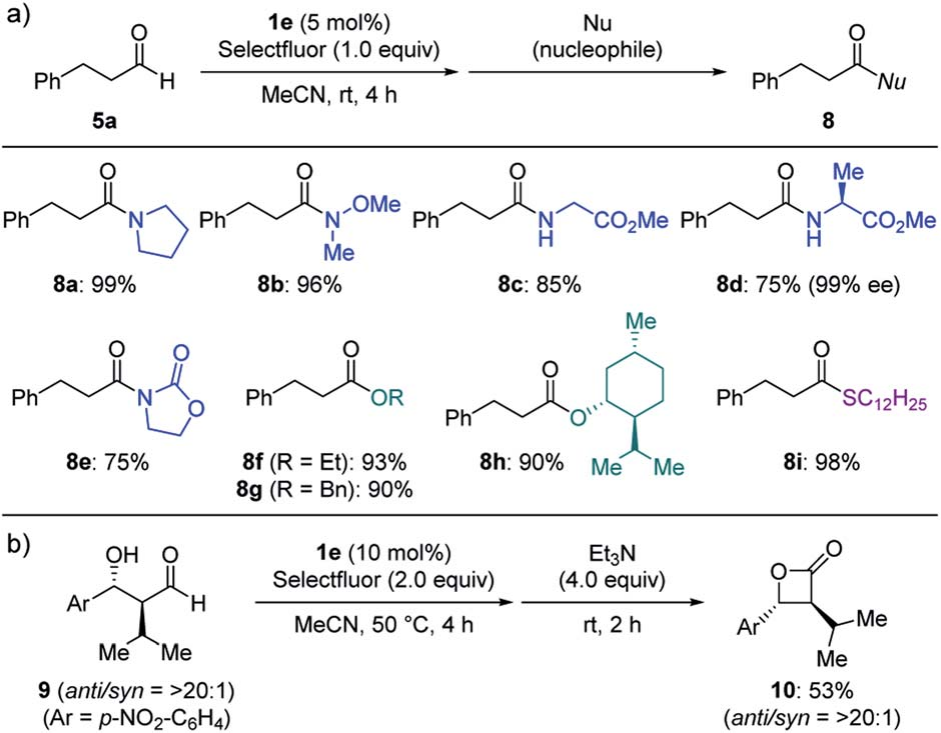
Subsequent transformations of acyl fluoride. (a) The reaction was carried out in MeCN for 4 h in the presence of **5a** (0.20 mmol), Selectfluor (1.0 equiv.) and **1e** (5 mol%), followed by a treatment with a nucleophile (2.0 equiv.) and triethylamine (0–2.0 equiv.) for 2 h. Isolated yields. (b) Synthesis of β-lactone **10**. The reaction was carried out in MeCN for 4 h in the presence of **9** (0.20 mmol), Selectfluor (2.0 equiv.) and **1e** (10 mol%), followed by a treatment with triethylamine (4.0 equiv.) for 2 h. Isolated yields.

The direct conversion of aldehydes into acyl fluorides and the subsequent reaction with various nucleophiles allow for the divergent synthesis of carbonyl compounds (Scheme 3). Other than benzylamine, secondary amines (**8a–b**), amino acid derivatives (**8c–d**) and even less nucleophilic oxazolidinone (**8e**) were also applicable to the one-pot procedure. The use of other nucleophiles such as alcohol (**8f–h**) and thiol (**8i**) provided the corresponding esters and thioester in good to high yields. This protocol was also applied to the conversion of β-hydroxy aldehyde **9** into β-lactone **10** in one pot.

**Scheme 4:**
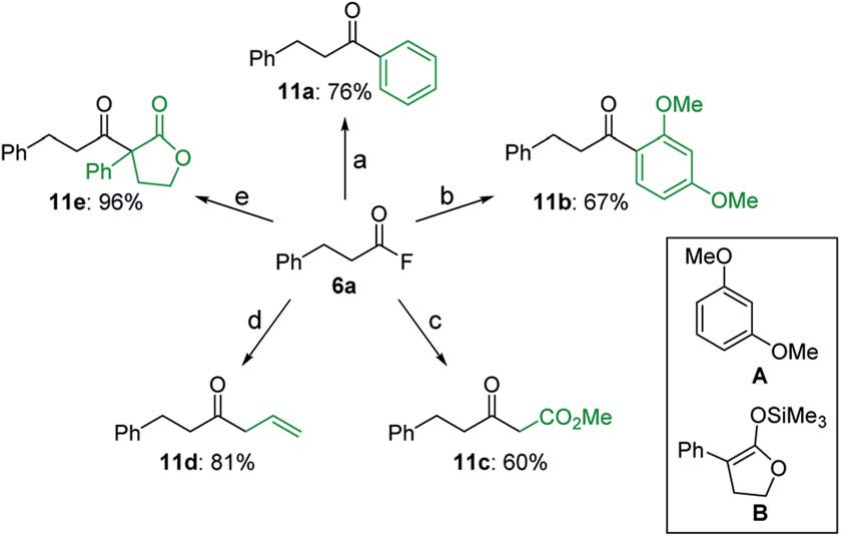
Synthesis of unsymmetrical ketones from **6a**. (a) PhB(OH)_2_ (1.5 equiv.), Pd(OAc)_2_ (1.0 mol%), P(*p*-MeO–C_6_H_4_)_3_ (4.0 mol%), KF (1.5 equiv.), toluene, 120 °C. (b) **A** (1.0 equiv.), TMSOTf (1.2 equiv.), MeCN, rt. (c) Ph_3_P=CHCO_2_Me (1.0 equiv.), KF (6.0 equiv.), MeCN, 90 °C. (d) allyltrimethylsilane (1.2 equiv.), TiCl_4_ (1.0 equiv.), CH_2_Cl_2_, –78 °C. (e) **B** (2.0 equiv.), *n*-Bu_3_SnF (2.0 equiv.), toluene, 120 °C.

Moreover, starting from isolated acyl fluoride **6a**, several unsymmetrical ketones (**11a–e**) could be synthesized *via* the reaction with various carbon nucleophiles (Scheme 4).^20^ Both the arylation of **6a** by a palladium-catalyzed cross-coupling with phenylboronic acid and the Friedel–Crafts reaction with 1,3-dimethoxybenzene (**A**) afforded aryl ketones **11a** and **11b**, respectively, in good yields. Alternatively, the reaction with a Wittig reagent furnished β-keto ester **11c**, while the use of allyltrimethylsilane afforded β,γ-unsaturated ketone **11d** in high yield. Finally, the reaction with silyl enol ether **B** in the presence of tributyltin fluoride afforded β-keto ester **11e** quantitatively. These transformations demonstrate the utility of acyl fluorides as versatile synthetic intermediates.

**Scheme 5:**
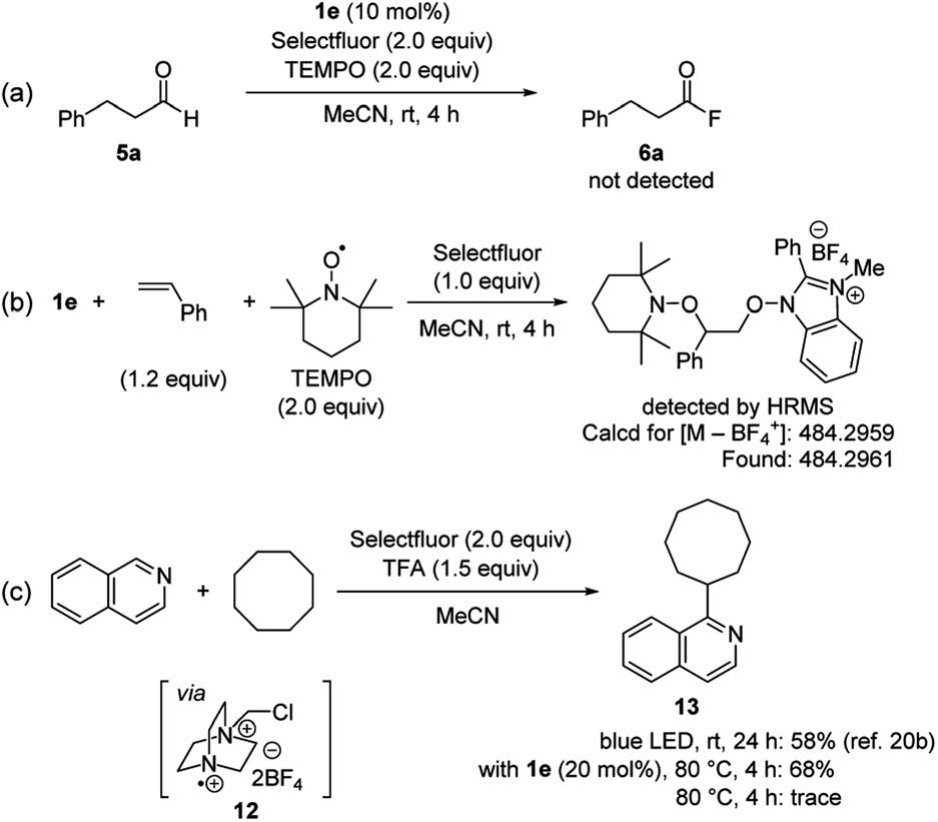
Mechanistic studies.

To better understand the reaction mechanism, several experiments were carried out (Scheme 5). The addition of 2,2,6,6-tetramethylpiperidine 1-oxyl (TEMPO) as a radical scavenger under standard conditions completely inhibited the formation of acyl fluoride (Scheme 5a). In addition, when a stoichiometric reaction of **1e** with styrene and TEMPO was run in the presence of Selectfluor, a 1 : 1 : 1 adduct of **1e**, styrene and TEMPO was detected by high-resolution mass spectrometry (Scheme 5b). On the other hand, Selectfluor is known to be activated under photoirradiation condition, generating the corresponding radical cation **12**.^21^ This species can work as a strong hydrogen atom abstraction reagent for C–H activation of various hydrocarbons.21a–c,^22^ Based on recent reports, we conducted the C–H oxidative arylation of cyclooctane with isoquinoline in the presence of a catalytic amount of **1e** (Scheme 5c).21b,c As a result, arylated product **13** could be obtained in good yield even without photoirradiation. We also confirmed **13** was not detected in the absence of **1e** and photoirradiation. Considering a high BDE value for C–H bond of cyclooctane (95.8 kcal mol^–1^, see the ESI†), a reaction pathway where the *N*-oxyl radical derived from **1e** abstracts a hydrogen atom from C–H bond of cyclooctane would not be feasible. Therefore, these results indicate that our NHBI/Selectfluor system allows for the efficient generation of highly reactive radical cation **12**, which is a key species for the activation of aldehydic C–H bond.

**Fig. 3:**
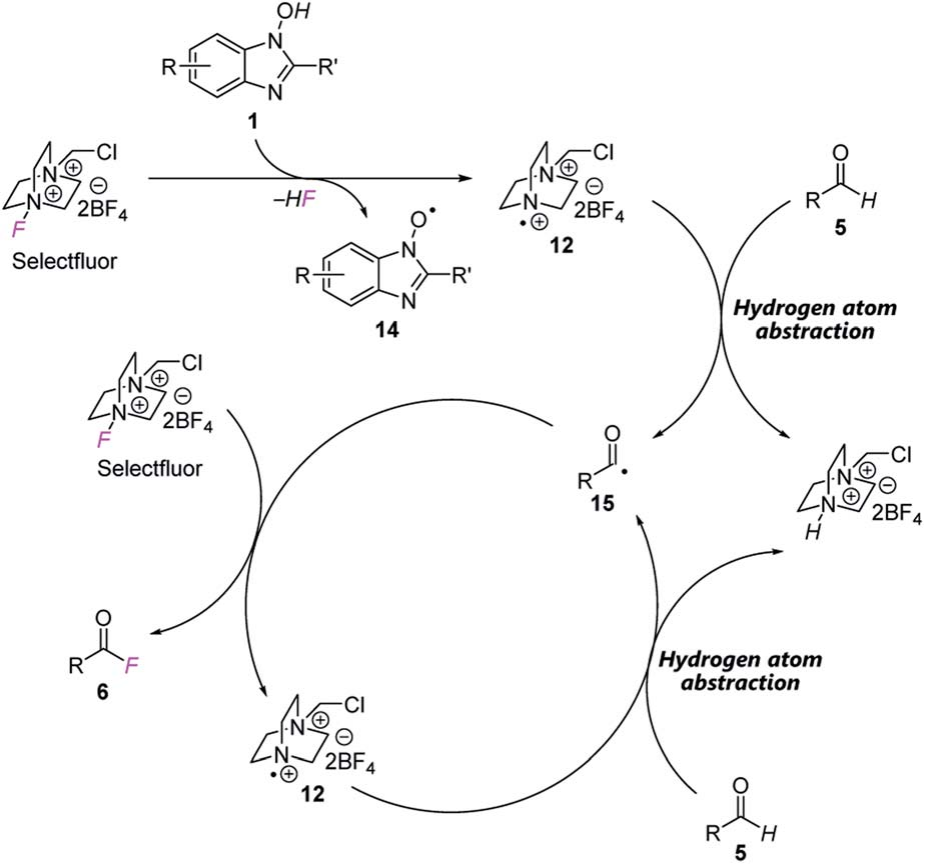
Proposed reaction mechanism.

Based on these studies, we propose a reaction mechanism as shown in Fig. 3. In the presence of NHBI **1**, radical cation **12** is generated from Selectfluor with concomitant formation of *N*-oxyl radical **14**. Then **12** abstracts a hydrogen atom from aldehyde **5** to form the corresponding acyl radical **15**. Subsequently, a fluorine atom of another Selectfluor is trapped by **15** to give the desired acyl fluoride **6** and radical cation **12**, and the reaction proceeds *via* a chain process until completion.

**Fig. 4:**
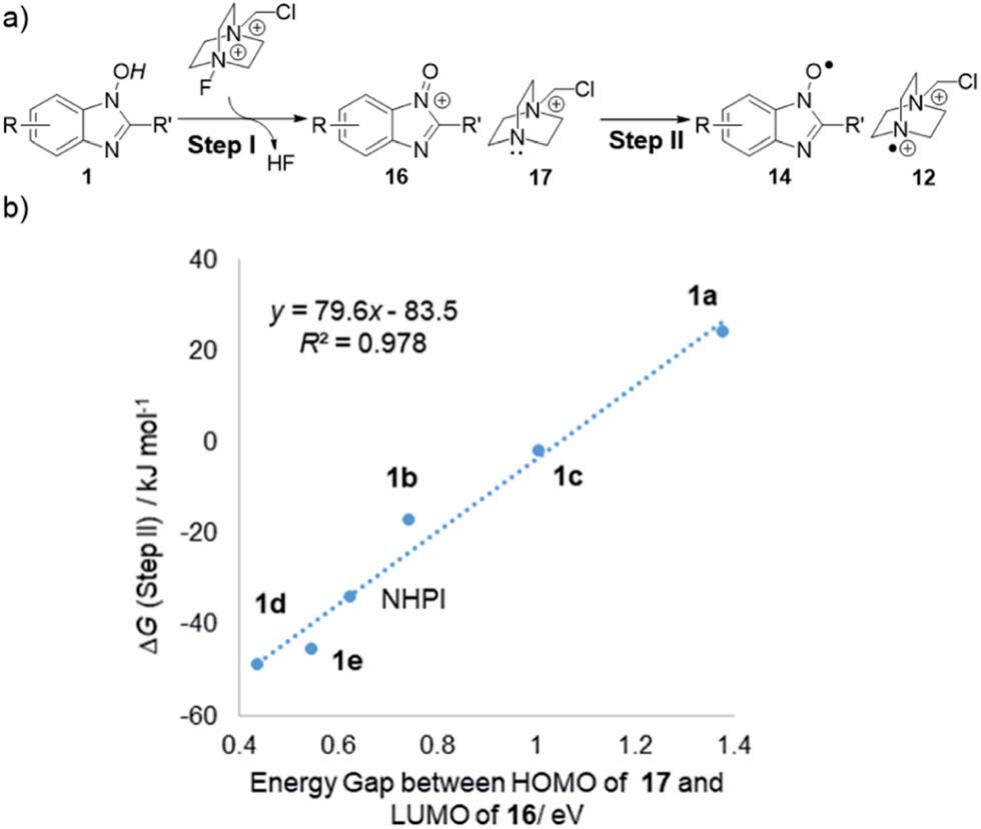
(a) Proposed mechanism for initiation process by **1** and Selectfluor. (b) Correlation of energy gap between HOMO of **17** and LUMO of **16** (in eV) and Gibbs free energy difference (Δ*G*) for step II (in kJ mol^–1^).

With respect to the initiation process generating two different radicals from **1** and Selectfluor, further theoretical studies by DFT calculation provided some insights into the remarkable reactivity difference among NHBI derivatives (Fig. 4). Namely, when this process is divided into two steps as shown in Fig. 4a, the energy gap between LUMO of putative oxoammonium **16** and HOMO of monocationic amine **17** is widely changed depending on the substitution pattern of NHBI structure. In addition, these values of HOMO–LUMO energy gap are in proportion to the Gibbs free energy difference (Δ*G*) for step II (Fig. 4b). It should be noted this correlation is not the only determinant for the efficiency of initiation process; the exceptionally lower reactivity of **1f** (Table 1, entry 7) would be attributed to an unfavorable uphill in free energy for the step I, which makes it difficult to be oxidized by Selectfluor to form the corresponding oxoammonium species leading to step II (see the ESI†). Thus, these results demonstrated that the highly designable structures of **1** allow for the tuning of their electronic state, which can alter the energy profile of the process for generation of active radical species.

## Conclusions

In conclusion, we have synthesized a novel class of *N*-oxyl radicals based on NHBI, which is a flexibly modifiable structural platform. The evaluation of these NHBI derivatives as organoradical catalysts revealed that the substituents on these structures significantly alter their catalytic performance in benzylic C–H amination reaction. Moreover, we have developed a novel metal-free and non-photolytic method for the synthesis of acyl fluorides by using a catalytic amount of NHBI derivatives. Mechanistic studies indicated a distinct character of NHBI derivatives as an efficient radical initiator to generate a more active radical species for hydrogen atom abstraction. Further investigations into the applications of NHBI derivatives to other C–H activation strategies, as well as the development of novel NHBI catalysts, are currently underway in our laboratory. We believe that these studies indicate a new direction for the chemistry of *N*-oxyl radicals, which will spur further research on organoradical catalysis for direct C–H activation reactions.

## Conflicts of interest

There are no conflicts to declare.

## Supplementary Material

Supplementary information
