# Supplementary material for: N-Hydroxybenzimidazole as a structurally modifiable platform for N-oxyl radicals for direct C–H functionalization reactions
Source: Chem Sci. 2020 May 18;11(22):5772–8. doi: 10.1039/d0sc02134b (PMC7416693; doi:10.1039/d0sc02134b)
Supplement: Supplementary file 1 [file SC-011-D0SC02134B-s001.pdf]

Electronic Supplementary Material (ESI) for Chemical Science.
This journal is © The Royal Society of Chemistry 2020

Supporting Information for

N-Hydroxybenzimidazole as Structurally Modifiable Platform of *N*-Oxyl Radicals for Direct C–H Functionalization Reactions

Tomomi Yoshii,[§] Saori Tsuzuki,[§] Shunya Sakurai,[§] Ryu Sakamoto,[§] Julong Jiang,[†] Miho Hatanaka,[†,⊥] Akira Matsumoto,[‡] and Keiji Maruoka*[,§,‡,∥]

E-mail : maruoka.keiji.4w@kyoto-u.ac.jp

[§] *Department of Chemistry, Graduate School of Science, Kyoto University, Sakyo, Kyoto 606-8502, Japan*

[†] *Institute for Research Initiatives, Division for Research Strategy, Graduate School of Materials Science, Data Science Center, Nara Institute of Science and Technology, Ikoma, Nara 630-0192, Japan*

[⊥] *PRESTO, Japan Science and Technology (JST), Kawaguchi, Saitama 332-0012, Japan*

[‡] *Graduate School of Pharmaceutical Sciences, Kyoto University, Sakyo, Kyoto 606-8501, Japan*

[∥] *School of Chemical Engineering and Light Industry, Guangdong University of Technology, Guangzhou 510006, China*

Table of Contents

1. General Information **S3**

2. Synthesis of *N*-Hydroxybenzimidazoles **S4**

3. Procedure for Benzylic C–H Amination Reaction **S15**

4. Optimization of Reaction Conditions **S16**

5. General Procedure for Aldehydic C–H Fluorination Reaction **S19**

6. General Procedure for One-pot Transformation **S22**

7. Derivatizations of Acyl Fluoride **S27**

8. Procedures for Synthesis of Unsymmetrical Ketones from Acyl Fluoride **S32**

9. Procedures for Mechanistic Studies **S35**

10. Computational Studies **S37**

11. References **S62**

12. NMR Spectra of Products and Chiral HPLC Charts **S64**

1. General Information

^{1}H-NMR spectra were measured on JEOL JNM-ECA500 (500 MHz) spectrometer. Data were reported as follows: chemical shifts in ppm from tetramethylsilane as an internal standard in $CDCl_3$, integration, multiplicity (s = singlet, d = doublet, t = triplet, q = quartet, dd = doublet-doublet, dt = doublet-triplet, dq = doublet-quartet, td = triplet-doublet, m = multiplet, app = apparent), coupling constants (Hz), and assignment. ^{13}C-NMR spectra were measured on JEOL JNM-ECA500 (125 MHz) spectrometer with complete proton decoupling. Chemical shifts were reported in ppm from the residual solvent as an internal standard. ^{19}F NMR spectra were measured on JEOL JNM-ECA500 (470 MHz) spectrometer. High-resolution mass spectra (HRMS) were performed on Thermo Exactive plus (ESI) spectrometer. For thin layer chromatography (TLC) analysis throughout this work, Merck precoated TLC plates (Merck, TLC Silica-gel 60 F_{254}) were used. The products were purified by flash column chromatography (Kanto Chemical Co., Inc., Silica-gel 60 N, spherical, neutral, 40-50 μm) or preparative thin layer chromatography silica-gel (Merck, PLC Silica-gel 60 F_{254}. 0.5 mm). Benzyl (3-oxopropyl)carbamate (**5d**)[1], 6-oxohexyl benzoate (**5e**)[2], 3-(benzyloxy)propanal (**5f**)[3], 1-tosylpiperidine-4-carbaldehyde (**5h**)[4], tetrahydro-2*H*-pyran-4-carbaldehyde (**5i**)[5], benzyl ((1*S*,2*R*)-2-methyl-3-oxo-1-phenylpropyl)carbamate (**5k**)[6], 2-(hydroxy(4-nitrophenyl)methyl)-3-methylbutanal (**9**)[7] and trimethyl((3-phenyl-4,5-dihydrofuran-2-yl)oxy)silane (**b**)[8] were prepared according to the literature procedures. Aldehydes and MeCN were used after the distillation.Commercially available reagents and solvents were purchased from FUJIFILM Wako, Sigma-Aldrich, TCI, and used as received. Raney-Ni was used for the synthesis after activated.

2. Synthesis of *N*-Hydroxybenzimidazoles

Procedure for Synthesis of 1a

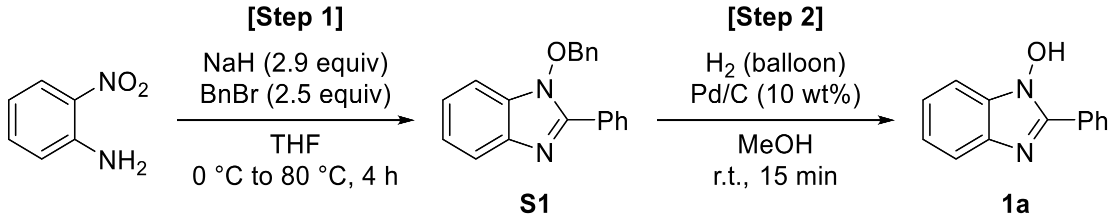

[Step 1]

To a suspension of NaH (60% in oil, 2.3 g, 58 mmol, 2.9 equiv) in THF (30 mL) was added 2-nitroaniline (2.68 g, 20 mmol) portionwise at 0 °C, and the mixture was stirred at the same temperature for 15 min. Benzyl bromide (4.9 mL, 50 mmol, 2.5 equiv) was added slowly to the solution, and the mixture was stirred at 80 °C for 4 h. The reaction mixture was cooled to room temperature, quenched with H_2O and extracted with ethyl acetate three times. The combined organic layer was dried over Na_2SO_4 and concentrated. The residue was purified by trituration with hexane and filtration to afford the following compound.

1-(Benzyloxy)-2-phenyl-1*H*-benzo[d]imidazole (S1)

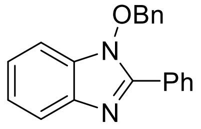

White solid; 4.4 g, 97%.
^{1}H NMR (500 MHz, $CDCl_3$) δ 8.18–8.16 (m, 2H), 7.80–7.79 (m, 1H), 7.49 (m, 3H), 7.45 (m, 1H), 7.34 (m, 1H), 7.30 (m, 4H), 7.23 (m, 2H), 5.04 (s, 2H); **^{13}C NMR** (125 MHz, $CDCl_3$) δ 147.8, 138.7, 133.0, 131.6, 130.3, 130.0, 129.7, 128.83, 128.80, 128.77, 128.6, 123.5, 123.0, 120.5, 109.0, 80.3; **HRMS (ESI)** calculated for $C_{20}H_{17}ON_2$: *m/z* 301.1335 ([M + H]$^+$), found: *m/z* 301.1346 ([M + H]$^+$); **IR (neat)** 3053, 905, 758, 689 cm^{-1}.

[Step 2]

To a solution of **S1** (1.4 g, 4.8 mmol) in MeOH (20 mL) was added Pd/C (140 mg, 10 wt%), and the mixture was stirred under a H_2 atmosphere at room temperature for 15 min. The reaction mixture was filtered through high-flow celite, and the filtrate was concentrated to afford the following compound without further purification.

2-Phenyl-1*H*-benzo[d]imidazol-1-ol (1a)

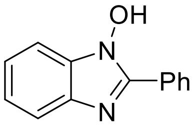

White solid; 560 mg, 56%.

¹H NMR (500 MHz, DMSO-d_6) δ 12.04 (br s, 1H), 8.26 (d, J = 6.5 Hz, 2H), 7.66 (d, J = 7.9 Hz, 1H), 7.55 (m, 4H), 7.30 (t, J = 7.5 Hz, 1H), 7.24 (t, J = 7.7 Hz, 1H); **¹³C NMR** (125 MHz, DMSO-d_6) δ 146.9, 137.8, 133.5, 129.9, 128.6, 128.2, 126.5, 122.8, 122.2, 119.2, 109.2; **HRMS (ESI)** calculated for $C_{13}H_9ON_2$: *m/z* 209.0709 ($[M - H]^-$ found: *m/z* 209.0714 ($[M - H]^-$); **IR (neat)** 3352, 2377, 1521, 1141, 736 cm^{-1}.

Procedure for Synthesis of 1c

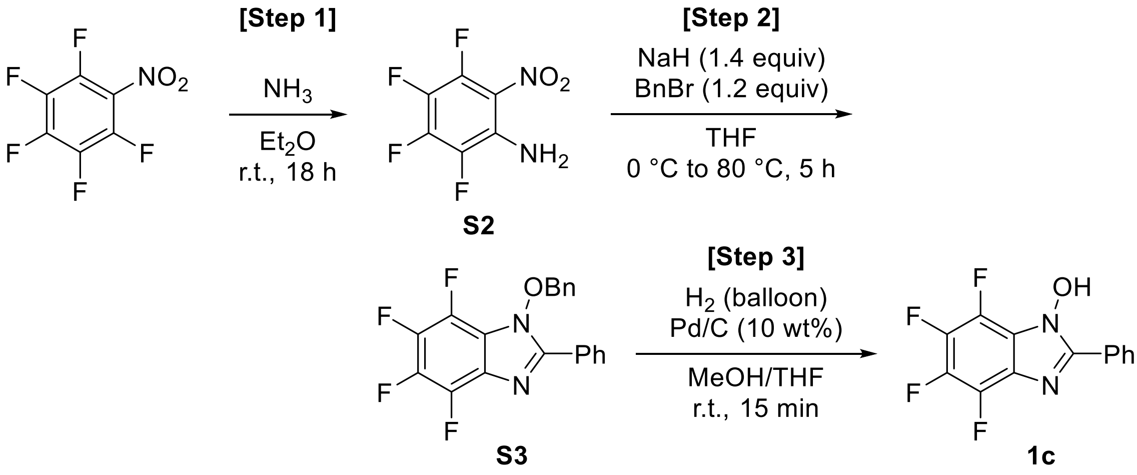

[Step 1]

Dry ammonia gas was passed through a solution of pentafluoronitrobenzene (3.0 g, 14 mmol) in Et_2O (160 mL) at room temperature for 3 h. The mixture was stirred for further 18 h and then filtered to remove the precipitated ammonium fluoride. The filtrate was washed with H_2O, dried over Na_2SO_4 and concentrated. The residue was purified by flash column chromatography on silica gel to afford the following compound.

2,3,4,5-Tetrafluoro-6-nitroaniline (S2)

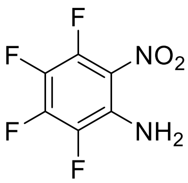

Yellow crystal; 1.5 g, 50%.

¹³C NMR (125 MHz, $CDCl_3$) δ 144.3 (dtd, J_{C-F} = 260.3, 13.9, 4.4 Hz), 143.9 (dtd, J_{C-F} = 262.3, 8.9, 4.4 Hz), 136.3 (dd, J_{C-F} = 243.2, 13.1 Hz),

132.4 (d, J_{C-F} = 11.9 Hz), 132.3 (dt, J_{C-F} = 244.8, 16.1 Hz), 121.2; **^{19}F NMR** (470 MHz, $CDCl_3$) δ –145.1 (dt, J = 22.6, 8.9 Hz), –147.2 (td, J = 21.3, 8.8 Hz), –160.2 (ddd, J = 20.6, 9.0, 5.9 Hz), –172.5 (td, J = 22.3, 5.9 Hz); **HRMS (ESI)** calculated for $C_6HO_2N_2F_4$: *m/z* 208.9969 ([M – H]$^-$), found: *m/z* 208.9972 ([M – H]$^-$); **IR (neat)** 3494, 3374, 1518, 1516, 1118, 997 cm^{-1}.

[Step 2]

To a suspension of NaH (60% in oil, 116 mg, 2.9 mmol, 1.4 equiv) in THF (20 mL) was added **S2** (440 mg, 2.1 mmol) portionwise at 0 °C, and the mixture was stirred for 15 min. Benzyl bromide (0.24 mL, 2.5 mmol, 1.2 equiv) was added slowly to the solution, and the mixture was stirred at 80 °C for 5 h. The reaction mixture was cooled to room temperature, quenched with H_2O and extracted with ethyl acetate three times. The combined organic layer was dried over Na_2SO_4 and concentrated. The residue was purified by flash column chromatography on silica gel to afford the following compound.

1-(Benzyloxy)-4,5,6,7-tetrafluoro-2-phenyl-1*H*-benzo[d]imidazole (S3)

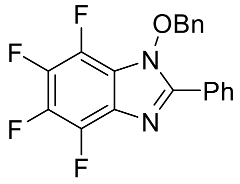

Orange solid; 352 mg, 45%.

^{1}H NMR (500 MHz, $CDCl_3$) δ 8.11 (d, J = 7.9 Hz, 2H), 7.55–7.48 (m, 3H), 7.35 (t, J = 6.8 Hz, 1H), 7.29–7.26 (m, 2H), 7.18 (d, J = 7.7 Hz, 2H), 5.07 (s, 2H); **^{13}C NMR** (125 MHz, $CDCl_3$) δ 150.5, 138.9 (dd, J_{C-F} = 253.9, 10.7 Hz), 138.1 (dt, J_{C-F} = 247.2, 14.6 Hz), 137.5 (dt, J_{C-F} = 245.2, 14.0 Hz), 133.0 (dd, J_{C-F} = 249.7, 13.7 Hz), 131.7, 131.2, 130.4, 130.1, 128.9, 128.87, 128.86, 127.2, 124.7 (d, J_{C-F} = 16.7 Hz), 117.2 (q, J_{C-F} = 7.2 Hz), 82.2; **^{19}F NMR** (470 MHz, $CHCl_3$) δ –154.8 (dd, J = 19.6, 17.3 Hz), –162.1 (t, J = 20.3 Hz), –163.5–-163.4 (m), –164.6 (td, J = 19.8, 3.4 Hz); **HRMS (ESI)** calculated for $C_{20}H_{12}ON_2F_4Na$: *m/z* 395.0778 ([M + Na]$^+$), found: *m/z* 395.0779 ([M + Na]$^+$); **IR (neat)** 2924, 1547, 1317, 1007 cm^{-1}.

[Step 3]

To a solution of **S3** (31.3 mg, 0.084 mmol) in MeOH (2.0 mL) and THF (2.0 mL) was added Pd/C (3.1 mg, 10 wt%) and the mixture was stirred under a H_2 atmosphere at room temperature for 15 min. The reaction mixture was filtered through high-flow celite and

the filtrate was concentrated to afford the following compound without further purification.

4,5,6,7-Tetrafluoro-2-phenyl-1*H*-benzo[d]imidazol-1-ol (1c)

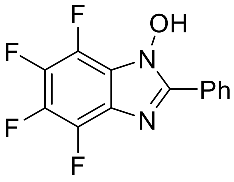

Brown solid; 22 mg, 94%.

^{1}H NMR (500 MHz, CD_3OD) δ 8.18–8.17 (m, 2H), 7.58–7.57 (m, 3H); **^{13}C NMR** (125 MHz, CD_3OD) δ 152.7, 132.2, 130.0, 129.8, 128.4, 125.4 (d, $J_{C\text{–}F}$ = 15.5 Hz), 120.5 (q, $J_{C\text{–}F}$ = 7.2 Hz) (Other peaks were not detected due to C–F coupling.); **^{19}F NMR** (470 MHz, CD_3OD) δ –159.24–-159.31 (m), –166.55–-166.63 (m), –168.96–-169.05 (m); **HRMS (ESI)** calculated for $C_{13}H_5ON_2F_4$: *m/z* 281.0333 ([M – H]$^-$), found: *m/z* 281.0343 ([M – H]$^-$); **IR (neat)** 3438, 1545, 1011, 692 cm^{-1}.

Procedure for Synthesis of 1b

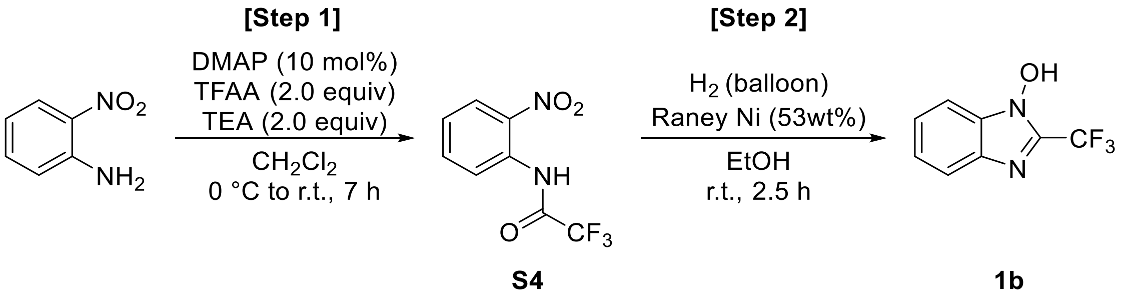

[Step 1]

A solution of 2-nitroaniline (2.76 g, 20 mmol) in CH_2Cl_2 (80 mL) was cooled to 0 °C and stirred for 30 min. To the solution were added 4-dimethylaminopyridine (244 mg, 2.0 mmol, 10 mol%), triethylamine (5.64 mL, 40 mmol, 2.0 equiv) and trifluoroacetic anhydride (5.64 mL, 40 mmol, 2.0 equiv), and the mixture was stirred at room temperature for 7 h. The reaction mixture was quenched with H_2O and extracted with ethyl acetate three times. The combined organic layer was dried over Na_2SO_4 and concentrated. The residue was purified by flash column chromatography on silica gel to afford the following compound.

2,2,2-Trifluoro-*N*-(2-nitrophenyl)acetamide (S4)

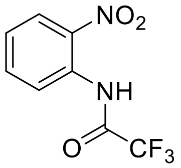

Yellow solid; 4.7 g, quant.

^{1}H NMR (500 MHz, $CDCl_3$) δ 11.39 (br s, 1H), 8.75 (dd, J = 8.4, 1.3 Hz, 1H), 8.33 (dd, J = 8.5, 1.4 Hz, 1H), 7.79–7.75 (m, 1H), 7.39–7.36 (m, 1H); **^{13}C NMR** (125 MHz, $CDCl_3$) δ 155.5 (q, J = 38.1 Hz), 137.2, 136.5, 132.2, 126.3, 125.7, 122.3, 115.5 (q, J = 288.9 Hz); **^{19}F NMR** (470 MHz, $CDCl_3$) δ –76.1; **HRMS (ESI)** calculated for $C_8H_4O_3N_2F_3$: *m/z* 233.0169 ($[M – H]^–$), found: *m/z* 233.0176 ($[M – H]^–$); **IR (neat)** 3298, 1728, 11138, 763, 671cm$^{–1}$.

[Step 2]

To a solution of **S4** (1.17 g, 5.0 mmol) in EtOH (20 mL) was added Raney-Ni (625 mg, 53 wt%), and the mixture was stirred under a H_2 atmosphere at room temperature for 2.5 h. The reaction mixture was filtered through high-flow celite, and the filtrate was concentrated. The residue was purified by flash column chromatography on silica gel to afford the following compound.

2-(Trifluoromethyl)-1*H*-benzo[d]imidazol-1-ol (1b)

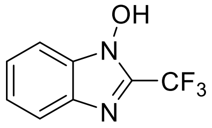

White solid; 725 mg, 72%.

^{1}H NMR (500 MHz, CD_3OD) δ 7.75 (d, J = 8.2 Hz, 1H), 7.63 (d, J = 8.2 Hz, 1H), 7.50 (app t, 1H), 7.40 (app t, 1H); **^{13}C NMR** (125 MHz, CD_3OD) δ 138.2 (q, $J_{C–F}$ = 39.7 Hz), 137.9, 134.0, 126.9, 125.1, 121.6, 119.9 (q, $J_{C–F}$ = 270.2 Hz), 110.9; **^{19}F NMR** (470 MHz, CD_3OD) δ –65.1; **HRMS (ESI)** calculated for $C_8H_4ON_2F_3$: *m/z* 201.0270 ($[M – H]^–$), found: *m/z* 201.0274 ($[M – H]^–$); **IR (neat)** 3371, 2486, 1210, 1125, 738 cm$^{–1}$.

Procedure for Synthesis of 1d

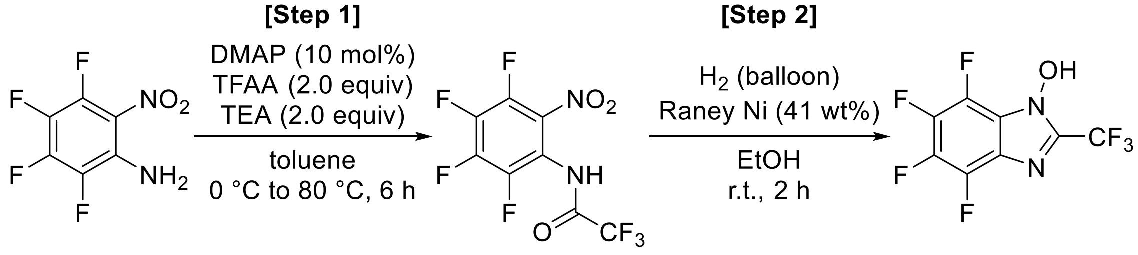

S5 **1d**

[Step 1]

A solution of 2,3,4,5-tetrafluoro-6-nitroaniline (210 mg, 1.0 mmol) in toluene (4.0 mL) was cooled to 0 °C and stirred for 30 min. To the mixture were added 4-dimethylaminopyridine (12 mg, 0.1 mmol, 10 mol%), triethylamine (280 μL, 2.0 mmol, 2.0 equiv) and trifluoroacetic anhydride (280 μL, 2.0 mmol, 2.0 equiv), and the mixture was stirred at 80 °C for 6 h. The reaction mixture was quenched with H_2O and extracted with ethyl acetate three times. The combined organic layer was dried over Na_2SO_4 and concentrated. The residue was purified by flash column chromatography on silica gel to afford the following compound.

2,2,2-Trifluoro-*N*-(2,3,4,5-tetrafluoro-6-nitrophenyl)acetamide (S5)

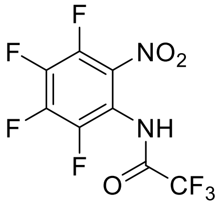

White solid; 252 mg, 82%.
^{13}C NMR (125 MHz, $CDCl_3$) δ 155.7 (q, J = 40.1 Hz), 144.0 (dt, J = 264.6, 13.7 Hz), 143.4 (dd, J = 261.1, 11.9 Hz), 142.6 (dd, J = 265.8, 13.1 Hz), 140.8 (dt, J = 261.5, 13.7 Hz), 131.9, 115.2 (q, J = 287.7 Hz), 114.3 (d, J = 15.5 Hz); **^{19}F NMR** (470 MHz, $CDCl_3$) δ –75.1, –135.60–135.64 (m), –142.18 (m), –144.07 (m), –150.19–150.28 (m); **HRMS (ESI)** calculated for $C_8O_3N_2F_7$: *m/z* 304.9792 ([M – H]$^-$), found: *m/z* 304.9803 ([M – H]$^-$); **IR (neat)** 3293, 1739, 1558, 1139 cm^{-1}.

[Step 2]

To a solution of **S5** (2.5 g, 8.2 mmol) in EtOH (36 mL) was added Raney-Ni (1.03 g, 41wt%), and the mixture was stirred under a H_2 atmosphere at room temperature for 2 h. The reaction mixture was filtered through high-flow celite and the filtrate was

concentrated. The residue was purified by flash column chromatography on silica gel to afford the following compound.

4,5,6,7-Tetrafluoro-2-(trifluoromethyl)-1*H*-benzo[d]imidazol-1-ol (1d)

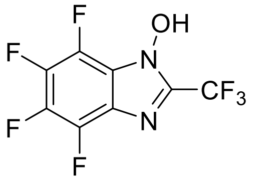

White solid; 663 mg, 66%.

^{13}C NMR (125 MHz, $CDCl_3$); Peaks were not detected due to C–F coupling; **^{19}F NMR** (470 MHz, $CDCl_3$) δ –63.8, –152.3, –157.2, –161.8, –162.7; **HRMS (ESI)** calculated for $C_8ON_2F_7$: *m/z* 272.9893 ($[M-H]^-$), found: *m/z* 272.9905 ($[M-H]^-$); **IR (neat)** 2592, 1554, 1198, 1004, 744 cm^{-1}.

Procedure for Synthesis of 1h and 1e

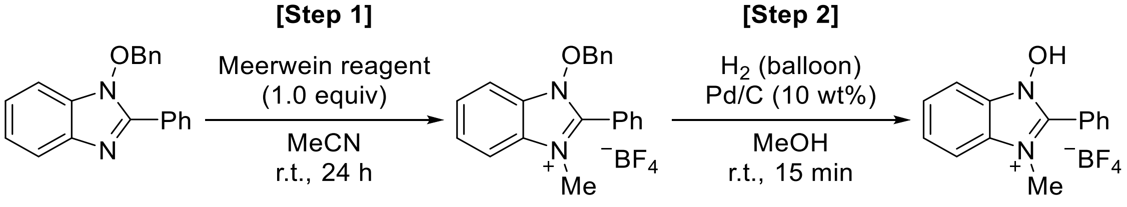

[Step 1]

To a solution of **S1** (601 mg, 2.0 mmol) in MeCN (20 mL) was added Meerwein reagent (296 mg, 2.0 mmol, 1.0 equiv), and the mixture was stirred under an argon atmosphere at room temperature for 24 h. The reaction mixture was filtered through high-flow celite and the filtrate was concentrated. The residue was purified by trituration with Et_2O and filtration to afford the following compound.

1-(Benzyloxy)-3-methyl-2-phenyl-1*H*-benzo[d]imidazol-3-ium Tetrafluoroborate (1h)

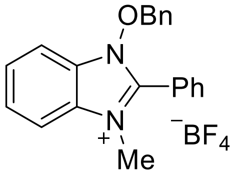

White solid; 651 mg, 81%.

^{1}H NMR (500 MHz, DMSO-d_6) δ 8.21–8.18 (m, 1H), 8.08–8.04 (m, 1H), 7.94 (d, J = 7.1 Hz, 2H), 7.87 (t, J = 7.5 Hz, 1H), 7.83–7.76 (m, 4H), 7.39 (t, J = 7.5 Hz, 1H), 7.31 (t, J = 7.7 Hz, 2H), 7.16 (d, J = 7.1 Hz, 2H), 5.32 (s, 2H), 3.99 (s, 3H); **^{13}C NMR** (125 MHz, DMSO-d_6) δ 147.3, 133.4, 132.0, 131.0, 130.3, 130.0, 129.5, 129.4, 128.8, 127.5, 127.34, 127.31, 119.5, 113.9, 111.8, 82.3,

33.1; **^{19}F NMR** (470 MHz, DMSO-*d*$_6$) δ –148.5 (q, *J* = 1.1 Hz); **HRMS (ESI)** calculated for $C_{21}H_{19}ON_2$: *m/z* 315.1492 ([M – BF_4]$^+$), found: *m/z* 315.1513 ([M – BF_4]$^+$); **IR (neat)** 3432, 2538, 1461, 752, 698 cm^{-1}.

[Step 2]

To a solution of **1h** (650 mg, 1.6 mmol) in MeOH (5.0 mL) was added Pd/C (65 mg, 10 wt%), and the mixture was stirred under a H_2 atmosphere at room temperature for 15 min. The reaction mixture was filtered through high-flow celite, and the filtrate was concentrated to afford the following compound without further purification.

1-Hydroxy-3-methyl-2-phenyl-1*H*-benzo[d]imidazol-3-ium Tetrafluoroborate (1e)

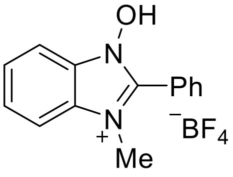

White solid; 367 mg, 73%.
^{1}H NMR (500 MHz, DMSO-*d*$_6$) δ 8.13 (d, *J* = 7.4 Hz, 1H), 7.96–7.91 (m, 3H), 7.81–7.68 (m, 5H), 3.96 (s, 3H); **^{13}C NMR** (125 MHz, DMSO-*d*$_6$) δ 145.9, 133.0, 131.0, 129.4, 129.2, 128.9, 127.0, 126.9, 120.1, 113.5, 111.6, 33.0; **^{19}F NMR** (470 MHz, DMSO-*d*$_6$) δ –148.4; **HRMS (ESI)** calculated for $C_{14}H_{13}ON_2$: *m/z* 225.1022 ([M – BF_4]$^+$), found: *m/z* 225.1018 ([M – BF_4]$^+$); **IR (neat)** 3385, 2504, 1463, 1055, 751, 697 cm^{-1}.

Procedure for Synthesis of 1f

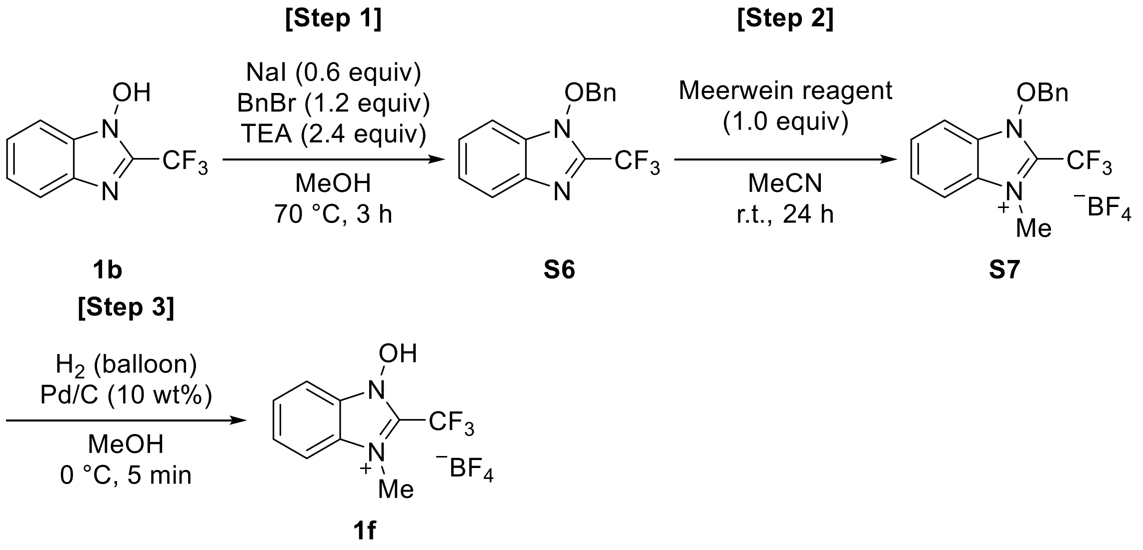

[Step 1]

To a solution of **1b** (404 mg, 2.0 mmol) in MeOH (40 mL) were added sodium iodide (179 mg, 1.2 mmol, 0.6 equiv), benzyl bromide (240 μL, 2.4 mmol, 1.2 equiv) and triethylamine (0.67 mL, 4.8 mmol, 2.4 equiv), and the mixture was stirred under an argon atmosphere at 70 °C for 3 h. The reaction mixture was cooled to room temperature, quenched with H_2O and extracted with ethyl acetate three times. The combined organic layer was dried over Na_2SO_4 and concentrated. The residue was purified by flash column chromatography on silica gel to afford the following compound.

1-(Benzyloxy)-2-(trifluoromethyl)-1*H*-benzo[d]imidazole (S6)

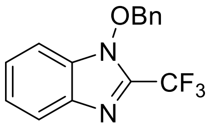

White solid; 488 mg, 84%.

^{1}H NMR (500 MHz, $CDCl_3$) δ 7.85 (d, J = 7.1 Hz, 1H), 7.49–7.44 (m, 5H), 7.41–7.35 (m, 2H), 7.31 (d, J = 7.1 Hz, 1H), 5.34 (s, 2H); **^{13}C NMR** (125 MHz, $CDCl_3$) δ 136.9, 136.6 (q, $J_{C–F}$ = 40.1 Hz), 132.7, 131.2, 129.80, 129.77, 128.8, 125.8, 123.9, 121.8, 118.5 (q, $J_{C–F}$ = 271.0 Hz), 109.6, 82.1; **^{19}F NMR** (470 MHz, $CDCl_3$) δ –63.4; **HRMS (ESI)** calculated for $C_{15}H_{12}ON_2F_3$: *m/z* 293.0896 ([M + H]$^+$), found: *m/z* 293.0901 ([M + H]$^+$); **IR (neat)** 3036, 1530, 1280, 729, 695 cm^{-1}.

[Step 2]

To a solution of **S6** (585 mg, 2.0 mmol) in MeCN (20 mL) was added Meerwein reagent (296 mg, 2.0 mmol, 1.0 equiv), and the mixture was stirred under an argon atmosphere at room temperature for 24 h. The reaction mixture was then concentrated, and the residue was purified by trituration with ethyl acetate and filtration to afford the following compound.

1-(Benzyloxy)-3-methyl-2-(trifluoromethyl)-1*H*-benzo[d]imidazol-3-ium Tetrafluoroborate (S7)

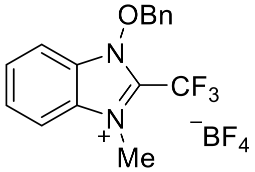

White solid; 449 mg, 57%.

^{1}H NMR (500 MHz, CD_3CN) δ 8.09 (d, J = 8.5 Hz, 1H), 7.93–7.88 (m, 1H), 7.87–7.86 (m, 2H), 7.62–7.61 (m, 2H), 7.56–7.50 (m, 3H), 5.64 (s, 2H), 4.26 (s, 3H); **^{13}C NMR** (125 MHz, CD_3CN) δ 134.28

(q, $J_{C–F}$ = 43.3 Hz), 132.6, 131.81, 131.76, 131.0, 130.9, 130.2, 130.1, 129.0, 117.28 (q, $J_{C–F}$ = 276.0 Hz), 115.4, 113.7, 86.3, 35.4; **^{19}F NMR** (470 MHz, CD_3CN) δ –60.0, –151.8; **HRMS (ESI)** calculated for $C_{16}H_{14}ON_2F_3$: *m/z* 307.1053 ($[M - BF_4]^+$), found: *m/z* 307.1058 ($[M - BF_4]^+$); **IR (neat)** 3466, 1628, 1320, 1062, 775 cm^{-1}.

[Step 3]

To a solution of **S7** (79 mg, 0.2 mmol) in MeOH (2.0 mL) was added Pd/C (7.9 mg, 10 wt%), and the mixture was stirred under a H_2 atmosphere at 0 °C for 5 min. The reaction mixture was filtered through high-flow celite, and the filtrate was concentrated. The residue was purified by trituration with Et_2O and filtration to afford the following compound.

1-Hydroxy-3-methyl-2-(trifluoromethyl)-1*H*-benzo[d]imidazol-3-ium Tetrafluoroborate (1f)

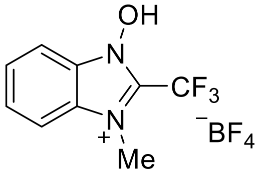

White solid; 35 mg, 57%.

^{1}H NMR (500 MHz, CD_3OD) δ 8.04 (d, *J* = 8.5 Hz, 1H), 8.00 (d, *J* = 8.2 Hz, 1H), 7.83 (t, *J* = 7.5 Hz, 1H), 7.78 (t, *J* = 7.4 Hz, 1H), 4.22 (s, 3H); **^{13}C NMR** (125 MHz, CD_3OD) δ 132.8 (q, $J_{C–F}$ = 40.9 Hz), 131.5, 130.4, 129.2, 129.0, 118.5 (q, $J_{C–F}$ = 273.4 Hz), 114.5, 113.9, 34.0; **^{19}F NMR** (470 MHz, CD_3OD) δ –61.4, –155.0; **HRMS (ESI)** calculated for $C_9H_8ON_2F_3$: *m/z* 217.0583 ($[M - BF_4]^+$), found: *m/z* 217.0585 ($[M - BF_4]^+$); **IR (neat)** 3379, 1652, 1525, 1093, 904, 751 cm^{-1}.

Procedure for Synthesis of 1g

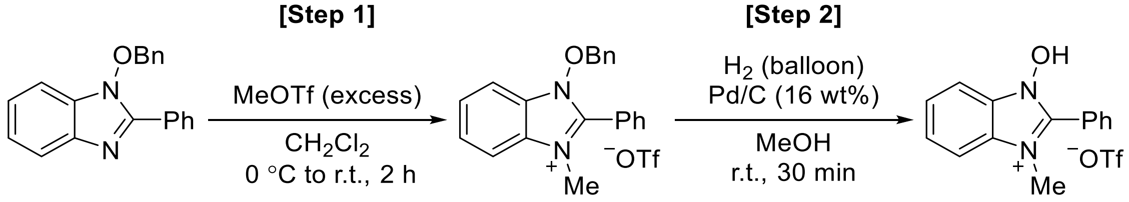

S1 S8 1g

[Step 1]

To a solution of **S1** (200 mg, 0.67 mmol) in CH_2Cl_2 (1.0 mL) was added methyl trifluoromethanesulfonate (1.0 mL) dropwise at 0 °C, and the mixture was stirred under

an argon atmosphere at room temperature for 2 h. The reaction mixture was concentrated, and the residue was purified by trituration with Et_2O and filtration to afford the following compound.

1-(Benzyloxy)-3-methyl-2-phenyl-1*H*-benzo[d]imidazol-3-ium Trifluoromethanesulfonate (S8)

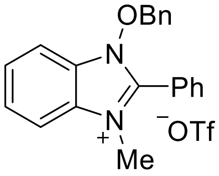

White solid; 307 mg, quant.

^{1}H NMR (500 MHz, DMSO-d_6) δ 8.24–8.19 (m, 1H), 8.10–8.06 (m, 1H), 7.91–7.90 (m, 2H), 7.86 (t, J = 7.5 Hz, 1H), 7.83–7.75 (m, 4H), 7.40 (t, J = 7.5 Hz, 1H), 7.30 (t, J = 7.7 Hz, 2H), 7.14 (d, J = 7.1 Hz, 2H), 5.30 (s, 2H), 3.97 (s, 3H); **^{13}C NMR** (125 MHz, DMSO-d_6) δ 147.3, 133.4, 131.9, 131.0, 130.3, 130.0, 129.3, 128.7, 127.4, 127.3, 127.2, 122.0, 119.4, 113.9, 111.8, 82.1, 33.1; **^{19}F NMR** (470 MHz, DMSO-d_6) δ –77.7; **HRMS (ESI)** calculated for $C_{21}H_{19}ON_2$: *m/z* 315.1492 ([M – OTf]$^+$), found: *m/z* 315.1495 ([M – OTf]$^+$); **IR (neat)** 3062, 1475,1258, 751, 636 cm^{-1}.

[Step 2]

To a solution of **S8** (307 mg, 0.67 mmol) in MeOH (3.0 mL) was added Pd/C (50 mg, 16 wt%), and the mixture was stirred under a H_2 atmosphere at room temperature for 30 min. The reaction mixture was concentrated, and the residue was purified by trituration with Et_2O and filtration to afford the following compound.

1-Hydroxy-3-methyl-2-phenyl-1*H*-benzo[d]imidazol-3-ium Trifluoromethanesulfonate (1g)

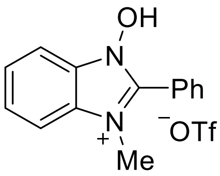

White solid; 173 mg, 69%.

^{1}H NMR (500 MHz, DMSO-d_6) δ 8.03 (d, J = 8.5 Hz, 1H), 7.81 (d, J = 7.7 Hz, 2H), 7.72–7.59 (m, 6H), 3.89 (s, 3H); **^{13}C NMR** (125 MHz, DMSO-d_6) δ 144.4, 132.4, 130.6, 129.4, 129.3, 128.9, 126.6, 126.0, 120.4, 113.1, 111.6, 32.8; **^{19}F NMR** (470 MHz, DMSO-d_6) δ –77.7; **HRMS (ESI)** calculated for $C_{21}H_{19}ON_2$: *m/z* 315.1492 ([M – OTf]$^+$), found: *m/z* 315.1495 ([M – OTf]$^+$); **IR (neat)** 3062, 1475,1258, 751, 636 cm^{-1}.

3. Procedure for Benzylic C–H Amination Reaction

A vial with a magnetic stir bar was charged with catalyst (0.020 mmol, 10 mol%), ethylbenzene (**2**, 25 μL, 0.20 mmol) and 1,2-dichloroethane (1.5 mL) under an argon atmosphere. To the solution was added diethyl azodicarboxylate (**3**, 63 μL, 0.40 mmol, 2.0 equiv), and the mixture was stirred at 80 °C for 24 h. After cooling to room temperature, the reaction mixture was concentrated and the residue was analyzed by ^{1}H NMR using 1,1,2,2-tetrachloroethane as an internal standard. the crude product was then purified by flash column chromatography on silica gel (eluting with hexane/ ethyl acetate = 3/1) to afford the following compound.

Diethyl 1-(1-Phenylethyl)hydrazine-1,2-dicarboxylate (4)[9]

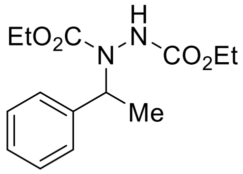

Colorless oil; 93% NMR yield; 50 mg, 90 % isolated yield. **^{1}H NMR** (500 MHz, $CDCl_3$) δ 7.34–7.27 (m, 5H), 6.05 (br s, 1H), 5.51 (br s, 1H), 4.23–4.10 (m, 4H), 1.56 (s, 3H), 1.26 (t, J = 2.4 Hz, 6H); **^{13}C NMR** (125 MHz, $CDCl_3$) δ 156.8, 155.9, 140.7, 128.6, 127.8, 127.3, 62.6, 62.0, 56.6, 16.8, 14.6, 14.5.

4. Optimization of Reaction Conditions

Table S-1. Effects of Fluorinating Agents

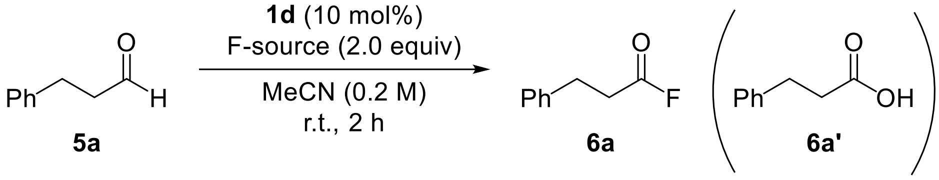

Entry	F-source	Recovered **5a** (%)[a]	**6a** Yield (%)[a]	**6a'** Yield (%)[a]
1	Selectfluor	8	61	16
2	**A**	0	62	10
3	NFSI	53	15	19
4	**B**	95	<1	0
5	**C**	>99	<1	0
6	**D**	>99	<1	0

[a]Yields were determined by ^1H NMR spectroscopy using benzotrifluoride as an internal standard.

F-Source

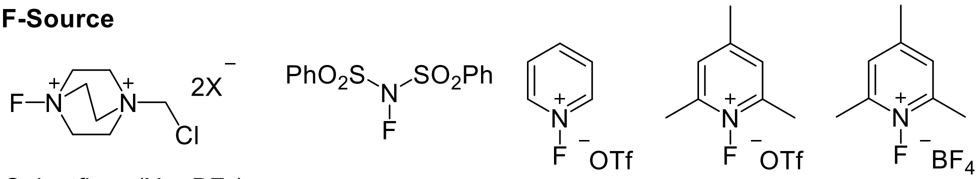

Selectfluor (X = BF_4)
A (X = PF_6)

NFSI

B

C

D

Table S-2. Effects of Solvents and Temperature

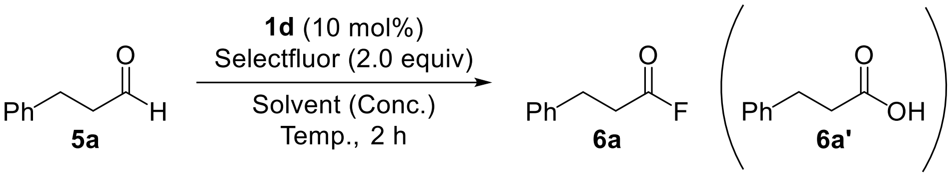

Entry	Solvent	Conc.	Temp.	Recovered **5a** (%)[a]	**6a** Yield (%)[a]	**6a'** Yield (%)[a]
1	MeCN	0.2 M	r.t.	8	61	16
2	EtCN	0.2 M	r.t.	79	<1	16
3	benzene	0.2 M	r.t.	62	<1	28
4	DCE	0.2 M	r.t.	80	<1	20
5	DMSO	0.2 M	r.t.	>99	<1	<1
6	DMF	0.2 M	r.t.	83	<1	<1
7	MeCN	0.4 M	r.t.	23	42	21
8	MeCN	0.1 M	r.t.	5	62	9
9	MeCN	0.1 M	0 °C	71	7	10
10	MeCN	0.1 M	50 °C	<1	56	8

[a]Yields were determined by ^{1}H NMR spectroscopy using benzotrifluoride as an internal standard.

Table S-3. Optimizations for Fluorination and One-pot Amidation

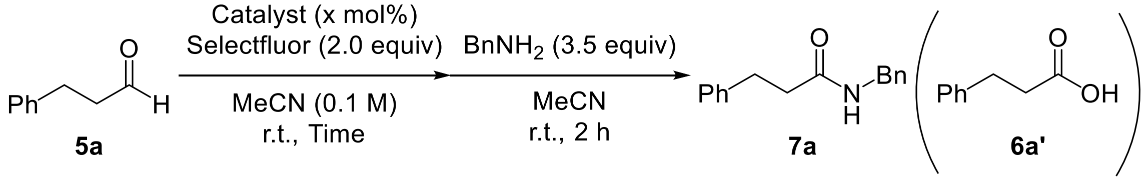

Entry	Catalyst	x (mol%)	Time	**7a** Yield (%)[a,b]	**6a'** Yield (%)[a]
1	**1d**	10	2 h	82 (78)	9
2	**1e**	10	2 h	93 (85)	<1
3	**1e**	5	2 h	57	18
4	**1e**	1	20 h	17	83
5	**1e**	5	4 h	78	11
6[c]	**1e**	5	4 h	97 (99)	<1
7[c,d]	**1e**	5	4 h	99	<1
8[c,d,e]	**1e**	5	4 h	99 (quant.)	<1

[a]Yields were determined by ^{1}H NMR spectroscopy using 1,1,2,2-tetrachloroethane as an internal standard. [b]The value in parentheses is isolated yield. [c]Schlenk flask was used. [d]Selectfluor (1.0 equiv) was used. [e]$BnNH_2$ (2.0 equiv) was used.

5. General Procedure for Aldehydic C–H Fluorination Reaction

A Schlenk tube with a magnetic stir bar was charged with an aldehyde (2.0 mmol, 1.0 equiv), **1e** (62.4 mg, 0.20 mmol, 10 mol%), Selectfluor (1.42 g, 4.0 mmol, 2.0 equiv) and MeCN (20 mL) under an argon atmosphere. After being stirred at room temperature for 4 h, the reaction mixture was quenched with H_2O and extracted with ethyl acetate three times. The combined organic layer was dried over Na_2SO_4 and concentrated. The residue was purified by flash column chromatography on silica gel to afford the following compounds.

3-Phenylpropanoyl Fluoride (6a)

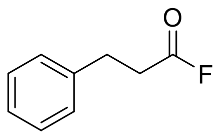

Colorless liquid; 161 mg, 53%.
^{1}H NMR (500 MHz, $CDCl_3$) δ 7.32 (t, J = 7.4 Hz, 2H), 7.25–7.21 (m, 3H), 3.00 (t, J = 7.7 Hz, 2H), 2.84 (t, J = 7.5 Hz, 2H); **^{13}C NMR** (125 MHz, $CDCl_3$) δ 162.9 (d, $J_{C–F}$ = 361.3 Hz), 139.0, 128.9, 128.4, 127.0, 34.0 (d, $J_{C–F}$ = 51.3 Hz), 30.1; **^{19}F NMR** (470 MHz, $CDCl_3$) δ 45.4; **HRMS (ESI)** calculated for C_9H_9OFNa: m/z 175.0530 ($[M + Na]^+$), found: m/z 175.0529 ($[M + Na]^+$); **IR (neat)** 2930, 1838, 1088, 1074, 748, 697 cm^{-1}.

Benzyl (3-Fluoro-3-oxopropyl)carbamate (6d)

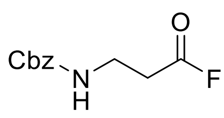

White solid; 288 mg, 64%.
^{1}H NMR (500 MHz, $CDCl_3$) δ 7.38–7.31 (m, 5H), 5.28 (br s, 1H), 5.10 (s, 2H), 3.49 (app q, 2H), 2.77 (t, J = 5.5 Hz, 2H); **^{13}C NMR** (125 MHz, $CDCl_3$) δ 162.6 (d, $J_{C–F}$ = 361.3 Hz), 156.4, 136.3, 128.7, 128.4, 128.3, 67.1, 36.0, 33.0 (d, $J_{C–F}$ = 48.8 Hz); **^{19}F NMR** (470 MHz, $CHCl_3$) δ 46.8; **HRMS (ESI)** calculated for $C_{11}H_{12}O_3NFNa$: m/z 248.0693 ($[M + Na]^+$), found: m/z 248.0696 ($[M + Na]^+$); **IR (neat)** 3335, 1838, 1698, 1252, 1094, 697 cm^{-1}.

6-Fluoro-6-oxohexyl Benzoate (6e)

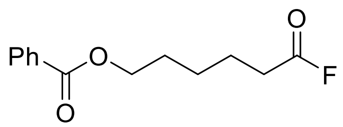

The reaction was performed on 1.0 mmol scale.
Colorless liquid; 123 mg, 52%.

^{1}H NMR (500 MHz, $CDCl_3$) δ 8.03 (dd, J = 8.5, 1.4 Hz, 2H), 7.56 (t, J = 7.4 Hz, 1H), 7.44 (t, J = 7.7 Hz, 2H), 4.34 (t, J = 6.5 Hz, 2H), 2.55 (t, J = 7.2 Hz, 2H), 1.84-1.74 (m, 4H), 1.59–1.54 (m, 2H); **^{13}C NMR** (125 MHz, $CDCl_3$) δ 166.7, 163.4 (d, $J_{C\text{–}F}$ = 360.0 Hz), 133.0, 130.4, 129.6, 128.5, 64.6, 32.1 (d, $J_{C\text{–}F}$ = 51.3 Hz), 28.4, 25.4, 23.8; **^{19}F NMR** (470 MHz, $CDCl_3$) δ 45.6; **HRMS (ESI)** calculated for $C_{13}H_{15}O_3FNa$: *m/z* 261.0897 ([M + Na]$^+$), found: *m/z* 261.0902 ([M + Na]$^+$); **IR (neat)** 2950, 1837, 1714, 1315, 1271, 709 cm^{-1}.

1-Tosylpiperidine-4-carbonyl Fluoride (6h)

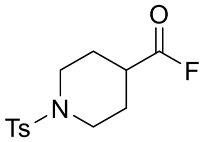

White solid; 348 mg, 61%.

^{1}H NMR (500 MHz, $CDCl_3$) δ 7.64 (d, J = 8.2 Hz, 2H), 7.33 (d, J = 7.9 Hz, 2H), 3.55 (m, 2H), 2.63 (app t, 2H), 2.54-2.49 (m, 1H), 2.44 (s, 3H), 2.07–2.04 (m, 2H), 1.91 (td, J = 13.7, 10.0 Hz, 2H); **^{13}C NMR** (125 MHz, $CDCl_3$) δ 163.8 (d, $J_{C\text{–}F}$ = 367.5 Hz), 144.0, 133.1, 129.9, 127.8, 45.0, 38.6 (d, $J_{C\text{–}F}$ = 50 Hz), 26.7, 21.6; **^{19}F NMR** (470 MHz, $CDCl_3$) δ 37.7; **HRMS (ESI)** calculated for $C_{13}H_{17}O_3NFS$: *m/z* 286.0908 ([M + H]$^+$), found: *m/z* 286.0913 ([M + H]$^+$); **IR (neat)** 2934, 1835, 1162, 931, 725 cm^{-1}.

4-Methoxybenzoyl Fluoride (6m)

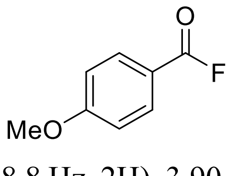

The reaction was conducted at 80 °C.

White solid; 244 mg, 79%.

^{1}H NMR (500 MHz, $CDCl_3$) δ 8.00 (d, J = 8.8 Hz, 2H), 6.98 (d, J = 8.8 Hz, 2H), 3.90 (s, 3H); **^{13}C NMR** (125 MHz, $CDCl_3$) δ 165.4, 157.5 (d, $J_{C\text{–}F}$ = 345 Hz), 133.9 (d, $J_{C\text{–}F}$ = 3.8 Hz), 117.1 (d, $J_{C\text{–}F}$ = 61.3 Hz), 114.6, 55.8; **^{19}F NMR** (470 MHz, $CHCl_3$) δ 16.1; **HRMS (ESI)** calculated for $C_8H_7O_2FNa$: *m/z* 177.0322 ([M + Na]$^+$), found: *m/z* 177.0322 ([M + Na]$^+$); **IR (neat)** 2940, 1792, 1167, 1016, 758 cm^{-1}.

4-Methylbenzoyl Fluoride (6n)

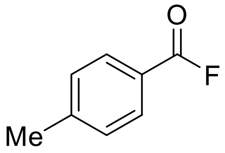

The reaction was performed on 5.0 mmol scale at 80 °C.

Colorless liquid; 295 mg, 43%.

^{1}H NMR (500 MHz, $CDCl_3$) δ 7.93 (d, J = 7.9 Hz, 2H), 7.32 (d, J = 7.9 Hz, 2H), 2.45 (s, 3H); **^{13}C NMR** (125 MHz, $CDCl_3$) δ 157.7 (d, $J_{C–F}$ = 343.3 Hz), 146.7, 131.6 (d, $J_{C–F}$ = 3.6 Hz), 129.9, 122.2 (d, $J_{C–F}$ = 60.8 Hz), 22.0; **^{19}F NMR** (470 MHz, $CHCl_3$) δ 17.5; **HRMS (ESI)** calculated for C_8H_7OFNa: *m/z* 161.0373 ([M + Na]$^+$), found: *m/z* 161.0374 ([M + Na]$^+$); **IR (neat)** 2925, 1800, 1255, 1032, 738 cm^{-1}.

Cinnamoyl Fluoride (6o)

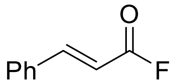

The reaction was conducted at 80 °C.

Colorless liquid; 209 mg, 69%.

^{1}H NMR (500 MHz, $CDCl_3$) δ 7.84 (d, J = 15.9 Hz, 1H), 7.58–7.56 (m, 2H), 7.46 (m, 3H), 6.38 (dd, J = 15.9, 7.4 Hz, 1H); **^{13}C NMR** (125 MHz, $CDCl_3$) δ 157.1 (d, $J_{C–F}$ = 338.6 Hz), 151.5 (d, $J_{C–F}$ = 6.0 Hz), 133.27, 131.9, 129.2, 128.8, 112.1 (d, $J_{C–F}$ = 66.8 Hz); **^{19}F NMR** (470 MHz, $CHCl_3$) δ 25.7; **HRMS (ESI)** calculated for C_9H_7OFNa: *m/z* 173.0373 ([M + Na]$^+$), found: *m/z* 173.0371 ([M + Na]$^+$); **IR (neat)** 1790, 1628, 1187, 1103, 762 cm^{-1}.

6. General Procedure for One-pot Transformation

A Schlenk tube with a magnetic stir bar was charged with an aldehyde (0.2 mmol, 1.0 equiv), **1e** (3.1 mg, 0.01 mmol, 5 mol%), Selectfluor (71 mg, 0.2 mmol, 1.0 equiv) and MeCN (2.0 mL) under an argon atmosphere. After being stirred at room temperature for 4 h, benzylamine (44 µL, 0.4 mmol, 2.0 equiv) was added and the mixture was stirred at room temperature for 2 h. The reaction mixture was quenched with H_2O and extracted with ethyl acetate three times. The combined organic layer was dried over Na_2SO_4 and concentrated. The residue was purified by flash column chromatography on silica gel to afford the following compound.

***N*-Benzyl-3-phenylpropanamide (7a)**[10]

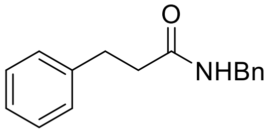

White solid; 48 mg, quant.
^{1}H NMR (500 MHz, $CDCl_3$) δ 7.30–7.23 (m, 5H), 7.22–7.16 (m, 3H), 7.14–7.13 (m, 2H), 5.80 (br s, 1H), 4.38 (d, J = 5.7 Hz, 2H), 2.98 (t, J = 7.7 Hz, 2H), 2.50 (t, J = 7.7 Hz, 2H); **^{13}C NMR** (125 MHz, $CDCl_3$) δ 172.1, 140.9, 138.3, 128.7, 128.6, 128.5, 127.8, 127.5, 126.3, 43.6, 38.5, 31.8.

***N*-Benzylhexanamide (7b)**[11]

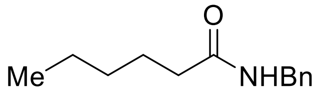

White solid; 29 mg, 70%.
^{1}H NMR (500 MHz, $CDCl_3$) δ 7.33–7.30 (m, 2H), 7.26 (m, 3H), 5.86 (br s, 1H), 4.41 (d, J = 5.7 Hz, 2H), 2.19 (t, J = 7.7 Hz, 2H), 1.67–1.61 (m, 2H), 1.30 (m, 4H), 0.88 (t, J = 6.8 Hz, 3H); **^{13}C NMR** (125 MHz, $CDCl_3$) δ 173.2, 138.5, 128.8, 127.9, 127.6, 43.7, 36.9, 31.6, 25.6, 22.5, 14.0.

***N*-Benzyl-3-methylbutanamide (7c)**[12]

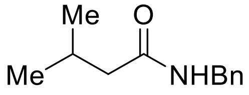

White solid; 37 mg, 98%.
^{1}H NMR (500 MHz, $CDCl_3$) δ 7.32–7.26 (m, 2H), 7.26–7.24 (m, 3H), 5.81 (br s, 1H), 4.41 (d, J = 5.7 Hz, 2H), 2.15–2.08 (m, 1H), 2.05 (d, J = 7.1 Hz, 2H), 0.94 (d, J = 6.5 Hz, 6H); **^{13}C NMR** (125 MHz, $CDCl_3$) δ 172.5, 138.6, 128.8, 127.9, 127.6, 46.2, 43.6, 26.3, 22.6.

Benzyl (3-(Benzylamino)-3-oxopropyl)carbamate (7d)

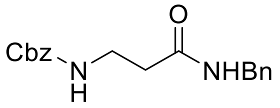

White solid; 45 mg, 72%.

^{1}H NMR (500 MHz, $CDCl_3$) δ 7.35–7.31 (m, 6H), 7.27 (m, 4H), 5.90 (br s, 1H), 5.45 (br s, 1H), 5.08 (s, 2H), 4.42 (d, J = 5.7 Hz, 2H), 3.50 (app q, 2H), 2.45 (t, J = 5.7 Hz, 2H); **^{13}C NMR** (125 MHz, $CDCl_3$) δ 171.2, 156.7, 138.1, 136.7, 128.9, 128.7, 128.2, 128.1, 127.9, 127.8, 66.8, 43.8, 37.3, 36.2; **HRMS (ESI)** calculated for $C_{18}H_{20}O_3N_2Na$: *m/z* 335.1366 ($[M + Na]^+$), found: *m/z* 335.1370 ($[M + Na]^+$); **IR (neat)** 3301, 1690, 1642, 1544, 1266, 733, 695 cm^{-1}.

6-(Benzylamino)-6-oxohexyl Benzoate (7e)

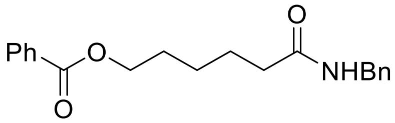

White solid; 65 mg, quant.

^{1}H NMR (500 MHz, $CDCl_3$) δ 8.02 (d, J = 7.1 Hz, 2H), 7.55 (t, J = 7.4 Hz, 1H), 7.42 (t, J = 7.7 Hz, 2H), 7.31 (t, J = 7.2 Hz, 2H), 7.25 (d, J = 7.7 Hz, 3H), 6.02 (br s, 1H), 4.41 (d, J = 5.7 Hz, 2H), 4.30 (t, J = 6.7 Hz, 2H), 2.23 (t, J = 7.7 Hz, 2H), 1.80–1.70 (m, 4H), 1.51–1.44 (m, 2H); **^{13}C NMR** (125 MHz, $CDCl_3$) δ 172.7, 166.7, 138.5, 133.0, 130.4, 129.6, 128.7, 128.4, 127.8, 127.5, 64.9, 43.6, 36.6, 28.6, 25.8, 25.4; **HRMS (ESI)** calculated for $C_{20}H_{24}O_3N$: *m/z* 326.1751 ($[M + H]^+$), found: *m/z* 326.1751 ($[M + H]^+$); **IR (neat)** 3289, 1715, 1644, 1314, 710 cm^{-1}.

***N*-Benzyl-3-(benzyloxy)propanamide (7f)**

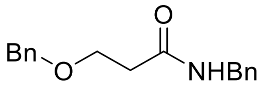

Orange solid; 24 mg, 45%.

^{1}H NMR (500 MHz, $CDCl_3$) δ 7.37 (app d, 1H), 7.32–7.29 (m, 5H), 7.26 (app t, 2H), 7.23–7.21 (m, 2H), 6.53 (br s, 1H), 4.51 (s, 2H), 4.44 (d, J = 5.7 Hz, 2H), 3.77 (t, J = 5.7 Hz, 2H), 2.55 (t, J = 5.8 Hz, 2H); **^{13}C NMR** (125 MHz, $CDCl_3$) δ 171.5, 138.4, 137.7, 128.8, 128.6, 128.0, 127.9, 127.8, 127.5, 73.5, 66.5, 43.6, 37.3; **HRMS (ESI)** calculated for $C_{17}H_{19}O_2NNa$: *m/z* 292.1308 ($[M + Na]^+$), found: *m/z* 292.1309 ($[M + Na]^+$); **IR (neat)** 3294, 2925, 1647, 1545, 1094, 736, 698 cm^{-1}.

***N*-Benzylcyclohexanecarboxamide (7g)**[10]

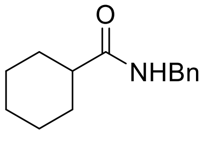

White solid; 35 mg, 81%.

^{1}H NMR (500 MHz, $CDCl_3$) δ 7.32 (t, J = 7.5 Hz, 2H), 7.27–7.24 (m, 3H), 5.84 (br s, 1H), 4.42 (d, J = 5.7 Hz, 2H), 2.11 (tt, J = 11.8, 3.4 Hz,

1H), 1.88 (m, 2H), 1.78 (m, 2H), 1.67 (m, 1H), 1.49–1.42 (m, 2H), 1.24 (m, 3H); **^{13}C NMR** (125 MHz, $CDCl_3$) δ 176.1, 138.7, 128.8, 127.8, 127.5, 45.6, 43.5, 29.8, 25.8 (Two peaks were overlapped).

***N*-Benzyl-1-tosylpiperidine-4-carboxamide (7h)**

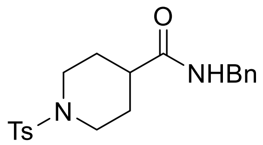

White solid; 52 mg, 70%.

^{1}H NMR (500 MHz, $CDCl_3$) δ 7.63 (d, J = 8.2 Hz, 2H), 7.31 (app d, 4H), 7.28 (d, J = 7.1 Hz, 1H), 7.22 (d, J = 7.4 Hz, 2H), 5.72 (br s, 1H), 4.40 (d, J = 5.7 Hz, 2H), 3.75 (d, J = 11.9 Hz, 2H), 2.43 (s, 3H), 2.38 (td, J = 11.5, 2.6 Hz, 2H), 2.10–2.04 (m, 1H), 1.91 (m, 2H), 1.82 (m, 2H); **^{13}C NMR** (125 MHz, $CDCl_3$) δ 173.5, 143.7, 138.2, 133.4, 129.8, 128.9, 127.9, 127.8 (Two peaks were overlapped), 45.6, 43.7, 42.3, 28.3, 21.7; **HRMS (ESI)** calculated for $C_{20}H_{25}O_3N_2S$: *m/z* 373.1580 ([M + H]$^+$), found: *m/z* 373.1585 ([M + H]$^+$); **IR (neat)** 3295, 1646, 1160, 931 cm^{-1}.

***N*-Benzyltetrahydro-2*H*-pyran-4-carboxamide (7i)[13]**

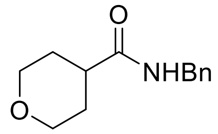

The reaction was conducted at 50 °C.

White solid; 32 mg, 74%.

^{1}H NMR (500 MHz, $CDCl_3$) δ 7.33 (t, J = 7.2 Hz, 2H), 7.29–7.25 (m, 3H), 5.79 (br s, 1H), 4.44 (d, J = 5.4 Hz, 2H), 4.01 (d, J = 11.3 Hz, 2H), 3.40 (t, J = 11.5 Hz, 2H), 2.40–2.33 (m, 1H), 1.87–1.76 (m, 4H); **^{13}C NMR** (125 MHz, $CDCl_3$) δ 174.3, 138.3, 128.9, 127.9, 127.7, 67.4, 43.7, 42.4, 29.4.

Benzyl ((1*S*, 2*R*)-3-(Benzylamino)-2-methyl-3-oxo-1-phenylpropyl)carbamate (7k)

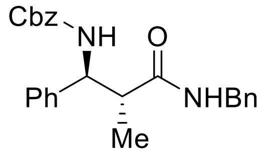

White solid; 34 mg, 42%; 99% ee, *anti*/*syn* = >20/1.

^{1}H NMR (500 MHz, $CDCl_3$) δ 7.36 (m, 4H), 7.28 (m, 3H), 7.24 (m, 2H), 7.21 (t, J = 3.1 Hz, 3H), 6.95 (d, J = 8.5 Hz, 1H), 6.88 (s, 2H), 5.49 (br s, 1H), 5.10 (m, 3H), 4.85 (q, J = 4.0 Hz, 1H), 4.31 (dd, J = 14.9, 6.4 Hz, 1H), 4.13 (dd, J = 14.9, 5.0 Hz, 1H), 2.62–2.60 (m, 1H), 1.36 (d, J = 6.8 Hz, 3H); **^{13}C NMR** (125 MHz, $CDCl_3$) δ 174.4, 156.5, 141.6, 137.6, 136.8, 128.7, 128.5, 128.4, 128.2, 128.0, 127.6, 127.5, 127.4, 126.2, 66.8, 58.1, 46.6, 43.3, 16.6; **HRMS (ESI)** calculated for $C_{25}H_{27}O_3N_2$: *m/z* 403.2016 ([M + H]$^+$), found: *m/z* 403.2019 ([M + H]$^+$); **IR (neat)** 3320,

1687, 1647, 1058, 697 cm^{-1}; **HPLC analysis**: Daicel Chiralpak IC-3, hexane/*i*-PrOH = 2/1, flow rate = 1.0 mL/min, retention time; 16.1 min (major) and 35.4 min; $[\alpha]_D^{28}$ = –16.6 (*c* 1.5, $CHCl_3$, 99% ee).

***N*-Benzylbenzamide (7l)**[14]

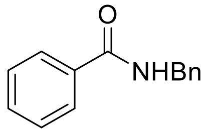

The reaction was conducted at 80 °C.
White solid; 34 mg, 80%.
^{1}H NMR (500 MHz, $CDCl_3$) δ 7.79 (d, J = 7.1 Hz, 2H), 7.50 (t, J = 7.4 Hz, 1H), 7.42 (t, J = 7.5 Hz, 2H), 7.35 (app d, 4H), 7.32–7.28 (m, 1H), 6.50 (br s, 1H), 4.64 (d, J = 5.7 Hz, 2H); **^{13}C NMR** (125 MHz, $CDCl_3$) δ 167.5, 138.3, 134.5, 131.7, 128.9, 128.7, 128.1, 127.8, 127.1, 44.3.

***N*-Benzyl-4-methoxybenzamide (7m)**[14]

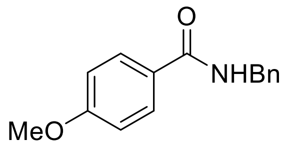

The reaction was conducted at 80 °C.
White solid; 44 mg, 91%.
^{1}H NMR (500 MHz, $CDCl_3$) δ 7.72 (d, J = 7.9 Hz, 2H), 7.26 (m, 4H), 7.22 (t, J = 4.3 Hz, 1H), 6.82 (d, J = 8.8 Hz, 2H), 4.53 (d, J = 5.7 Hz, 2H), 3.76 (s, 3H); **^{13}C NMR** (125 MHz, $CDCl_3$) δ 167.1, 162.2, 138.6, 128.9, 128.7, 127.8, 127.4, 126.7, 113.7, 55.4, 44.0.

***N*-Benzyl-4-methylbenzamide (7n)**[14]

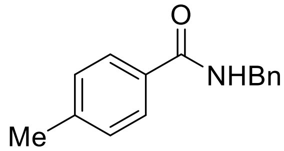

The reaction was conducted at 80 °C.
White solid; 33 mg, 74%.
^{1}H NMR (500 MHz, $CDCl_3$) δ 7.69 (d, J = 8.2 Hz, 2H), 7.35 (app d, J = 4.5 Hz, 4H), 7.32–7.28 (m, 1H), 7.22 (d, J = 7.9 Hz, 2H), 6.42 (br s, 1H), 4.64 (d, J = 5.7 Hz, 2H), 2.39 (s, 3H); **^{13}C NMR** (125 MHz, $CDCl_3$) δ 167.5, 142.1, 138.4, 131.8, 129.4, 128.9, 128.1, 127.7, 127.1, 44.2, 21.6.

***N*-Benzylcinnamamide (7o)**[14]

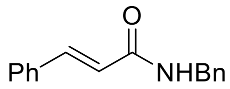

The reaction was conducted at 80 °C.
White solid; 26 mg, 54%.

^{1}H NMR (500 MHz, $CDCl_3$) *δ* 7.68 (d, *J* = 15.6 Hz, 1H), 7.50 (dd, *J* = 7.5, 2.1 Hz, 2H), 7.39–7.33 (m, 7H), 7.29 (t, *J* = 8.6 Hz, 1H), 6.41 (d, *J* = 15.6 Hz, 1H), 5.89 (br s, 1H), 4.59 (d, *J* = 5.7 Hz, 2H); **^{13}C NMR** (125 MHz, $CDCl_3$) *δ* 165.9, 141.5, 138.3, 134.9, 129.8, 128.93, 128.87, 128.0, 127.9, 127.7, 120.6, 44.0.

7. Derivatizations of Acyl Fluoride

Procedure A

A Schlenk tube with a magnetic stir bar was charged with an aldehyde (0.2 mmol, 1.0 equiv), **1e** (3.1 mg, 0.01 mmol, 5 mol%), Selectfluor (71 mg, 0.2 mmol, 1.0 equiv) and MeCN (2.0 mL) under an argon atmosphere. After being stirred at room temperature for 4 h, an amine (0.4 mmol, 2.0 equiv) was added, and the mixture was stirred at room temperature for 2 h. The reaction mixture was quenched with H_2O and extracted with ethyl acetate three times. The combined organic layer was dried over Na_2SO_4 and concentrated. The residue was purified by flash column chromatography on silica gel to afford the following compounds.

Procedure B

A Schlenk tube with a magnetic stir bar was charged with an aldehyde (0.2 mmol, 1.0 equiv), **1e** (3.1 mg, 0.01 mmol, 5 mol%), Selectfluor (71 mg, 0.2 mmol, 1.0 equiv) and MeCN (2.0 mL) under an argon atmosphere. After being stirred at room temperature for 4 h, an amine hydrochloride (0.2 mmol, 1.0 equiv) and triethylamine (84 μL, 0.6 mmol, 3.0 equiv) were added, and the mixture was stirred at room temperature for 2 h. The reaction mixture was quenched with H_2O and extracted with ethyl acetate three times. The combined organic layer was dried over Na_2SO_4 and concentrated. The residue was purified by flash column chromatography on silica gel to afford the following compounds.

Procedure C

A Schlenk tube with a magnetic stir bar was charged with an aldehyde (0.2 mmol, 1.0 equiv), **1e** (3.1 mg, 0.01 mmol, 5 mol%), Selectfluor (71 mg, 0.2 mmol, 1.0 equiv) and MeCN (2.0 mL) under an argon atmosphere. After being stirred at room temperature for 4 h, oxazolidin-2-one (17 mg, 0.2 mmol, 1.0 equiv), 4-dimethylaminopyridine (24 mg, 0.2 mmol, 1.0 equiv) and triethylamine (56 μL, 0.4 mmol, 2.0 equiv) were added, and the mixture was stirred at room temperature for 2 h. The reaction mixture was quenched with H_2O and extracted with ethyl acetate three times. The combined organic layer was dried over Na_2SO_4 and concentrated. The residue was purified by flash column chromatography on silica gel to afford the following compounds.

Procedure D

A Schlenk tube with a magnetic stir bar was charged with an aldehyde (0.2 mmol, 1.0 equiv), **1e** (3.1 mg, 0.01 mmol, 5 mol%), Selectfluor (71 mg, 0.2 mmol, 1.0 equiv) and MeCN (2.0 mL) under an argon atmosphere. After being stirred at room temperature for 4 h, an alcohol or dodecanethiol (2.0 mmol, 10 equiv), 4-dimethylaminopyridine (24 mg, 0.2 mmol, 1.0 equiv) and triethylamine (56 μL, 0.4 mmol, 2.0 equiv) were added, and the mixture was stirred at room temperature for 2 h. The reaction mixture was quenched with H_2O and extracted with ethyl acetate three times. The combined organic layer was dried over Na_2SO_4 and concentrated. The residue was purified by flash column chromatography on silica gel to afford the following compounds.

Procedure E

A Schlenk tube with a magnetic stir bar was charged with an aldehyde (0.2 mmol, 1.0 equiv), **1e** (6.2 mg, 0.02 mmol, 10 mol%), Selectfluor (142 mg, 0.4 mmol, 2.0 equiv) and MeCN (2.0 mL) under an argon atmosphere. After being stirred at 50 °C for 4 h, triethylamine (112 μL, 0.8 mmol, 4.0 equiv) was added, and the mixture was stirred at room temperature for 2 h. The reaction mixture was quenched with H_2O and extracted with ethyl acetate three times. The combined organic layer was dried over Na_2SO_4 and concentrated. The residue was purified by flash column chromatography on silica gel to afford the following compounds.

3-Phenyl-1-(pyrrolidin-1-yl)propan-1-one (8a)[15]

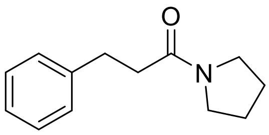

This compound was synthesized by procedure A.

Yellow liquid; 40 mg, 99%.

^{1}H NMR (500 MHz, $CDCl_3$) δ 7.31 (t, J = 7.7 Hz, 2H), 7.26–7.21 (m, 3H), 3.49 (t, J = 6.8 Hz, 2H), 3.31 (t, J = 6.8 Hz, 2H), 3.01 (t, J = 7.9 Hz, 2H), 2.59 (t, J = 7.9 Hz, 2H), 1.93–1.81 (m, 4H); **^{13}C NMR** (125 MHz, $CDCl_3$) δ 170.8, 141.6, 128.52, 128.50, 126.1, 46.6, 45.7, 36.8, 31.3, 26.1, 24.5.

***N*-Methoxy-*N*-methyl-3-phenylpropanamide (8b)**[16]

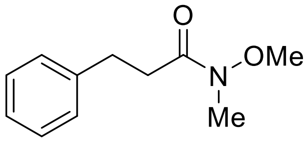

This compound was synthesized by procedure B.

Colorless liquid; 37 mg, 96%.

¹H NMR (500 MHz, $CDCl_3$) δ 7.29 (t, J = 7.4 Hz, 2H), 7.24–7.18 (m, 3H), 3.60 (s, 3H), 3.18 (s, 3H), 2.98–2.94 (m, 2H), 2.75 (t, J = 7.5 Hz, 2H); **¹³C NMR** (125 MHz, $CDCl_3$) δ 173.8, 141.5, 128.6 (Two peaks were overlapped), 126.2, 61.3, 33.9, 32.3, 30.8.

Methyl (3-Phenylpropanoyl)glycinate (8c)

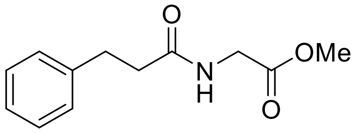

This compound was synthesized by procedure B.

Yellow solid; 38 mg, 85%.

¹H NMR (500 MHz, $CDCl_3$) δ 7.30–7.26 (m, 2H), 7.20 (m, 3H), 5.96 (br s, 1H), 4.02 (d, J = 5.1 Hz, 2H), 3.74 (s, 3H), 2.98 (t, J = 7.9 Hz, 2H), 2.55 (t, J = 7.9 Hz, 2H); **¹³C NMR** (125 MHz, $CDCl_3$) δ 172.4, 170.5, 140.8, 128.6, 128.4, 126.4, 52.5, 41.3, 38.1, 31.5; **HRMS (ESI)** calculated for $C_{12}H_{15}O_3NNa$: *m/z* 244.0944 ([M + Na]$^+$), found: *m/z* 244.0943 ([M + Na]$^+$); **IR (neat)** 3306, 1750, 1655, 1208, 700 cm^{-1}.

Methyl (3-Phenylpropanoyl)-*L*-alaninate (8d)[17]

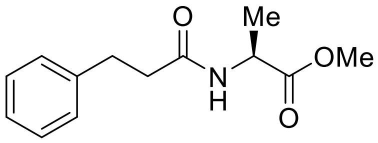

This compound was synthesized by procedure B.

White solid; 35 mg, 75%; 99% ee.

¹H NMR (500 MHz, $CDCl_3$) δ 7.28 (t, J = 7.5 Hz, 2H), 7.21–7.18 (m, 3H), 5.93 (br s, 1H), 4.61–4.56 (m, 1H), 3.73 (s, 3H), 2.97 (t, J = 7.7 Hz, 2H), 2.57–2.46 (m, 2H), 1.34 (d, J = 7.1 Hz, 3H); **¹³C NMR** (125 MHz, $CDCl_3$) δ 173.7, 171.6, 140.8, 128.7, 128.5, 126.4, 52.6, 48.1, 38.4, 31.7, 18.7; **HPLC analysis**: Daicel Chiralpak IC-3, hexane/*i*-PrOH = 10/1, flow rate = 1.0 mL/min, retention time; 26.3 min (major) and 34.6 min; $[\alpha]_D^{28}$ = –2.3 (*c* 0.8, $CHCl_3$, 99% ee).

3-(3-Phenylpropanoyl)oxazolidin-2-one (8e)[18]

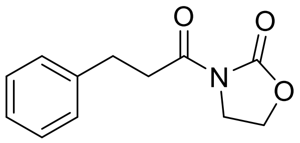

This compound was synthesized by procedure C.

White solid; 33 mg, 75%.

^{1}H NMR (500 MHz, $CDCl_3$) δ 7.31 (t, J = 7.7 Hz, 2H), 7.28–7.26 (m, 2H), 7.22 (t, J = 7.2 Hz, 1H), 4.42 (t, J = 8.1 Hz, 2H), 4.03 (t, J = 8.1 Hz, 2H), 3.28 (t, J = 7.7 Hz, 2H), 3.01 (t, J = 7.7 Hz, 2H); **^{13}C NMR** (125 MHz, $CDCl_3$) δ 172.6, 153.6, 140.6, 128.65, 128.57, 126.3, 62.2, 42.6, 36.9, 30.3.

Ethyl 3-Phenylpropanoate (8f)

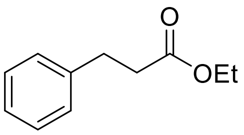

This compound was synthesized by procedure D.
Colorless liquid; 33 mg, 93%.

^{1}H NMR (500 MHz, $CDCl_3$) δ 7.31–7.28 (m, 2H), 7.22–7.19 (m, 3H), 4.14 (q, J = 7.2 Hz, 2H), 2.96 (t, J = 7.8 Hz, 2H), 2.63 (t, J = 7.7 Hz, 2H), 1.24 (t, J = 7.1 Hz, 3H); **^{13}C NMR** (125 MHz, $CDCl_3$) δ 173.0, 140.7, 128.6, 128.4, 126.3, 60.5, 36.1, 31.1, 14.3; **HRMS (ESI)** calculated for $C_{11}H_{14}O_2Na$: *m/z* 201.0886 ($[M + Na]^+$), found: *m/z* 201.0887 ($[M + Na]^+$); **IR (neat)** 2979, 1732, 1160, 749, 698 cm^{-1}.

Benzyl 3-Phenylpropanoate (8g)[19]

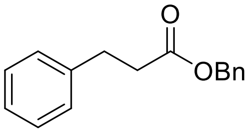

This compound was synthesized by procedure D.
Colorless liquid; 43 mg, 90%.

^{1}H NMR (500 MHz, $CDCl_3$) δ 7.39–7.28 (m, 7H), 7.23–7.20 (m, 3H), 5.13 (s, 2H), 2.99 (t, J = 7.8 Hz, 2H), 2.70 (t, J = 7.8 Hz, 2H); **^{13}C NMR** (125 MHz, $CDCl_3$) δ 172.8, 140.5, 136.0, 128.7, 128.6, 128.4, 128.3, 128.0, 126.4, 66.4, 36.0, 31.1.

(1*R*,2*S*,5*R*)-2-Isopropyl-5-methylcyclohexyl 3-phenylpropanoate (8h)

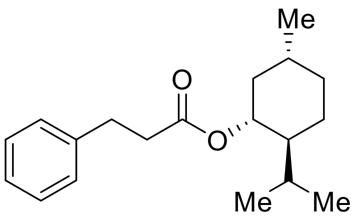

This compound was synthesized by procedure D.
Colorless liquid; 45 mg, 78%.

^{1}H NMR (500 MHz, $CDCl_3$) δ 7.30–7.26 (m, 2H), 7.22–7.18 (m, 3H), 4.68 (td, J = 10.9, 4.3 Hz, 1H), 2.96 (t, J = 8.1 Hz, 2H), 2.62 (t, J = 7.8 Hz, 2H), 1.97–1.92 (m, 1H), 1.77–1.70 (m, 1H), 1.70–1.63 (m, 2H), 1.51–1.44 (m, 1H), 1.37–1.31 (m, 1H), 1.09–1.00 (m, 1H), 0.97–0.92 (m, 2H), 0.90 (d, J = 6.5 Hz, 3H), 0.86 (d, J = 6.8 Hz, 3H), 0.71 (d, J = 7.1 Hz, 3H); **^{13}C NMR** (125 MHz, $CDCl_3$) δ 172.6, 140.7, 128.5, 128.4, 126.3, 74.3, 47.1, 41.0, 36.3, 34.4, 31.5, 31.2, 26.3, 23.5, 22.1, 20.9, 16.4; **HRMS (ESI)** calculated for $C_{19}H_{28}O_2Na$: *m/z* 311.1982 ([M

+ Na]$^+$), found: *m/z* 311.1990 ([M + Na]$^+$); **IR (neat)** 2954, 2927, 1730, 1455, 1175, 698 cm^{-1}.

***S*-Dodecyl 3-Phenylpropanethioate (8i)**[20]

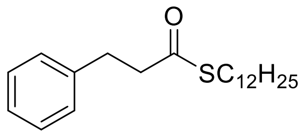

This compound was synthesized by procedure D.

Colorless liquid; 66 mg, 98%;

^{1}H NMR (500 MHz, $CDCl_3$) δ 7.28 (m, 2H), 7.20 (app t, 3H), 2.99 (t, *J* = 7.8 Hz, 2H), 2.89–2.84 (m, 4H), 1.60–1.53 (m, 3H), 1.26 (m, 17H), 0.90 (t, *J* = 6.9 Hz, 3H); **^{13}C NMR** (125 MHz, $CDCl_3$) δ 198.8, 140.3, 128.6, 128.4, 126.4, 45.7, 32.1, 31.7, 31.6, 29.8, 29.72, 29.67, 29.62, 29.5, 29.3, 29.1, 28.9, 22.8, 14.2.

3-Isopropyl-4-(4-nitrophenyl)oxetan-2-one (10)

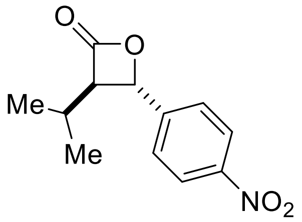

This compound was synthesized by procedure E.

Brown solid; 25 mg, 53%; *anti*/*syn* = >20/1.

^{1}**H NMR** (500 MHz, $CDCl_3$) δ 8.29 (d, *J* = 8.7 Hz, 2H), 7.56 (d, *J* = 8.7 Hz, 2H), 5.36 (d, *J* = 4.1 Hz, 1H), 3.33 (q, *J* = 4.3 Hz, 1H), 2.31 (dt, *J* = 21.7, 6.7 Hz, 1H), 1.18 (d, *J* = 6.8 Hz, 3H), 1.14 (d, *J* = 6.8 Hz, 3H); **^{13}C NMR** (125 MHz, $CDCl_3$) δ 169.3, 148.3, 144.8, 126.3, 124.4, 74.4, 67.6, 28.2, 20.5, 19.9; **HRMS (ESI)** calculated for $C_{12}H_{14}O_4N$: *m/z* 236.0917 ([M + H]$^+$), found: *m/z* 236.0937 ([M + H]$^+$); **IR (neat)** 2964, 1828, 1522, 1348, 1106 cm$^-$.

8. Procedures for Synthesis of Unsymmetrical Ketones from Acyl Fluoride

Procedure for Suzuki–Miyaura Coupling Reaction of Acyl Fluoride

To a Schlenk tube, $Pd(OAc)_2$ (2.2 mg, 0.01 mmol, 1 mol%), $P(4\text{-}MeOC_6H_4)_3$ (14.0 mg, 0.04 mmol, 4 mol%), KF (87.1 mg, 1.5 mmol, 1.5 equiv), phenylboronic acid (0.183 g, 1.5 mmol. 1.5 equiv) and toluene (2.0 mL) were added. To the mixture was added 3-phenylpropanoyl fluoride **6a** (141 μL, 1.0 mmol, 1.0 equiv), and the mixture was stirred overnight at 120 °C. The reaction mixture was quenched with H_2O and extracted with ethyl acetate three times. The combined organic layer was dried over Na_2SO_4 and concentrated. The residue was purified by flash column chromatography on silica gel to afford the following compound.

1,3-Diphenylpropan-1-one (11a)[21]

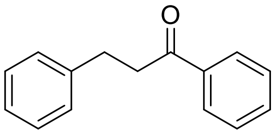

White solid, 160 mg, 76%.

^{1}H NMR (500 MHz, $CDCl_3$) *δ* 7.99 (d, *J* = 8.2 Hz, 2H), 7.57 (t, *J* = 7.4 Hz, 1H), 7.47 (t, *J* = 7.5 Hz, 2H), 7.34 (t, *J* = 7.5 Hz, 2H), 7.30 (d, *J* = 7.4 Hz, 2H), 7.25 (t, *J* = 7.2 Hz, 1H), 3.33–3.30 (m, 2H), 3.11 (t, *J* = 7.7 Hz, 2H); **^{13}C NMR** (125 MHz, $CDCl_3$) *δ* 199.1, 141.3, 136.8, 133.0, 128.6, 128.5, 128.4, 128.0, 126.1, 40.4, 30.1.

Procedure for Friedel-Crafts Acylation of Aromatic Compounds with Acyl Fluoride (Scheme 4b)

The solution of 3-phenylpropanoyl fluoride **6a** (28.3 μL, 0.2 mmol, 1.0 equiv) in MeCN (2.0 ml) was treated with TMSOTf (44 μL, 0.24 mmol, 1.2 equiv), followed by addition of 1,3-dimethoxybenzene (**a**) (29 μL, 0.2 mmol, 1.0 equiv). After being stirred overnight at room temperature, the solvent was evaporated and the residue was purified by flash column chromatography on silica gel to afford the following compound.

1-(2,4-Dimethoxyphenyl)-3-phenylpropan-1-one (11b)

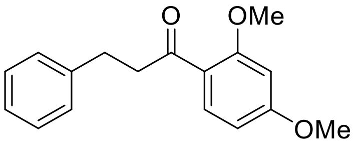

Orange liquid, 36 mg, 67%.

^{1}H NMR *δ* 7.83 (d, *J* = 8.8 Hz, 1H), 7.30 (t, *J* = 7.5 Hz, 2H), 7.27–7.25 (m, 2H), 7.20 (t, *J* = 7.1 Hz, 1H), 6.54 (dd, *J* =

8.8, 2.3 Hz, 1H), 6.46 (d, J = 2.3 Hz, 1H), 3.88 (s, 3H), 3.86 (s, 3H), 3.28 (t, J = 7.8 Hz, 2H), 3.02 (t, J = 7.8 Hz, 2H); **^{13}C NMR** (125 MHz, $CDCl_3$) δ 199.5, 164.5, 160.9, 142.1, 132.9, 128.6, 128.5, 125.9, 121.2, 105.3, 98.5, 55.64, 55.58, 45.4, 30.8; **HRMS (ESI)** calculated for $C_{17}H_{19}O_3$: m/z 271.1329 ([M + H]$^+$), found: m/z 271.1330 ([M + H]$^+$); **IR (neat)** 2940, 1598, 1285, 1028cm^{-1}.

Procedure for Reaction of Acyl Fluoride with Wittig Reagent (Scheme 4c)

A Schlenk tube with a magnetic stir bar was charged with 3-phenylpropanoyl fluoride **6a** (71 μL, 0.5 mmol, 1.0 equiv), methyl 2-(triphenyl-λ^5-phosphanylidene)acetate (167 mg, 0.5 mmol, 1.0 equiv), KF (0.17 g, 3.0 mmol, 6.0 equiv) and MeCN (0.5 mL) under an argon atmosphere. After being stirred overnight at 90 °C, the reaction mixture was concentrated. The residue was purified by flash column chromatography on silica gel to afford the following compound.

Methyl 3-Oxo-5-phenylpentanoate (11c)

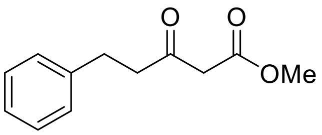

Yellow liquid, 62 mg, 60%.

^{1}H NMR (500 MHz, $CDCl_3$) δ 7.28 (t, J = 7.4 Hz, 2H), 7.19 (m, 3H), 3.72 (s, 3H), 3.44 (s, 2H), 2.94–2.91 (m, 2H), 2.89–2.86 (m, 2H); **^{13}C NMR** (125 MHz, $CDCl_3$) δ 201.8, 167.6, 140.6, 128.7, 128.4, 126.4, 52.5, 49.3, 44.6, 29.6; **HRMS (ESI)** calculated for $C_{12}H_{14}O_3Na$: m/z 229.0835 ([M + Na]$^+$), found: m/z 229.0836 ([M + Na]$^+$); **IR (neat)** 2925, 1743, 1716, 1260, 1260, 699 cm^{-1}.

Procedure for Allylation of Acyl Fluoride (Scheme 4d)

To a solution of allyltrimethylsilane (95 μL, 0.6 mmol, 1.2 equiv) in anhydrous CH_2Cl_2 (1.0 mL) was added 3-phenylpropanoyl fluoride **6a** (71 μL, 0.5 mmol, 1.0 equiv) slowly at –78 °C. After being stirred at –78 °C for several minutes, $TiCl_4$ (1.0 M in CH_2Cl_2, 500 μL, 0.5 mmol, 1.0 equiv) was added to the reaction mixture. After being stirred for 6 h, the reaction mixture was quenched with saturated aqueous NH_4Cl solution before being warmed to room temperature and then extracted with CH_2Cl_2 three times. The combined

organic layer was dried over Na_2SO_4 and concentrated. The residue was purified by flash column chromatography on silica gel to afford the following compound.

1-Phenylhex-5-en-3-one (11d)[22]

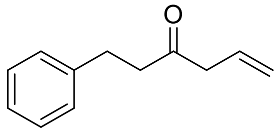

Colorless liquid, 71 mg, 81%.

^{1}H NMR (500 MHz, $CDCl_3$) δ 7.29 (t, J = 7.4 Hz, 2H), 7.22–7.19 (m, 3H), 5.96–5.88 (m, 1H), 5.20–5.12 (m, 2H), 3.16 (d, J = 7.1 Hz, 2H), 2.91 (t, J = 7.7 Hz, 2H), 2.78 (t, J = 7.5 Hz, 2H); **^{13}C NMR** (125 MHz, $CDCl_3$) δ 207.8, 141.0, 130.5, 128.6, 128.4, 126.2, 119.0, 48.0, 43.9, 29.7.

Procedure for Acylation of Silyl Ketene Acetal (Scheme 4e)

To a Schlenk tube, 3-phenylpropanoyl fluoride **6a** (28.3 μL, 0.2 mmol, 1.0 equiv), trimethyl((3-phenyl-4,5-dihydrofuran-2-yl)oxy)silane (**b**) (91 μL, 0.4 mmol, 2.0 equiv) and toluene (800 μL) were added. To the mixture was added *n*-Bu_3SnF (124 mg, 0.4 mmol, 2.0 equiv), and the mixture was stirred overnight at 120 °C. The reaction mixture was quenched with H_2O and extracted with ethyl acetate three times. The combined organic layer was dried over Na_2SO_4 and concentrated. The residue was purified by flash column chromatography on silica gel to afford the following compound.

3-Phenyl-3-(3-phenylpropanoyl)dihydrofuran-2(3*H*)-one (11e)

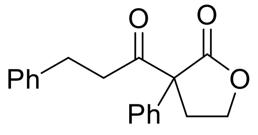

Yellow liquid, 57 mg, 96%.

^{1}H NMR (500 MHz, $CDCl_3$) δ 7.33 (m, 3H), 7.28 (d, J = 7.7 Hz, 2H), 7.20 (t, J = 7.4 Hz, 2H), 7.14 (t, J = 7.4 Hz, 1H), 7.04 (d, J = 7.1 Hz, 2H), 4.28–4.24 (m, 1H), 4.19–4.15 (m, 1H), 3.34–3.28 (m, 1H), 3.09–3.03 (m, 1H), 2.80 (dt, J = 10.3, 4.2 Hz, 2H), 2.70 (qd, J = 8.7, 6.6 Hz, 1H), 2.38–2.33 (m, 1H); **^{13}C NMR** (125 MHz, $CDCl_3$) δ 202.5, 173.5, 140.5, 136.3, 129.4, 128.4 (Three peaks were overlapped.), 126.8, 126.1, 65.7, 65.6, 40.1, 33.5, 30.1; **HRMS (ESI)** calculated for $C_{19}H_{18}O_3Na$: *m/z* 317.1148 ($[M + Na]^+$), found: *m/z* 317.1165 ($[M + Na]^+$); **IR (neat)** 1778, 1692, 1389, 1226, 756 cm^{-1}.

9. Procedures for Mechanistic Studies

Procedure for Reaction in the Presence of TEMPO

A Schlenk tube with a magnetic stir bar was charged with aldehyde **5a** (27 μL, 0.2 mmol, 1.0 equiv), **1e** (6.2 mg, 0.02 mmol, 10 mol%), Selectfluor (142 mg, 0.4 mmol, 2.0 equiv), TEMPO (62.5 mg, 0.40 mmol, 2.0 equiv) and MeCN (1.0 mL) under an argon atmosphere. After being stirred at room temperature for 4 h, the reaction mixture was quenched with H_2O and extracted with ethyl acetate three times. The combined organic layer was dried over Na_2SO_4 and concentrated. The residue was analyzed by ^{1}H NMR.

Procedure for Trapping of *N*-Oxyl Radical Derived from 1e

A Schlenk tube with a magnetic stir bar was charged with **1e** (62.4 mg, 0.20 mmol, 1.0 equiv), Selectfluor (70.8 mg, 0.20 mmol, 1.0 equiv), TEMPO (62.5 mg, 0.40 mmol, 2.0 equiv) and MeCN (1.0 mL). To the solution was added styrene (27.6 μL, 0.24 mmol, 1.2 equiv) via syringe under an argon atmosphere. After being stirred at room temperature for 4 h, the reaction mixture was passed through a short silica gel pad using MeCN/MeOH (v/v = 10/1) as an eluent, and the solution was concentrated. The residue was analyzed by ^{1}H NMR and HRMS.

Procedure for Trapping of *N*-Oxyl Radical Derived from 1e in the absence of Selectfluor

A Schlenk tube with a magnetic stir bar was charged with **1e** (62.4 mg, 0.20 mmol, 1.0 equiv), TEMPO (62.5 mg, 0.40 mmol, 2.0 equiv) and MeCN (1.0 mL). To the solution was added styrene (27.6 μL, 0.24 mmol, 1.2 equiv) via syringe under an argon atmosphere. After being stirred at room temperature for 4 h, the reaction mixture was passed through a short silica gel pad using MeCN/MeOH (v/v = 10/1) as an eluent, and the solution was concentrated. The residue was analyzed by ^{1}H NMR and HRMS.

Procedure for C–H Arylation of Cyclooctane with Isoquinoline

A Schlenk tube with a magnetic stir bar was charged with **1e** (12.5 mg, 0.040 mmol, 20 mol%), Selectfluor (142 mg, 0.40 mmol, 2.0 equiv), isoquinoline (25.8 mg, 0.20 mmol, 1.0 equiv) and MeCN (1.5 mL). To the solution was added cyclooctane (1.0 mL) and trifluoroacetic acid (23.0 µL, 0.30 mmol, 1.5 equiv) via syringe under an argon atmosphere. After being stirred at 80 °C for 4 h, the reaction mixture was cooled to room temperature, quenched with saturated aqueous $NaHCO_3$ and extracted with ethyl acetate three times. The combined organic layer was dried over Na_2SO_4 and concentrated. The residue was purified by flash column chromatography on silica gel to afford the following compound.

1-Cyclooctylisoquinoline (13)[23]

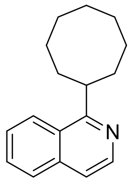

Yellow liquid; 32 mg, 68%.

^{1}**H NMR** (500 MHz, $CDCl_3$) δ 8.46 (d, J = 5.7 Hz, 1H), 8.21 (d, J = 8.5 Hz, 1H), 7.81 (d, J = 8.2 Hz, 1H), 7.67–7.64 (m, 1H), 7.61–7.57 (m, 1H), 7.47 (d, J = 5.7 Hz, 1H), 3.86–3.81 (m, 1H), 2.11–1.97 (m, 4H), 1.90–1.87 (m, 2H), 1.78–1.63 (m, 8H); ^{13}**C NMR** (125 MHz, $CDCl_3$) δ 168.0, 141.9, 136.6, 129.6, 127.7, 126.9, 126.1, 125.0, 118.8, 41.2, 33.2, 26.9, 26.9, 26.4.

10. Computational Studies

All of the calculations were carried out at the DFT level of theory with the dispersion corrected[24] B3LYP-D3 hybrid functional[25] and the 6-311G(d,p) basis sets.[26] The solvation effect was included through the SMD model[27] with a dielectric constant of 35.688 (acetonitrile). First, the approximate reaction coordinates were explored using an automated reaction path search method, called the multi-component artificial force induced reaction (MC-AFIR) method. The artificial forces were applied between the proposed reactive sites. Second, the approximated transition states and intermediates obtained from the MC-AFIR calculations were further optimized without any restrictions. The obtained transition states were confirmed by the frequency calculations and the intrinsic reaction coordinate (IRC) calculations.[28] The Gibbs free energy corrections were calculated at 1 atm and 298.15 K. The MC-AFIR calculations were performed via the global reaction route mapping (GRRM) program,[29] using the energies and energy derivatives computed by the Gaussian09 program.[30] All the other calculations, such as geometry optimizations, the frequency calculations and IRC calculations, were carried out with Gaussian 09 package.[30]

Method for Calculation of Bond Dissociation Energy

The O–H bond dissociation energies (BDEs) of *N*-hydroxyphthalimide (NHPI) and *N*-hydroxybenzimidazoles (NHBIs) have been calculated using isodesmic work reactions (Scheme S-1) to the experimental phenoic O–H BDE (88.74 kcal mol^{-1}).[31]

Scheme S-1

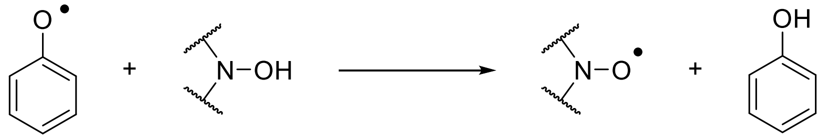

Energetics of Initiation Process

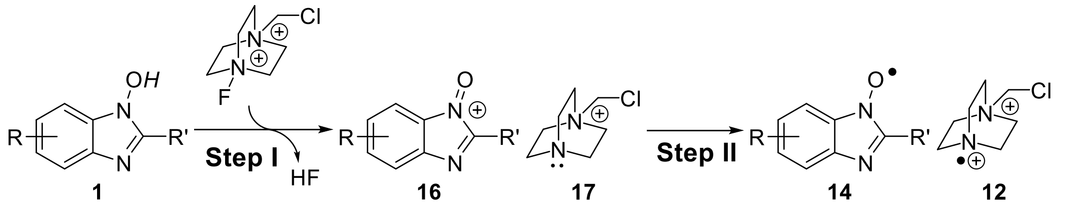

Table S-4. Gibbs Free Energy Differences (Δ*G* in kJ mol^{-1}) of Initial Steps I and II

NHBI	Δ*G*(Step I)	Δ*G*(Step II)	Δ*G*(Steps I + II)
**1a**	-79.7	23.9	-55.8
**1b**	-19.8	-17.3	-19.8
**1c**	-37.7	-2.1	-37.7
**1d**	19.6	-48.8	-29.2
**1e**	25.8	-45.5	-19.7
**1f**	98.6	--	--
**NHPI**	2.1	-34.0	-31.9

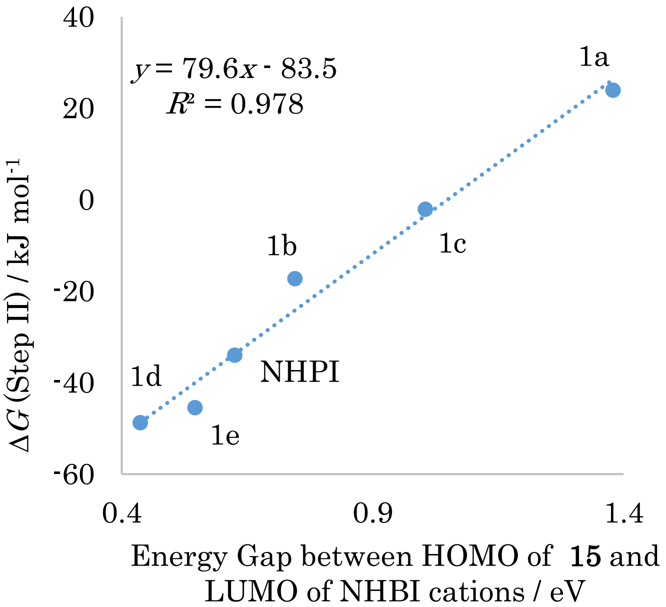

Figure S1. Correlation of energy gap between HOMO of **17** and LUMO of **16** (in eV) and Gibbs free energy difference for step II (in kJ mol^{-1})

Cartesian Coordinates of Optimized Structures

1a 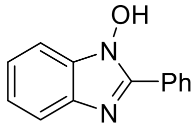

C	1.91980438	-0.56166667	-0.00735414
C	2.00924838	-1.96351967	-0.14336314
C	0.83439038	-2.72331267	-0.21291214
C	-0.37992362	-2.05149767	-0.14008114
C	-0.44091162	-0.64898067	-0.00105614
C	0.71150238	0.12673233	0.06823986
C	4.05733138	-1.25603467	-0.08527214
H	0.88326138	-3.80106067	-0.31873814
H	-1.30470962	-2.61509667	-0.19012614
H	-1.40924162	-0.16454167	0.05244886
H	0.67770938	1.20377033	0.17470986
N	3.23374138	-0.14978067	0.03026386
O	3.61128838	1.17151333	0.13745286
H	3.71605838	1.33774633	1.09144986
N	3.33430438	-2.35755467	-0.18837314
C	5.52502938	-1.22550667	-0.07156314
C	6.20628838	-2.41478267	0.24059386
C	6.27003638	-0.07675967	-0.38278614
C	7.59527238	-2.45114167	0.24793186
H	5.63441238	-3.30326767	0.47678786
C	7.66221338	-0.12172367	-0.37316814
H	5.76740838	0.84355433	-0.64228314
C	8.32987338	-1.30353467	-0.05665314
H	8.10627938	-3.37502767	0.49468386
H	8.22510138	0.77151633	-0.61986914
H	9.41372638	-1.33217767	-0.04889614

1a_radical (**14**) 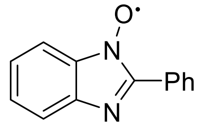

C	-0.78102194	1.59854012	0.00000000
C	-0.87287994	0.20876512	0.15816800
C	0.28477406	-0.55551688	0.24246100
C	1.50871806	0.11671212	0.16148500
C	1.57697806	1.50943912	0.00138600
C	0.41958706	2.28598112	-0.08374400
C	-2.94067094	0.88420312	0.08997000
H	0.23351506	-1.63076188	0.36450800
H	2.42926706	-0.45209088	0.22278900
H	2.54558806	1.99142712	-0.05738800
H	0.44955406	3.36103512	-0.20838700
N	-2.10774894	2.04852712	-0.05097800
O	-2.47712194	3.24371412	-0.23848700
N	-2.21470594	-0.19064588	0.21026200
C	-4.40259794	0.92728712	0.07418300
C	-5.09373894	-0.25724288	-0.23836800
C	-5.13388494	2.08429312	0.38930700
C	-6.48219194	-0.28023588	-0.24206800
H	-4.53071994	-1.14974788	-0.48137900
C	-6.52569994	2.05063912	0.38494800
H	-4.61900594	2.99907712	0.64295700
C	-7.20373994	0.87455912	0.06807000
H	-7.00342894	-1.19770988	-0.49063700
H	-7.08087594	2.94760612	0.63493100
H	-8.28780894	0.85582812	0.06370900

1a_cation (**16**) 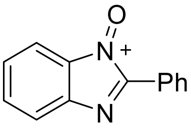

C	0.13869177	-0.06567592	-0.00085982
C	0.04178777	-1.46762792	0.17910318
C	1.18740977	-2.22061592	0.25754518
C	2.41350577	-1.53262092	0.15327518
C	2.50076877	-0.13733292	-0.02475482
C	1.35585077	0.63122808	-0.10761282
C	-2.05542923	-0.88159992	0.12662518
H	1.15075677	-3.29369192	0.38903018
H	3.33167877	-2.10535192	0.20994718
H	3.47388877	0.32958108	-0.09746982
H	1.37518377	1.70491208	-0.24469882
N	-1.13662723	0.40379508	-0.06025582
O	-1.52060723	1.53110608	-0.26583882
N	-1.29861723	-1.89579792	0.24931018
C	-3.48824123	-0.77323692	0.09119718
C	-4.21434123	-1.93231392	-0.26410782
C	-4.17569323	0.40881608	0.43489018
C	-5.59743823	-1.89758592	-0.28998582
H	-3.67805623	-2.83443792	-0.53003382
C	-5.56278823	0.42402208	0.41088418
H	-3.63712623	1.29210008	0.74403218
C	-6.27414323	-0.72016792	0.04544918
H	-6.15330123	-2.78282392	-0.57434782
H	-6.09214723	1.32801208	0.68640618
H	-7.35762823	-0.69751892	0.02580218

1b 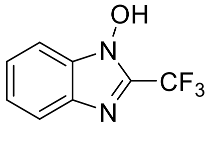

C	0.16289536	0.60798557	0.02249488
C	0.16460536	-0.80534343	0.02980488
C	-1.05308064	-1.49815143	0.02995488

C	-2.22097864	-0.74982243	0.01821788
C	-2.19539464	0.66240557	0.00734588
C	-1.00264464	1.37270857	0.01077288
C	2.22166936	-0.22431943	0.03203988
H	-1.06793864	-2.58164043	0.03824988
H	-3.17899564	-1.25668043	0.01669388
H	-3.13318364	1.20582557	-0.00358412
H	-0.97284964	2.45490957	0.00267988
N	1.50031236	0.93974757	0.04117588
O	2.02470436	2.20520557	-0.07972112
H	2.08712936	2.54836657	0.83010788
N	1.46599236	-1.29085543	0.03424388
C	3.72080836	-0.21344243	0.01675488
F	4.21745736	0.53254257	1.03040088
F	4.20554136	0.31571957	-1.12766612
F	4.21080336	-1.45312043	0.13617588

1b_radical (**14**) 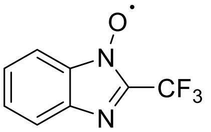

C	0.72271718	0.88313598	0.00210099
C	0.70461518	-0.52041802	0.00209199
C	1.89616018	-1.23377502	0.00226599
C	3.08311718	-0.49552702	0.00258699
C	3.07921718	0.90793198	0.00274399
C	1.88695418	1.63438098	0.00246299
C	-1.36505282	0.05787098	0.00206599
H	1.89695918	-2.31679302	0.00219699
H	4.03142918	-1.01959102	0.00277399
H	4.02246718	1.44079298	0.00310199
H	1.86665218	2.71665298	0.00259899
N	-0.62988982	1.26437998	0.00215899

O	-1.10831582	2.43337198	0.00211299
N	-0.61973682	-0.99625202	0.00206499
C	-2.86681382	0.08411298	0.00242099
F	-3.34733682	0.72542198	-1.08208601
F	-3.34638582	0.72562398	1.08742499
F	-3.36099982	-1.16018702	0.00277299

1b_cation (**16**) 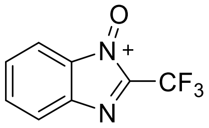

C	2.31367046	0.50400292	-0.02369244
C	2.34659546	-0.90779408	-0.02516644
C	1.18930046	-1.62561908	-0.01671244
C	-0.02538854	-0.87651908	-0.00791844
C	-0.05358054	0.51981792	-0.00882744
C	1.12930946	1.25422992	-0.01690344
C	4.43643146	-0.37308008	-0.02674344
H	1.18434046	-2.70742708	-0.01506144
H	-0.96172754	-1.42099108	-0.00040144
H	-1.00375654	1.03646092	-0.00289744
H	1.14366346	2.33685892	-0.01727244
N	3.62223846	0.91503892	-0.02283044
O	4.10167246	2.02225392	-0.01837644
N	3.69569746	-1.38703908	-0.02884244
C	5.94573646	-0.27782008	-0.01388244
F	6.35787646	0.34211292	1.10157856
F	6.37465246	0.43273592	-1.06671044
F	6.47949746	-1.49539008	-0.06022944

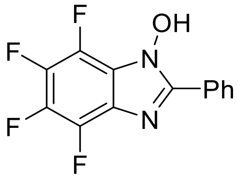

1c

C	-0.53234154	1.78766269	0.05378467
C	-0.47430254	0.37986069	-0.01921333
C	-1.66495254	-0.34957331	-0.05182233
C	-2.86436454	0.33442469	-0.00543033
C	-2.89839854	1.73569969	0.07370867
C	-1.73451454	2.48296869	0.10477367
C	1.58187346	1.03747769	0.00710867
N	0.78829746	2.17012169	0.07594967
O	1.20114346	3.48137369	0.11182967
H	1.29148246	3.70289869	1.05664467
N	0.83199346	-0.04899831	-0.04757633
C	3.04838746	1.03252369	0.02126567
C	3.69740646	-0.14901231	0.41840367
C	3.81905046	2.13870069	-0.36992033
C	5.08494846	-0.21801431	0.43177567
H	3.10428246	-1.00426831	0.71668767
C	5.20924346	2.05976269	-0.35464633
H	3.33943846	3.04988869	-0.69639033
C	5.84649946	0.88711069	0.04740467
H	5.57340046	-1.13365531	0.74519367
H	5.79439746	2.91856969	-0.66330333
H	6.92925546	0.83266369	0.05972267
F	-1.79225354	3.82323469	0.18519167
F	-4.09208054	2.35068669	0.11872367
F	-4.02981354	-0.33628731	-0.03543733
F	-1.65796654	-1.69072631	-0.12255433

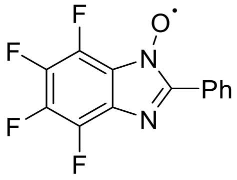

1c_radical (**14**)

C	-0.08029198	0.13868613	0.00000000
C	-0.01416198	1.53873113	0.07955200
C	-1.18574298	2.27751713	0.11422500
C	-2.39967398	1.59604613	0.06348700
C	-2.44652498	0.20294513	-0.02051000
C	-1.27890398	-0.55258087	-0.05260100
C	2.05764202	0.90578913	0.05325800
N	1.25200102	-0.28568687	-0.02330500
O	1.64753202	-1.47889887	-0.14653400
N	1.30733302	1.96936713	0.11283800
C	3.51747702	0.89378113	0.03523100
C	4.17872802	2.07346813	-0.35252400
C	4.27435502	-0.22659487	0.41607900
C	5.56584202	2.12588013	-0.36690500
H	3.59517802	2.93693913	-0.64639100
C	5.66444302	-0.16210887	0.40071400
H	3.78258602	-1.13500187	0.73061900
C	6.31357302	1.00749413	0.00795200
H	6.06565302	3.03731513	-0.67406800
H	6.24106502	-1.02939887	0.70104400
H	7.39684702	1.04949613	-0.00508800
F	-1.34725898	-1.88371987	-0.13128400
F	-3.63795198	-0.40734387	-0.06677700
F	-3.54883898	2.28071613	0.09612300
F	-1.17923698	3.61310813	0.18986100

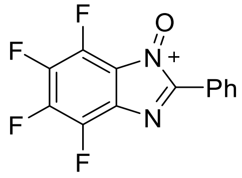

1c_cation (**16**)

C	0.75177919	0.10947967	-0.00840804
C	0.82870219	1.52270867	0.07742396
C	-0.33059581	2.25199267	0.09120396
C	-1.55520181	1.54799667	0.01689096
C	-1.62542681	0.15007667	-0.07220204
C	-0.46281981	-0.59799833	-0.08443504
C	2.92364319	0.96155467	0.06810396
N	2.03312019	-0.34536933	-0.02902204
O	2.42636419	-1.48140333	-0.14763504
N	2.14598719	1.97186567	0.12617396
C	4.35436819	0.88783967	0.02410396
C	5.04309919	2.06029767	-0.36946704
C	5.08062219	-0.26850533	0.38126296
C	6.42445419	2.06158267	-0.42140104
H	4.47835619	2.94167467	-0.64528304
C	6.46560719	-0.24658033	0.32882396
H	4.57469219	-1.15902633	0.72161696
C	7.13844619	0.90868067	-0.07560704
H	6.95116719	2.95448667	-0.73522304
H	7.02504419	-1.12963833	0.61200096
H	8.22142719	0.91381367	-0.11798904
F	-0.49489181	-1.90693733	-0.16340204
F	-2.81420781	-0.43464533	-0.14044704
F	-2.67494781	2.22911867	0.02913396
F	-0.35526181	3.57231267	0.16267396

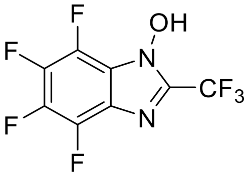

1d

C	-0.82475631	-0.10966350	0.00792832
C	-0.85002231	-1.52188350	0.00971232
C	-2.08046131	-2.18511850	0.00823032
C	-3.23477931	-1.42950750	0.00141532
C	-3.18634531	-0.02369550	-0.00135868
C	-1.98478031	0.65789050	0.00496232
C	1.20969969	-0.98402650	0.01680032
N	0.51563969	0.19712650	0.03179132
O	1.06455269	1.44733050	-0.10600468
H	1.12342569	1.80747450	0.79832632
N	0.43231269	-2.03352050	0.01395332
C	2.71180969	-1.00612750	-0.00034968
F	3.21948769	-0.27031150	1.01183832
F	3.19931669	-0.48899050	-1.14613168
F	3.16937869	-2.25610050	0.11976832
F	-2.14794131	-3.52255050	0.01348632
F	-4.43688631	-2.02682550	-0.00240668
F	-4.34170431	0.65523850	-0.00809168
F	-1.95866931	1.99885950	0.00830432

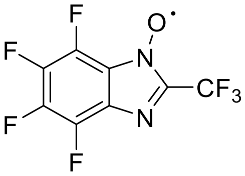

1d_radical (**14**)

C	1.29188213	0.25556324	-0.01356693
C	1.29759113	-1.15002776	-0.01358493
C	2.50161913	-1.83590276	-0.01335893
C	3.67957113	-1.09175876	-0.01314893

C	3.65604213	0.30487124	-0.01313893
C	2.45399213	1.00722424	-0.01337593
C	-0.77225387	-0.61109476	-0.01358293
N	-0.06344087	0.61174724	-0.01348593
O	-0.56256287	1.77026024	-0.01334993
N	-0.00609287	-1.64958276	-0.01358593
C	-2.27675687	-0.61534176	-0.01313693
F	-2.76381487	0.01667324	-1.09758493
F	-2.76275887	0.01667424	1.07195207
F	-2.74101587	-1.86907976	-0.01289293
F	2.55802713	-3.16722276	-0.01334293
F	4.85978113	-1.71632976	-0.01290193
F	4.81346213	0.97097524	-0.01285093
F	2.45545913	2.33879224	-0.01330193

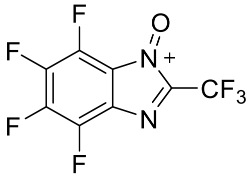

1d_cation (**16**)

C	2.72239464	0.56278411	-0.02091778
C	2.71286564	1.97479011	-0.02107978
C	3.89073464	2.65964111	-0.02029678
C	5.10518764	1.89134411	-0.01952178
C	5.11021164	0.50357111	-0.01963978
C	3.89900864	-0.19880089	-0.02031578
C	0.62775364	1.46449411	-0.02060578
N	1.40572264	0.17315211	-0.02035178
O	0.91798464	-0.93189689	-0.01969478
N	1.39072964	2.46886011	-0.02113278
C	-0.88535336	1.41098911	-0.01949878
F	-1.32105036	0.76284211	1.06895922
F	-1.32242936	0.75257611	-1.10121478

F	-1.38015536	2.64453111	-0.02503878
F	3.98247964	3.96591211	-0.02007478
F	6.24121764	2.53577711	-0.01880778
F	6.25757164	-0.15044289	-0.01914778
F	3.88078264	-1.49992889	-0.02029378

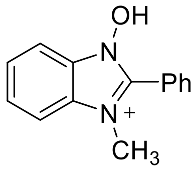

1e

C	-1.61300054	0.35766423	0.02111533
C	-1.60087754	1.74781123	-0.14889467
C	-0.40142254	2.45438523	-0.21825667
C	0.76649246	1.70960323	-0.11036567
C	0.74378646	0.31140623	0.06114433
C	-0.44943754	-0.39631177	0.13076133
C	-3.74167754	1.10348423	-0.09426267
H	-0.38575154	3.52853423	-0.34660067
H	1.72154146	2.21859923	-0.15773867
H	1.68117746	-0.22561877	0.14075133
H	-0.48330654	-1.46960377	0.26233433
N	-2.95367754	0.01314323	0.05277933
O	-3.41757754	-1.26808177	0.17397133
H	-3.48643554	-1.43618477	1.13323433
C	-3.32498754	3.55634923	-0.48645667
H	-3.27464054	4.13325623	0.43701533
H	-2.63976054	3.97308323	-1.22303667
H	-4.33587054	3.57491223	-0.88397667
N	-2.92831854	2.16830523	-0.22736867
C	-5.20210354	1.08539623	-0.07922867
C	-5.90836254	2.02749823	0.68428133
C	-5.89683754	0.11056123	-0.81186867

C	-7.29828954	1.99306423	0.70776933
H	-5.37566354	2.76455923	1.27178533
C	-7.28639054	0.09152323	-0.78612067
H	-5.35328454	-0.61523377	-1.40183867
C	-7.98792854	1.02950223	-0.02784267
H	-7.84118654	2.71640323	1.30449333
H	-7.82162154	-0.65583077	-1.35981867
H	-9.07154354	1.00755323	-0.00833267

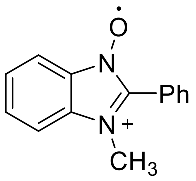

1e_radical (**14**)

C	-0.88310671	0.34296065	0.01605102
C	-0.87551071	1.72601365	-0.15791298
C	0.31020629	2.43854565	-0.22499198
C	1.48785429	1.69425965	-0.11095798
C	1.47313729	0.30244865	0.06239002
C	0.27610929	-0.40864135	0.13039602
C	-3.03333271	1.11201765	-0.10803498
H	0.32556729	3.51224165	-0.35429898
H	2.43858729	2.21105865	-0.15599998
H	2.41125429	-0.23182835	0.14635902
H	0.24155129	-1.48147735	0.26486002
N	-2.23342171	-0.03790235	0.04727102
O	-2.67620471	-1.20199635	0.24153702
C	-2.59415671	3.54289165	-0.51953298
H	-2.55507471	4.11839465	0.40556002
H	-1.88543071	3.94679765	-1.24014898
H	-3.59653271	3.56821365	-0.93768098
N	-2.21754271	2.15176265	-0.24419998
C	-4.48145271	1.08138665	-0.08803598

C	-5.19959171	2.06694165	0.61349002
C	-5.16822971	0.04879065	-0.75305298
C	-6.58675171	2.01674765	0.64267902
H	-4.67573771	2.84122265	1.15866502
C	-6.55575671	0.02030665	-0.72641498
H	-4.61950471	-0.70606335	-1.29911598
C	-7.26567171	0.99929665	-0.02931998
H	-7.13781271	2.76833665	1.19497202
H	-7.08448471	-0.76621735	-1.25127498
H	-8.34890771	0.96749065	-0.00734198

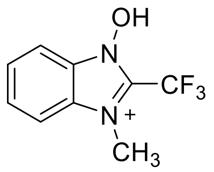

1g

C	3.52075789	0.49112272	-0.17809842
C	3.53774589	1.89393772	-0.17527342
C	2.34933489	2.62772472	-0.17573442
C	1.17314789	1.89516372	-0.17210842
C	1.16822389	0.48270672	-0.16818442
C	2.34264189	-0.25173628	-0.17167942
C	5.63907589	1.19961872	-0.18540842
H	2.34930289	3.70925472	-0.18209242
H	0.22679889	2.42187972	-0.17277742
H	0.21939589	-0.03947028	-0.16363442
H	2.35741189	-1.33329528	-0.16947242
N	4.85020089	0.11101972	-0.19398642
O	5.28845989	-1.17471028	-0.04519242
H	5.36329989	-1.53618528	-0.94997542
C	5.32792089	3.69483072	-0.16762542
H	5.50390689	4.01654572	-1.19321942
H	4.54440689	4.29604872	0.28534858

H	6.23412689	3.78334972	0.42458158
N	4.87273689	2.29380172	-0.16652342
C	7.15193589	1.16363672	-0.17355642
F	7.64839689	2.19602772	-0.87011542
F	7.62209989	1.24226072	1.07934858
F	7.60476189	0.03244972	-0.72288242

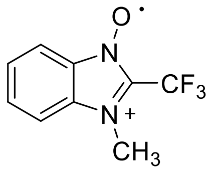

1g_radical (**14**)

C	-0.09488238	0.56204379	-0.00124208
C	-0.09385738	1.95961279	-0.00120208
C	-1.27987538	2.67915079	-0.00093308
C	-2.45800938	1.92873579	-0.00070808
C	-2.44838238	0.52690079	-0.00079308
C	-1.25430438	-0.19440321	-0.00106808
C	2.03188662	1.32005079	-0.00137308
H	-1.30003238	3.76001579	-0.00085708
H	-3.40669438	2.45053079	-0.00045408
H	-3.38840738	-0.01014721	-0.00061808
H	-1.22539038	-1.27573021	-0.00110708
N	1.25584862	0.17242879	-0.00131808
O	1.71631662	-0.99877621	-0.00120108
C	1.68617362	3.79575779	-0.00124208
H	2.27568862	3.98175679	-0.89670108
H	0.79998062	4.42109479	-0.00079208
H	2.27639962	3.98150079	0.89379692
N	1.25251862	2.38472179	-0.00129308
C	3.54280762	1.22251579	-0.00089808
F	4.10903662	2.43111479	-0.00197008
F	3.95648562	0.55682079	1.08477992

F	3.95717162	0.55458279	-1.08490408

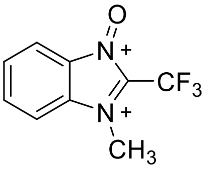

1g_cation (**16**)

C	0.57657295	0.81019898	0.01060535
C	0.59674895	2.21455298	0.01611535
C	-0.55395105	2.93962398	0.04347335
C	-1.77482505	2.18180498	0.06053035
C	-1.79978605	0.79221798	0.04944635
C	-0.61186905	0.05983998	0.02451235
C	2.72754195	1.59796698	0.01401235
H	-0.57914705	4.02049598	0.05884535
H	-2.70647605	2.73300998	0.08213735
H	-2.74647205	0.26984598	0.06152535
H	-0.59380405	-1.02291802	0.01789135
N	1.87921295	0.38772198	0.01353035
O	2.35143095	-0.72614302	0.02135235
C	2.41725395	4.05308698	0.01150435
H	3.01268395	4.21349798	-0.88625865
H	1.53682895	4.68512998	0.00951835
H	3.01126595	4.21661798	0.90981735
N	1.97424095	2.63688598	0.01280735
C	4.24799995	1.44123098	0.04372835
F	4.84559595	2.62303598	-0.01233765
F	4.58220595	0.81958598	1.17372335
F	4.62391895	0.69620698	-0.99340865

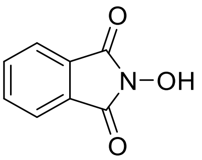

NHPI

C	1.58380596	0.15328467	-0.02073316
C	1.58380596	-1.24431333	-0.02073316
C	2.76364596	-1.96719633	-0.01799816
C	3.96309096	-1.24417333	-0.01830716
C	3.96309096	0.15314467	-0.01830716
C	2.76364596	0.87616767	-0.01799816
C	0.17419696	0.63477767	-0.02028716
C	0.17419696	-1.72580633	-0.02028816
H	2.75865796	-3.05058533	-0.01694116
H	4.90689996	-1.77679333	-0.01874516
H	4.90689996	0.68576467	-0.01874516
H	2.75865796	1.95955667	-0.01694116
O	-1.95330804	-0.54551433	-0.12658416
H	-2.31272104	-0.54551433	0.77677384
O	-0.26847504	1.75696867	-0.04288916
O	-0.26847504	-2.84799733	-0.04288916
N	-0.58812104	-0.54551433	0.02236484

NHPI radical (**14**) 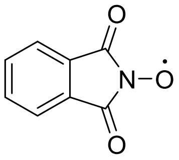

C	-0.08026071	0.28464027	0.00477709
C	-0.08026071	0.28464027	-1.39803291
C	-0.07974671	1.46899427	-2.11895291
C	-0.07936171	2.66503627	-1.39666391
C	-0.07936171	2.66503627	0.00340809
C	-0.07974671	1.46899427	0.72569709
C	-0.07985871	-1.10167873	0.50542109
C	-0.07985871	-1.10167873	-1.89867691
H	-0.07978671	1.46405127	-3.20201191
H	-0.07896071	3.60924127	-1.92802491

H	-0.07896071	3.60924127	0.53476909
H	-0.07978671	1.46405127	1.80875609
O	-0.07925971	-3.18230573	-0.69662791
O	-0.07946571	-1.55195673	1.61767309
O	-0.07946571	-1.55195673	-3.01092891
N	-0.07979671	-1.92263973	-0.69662791

NHPI cation (**16**) 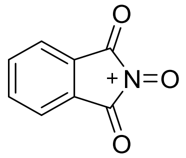

C	1.43792063	0.41614594	0.00544642
C	1.43792063	0.41614594	1.43370442
C	1.43787363	1.61006894	2.14616742
C	1.43777363	2.79965194	1.42295542
C	1.43777363	2.79965194	0.01619542
C	1.43787363	1.61006894	-0.70701658
C	1.43794263	-0.90465406	-0.57935558
C	1.43794263	-0.90465406	2.01850642
H	1.43784763	1.60868394	3.22853542
H	1.43772263	3.74426294	1.95206542
H	1.43772263	3.74426294	-0.51291458
H	1.43784763	1.60868394	-1.78938458
O	1.43757763	-2.99026206	0.71957542
O	1.43797663	-1.40450706	-1.64791758
O	1.43797663	-1.40450706	3.08706842
N	1.43784563	-1.81911906	0.71957542

Cyclooctane 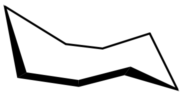

C	-0.46031749	-1.26984125	0.00000000
C	-1.86380549	-0.97351825	0.56678400

C	-1.86992349	-4.18237025	0.56652600
C	-3.04372049	-1.25143025	-0.39338600
C	-3.04861849	-3.89989625	-0.39362300
C	-3.79720449	-2.57436425	-0.16278300
H	0.26673651	-1.23391025	0.82131600
H	-1.87862949	0.08383675	0.85246000
H	-3.77910449	-0.44682925	-0.28413700
H	-2.02434549	-3.62741925	1.49613800
H	-3.78677649	-4.70197725	-0.28449900
H	-0.19168949	-0.45077225	-0.67901100
H	-2.01959349	-1.52860525	1.49606800
H	-1.88824949	-5.23985825	0.85149100
H	-2.70011049	-1.19795025	-1.43338500
H	-2.70500249	-3.95453925	-1.43357600
H	-4.17669249	-2.57366125	0.86799000
H	-4.68261049	-2.57275625	-0.81046800
C	-0.27962449	-2.58056125	-0.77739200
H	0.73505851	-2.58255325	-1.19327900
H	-0.94823549	-2.57933125	-1.64467100
C	-0.46523749	-3.89042325	0.00031100
H	-0.19887849	-4.71050925	-0.67835700
H	0.26124851	-3.92857825	0.82203500

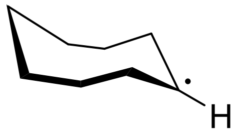

Cyclooctanyl radical

C	2.25319599	-0.25396825	-0.05900202
C	1.96076699	-1.62570125	0.57790298
C	5.15780699	-1.62635925	0.57790298
C	2.20807699	-2.84503425	-0.34379802
C	4.90998499	-2.84560025	-0.34379502
C	3.55889299	-3.49836725	-0.29776602

H	2.22599699	0.51428975	0.72402398
H	0.90582499	-1.63084925	0.87226098
H	1.47085399	-3.61820525	-0.10153402
H	4.59164399	-1.75148825	1.50381598
H	5.64687999	-3.61908025	-0.10152102
H	1.42905899	-0.02037325	-0.74464802
H	2.52688199	-1.75108825	1.50381098
H	6.21274899	-1.63193825	0.87225698
H	1.96776099	-2.54556325	-1.38080702
H	5.15043699	-2.54624525	-1.38080702
H	3.55866399	-4.56585725	-0.50821202
C	3.55959999	-0.12240425	-0.85197202
H	3.55980399	0.86135275	-1.33594902
H	3.55944999	-0.85037025	-1.67096702
C	4.86595199	-0.25450925	-0.05900702
H	5.69018799	-0.02126825	-0.74465402
H	4.89347299	0.51374375	0.72401398

Selectfluor 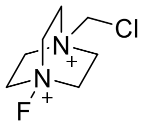

C	0.50360650	0.08029197	0.00659257
C	1.96926850	0.45036197	0.30175557
H	0.20087850	0.30977297	-1.01174443
H	-0.17087050	0.53332097	0.72880357
H	2.32126250	1.18595497	-0.41728043
H	2.10357450	0.81696997	1.31647257
C	1.05806350	-2.11500103	-0.97820243
H	1.07824050	-3.17265203	-0.72716043
H	0.45016750	-1.93439803	-1.86126343
C	2.46159350	-1.50074603	-1.12783343
H	3.18693650	-2.28497703	-1.32341743

H	2.49993050	-0.74870203	-1.91298843
C	2.51709150	-1.71988203	1.34007557
H	2.97565650	-2.68256603	1.13422557
H	2.94534750	-1.27900903	2.23673357
C	0.98744750	-1.84552903	1.47803257
H	0.68865950	-2.87941603	1.63165757
H	0.57028650	-1.20006303	2.24695957
N	0.39952550	-1.40671503	0.16904357
N	2.83211350	-0.80209003	0.16899357
C	4.27109450	-0.34755803	0.17008757
H	4.42990050	0.23937897	-0.72964243
H	4.42915550	0.23687097	1.07204257
Cl	5.42947750	-1.70976403	0.16781557
F	-0.95316250	-1.74874003	0.16754057

12 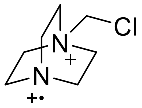

C	1.48172256	1.11678640	-0.00955393
C	2.93916556	1.64192940	0.24394107
H	1.13877156	1.35593140	-1.01267893
H	0.81065156	1.51253940	0.75101407
H	3.20818956	2.37972940	-0.50750293
H	3.05138156	2.04315940	1.24803507
C	2.19786656	-0.97937560	-1.01635393
H	2.29499556	-2.04237760	-0.81197093
H	1.59929356	-0.79562960	-1.90706393
C	3.60058156	-0.28845160	-1.16704793
H	4.36410356	-1.04479760	-1.32259393
H	3.60533456	0.44368640	-1.97096893
C	3.66804056	-0.47182860	1.31015207
H	4.22002456	-1.38803560	1.12521307

H	4.02291656	0.02714940	2.20895207
C	2.12625656	-0.75826760	1.40934907
H	1.95657856	-1.82632160	1.53481007
H	1.66301556	-0.18425560	2.20729407
N	1.61098356	-0.31789160	0.12925907
N	3.89944756	0.45753040	0.12550607
C	5.29619556	1.03462640	0.12575007
H	5.40462656	1.63345840	-0.77431393
H	5.40377456	1.63105340	1.02754007
Cl	6.56403156	-0.22746160	0.12689407

12-H 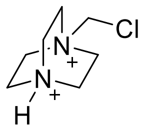

C	0.70802924	0.21166069	0.00277079
C	2.13891524	0.68429569	0.29794379
H	0.39488724	0.44771469	-1.01172921
H	0.00623224	0.63357969	0.71786179
H	2.44423324	1.45531069	-0.40492621
H	2.25475124	1.04199069	1.31854779
C	1.41144124	-1.91536431	-1.01580821
H	1.52043424	-2.97418231	-0.79159121
H	0.80600924	-1.78523731	-1.90947721
C	2.76606724	-1.20667331	-1.16423521
H	3.55151124	-1.92816331	-1.36997421
H	2.75393824	-0.44242431	-1.93899321
C	2.84948824	-1.45601331	1.29923279
H	3.37956624	-2.37652031	1.07233779
H	3.25928024	-0.99990631	2.19774479
C	1.33707224	-1.69297531	1.43656079
H	1.12395524	-2.74575131	1.60500079
H	0.89322424	-1.09343831	2.22802779

N	0.68398824	-1.28642431	0.14229379
N	3.09034324	-0.49965431	0.14153279
C	4.48791424	0.06013969	0.14301179
H	4.60525324	0.66049169	-0.75496021
H	4.60577224	0.65193369	1.04659879
Cl	5.74553324	-1.21552831	0.14089579
H	-0.28826776	-1.60844131	0.14265379

17 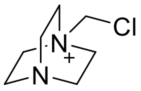

C	1.51094899	-0.13867424	-0.00181535
C	0.06694999	0.39057776	-0.19970735
H	1.88637599	0.14579776	0.98170365
H	2.16027699	0.30021776	-0.76008535
H	-0.22571501	1.10295576	0.56950765
H	-0.08943401	0.82733176	-1.18491435
C	0.82861899	-2.18444924	1.03109265
H	0.75156099	-3.26126124	0.87681665
H	1.39474999	-2.00687624	1.94604065
C	-0.58447001	-1.55978324	1.17399165
H	-1.35430101	-2.31649024	1.30284465
H	-0.64637801	-0.83352524	1.98345465
C	-0.63709001	-1.70529224	-1.30105235
H	-1.22650901	-2.60523624	-1.14340335
H	-0.99847301	-1.18074924	-2.18427835
C	0.88415899	-2.00789324	-1.35700735
H	1.03230999	-3.07658524	-1.51465435
H	1.34677299	-1.47021824	-2.18527535
N	1.54982999	-1.60290624	-0.11019335
N	-0.89129701	-0.79810024	-0.10590835
C	-2.27567801	-0.23057724	-0.10590335

H	-2.39821301	0.36528576	0.79408665
H	-2.39662301	0.36632976	-1.00551635
Cl	-3.56017701	-1.49102324	-0.10901135

HF

F	1.17518255	-0.00729864	-0.00009554
H	1.17518255	-0.00729864	-0.92676454

11. References

(1) Delcros, J. G.; Tomasi, S.; Carrington, S.; Martin, B.; Renault, J.; Blagbrough, I. S.; Uriac, P. *J. Med. Chem.* **2002**, *45*, 5098.

(2) Brehma, E.; Breinbauer, R. *Org. Biomol. Chem.* **2013**, *11*, 4750.

(3) Zhang, P.; Brozek, L. A.; Morken, J. P. *J. Am. Chem. Soc.* **2010**, *132*, 10686.

(4) Kubota, K.; Yamamoto, E.; Ito, H. *J. Am. Chem. Soc.* **2015**, *137*, 420.

(5) Shiroodi, R. K.; Dudnik, A. S.; Gevorgyan, V. *J. Am. Chem. Soc.* **2012**, *134*, 6928.

(6) (a) Kano, T.; Yamaguchi, Y.; Maruoka, K. *Angew. Chem., Int. Ed.* **2009**, *48*, 1838.

(b) Kano, T.; Yamaguchi, Y.; Maruoka, K. *Chem.–Eur. J.* **2009**, *15*, 6678.

(c) Gianelli, C.; Sambri, L.; Carlone, A.; Bartoli, G.; Melchiorre, P. *Angew. Chem., Int. Ed.* **2008**, *47*, 8700.

(7) Han, J.; Wu, H.; Teng, M.; Li, Z.; Wang, Y.; Wang, L.; Pan, Y. *Synlett* **2009**, *6*, 933.

(8) Katayev, D.; Matoušek, V.; Koller, R.; Togni, A. *Org. Lett.* **2015**, *17*, 5898.

(9) Amaoka, Y.; Kamijo, S.; Hoshikawa, T.; Inoue, M. *J. Org. Chem.* **2012**, *77*, 9959.

(10) Morimoto, H.; Fujiwara, R.; Shimizu, Y.; Morisaki, K.; Ohshima, T. *Org. Lett.* **2014**, *16*, 2018.

(11) Nordstrøm, L. U.; Vogt, H.; Madsen, R. *J. Am. Chem. Soc.* **2008**, *130*, 17672.

(12) Starkov, P.; Sheppard, T. D. *Org. Biomol. Chem.* **2011**, *9*, 1320.

(13) Simmons, B. J.; Hoffmann, M.; Hwang, J.; Jackl, M. K.; Garg, N. K. *Org. Lett.* **2017**, *19*, 1910.

(14) Ueda, T.; Konishi, H.; Manabe, K. *Org. Lett.* **2013**, *15*, 5370.

(15) Wu, Z.; Hull, K. L. *Chem. Sci.* **2016**, *7*, 969.

(16) Murphy, J. A.; Commeureuc, A. G. J.; Snaddon, T. N.; McGuire, T. M.; Khan, T. A.; Hisler, K; Dewis, M. L.; Carling, R. *Org. Lett.* **2005**, *7*, 1427.

(17) Wheeler, P.; Vora, H. U.; Rovis, T. *Chem. Sci.* **2013**, *4*, 1674.

(18) Zhang, J.; Hong, S. H. *Org. Lett.* **2012**, *14*, 4646.

(19) Iwasaki, T.; Maegawa, Y.; Hayashi, Y.; Ohshima, T.; Mashima, K. *J. Org. Chem.* **2008**, *73*, 5147.

(20) Uno, T.; Inokuma, T.; Takemoto, Y. *Chem. Commun.* **2012**, *48*, 1901.

(21) Briot, A.; Baehr, C.; Brouillard, R.; Wagner, A.; Mioskowski, C. *J. Org. Chem.* **2004**, *69*, 1374.

(22) Zhuo, L.-G.; Yao, Z.-K.; Yu, Z.-X. *Org. Lett.* **2013**, *15*, 4634.

(23) Zhou, L.; Togo, H. *Eur. J. Org. Chem.* **2019**, *7*, 1627.

(24) Grimme, S.; Anthony, J.; Ehrlich, S.; Krieg, H. *J. Chem. Phys.* **2010**, *132*, 154104.

(25) (a) Becke, A. D. *Phys. Rev. A* **1988**, *38*, 3098. (b) Becke, A. D. *J. Chem. Phys.* **1993**, *98*, 1372. (c) Lee, C.; Yang, W.; Parr, R. G. *Phys. Rev. B* **1988**, *37*, 785.

(26) (a) Krishnan, R.; Binkley, J. S.; Seeger, R.; Pople, J. A. *J. Chem. Phys.* **1980**, *72*, 650. (b) McLean, A. D.; Chandler, G. S. *J. Chem. Phys.* **1980**, *72*, 5639.

(27) Marenich, A. V.; Cramer, C. J.; Truhlar, D. G. *J. Phys. Chem. B* **2009**, *113*, 6378.

(28) Fukui, K. *Acc. Chem. Res.* **1981**, *14*, 363.

(29) (a) Maeda, S.; Ohno, K.; Morokuma, K. *Phys. Chem. Chem. Phys.* **2013**, *15*, 3683. (b) Maeda, S.; Harabuchi, Y.; Takagi, M.; Taketsugu, T.; Morokuma, K. *Chem. Rec.* **2016**, *16*, 2232. (c) Maeda, S.; Harabuchi, Y.; Takagi, M.; Saita, K.; Suzuki, K.; Ichino, T.; Sumiya, Y.; Sugiyama, K.; Ono, Y. *J. Comput. Chem.* **2018**, *39*, 233.

(30) Gaussian 09, Revision E.01, Frisch, M. J.; Trucks, G. W.; Schlegel, H. B.; Scuseria, G. E.; Robb, M. A.; Cheeseman, J. R.; Scalmani, G.; Barone, V.; Petersson, G. A.; Nakatsuji, H.; Li, X.; Caricato, M.; Marenich, A. V.; Bloino, J.; Janesko, B. G.; Gomperts, R.; Mennucci, B.; Hratchian, H. P.; Ortiz, J. V.; Izmaylov, A. F.; Sonnenberg, J. L.; Williams-Young, D.; Ding, F.; Lipparini, F.; Egidi, F.; Goings, J.; Peng, B.; Petrone, A.; Henderson, T.; Ranasinghe, D.; Zakrzewski, V. G.; Gao, J.; Rega, N.; Zheng, G.; Liang, W.; Hada, M.; Ehara, M.; Toyota, K.; Fukuda, R.; Hasegawa, J.; Ishida, M.; Nakajima, T.; Honda, Y.; Kitao, O.; Nakai, H.; Vreven, T.; Throssell, K.; Montgomery, J. A., Jr.; Peralta, J. E.; Ogliaro, F.; Bearpark, M. J.; Heyd, J. J.; Brothers, E. N.; Kudin, K. N.; Staroverov, V. N.; Keith, T. A.; Kobayashi, R.; Normand, J.; Raghavachari, K.; Rendell, A. P.; Burant, J. C.; Iyengar, S. S.; Tomasi, J.; Cossi, M.; Millam, J. M.; Klene, M.; Adamo, C.; Cammi, R.; Ochterski, J. W.; Martin, R. L.; Morokuma, K.; Farkas, O.; Foresman, J. B.; Fox, D. J. Gaussian, Inc., Wallingford CT, 2016.

(31) Borges dos Santos, R. M.; Martinho Simoes, J. A. *J. Phys. Chem. Ref. Data* **1998**, *27*, 707.

12. NMR Spectra of Products

^{1}H NMR of spectrum of **S1** (500 MHz, $CDCl_3$)

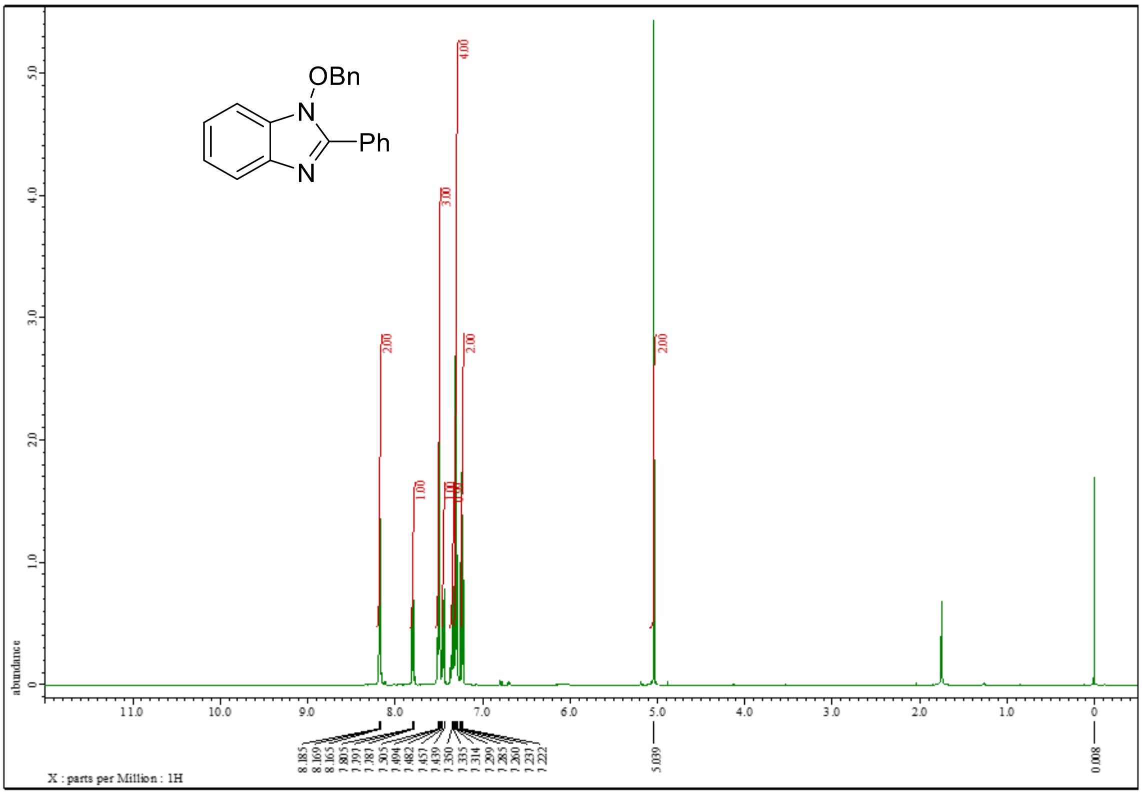

^{13}C NMR of spectrum of **S1** (125 MHz, $CDCl_3$)

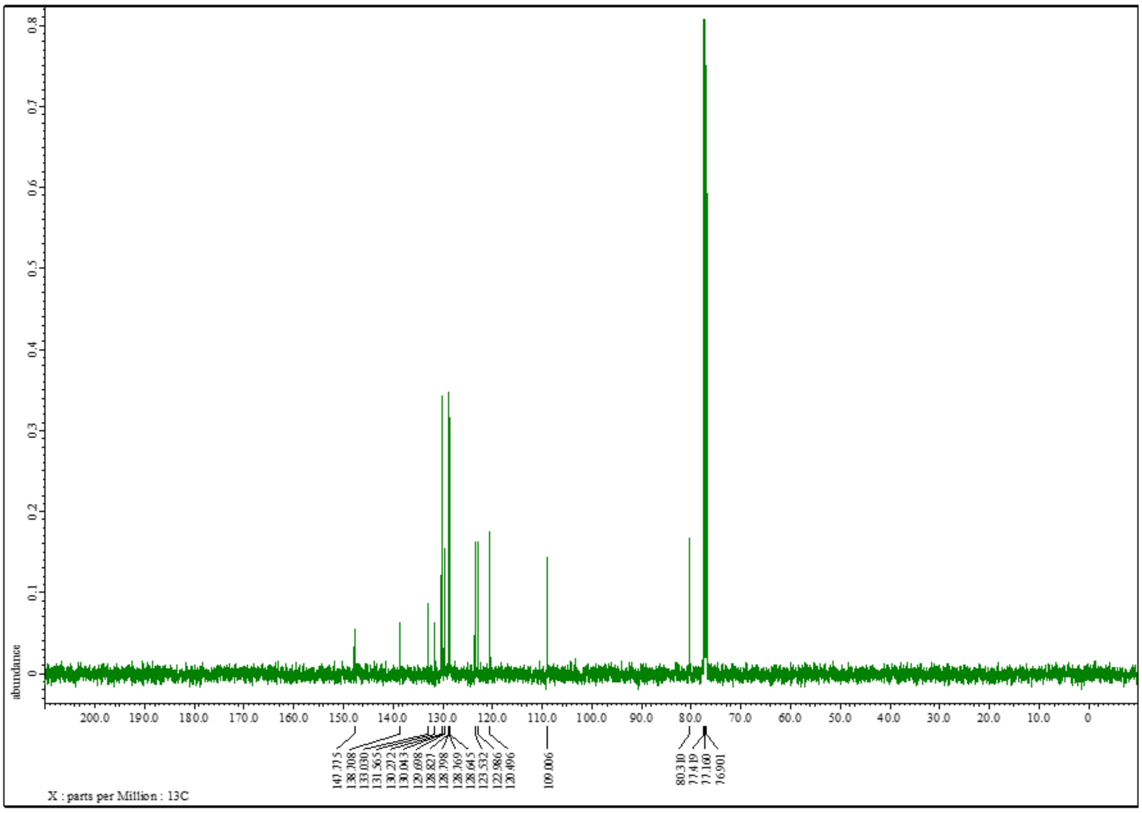

^{1}H NMR of spectrum of **1a** (500 MHz, DMSO-d_6)

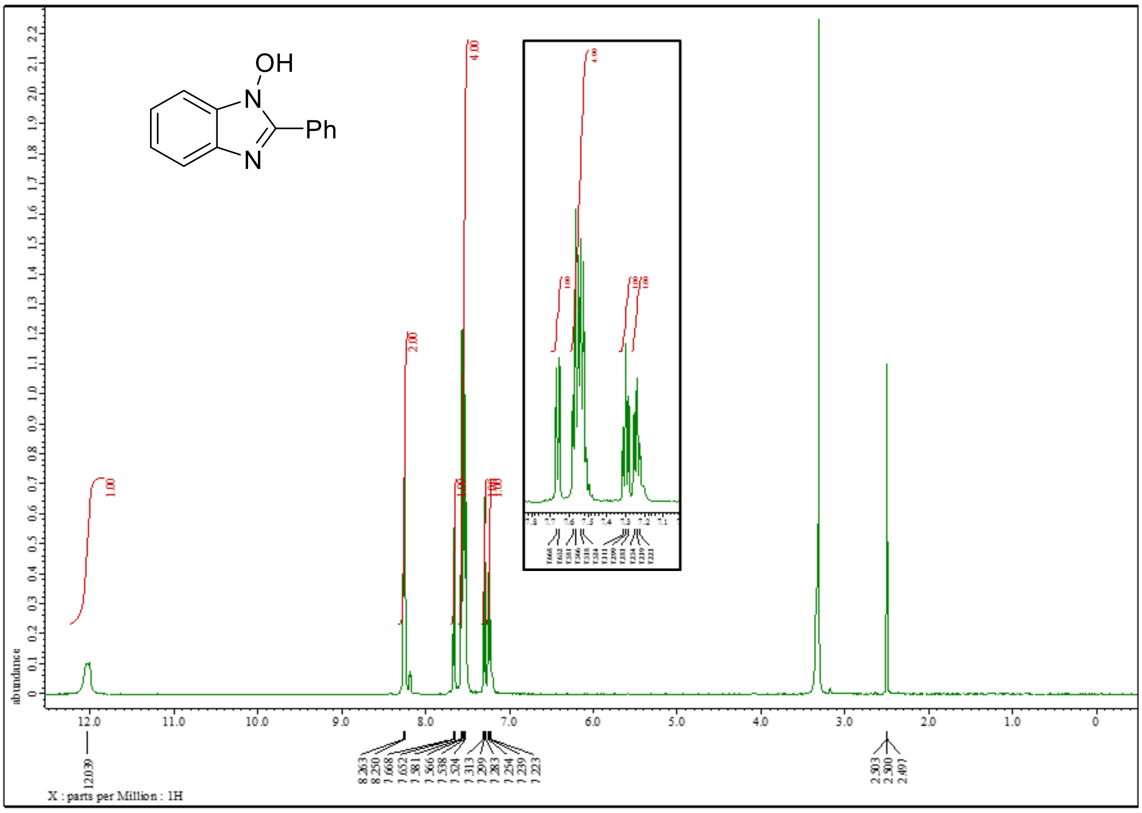

^{13}C NMR of spectrum of **1a** (125 MHz, DMSO-d_6)

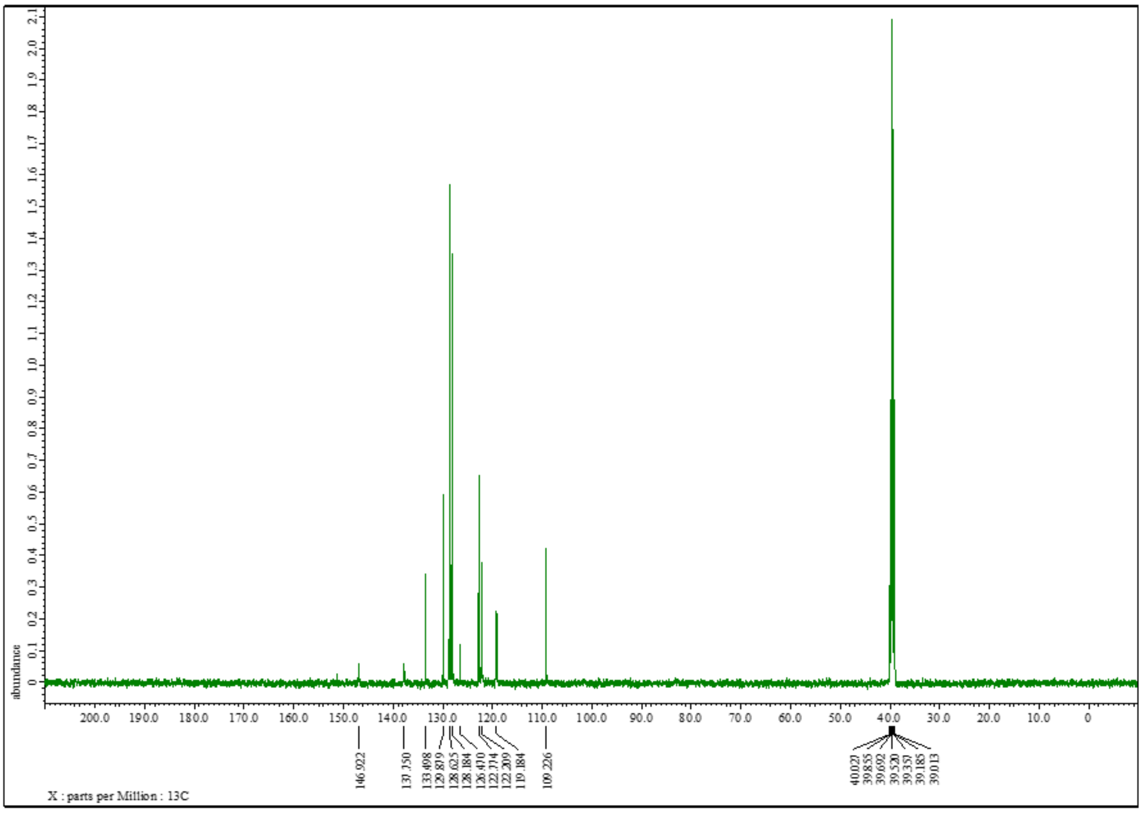

^{13}C NMR of spectrum of **S2** (125 MHz, $CDCl_3$)

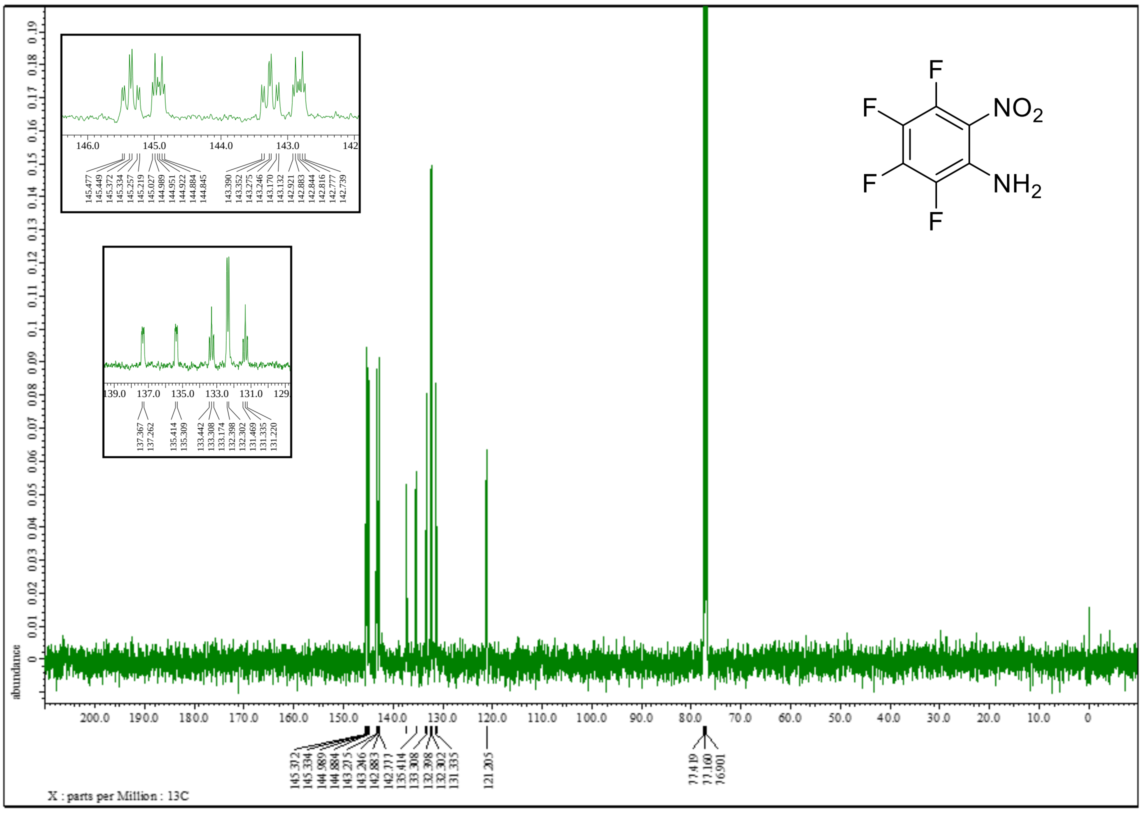

^{19}F NMR of spectrum of **S2** (470 MHz, $CDCl_3$)

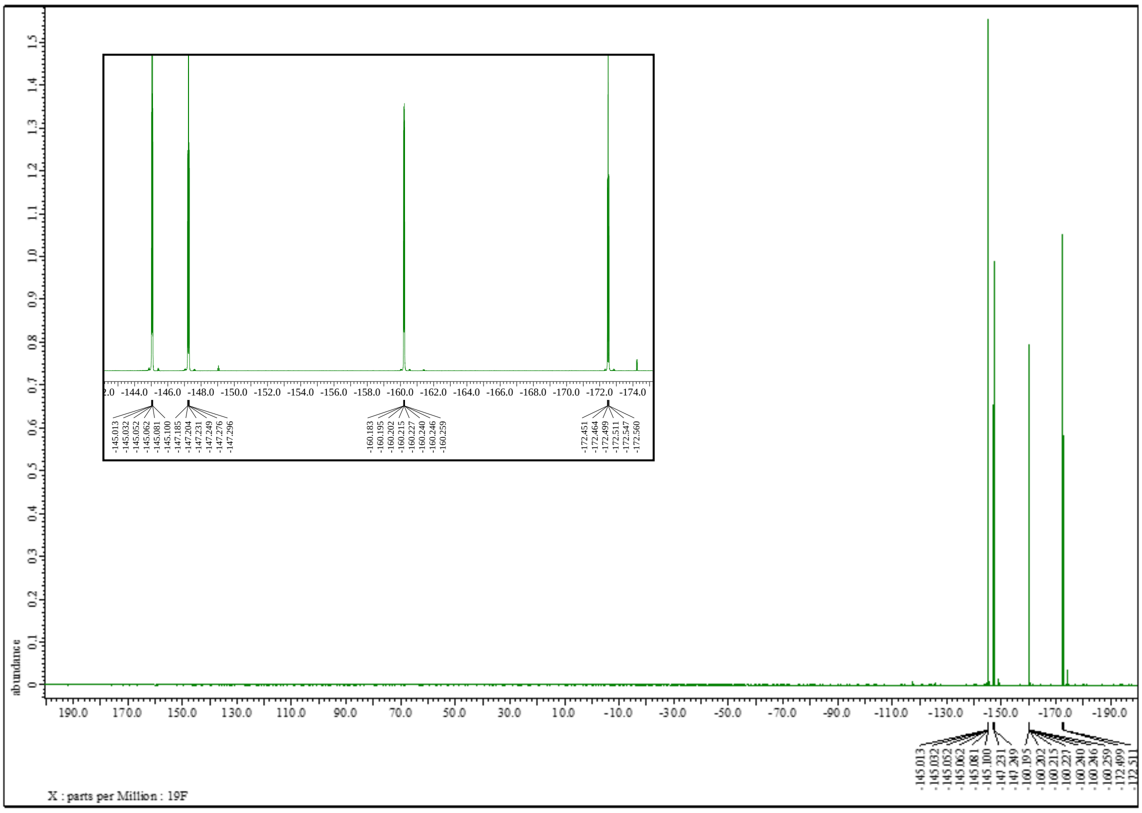

^{1}H NMR of spectrum of **S3** (500 MHz, $CDCl_3$)

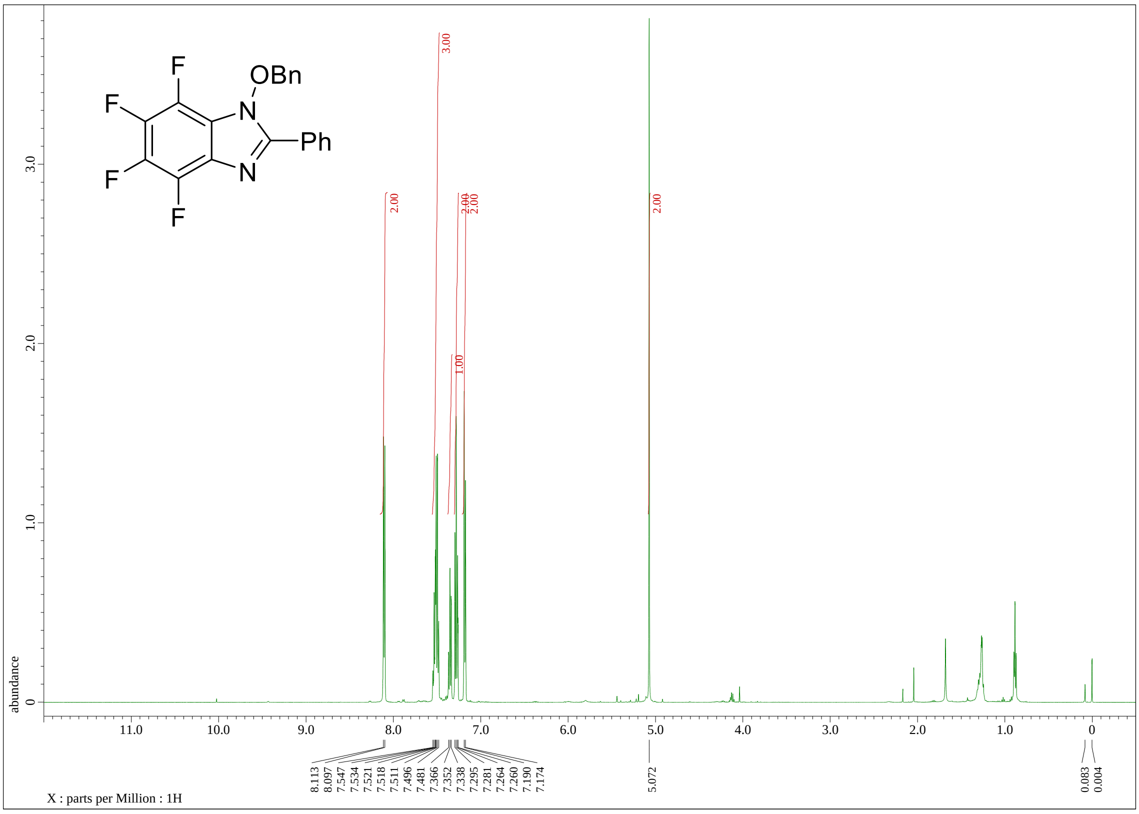

^{13}C NMR of spectrum of **S3** (125 MHz, $CDCl_3$)

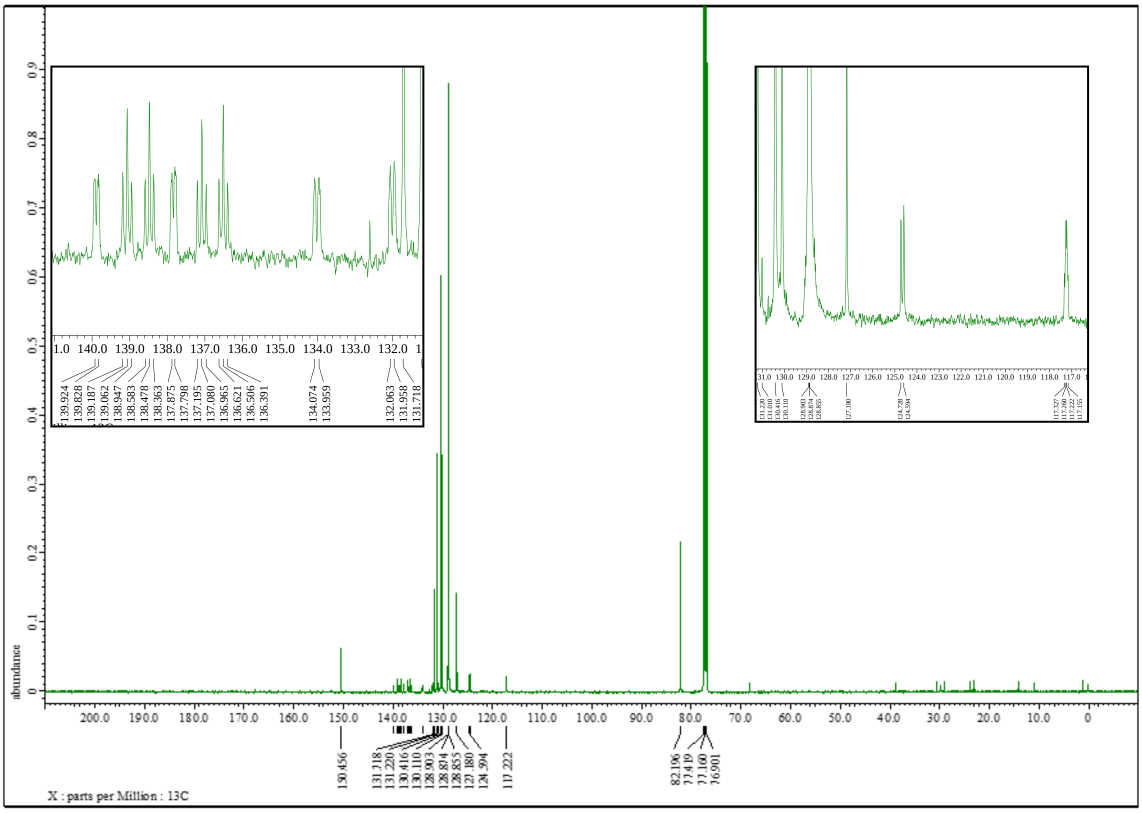

^{19}F NMR of spectrum of **S3** (470 MHz, $CDCl_3$)

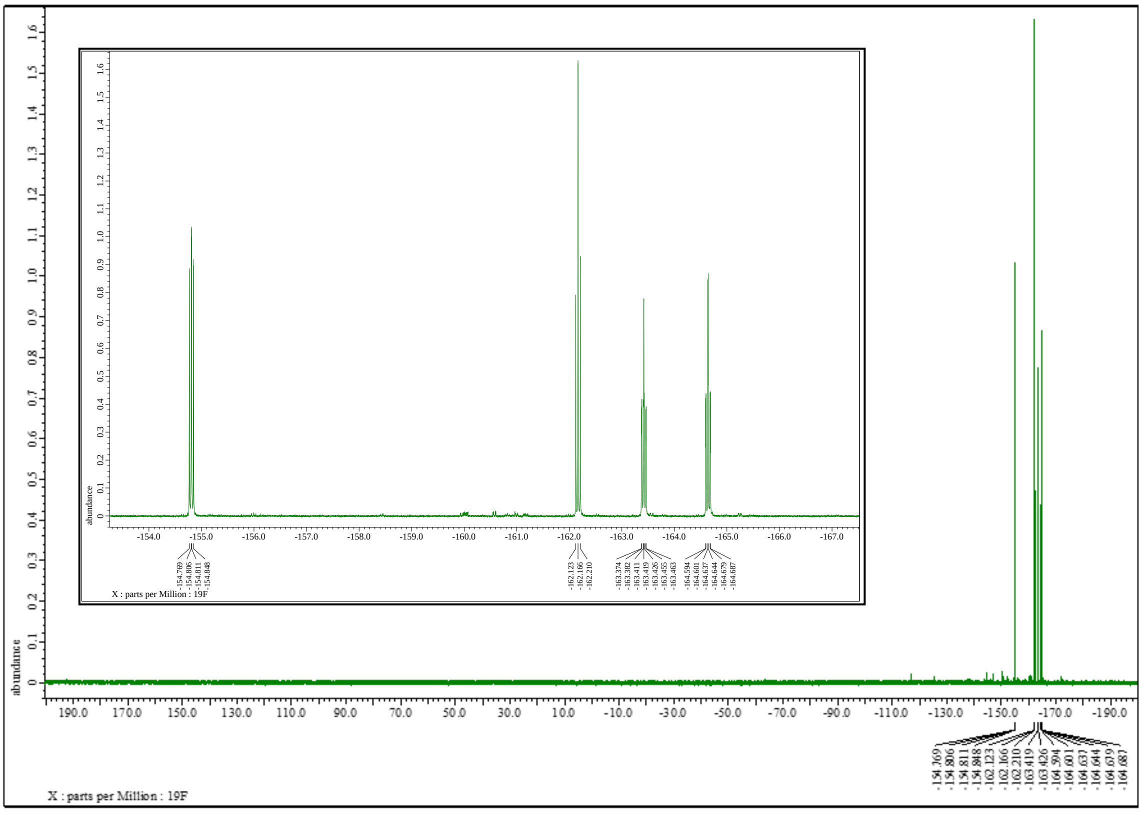

^{1}H NMR of spectrum of **1c** (500 MHz, CD_3OD)

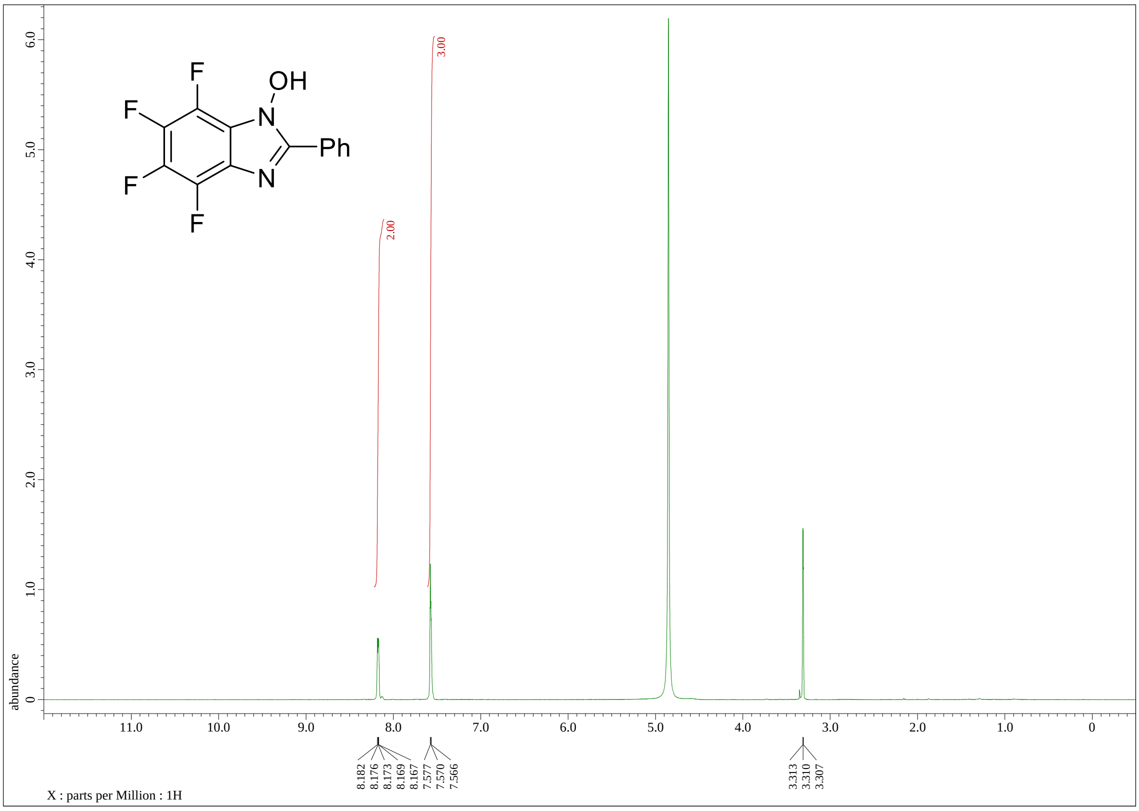

^{13}C NMR of spectrum of **1c** (125 MHz, CD_3OD)

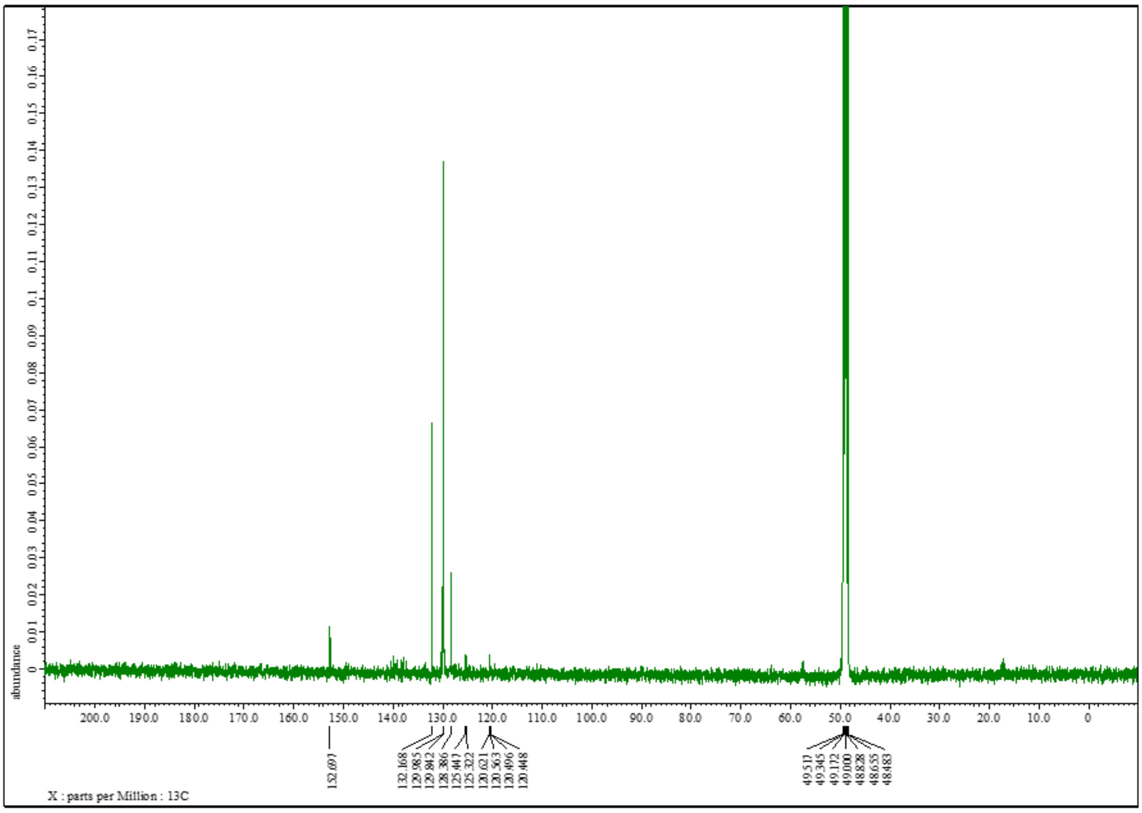

^{19}F NMR of spectrum of **1c** (470 MHz, CD_3OD)

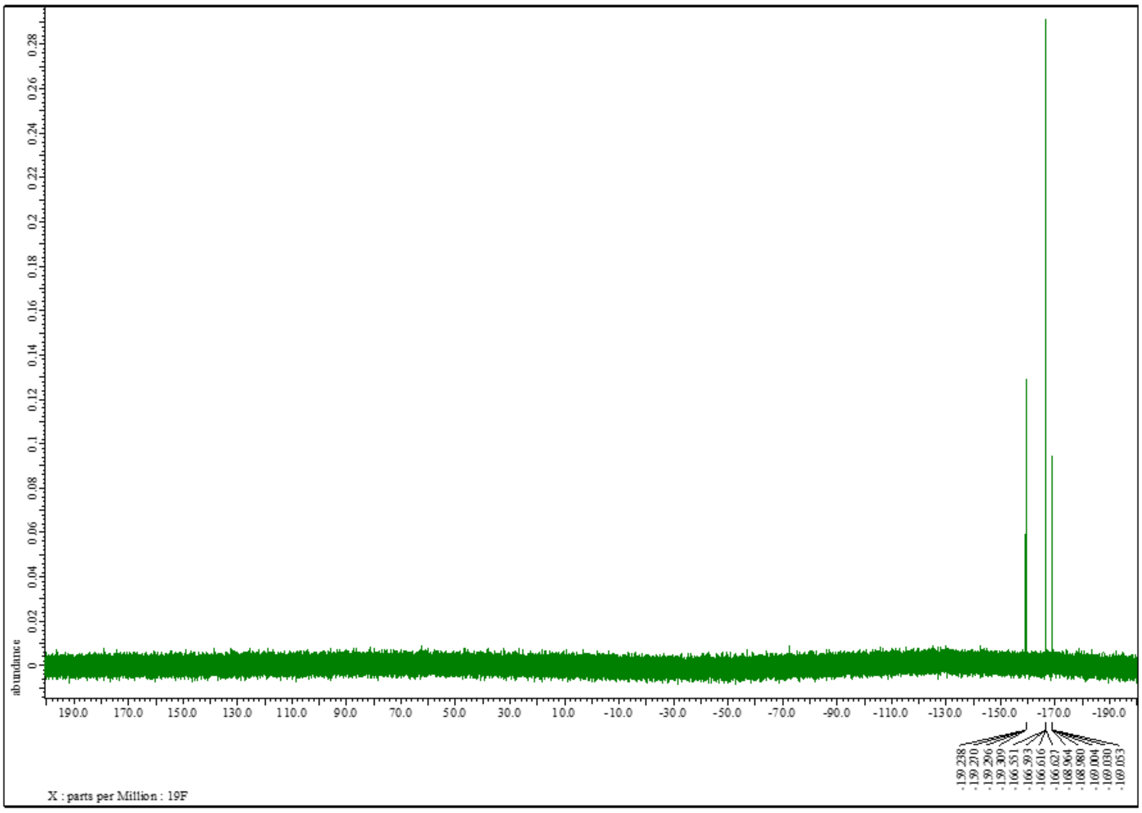

^1H NMR of spectrum of **S4** (500 MHz, $CDCl_3$)

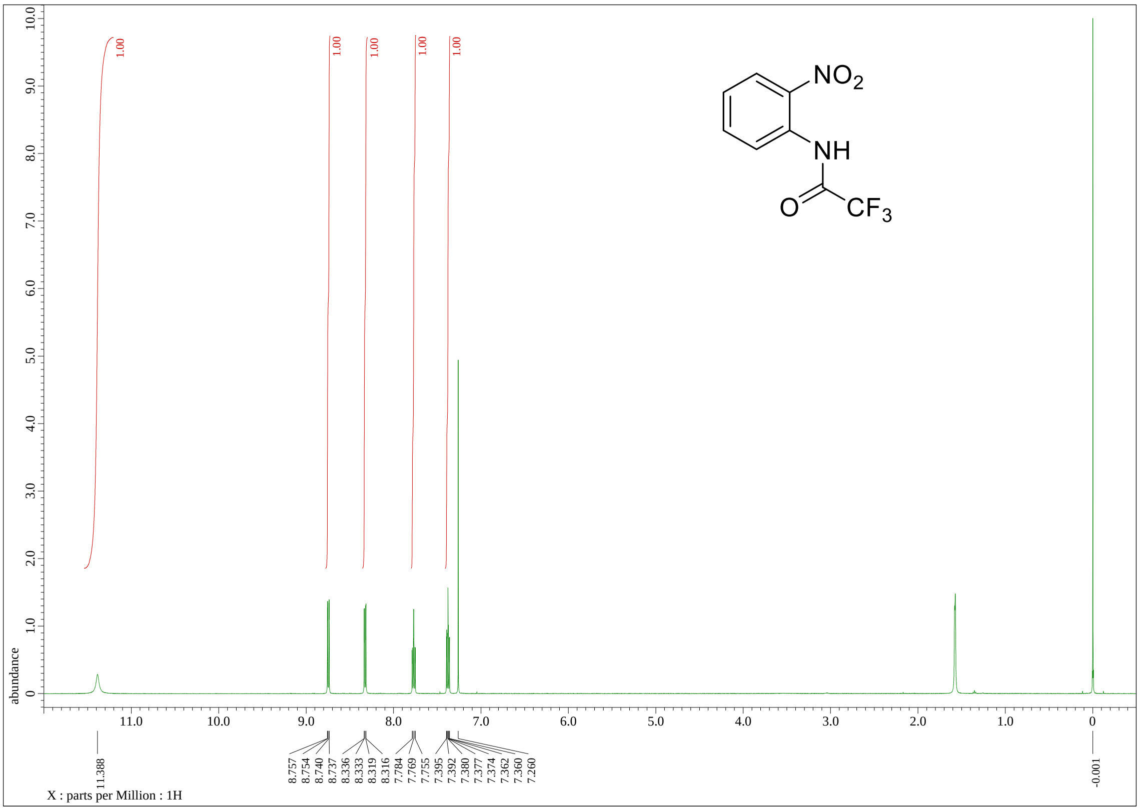

^{13}C NMR of spectrum of **S4** (125 MHz, $CDCl_3$)

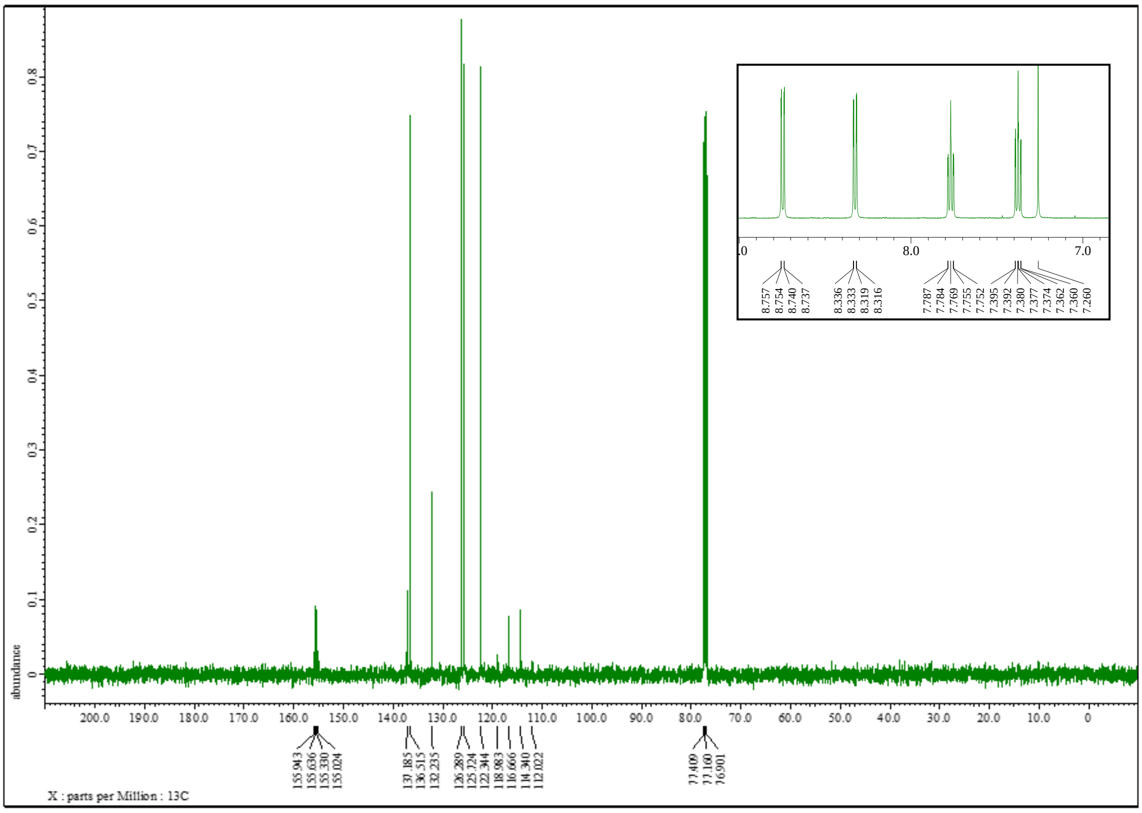

^{19}F NMR of spectrum of **S4** (470 MHz, $CDCl_3$)

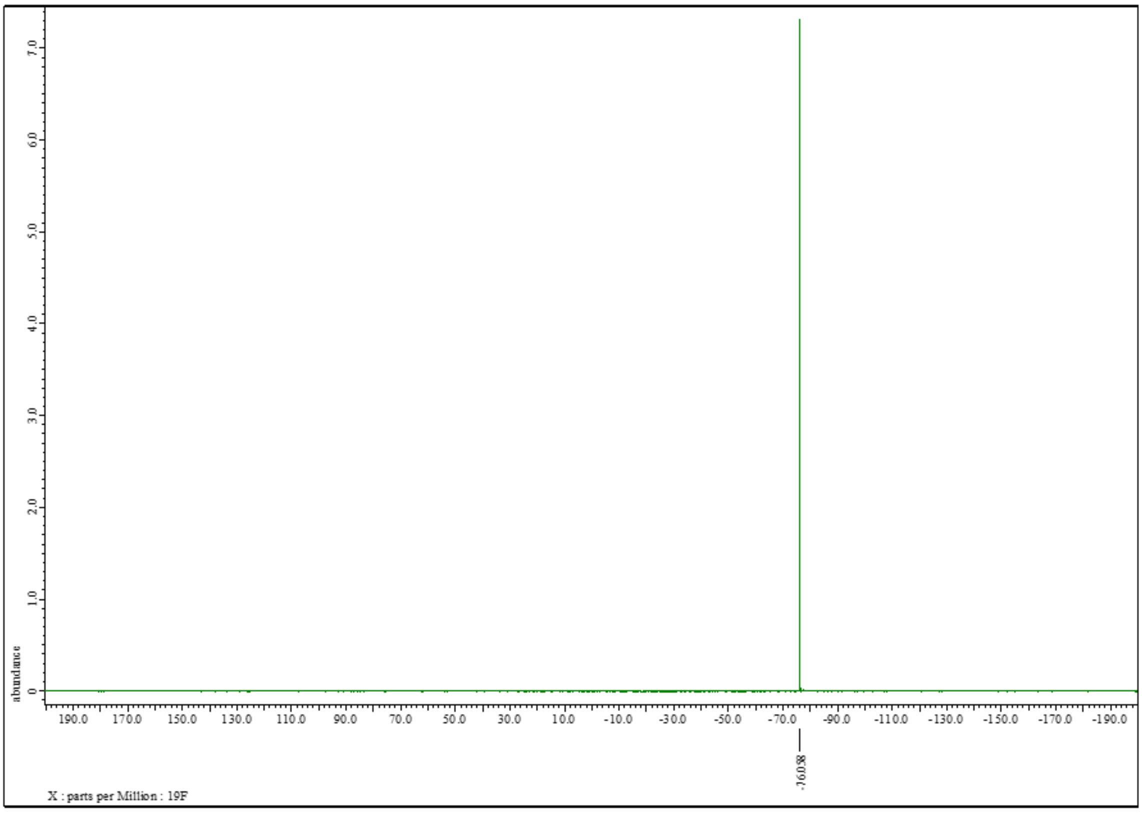

^{1}H NMR of spectrum of **1b** (500 MHz, CD_3OD)

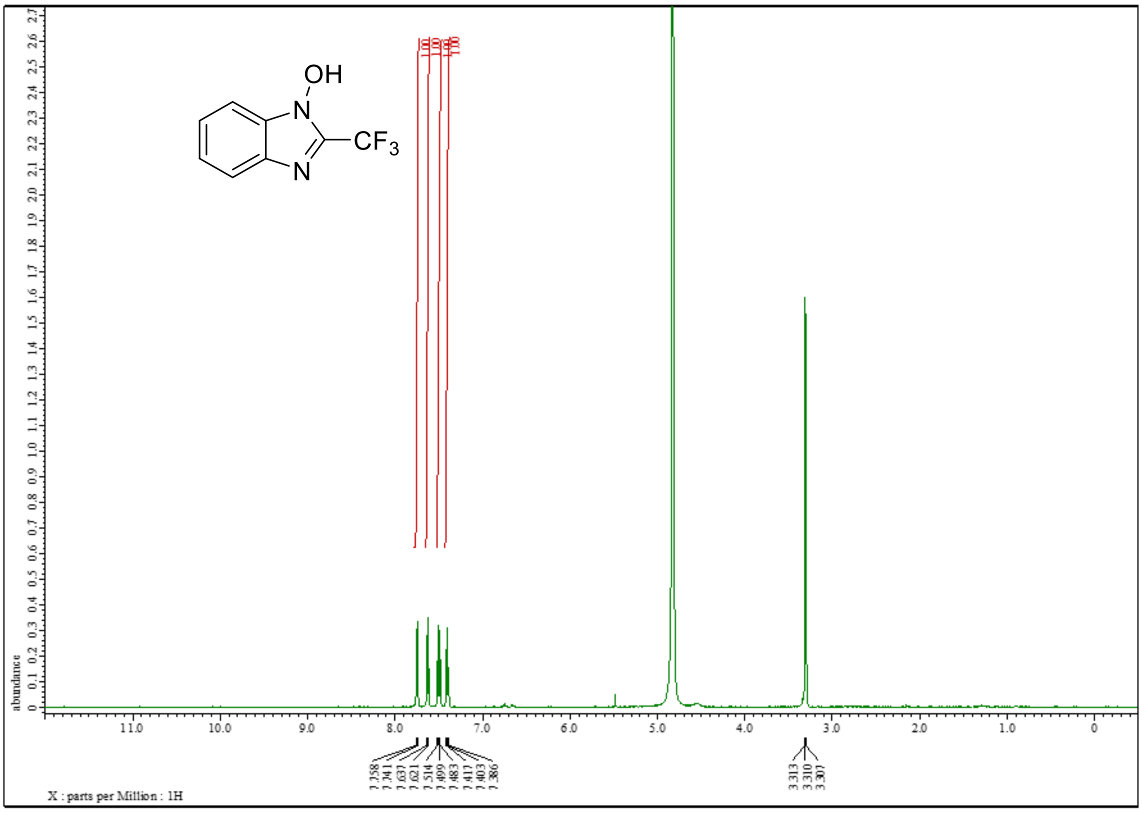

^{13}C NMR of spectrum of **1b** (125 MHz, CD_3OD)

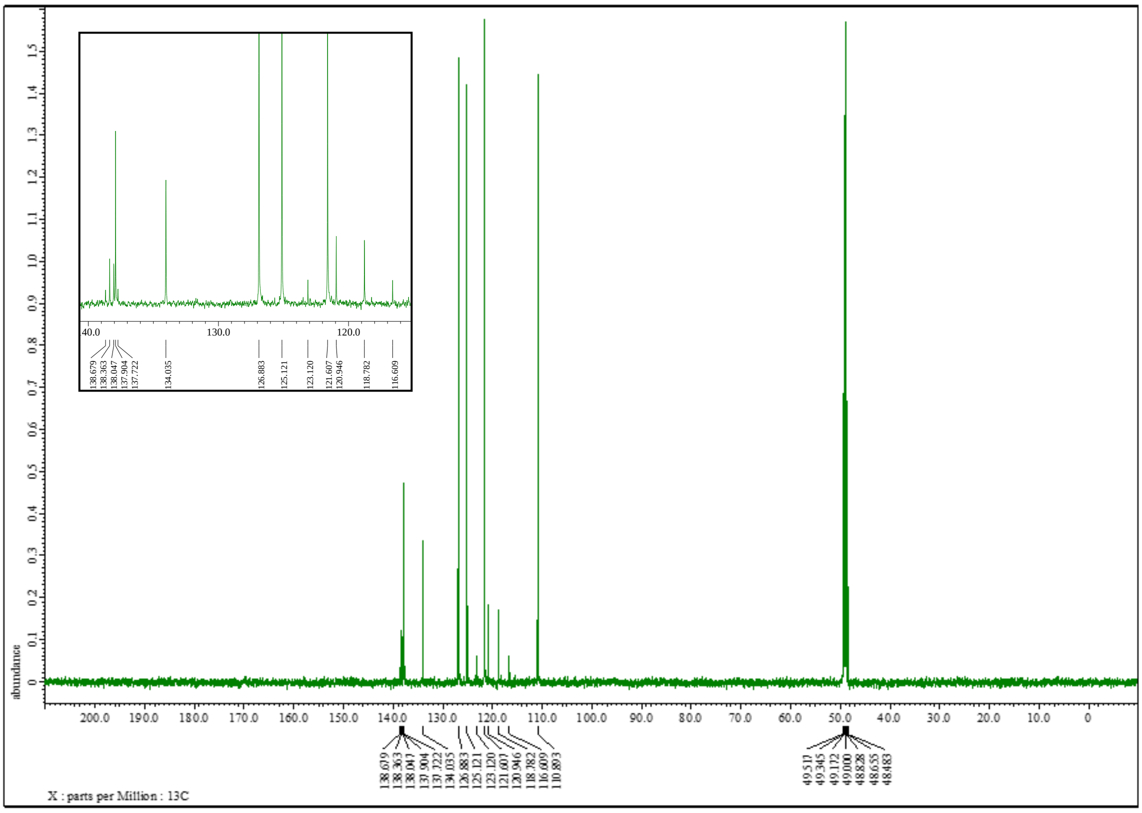

^{19}F NMR of spectrum of **1b** (470 MHz, CD_3OD)

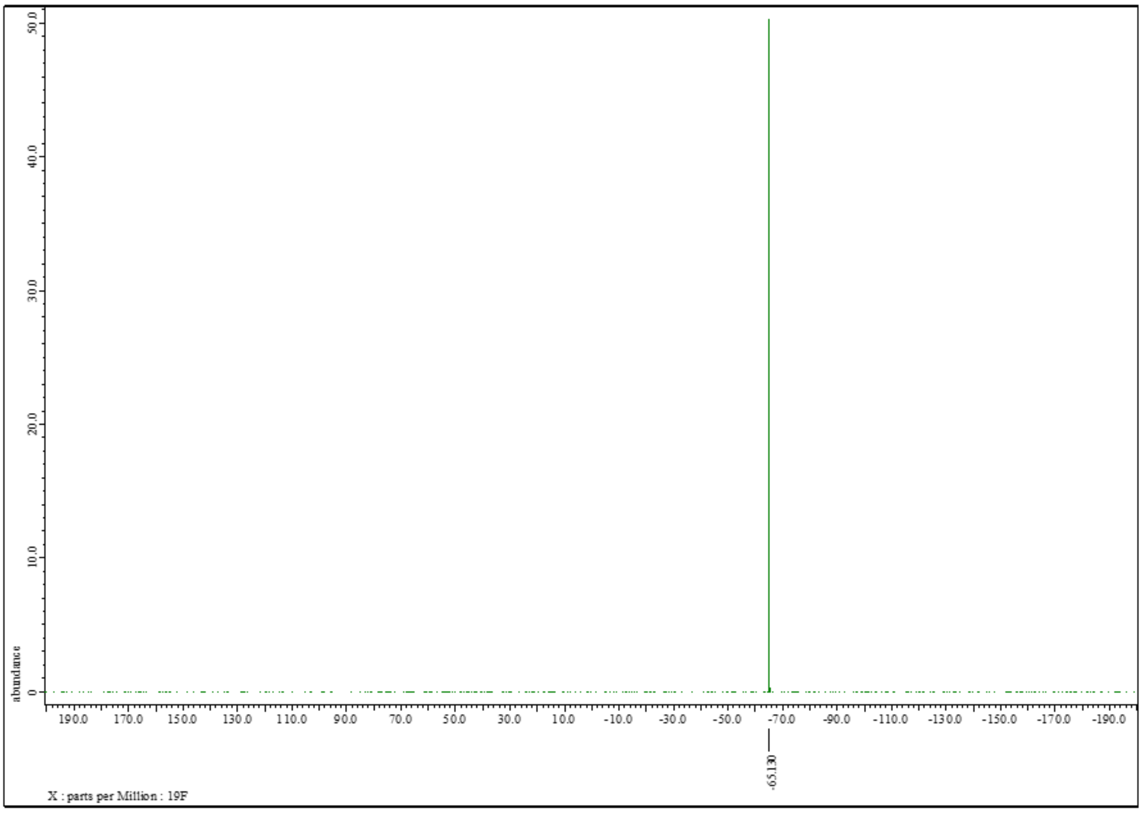

^{13}C NMR of spectrum of **S5** (500 MHz, $CDCl_3$)

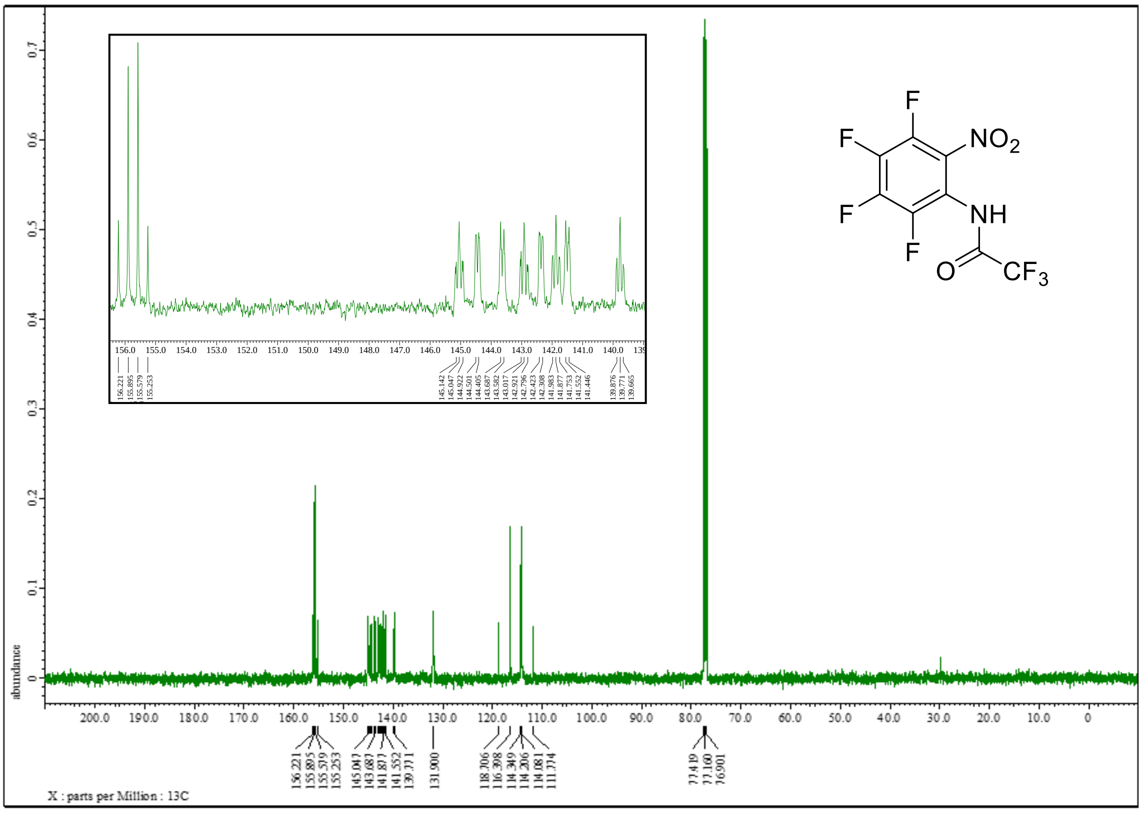

^{19}F NMR of spectrum of **S5** (470 MHz, $CDCl_3$)

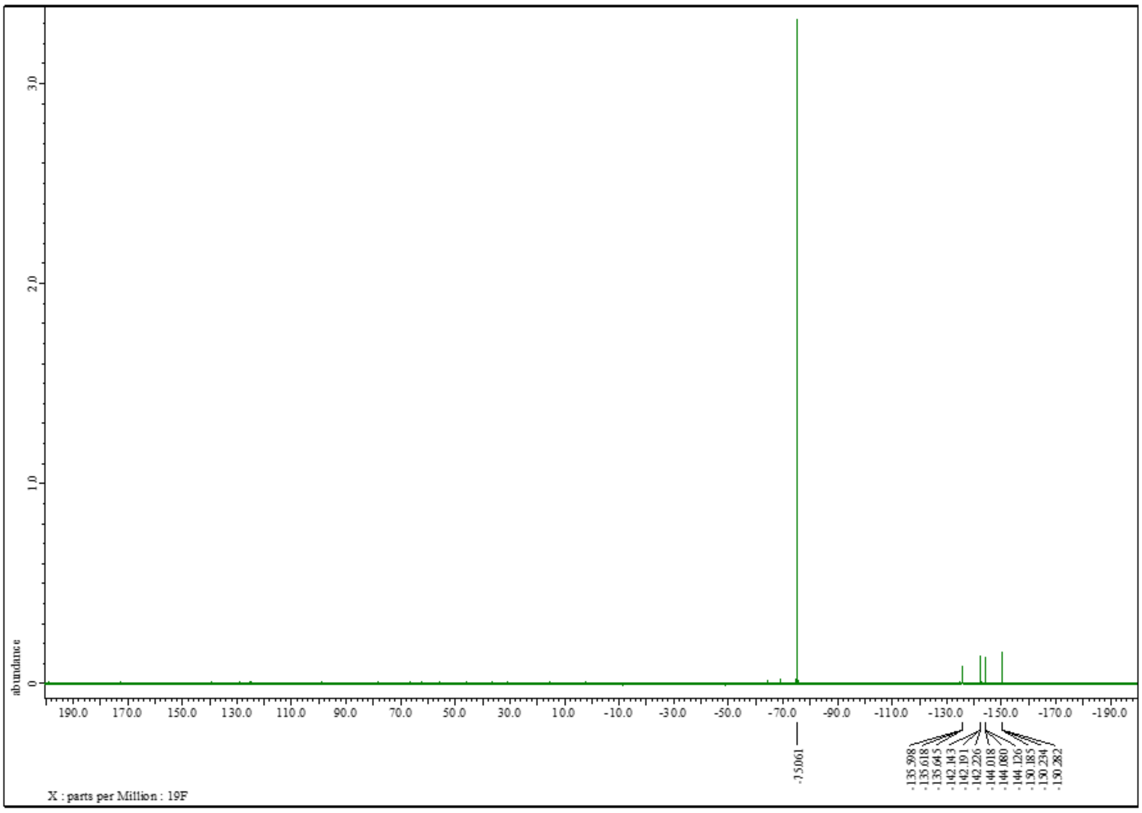

^{19}F NMR of spectrum of **1d** (470 MHz, $CDCl_3$)

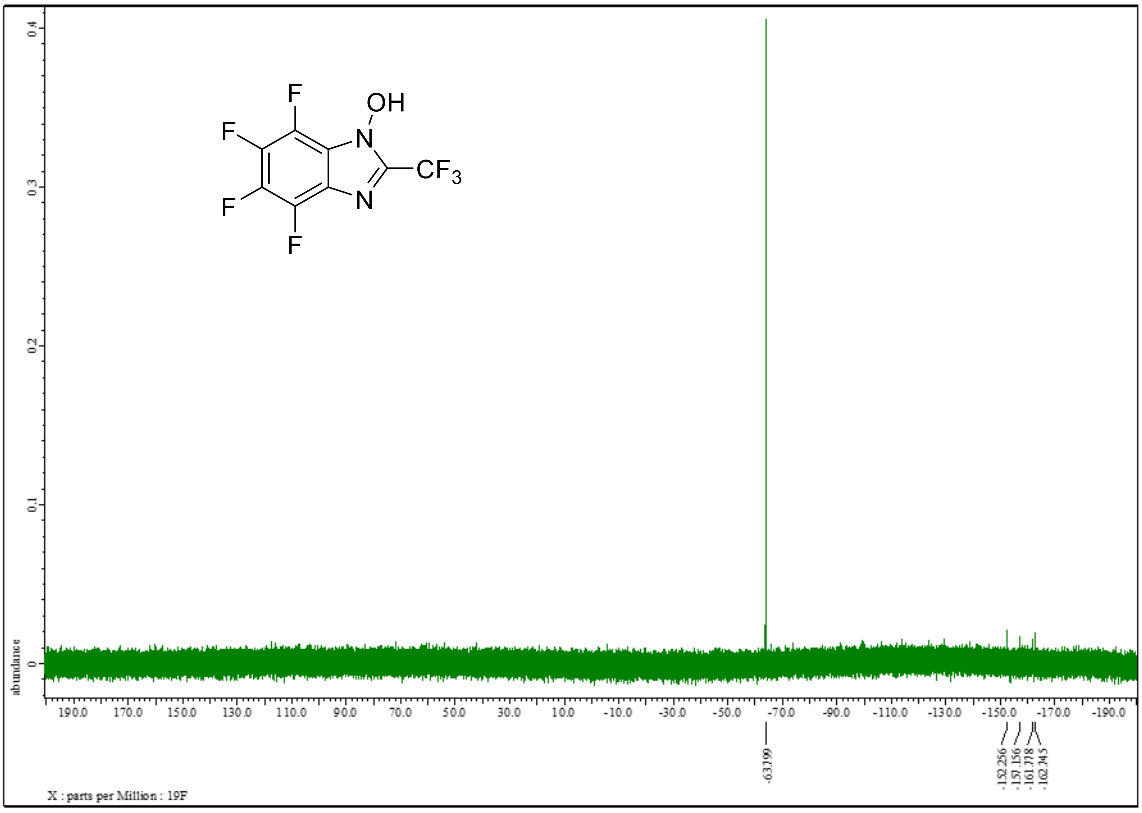

^{1}H NMR of spectrum of of **1h** (500 MHz, DMSO-d_6)

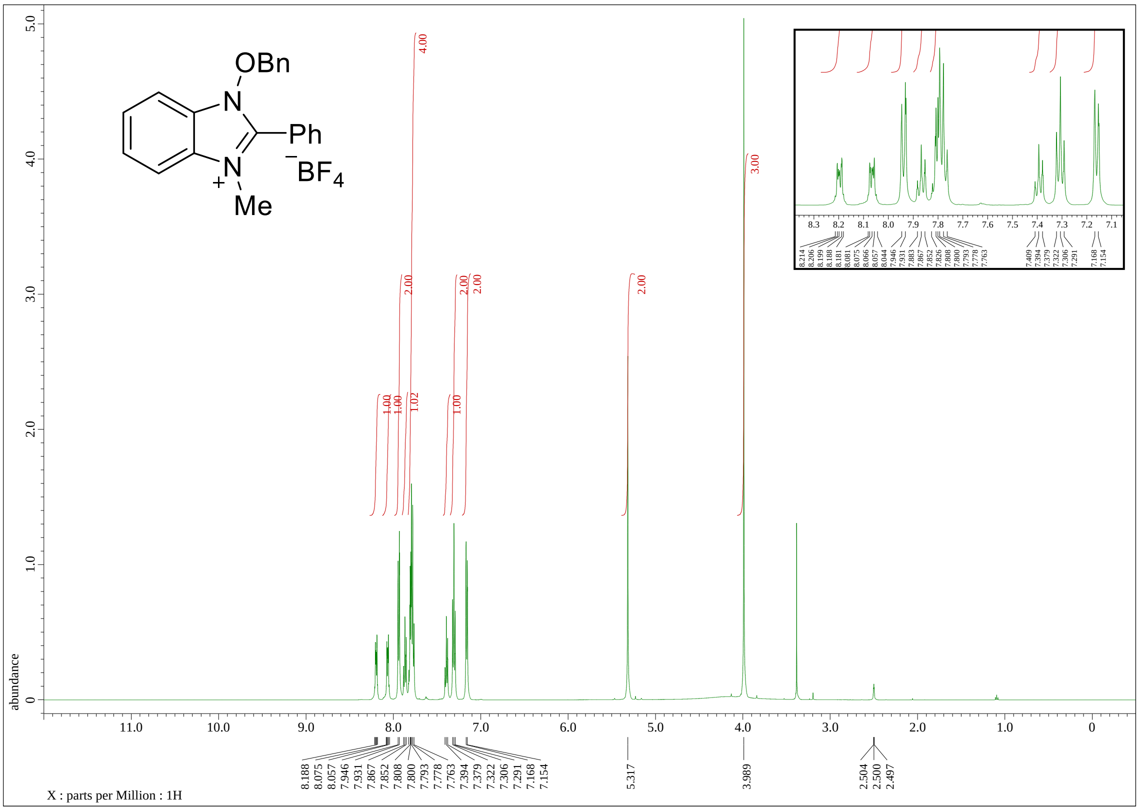

^{13}C NMR of spectrum of **1h** (125 MHz, DMSO-d_6)

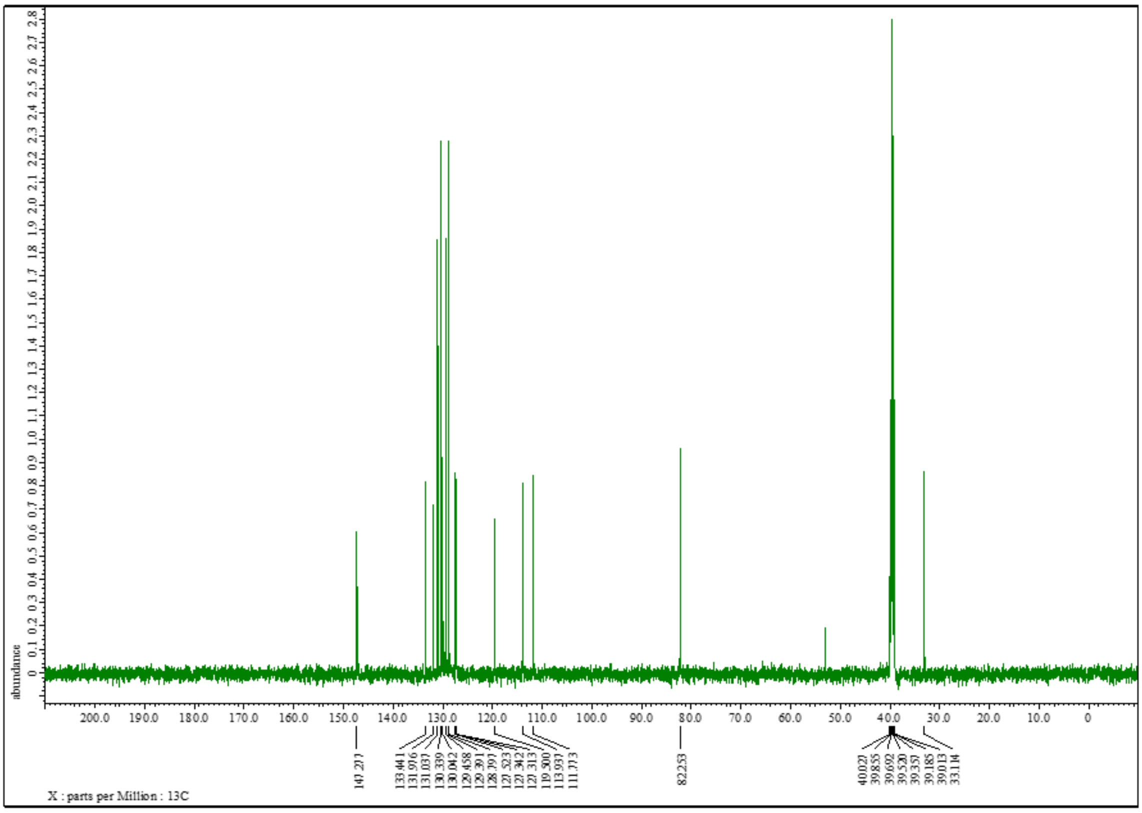

^{19}F NMR of spectrum of **1h** (125 MHz, DMSO-d_6)

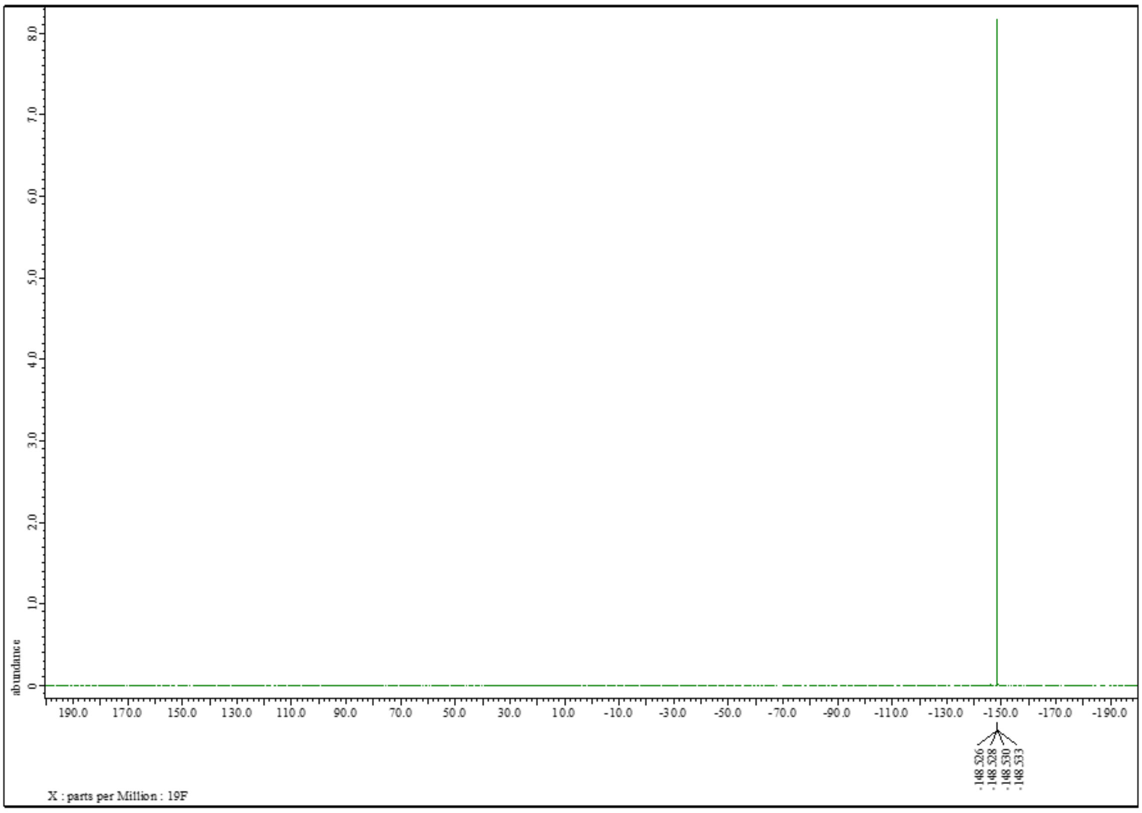

^{1}H NMR of spectrum of **1e** (500 MHz, DMSO-d_6)

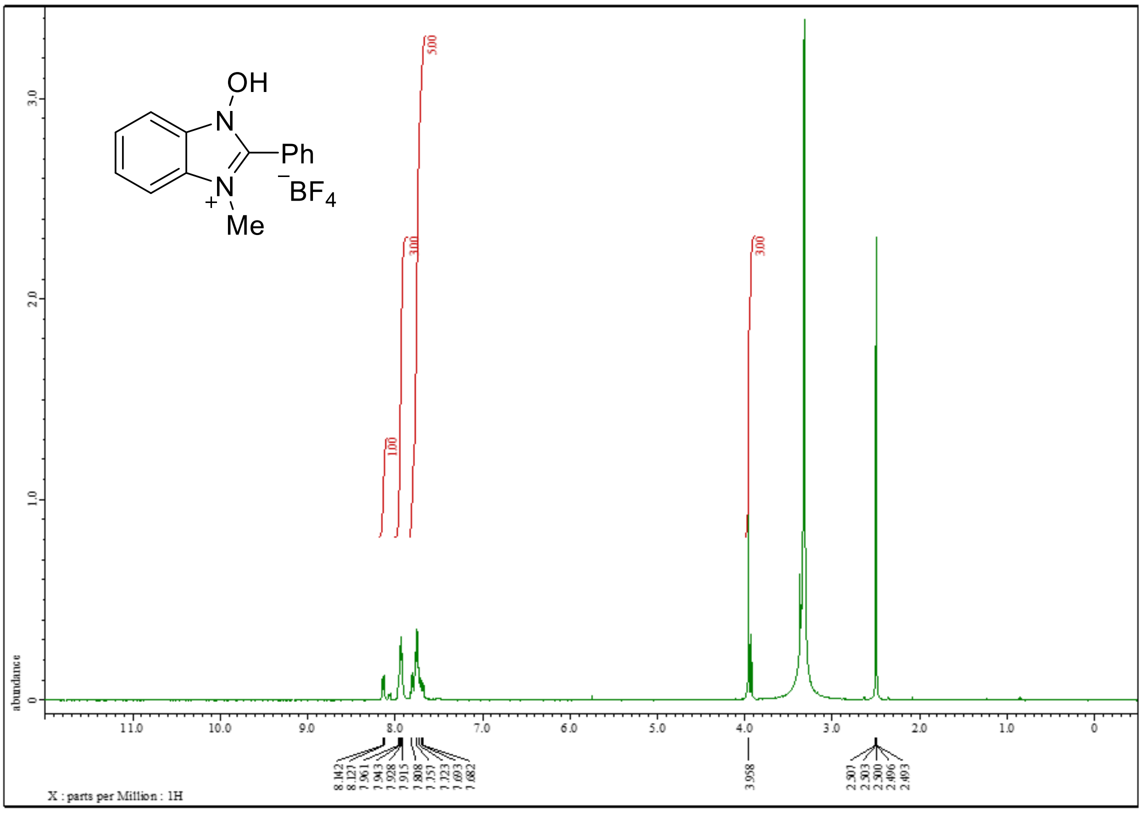

^{13}C NMR of spectrum of **1e** (125 MHz, DMSO-d_6)

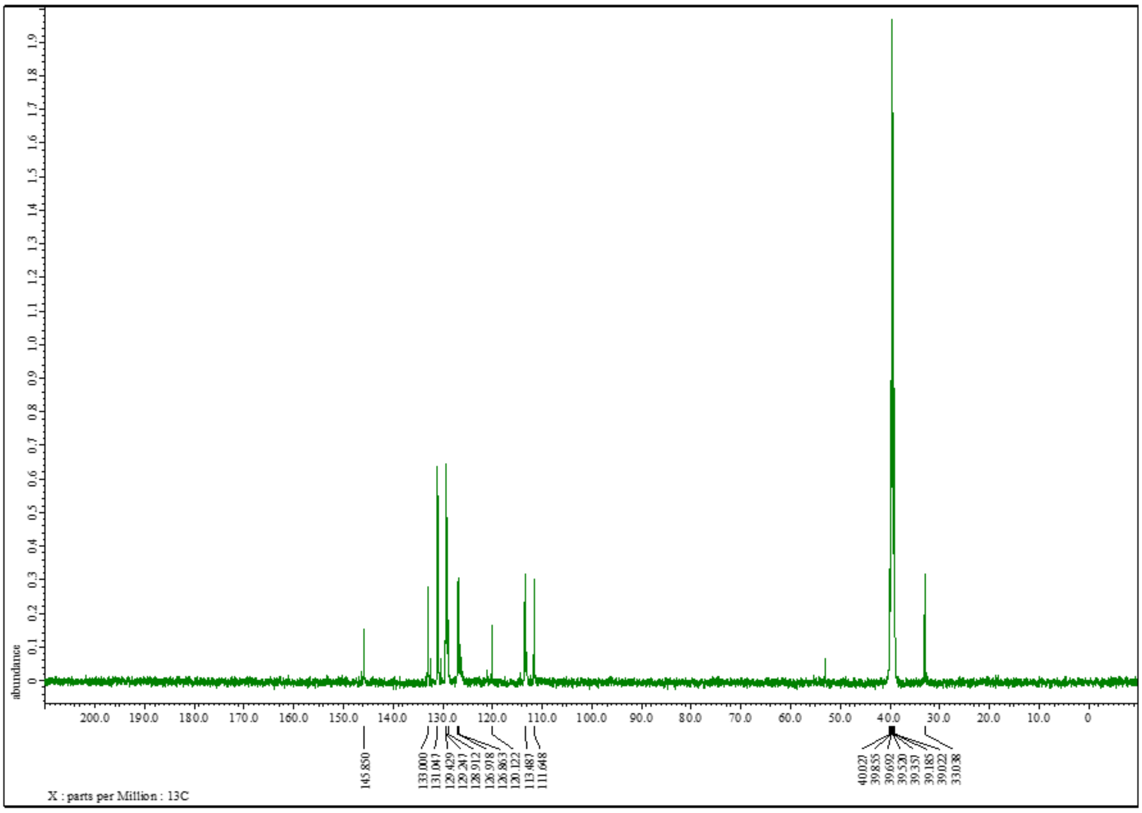

^{19}F NMR of spectrum of **1e** (470 MHz, DMSO-d_6)

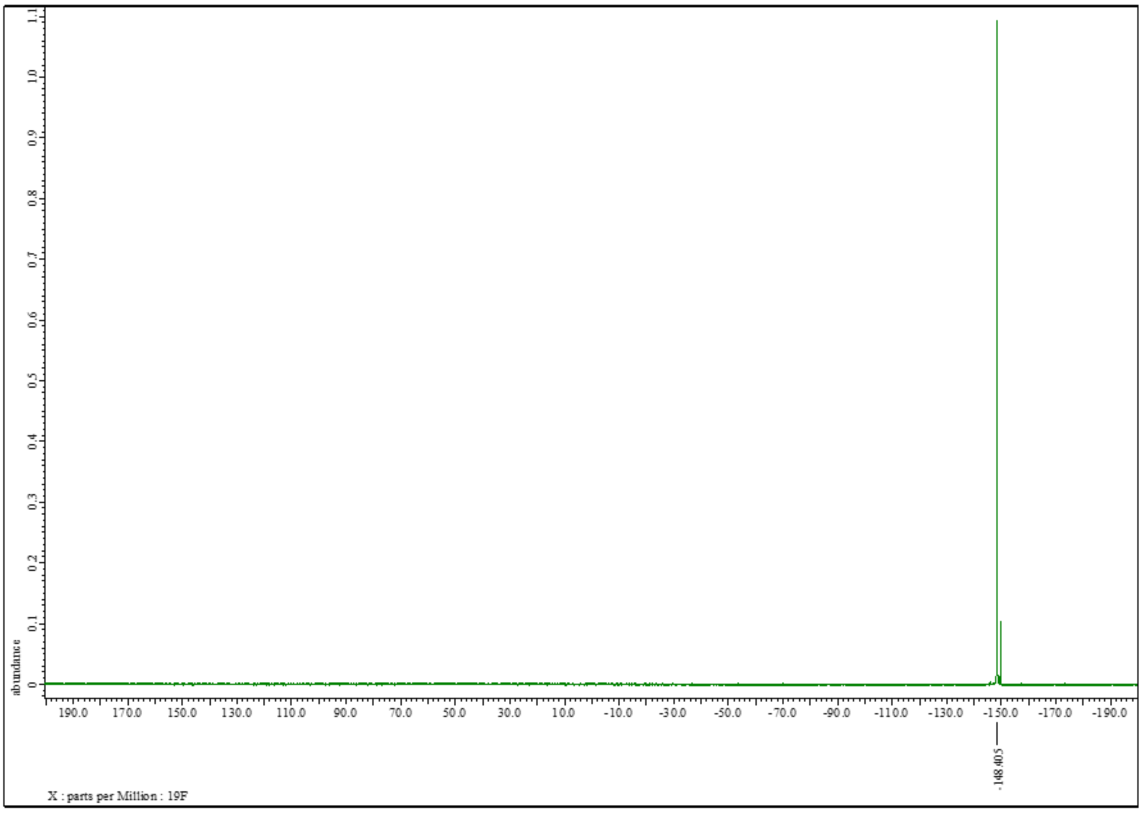

^{1}H NMR of spectrum of **S6** (500 MHz, $CDCl_3$)

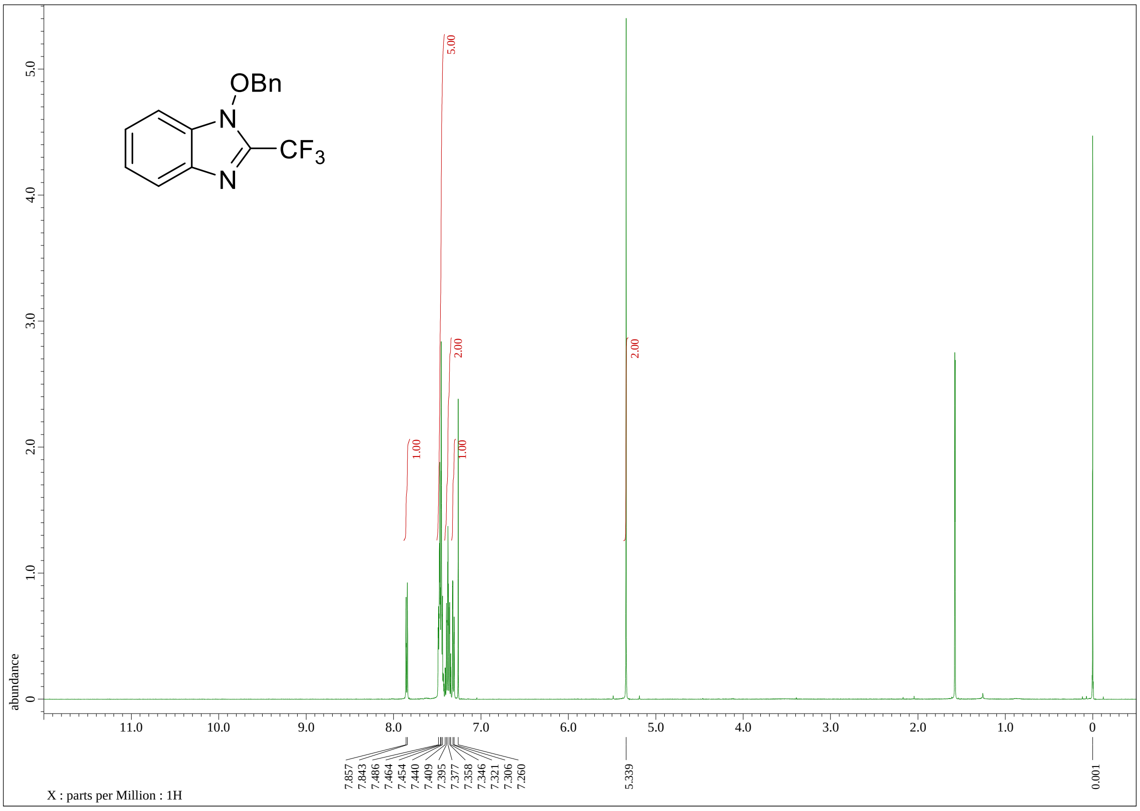

^{13}C NMR of spectrum of **S6** (125 MHz, $CDCl_3$)

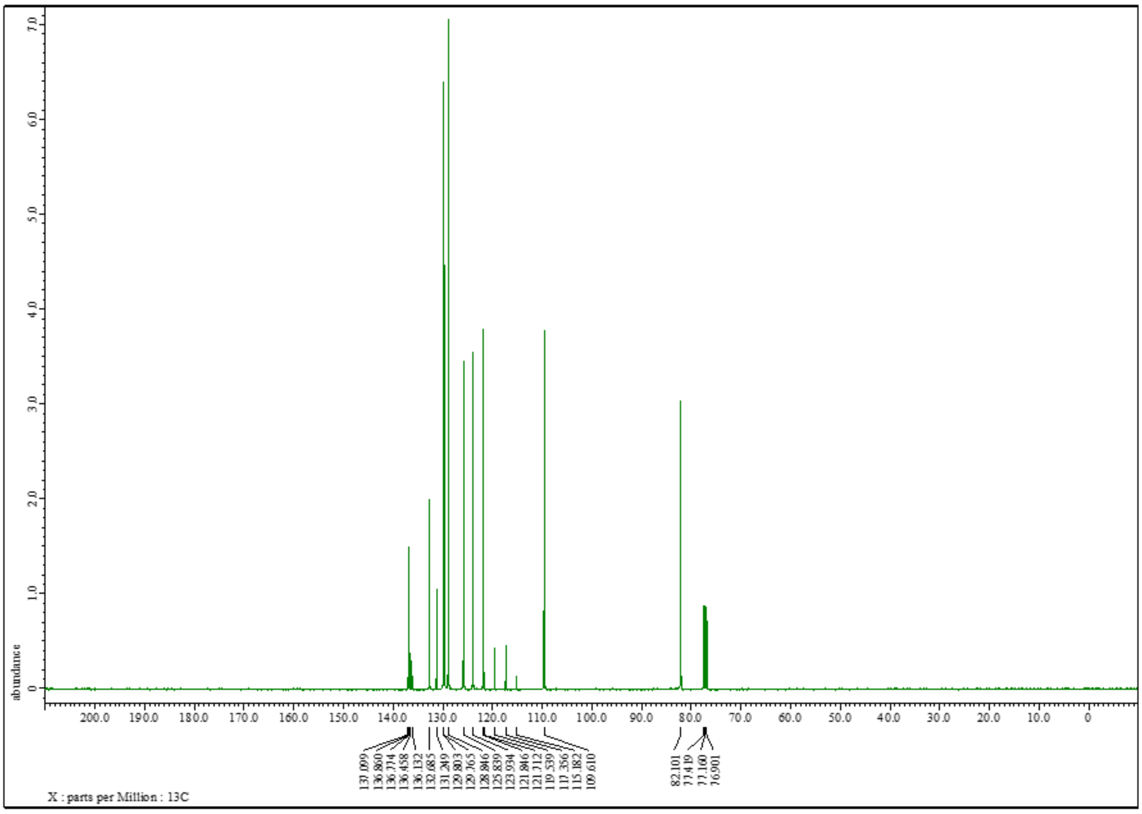

^{19}F NMR of spectrum of **S6** (470 MHz, $CDCl_3$)

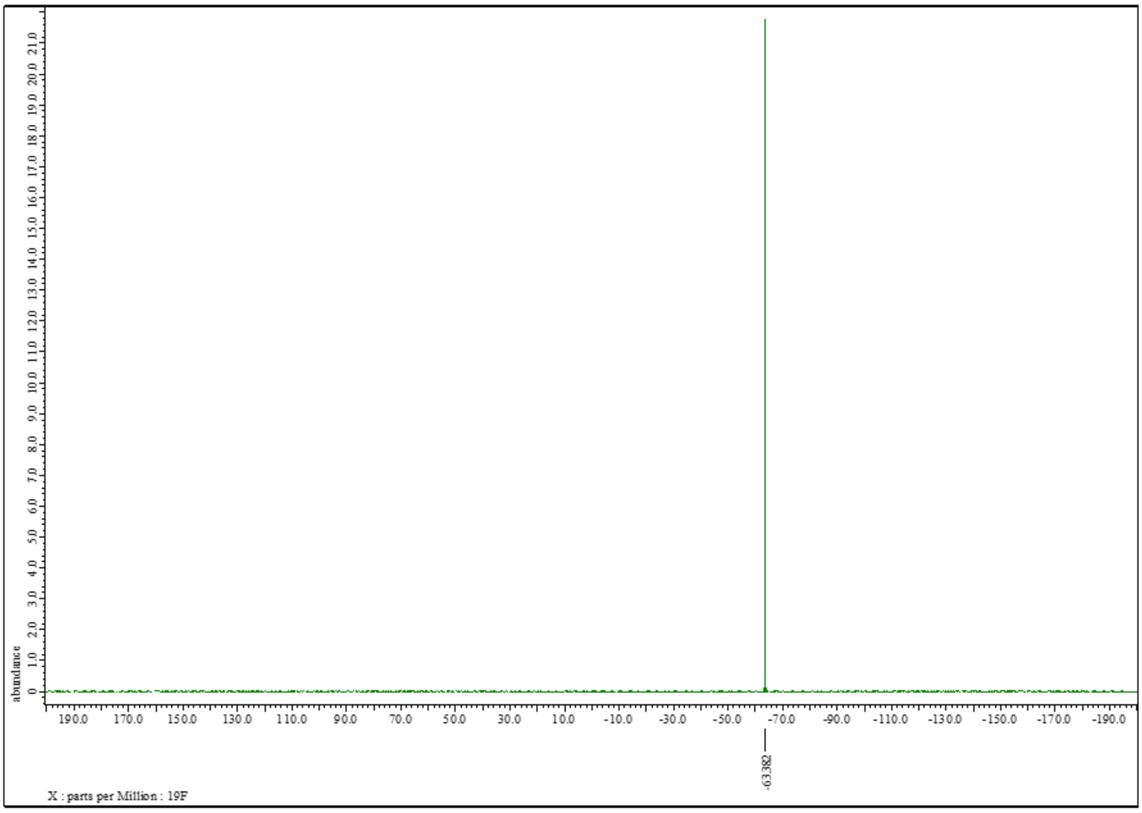

^{1}H NMR of spectrum of **S7** (500 MHz, CD_3CN)

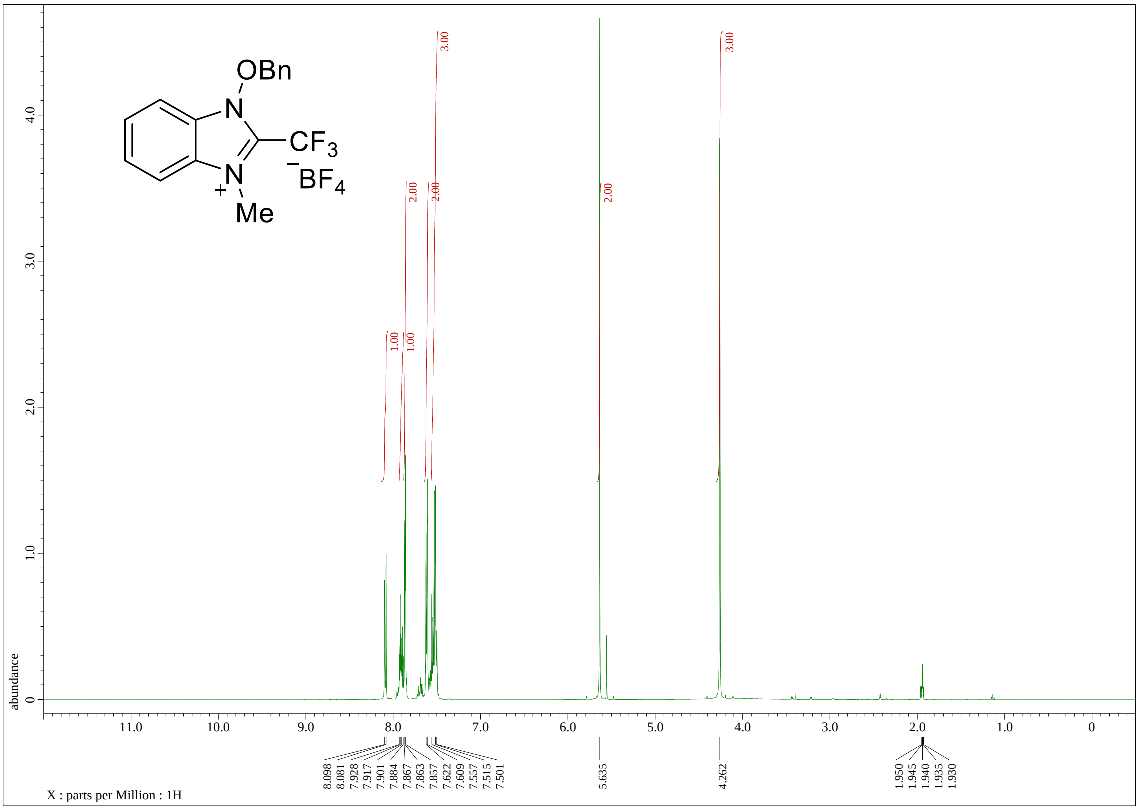

^{13}C NMR of spectrum of **S7** (125 MHz, CD_3CN)

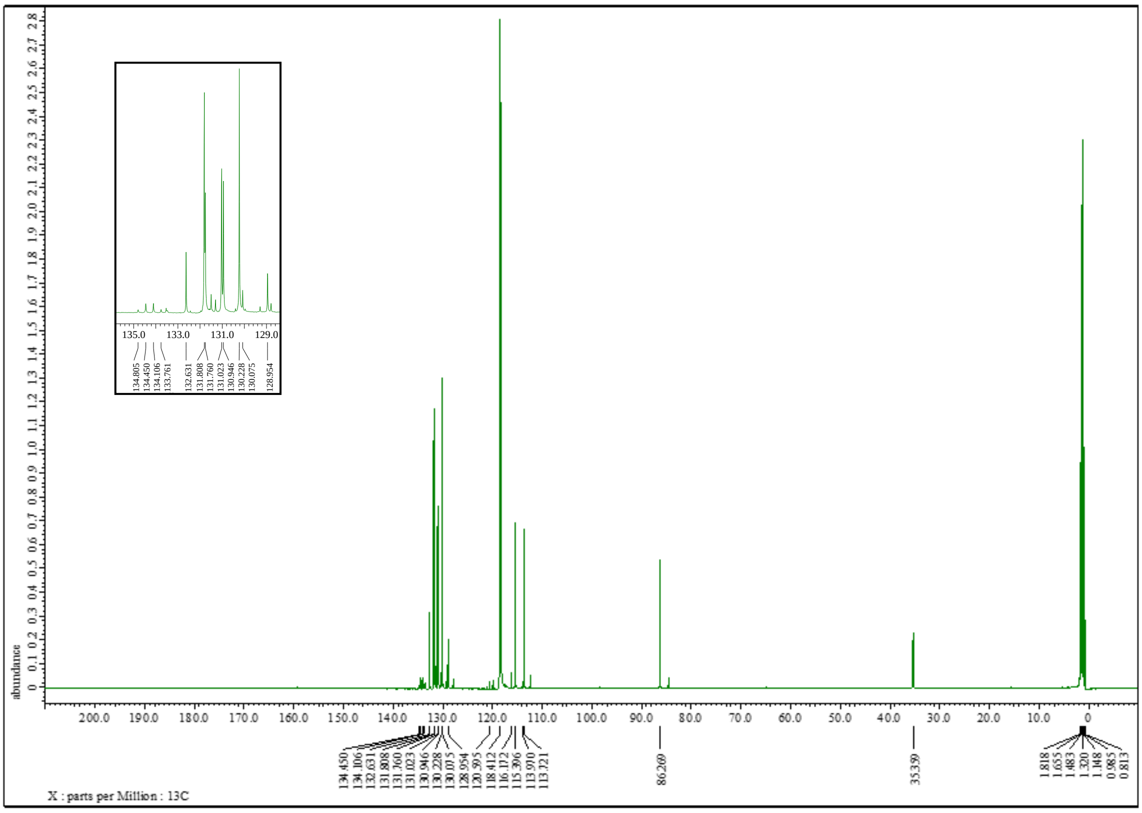

^{19}F NMR of spectrum of **S7** (470 MHz, CD_3CN)

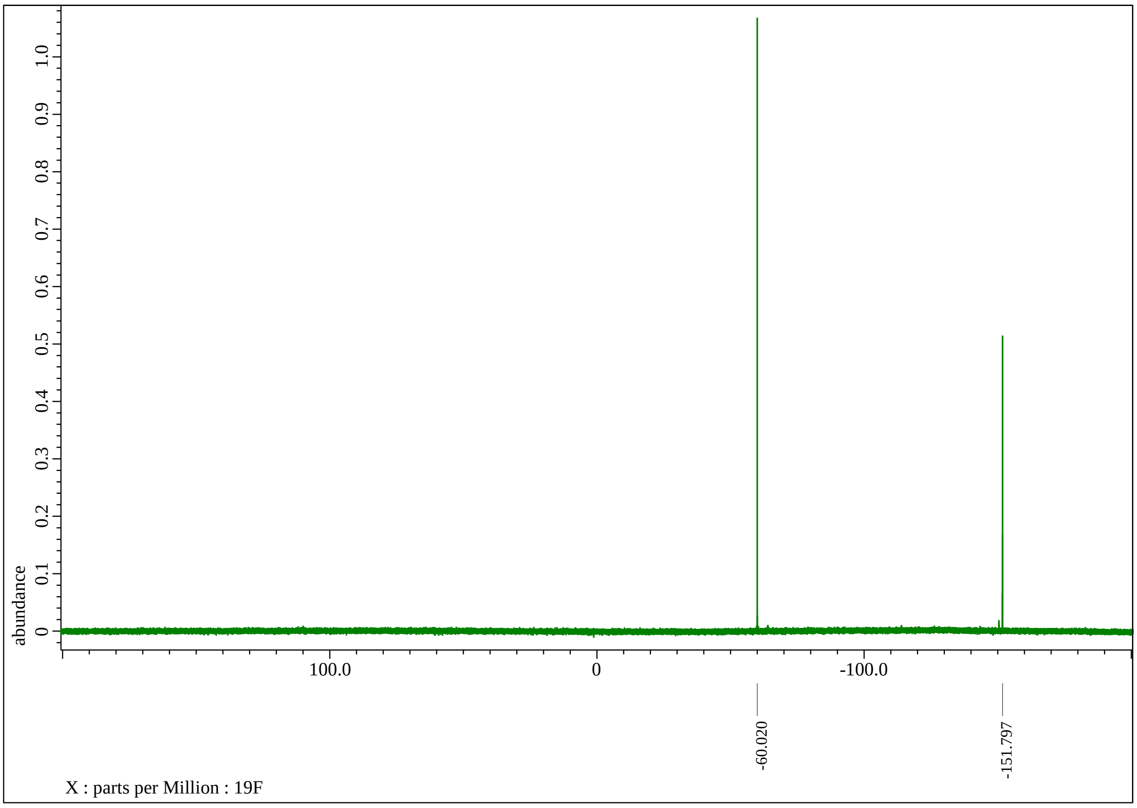

^1H NMR of spectrum **1f** (500 MHz, CD_3OD)

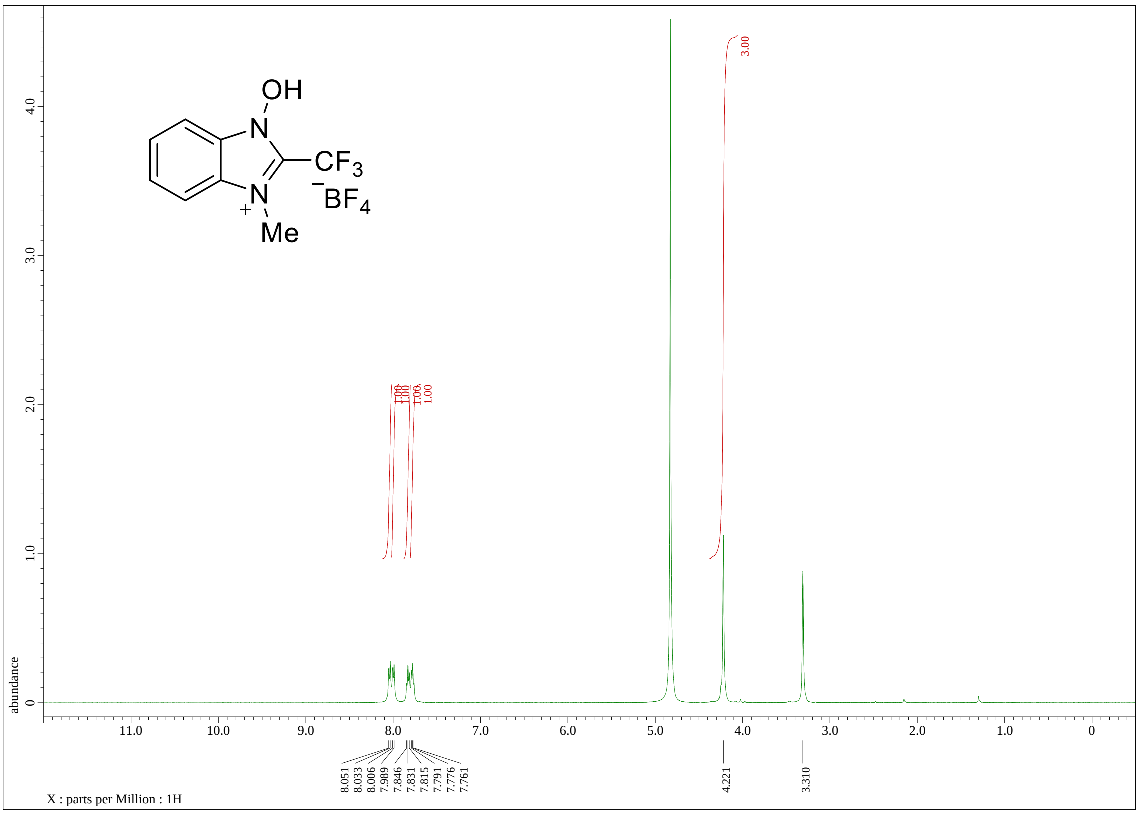

^{13}C NMR of spectrum of **1f** (125 MHz, CD_3OD)

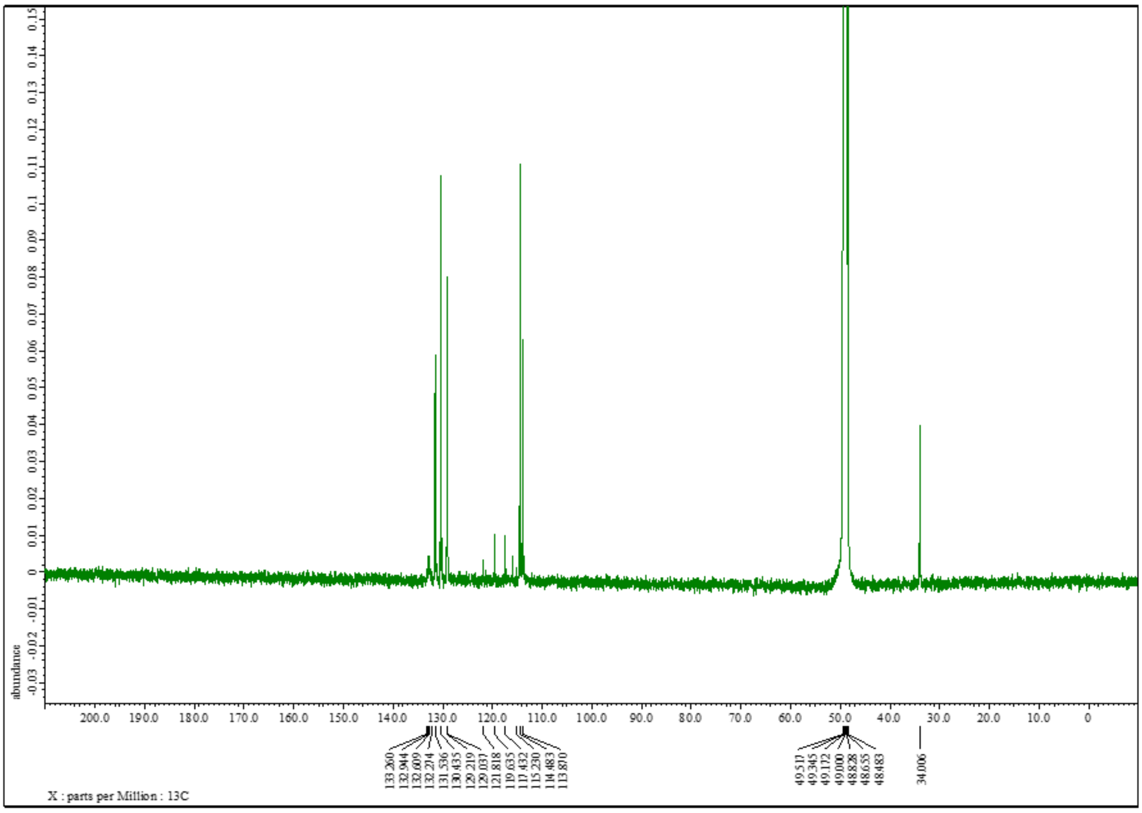

^{19}F NMR of spectrum of **1f** (470 MHz, CD_3OD)

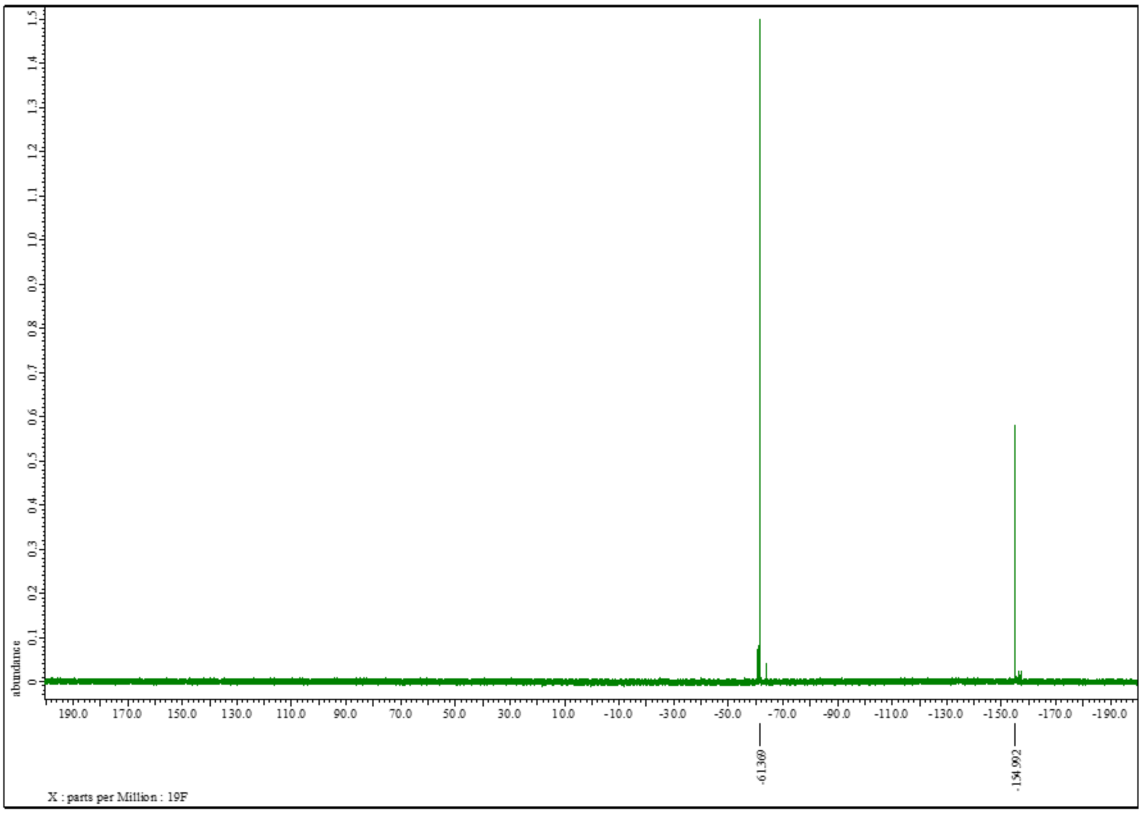

^{1}H NMR of spectrum of **S8** (500 MHz, DMSO-d_6)

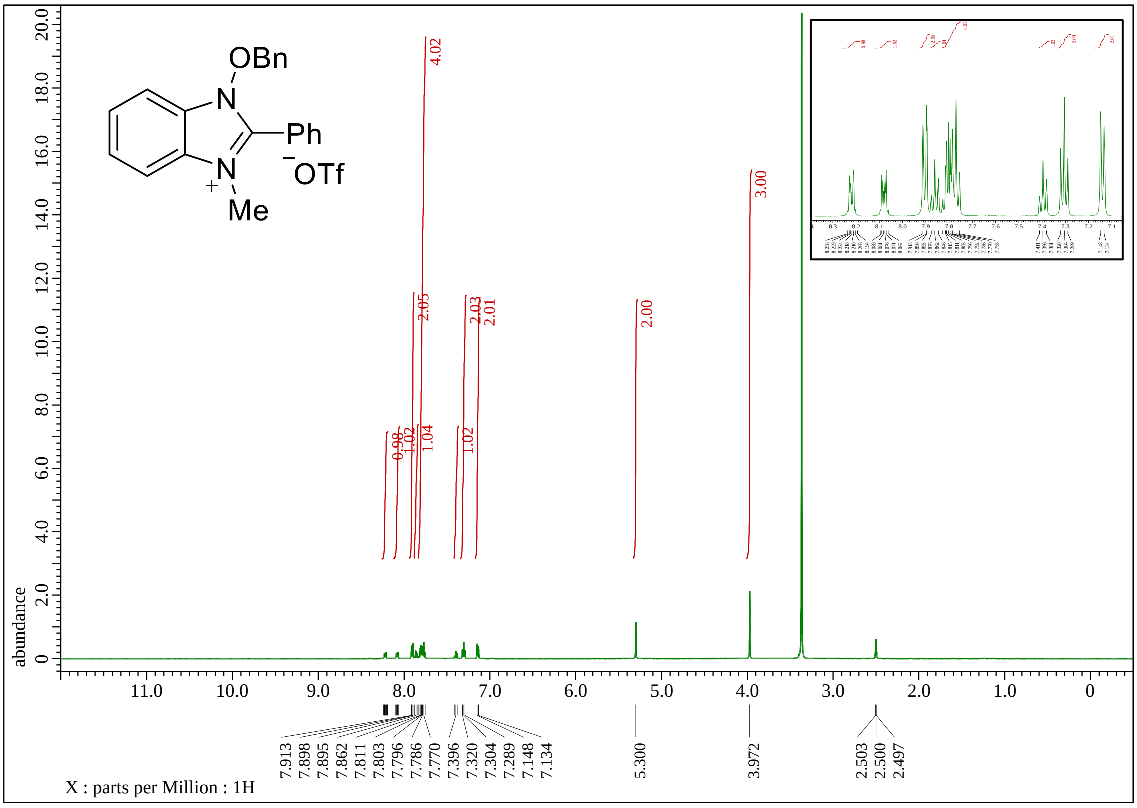

^{13}C NMR of spectrum of **S8** (125 MHz, DMSO-d_6)

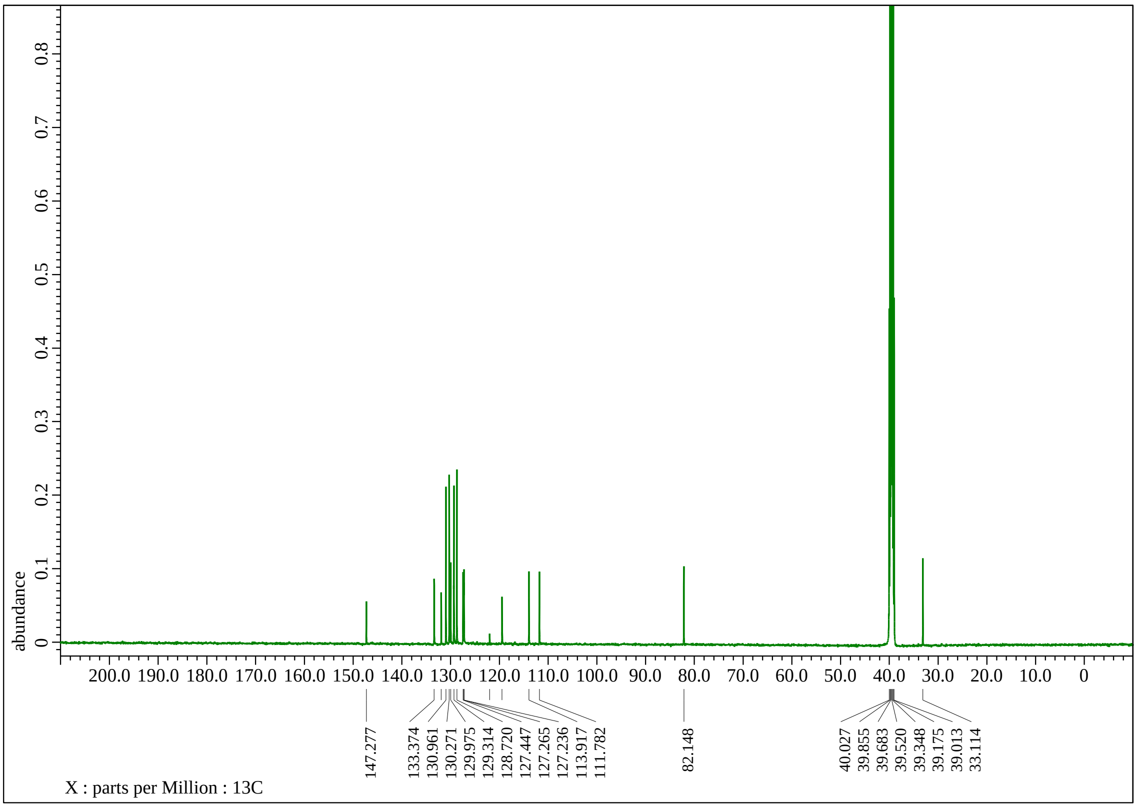

^{19}F NMR of spectrum of **S8** (125 MHz, DMSO-d_6)

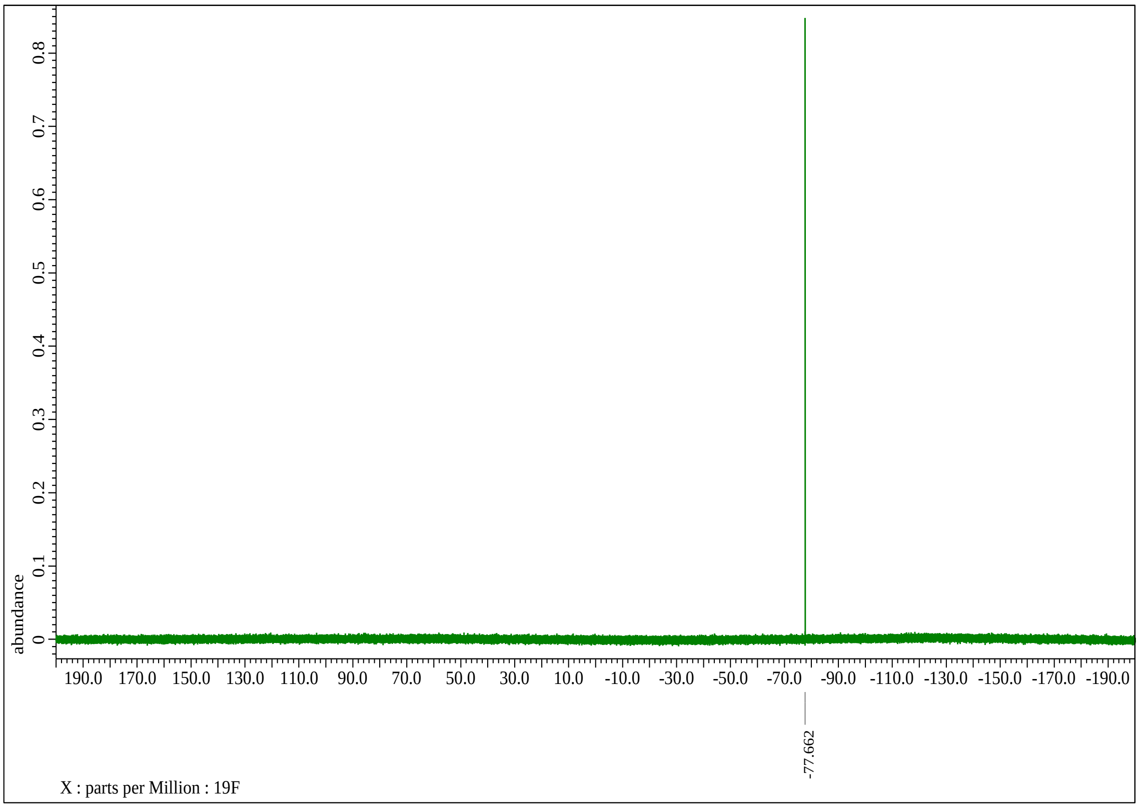

^{1}H NMR of spectrum of **1g** (500 MHz, DMSO-d_6)

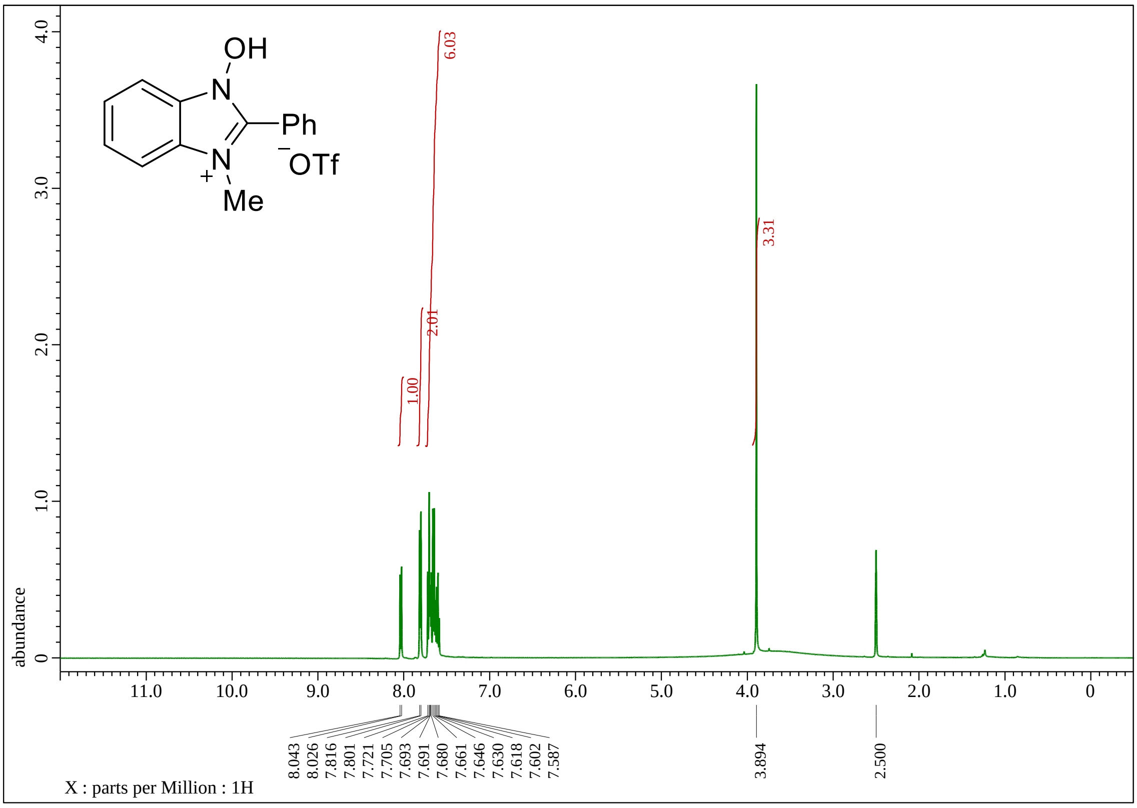

^{13}C NMR of spectrum of **1g** (125 MHz, DMSO-d_6)

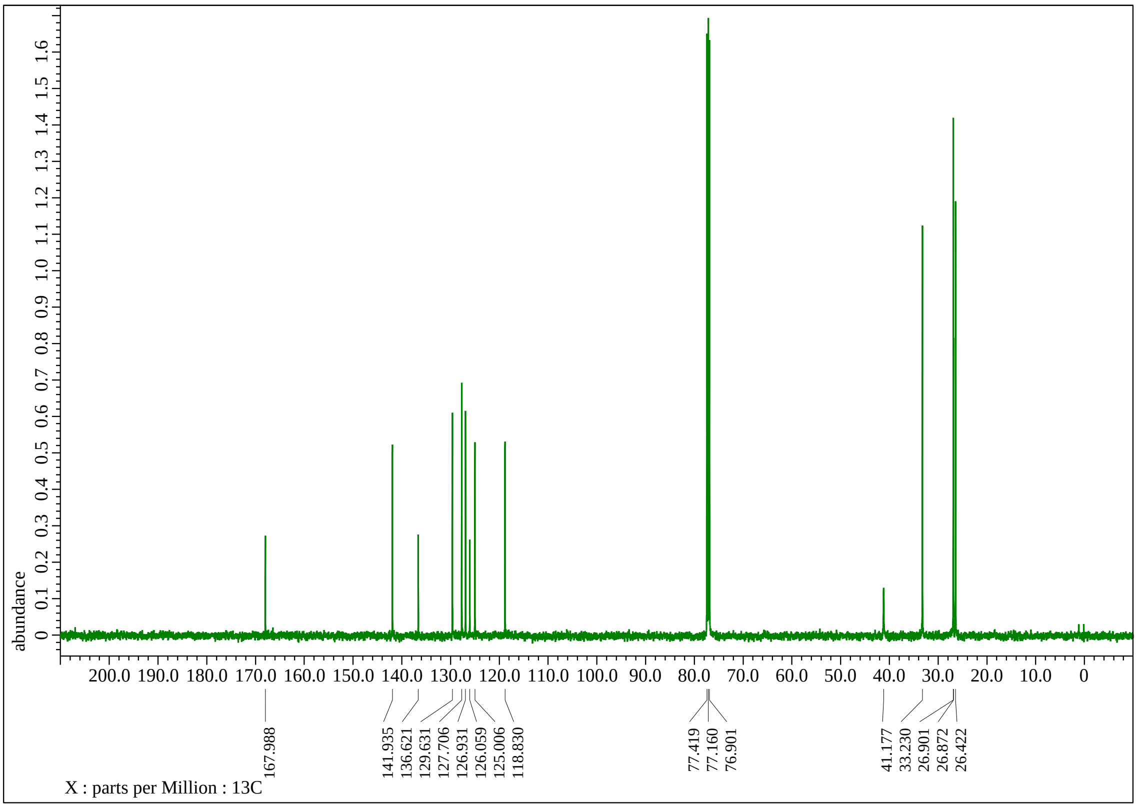

^{19}F NMR of spectrum of **1g** (470 MHz, DMSO-d_6)

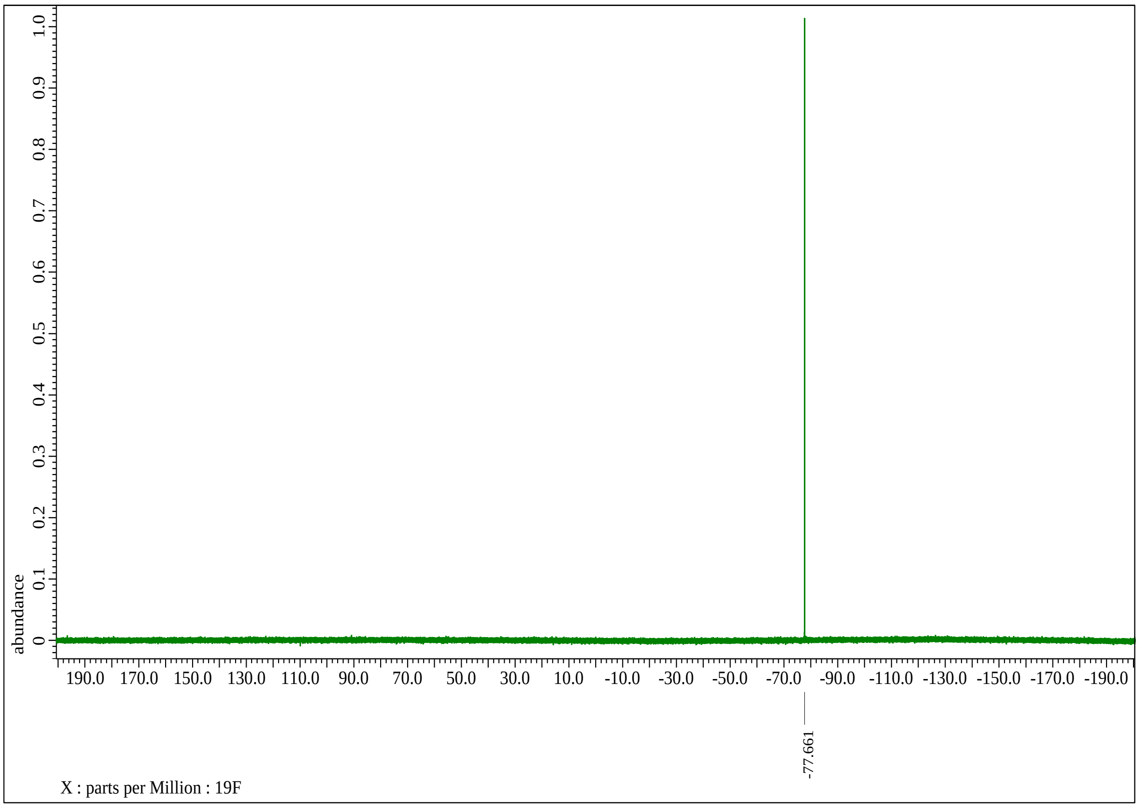

^{1}H NMR of spectrum of **4** (500 MHz, $CDCl_3$)

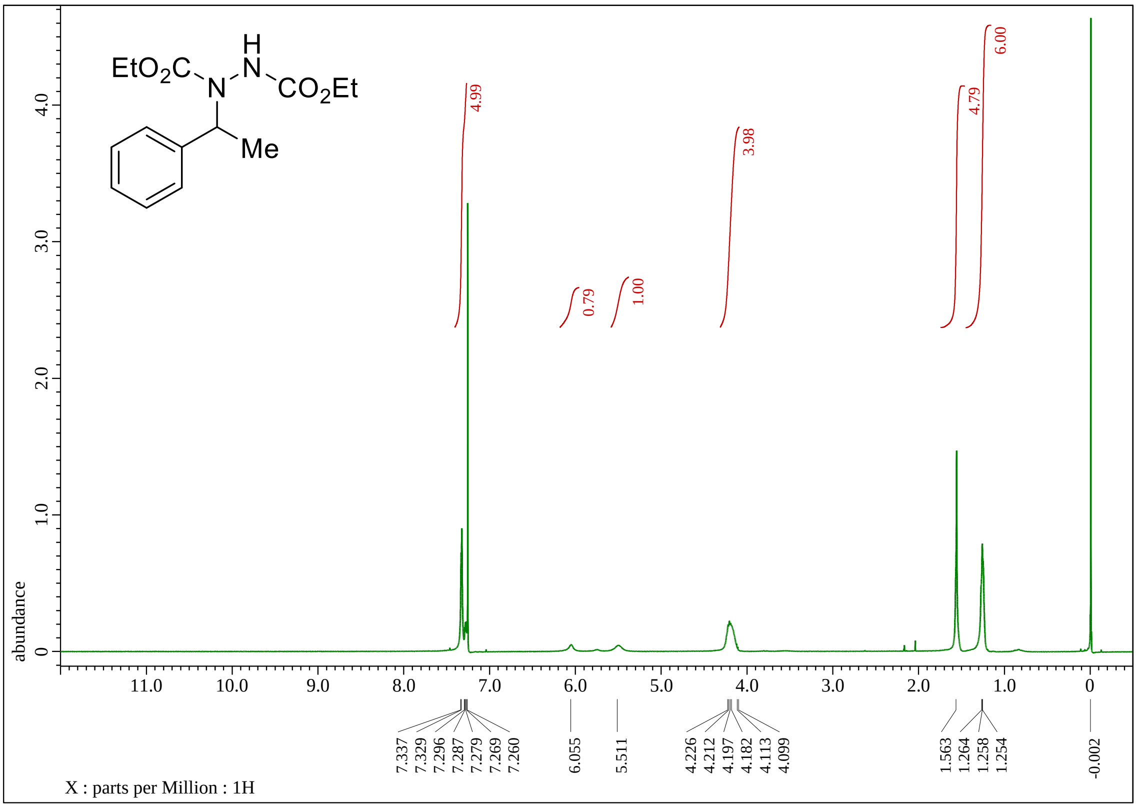

^{13}C NMR of spectrum of **4** (125 MHz, $CDCl_3$)

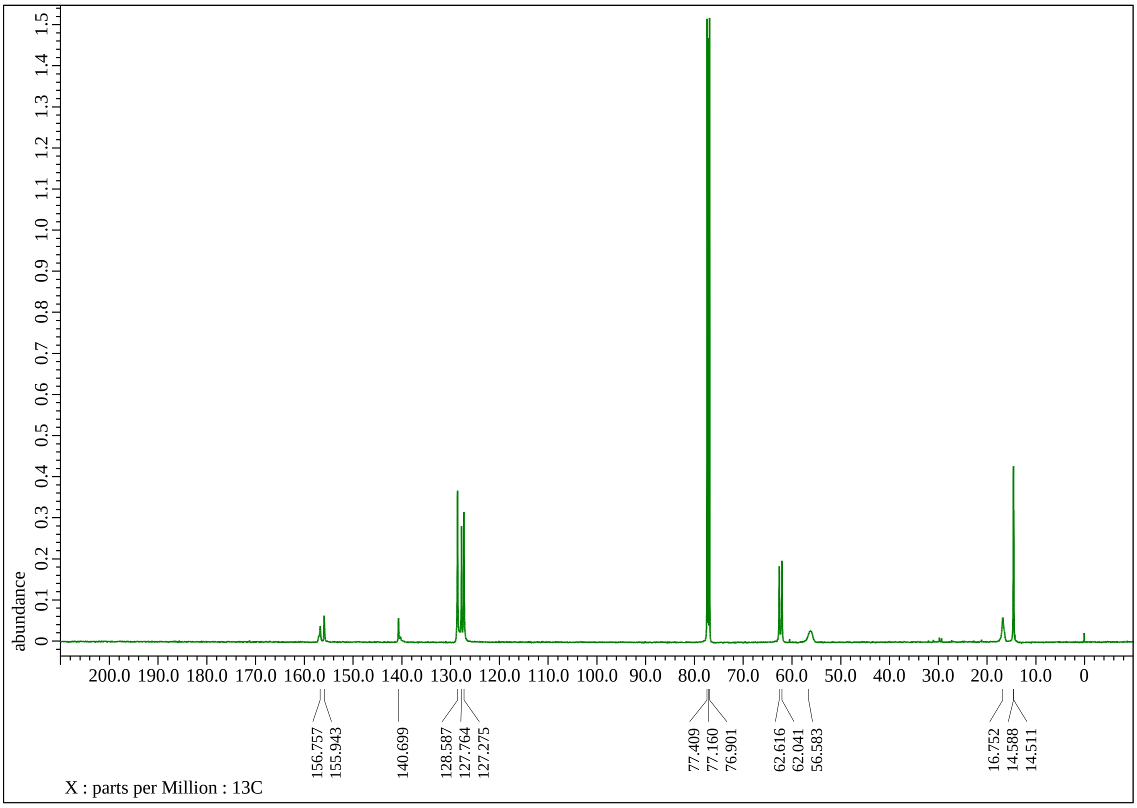

^{1}H NMR of spectrum of **6a** (500 MHz, $CDCl_3$)

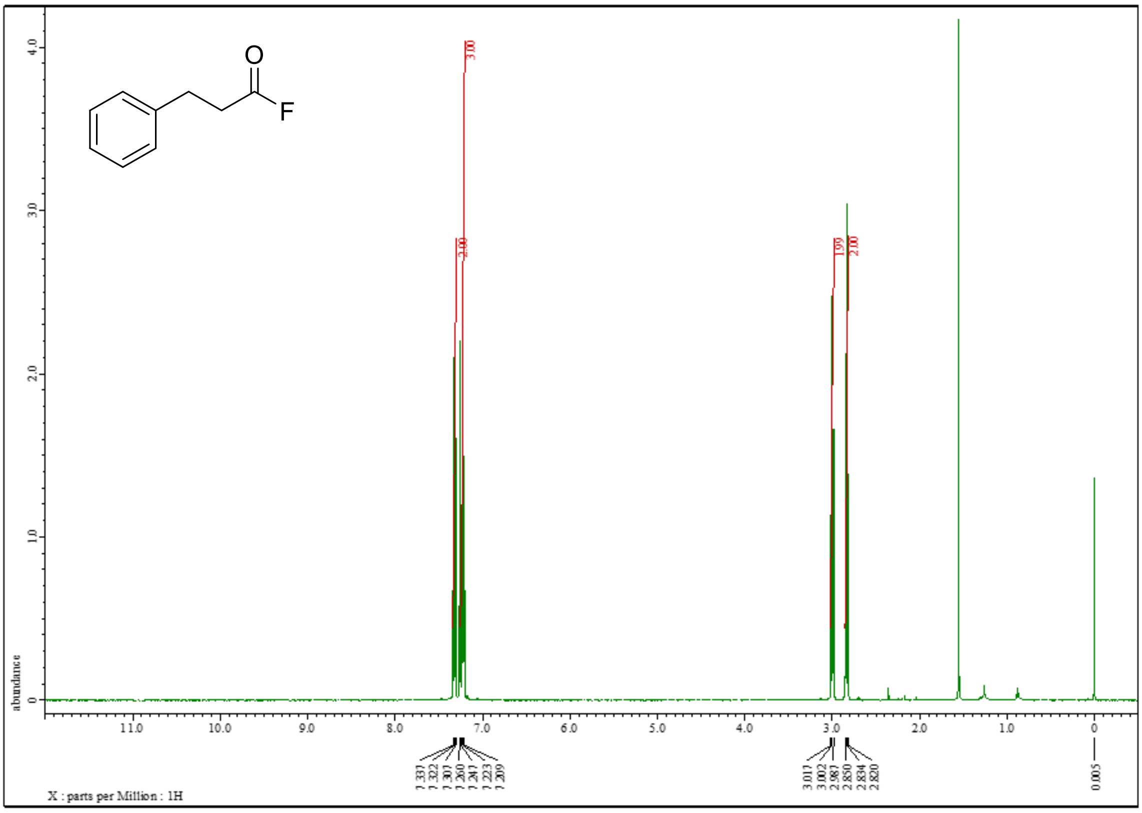

^{13}C NMR of spectrum of **6a** (125 MHz, $CDCl_3$)

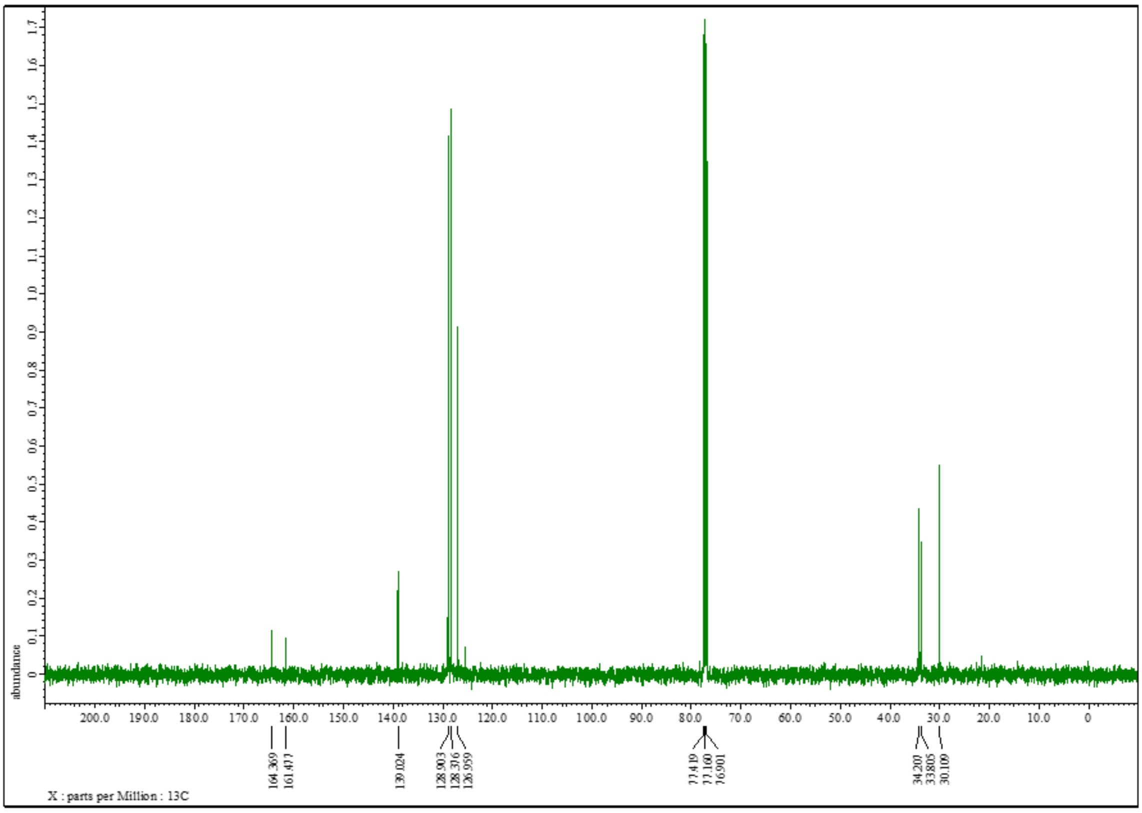

^{19}F NMR of spectrum of **6a** (470 MHz, $CDCl_3$)

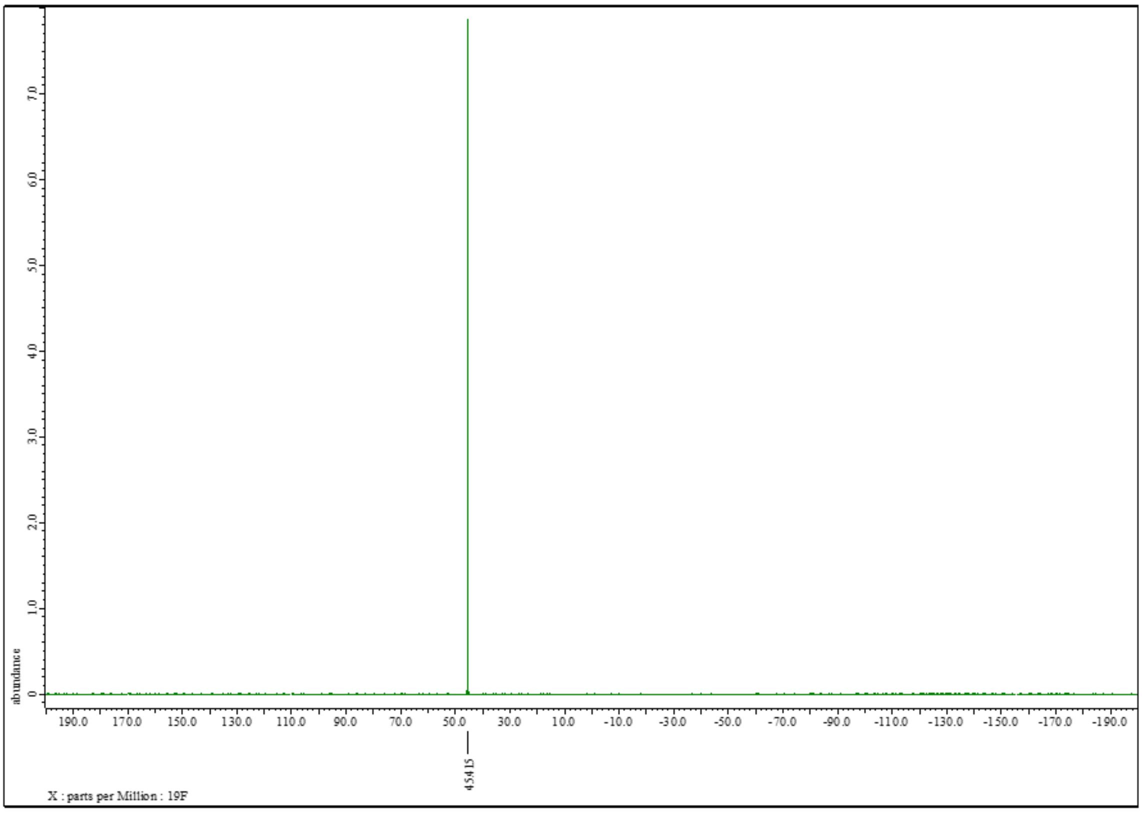

^{1}H NMR of spectrum of **6d** (500 MHz, $CDCl_3$)

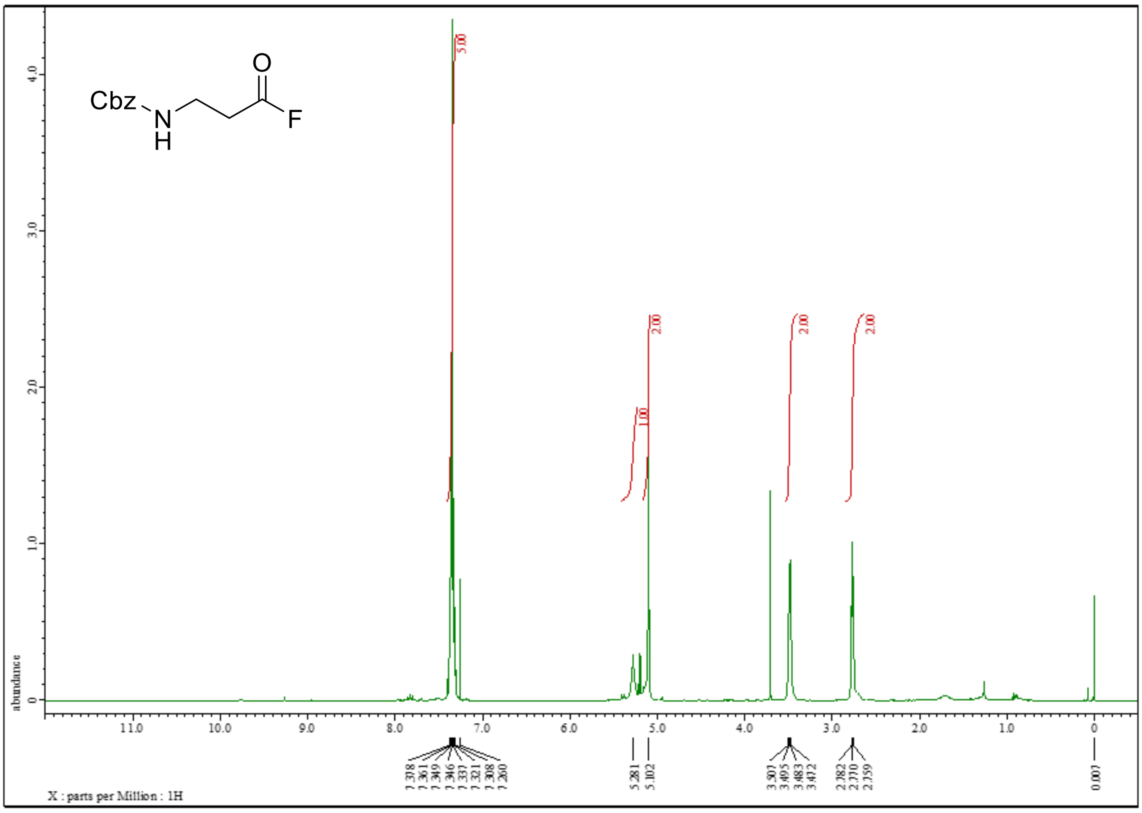

^{13}C NMR of spectrum of **6d** (125 MHz, $CDCl_3$)

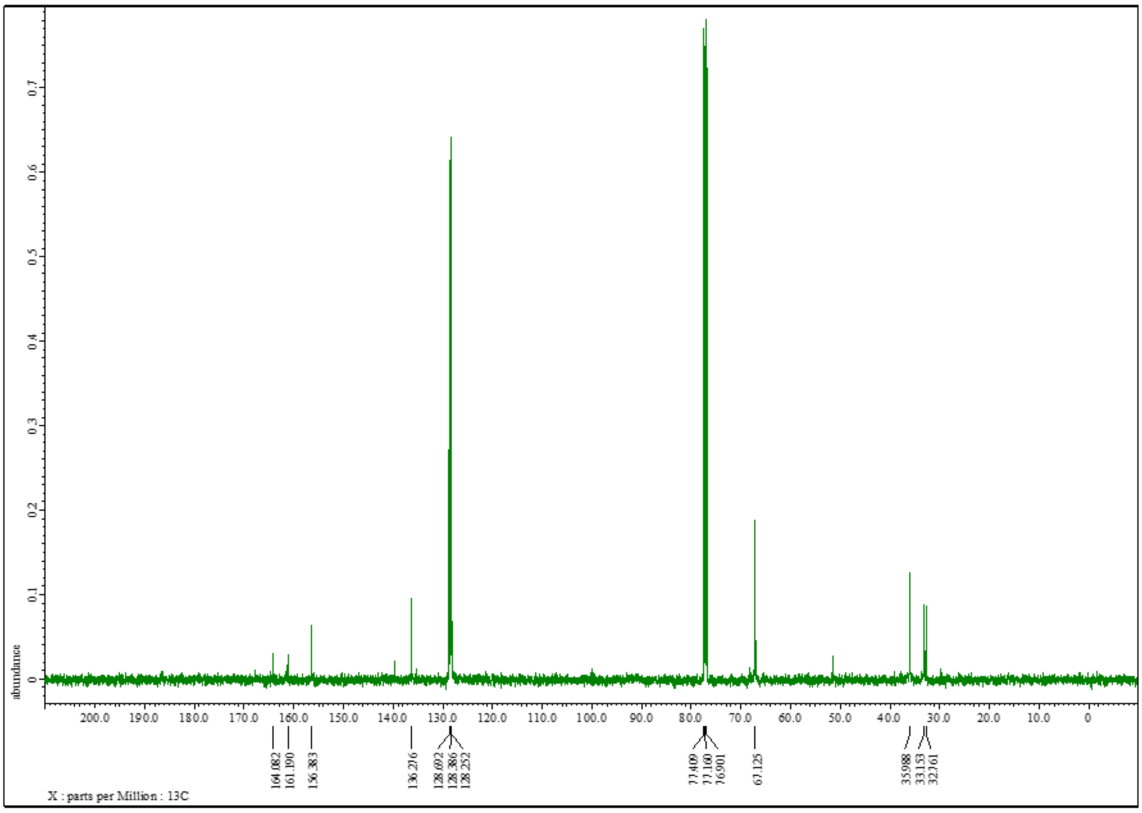

^{19}F NMR of spectrum of **6d** (470 MHz, $CDCl_3$)

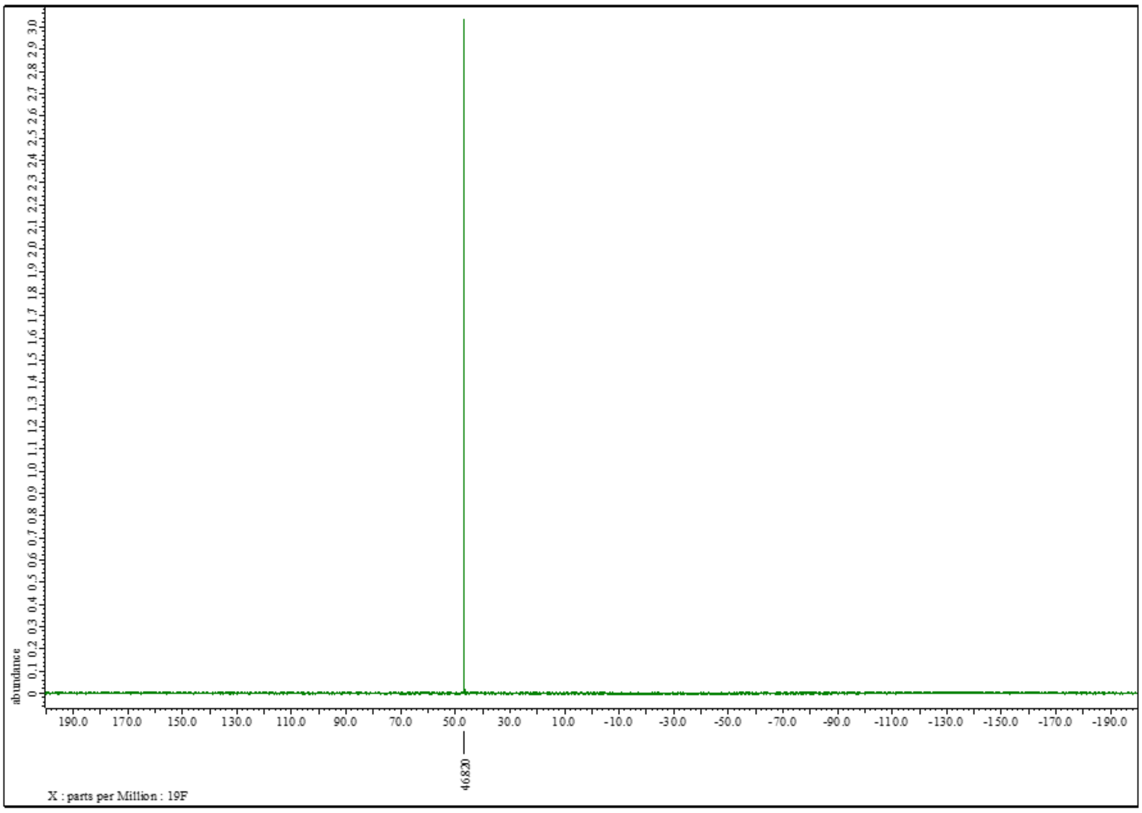

^{1}H NMR of spectrum of **6e** (500 MHz, $CDCl_3$)

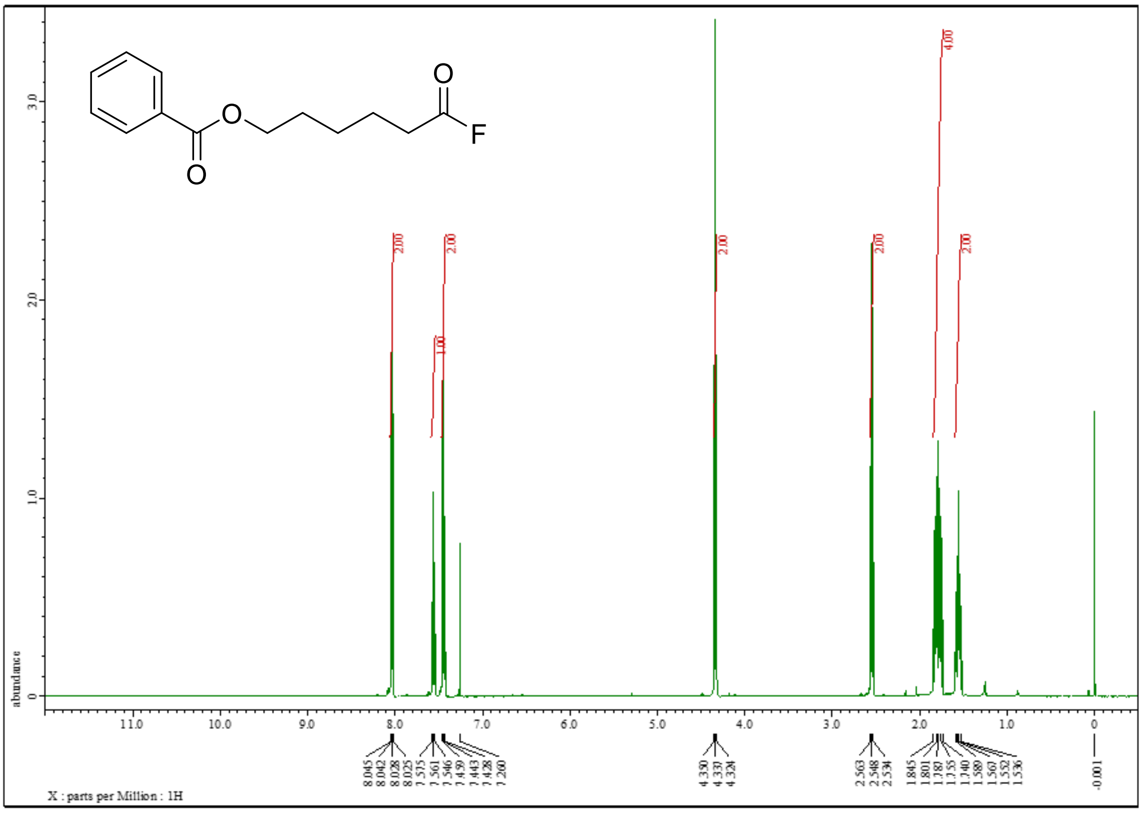

^{13}C NMR of spectrum of **6e** (125 MHz, $CDCl_3$)

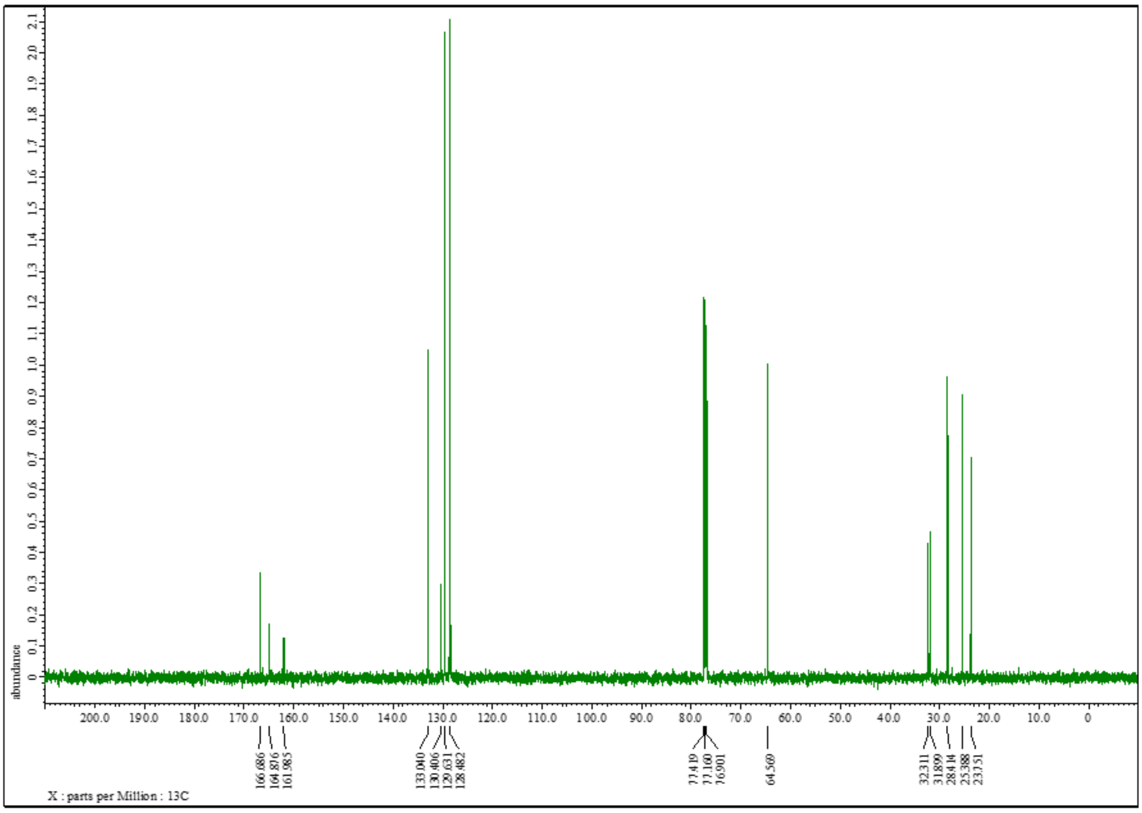

^{19}F NMR of spectrum of **6e** (470 MHz, $CDCl_3$)

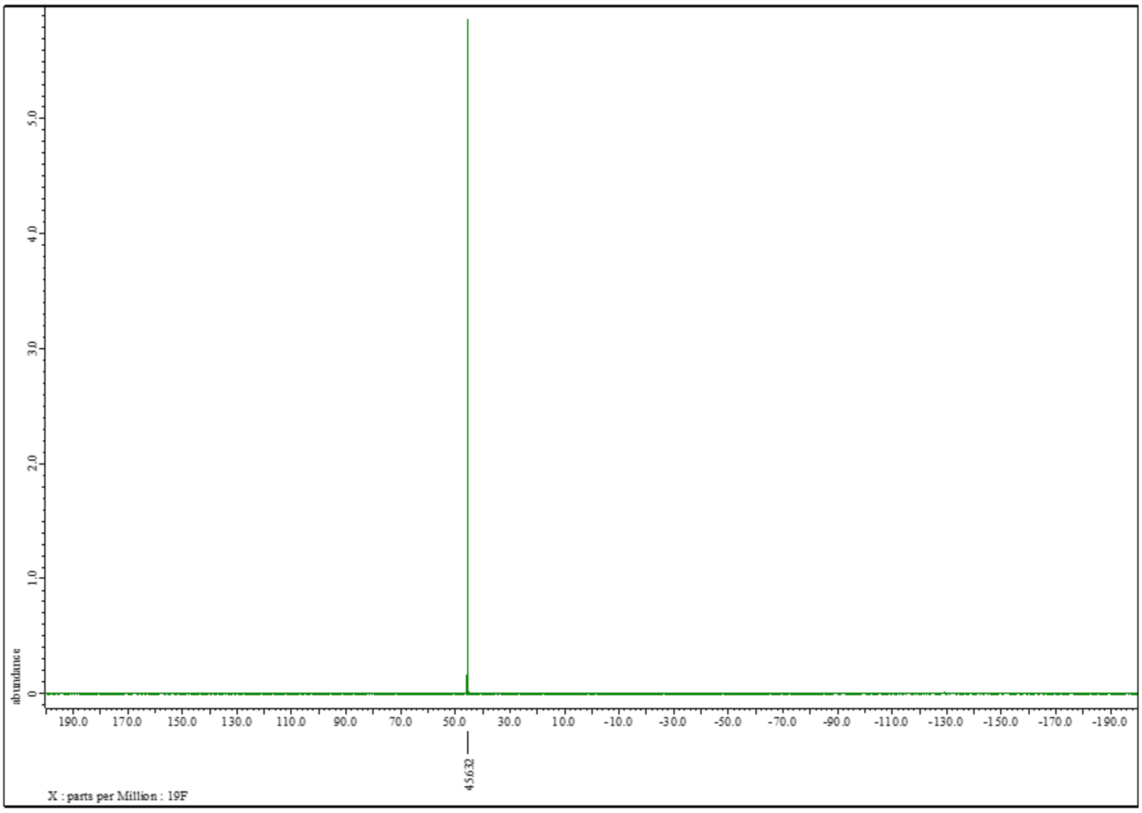

^{1}H NMR of spectrum of **6h** (500 MHz, $CDCl_3$)

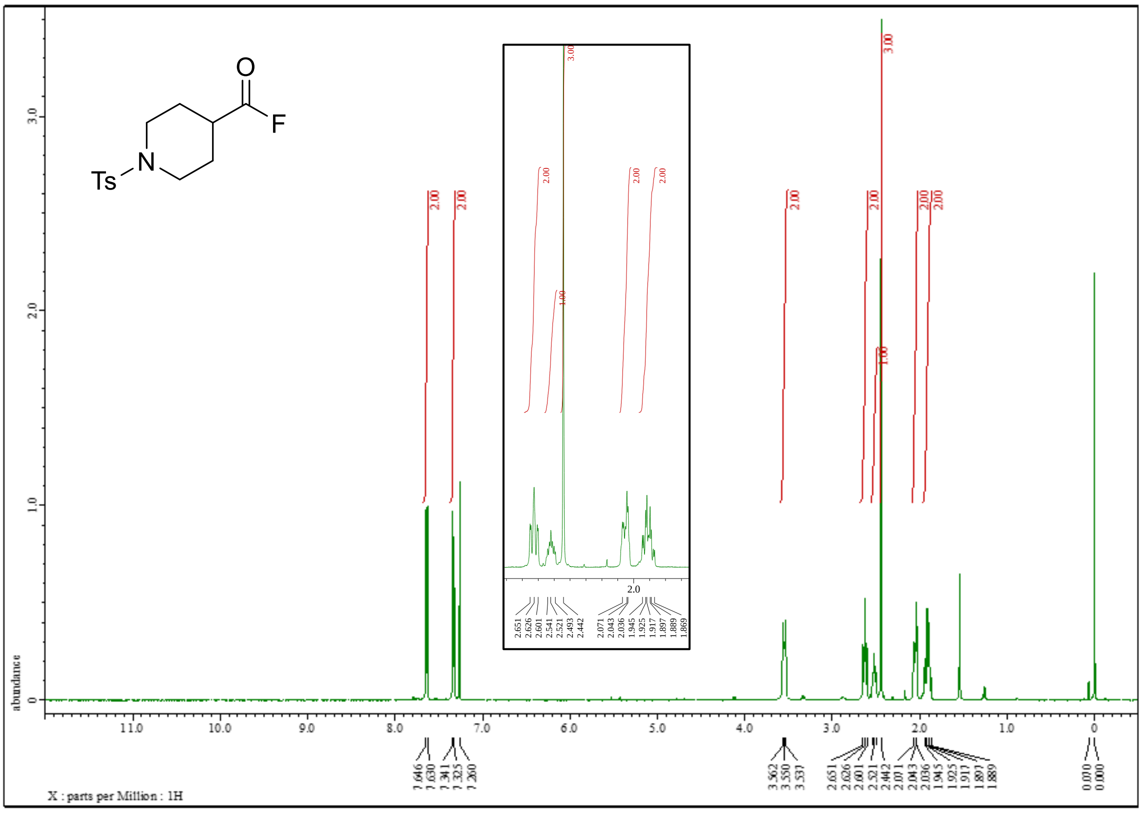

^{13}C NMR of spectrum of **6h** (125 MHz, $CDCl_3$)

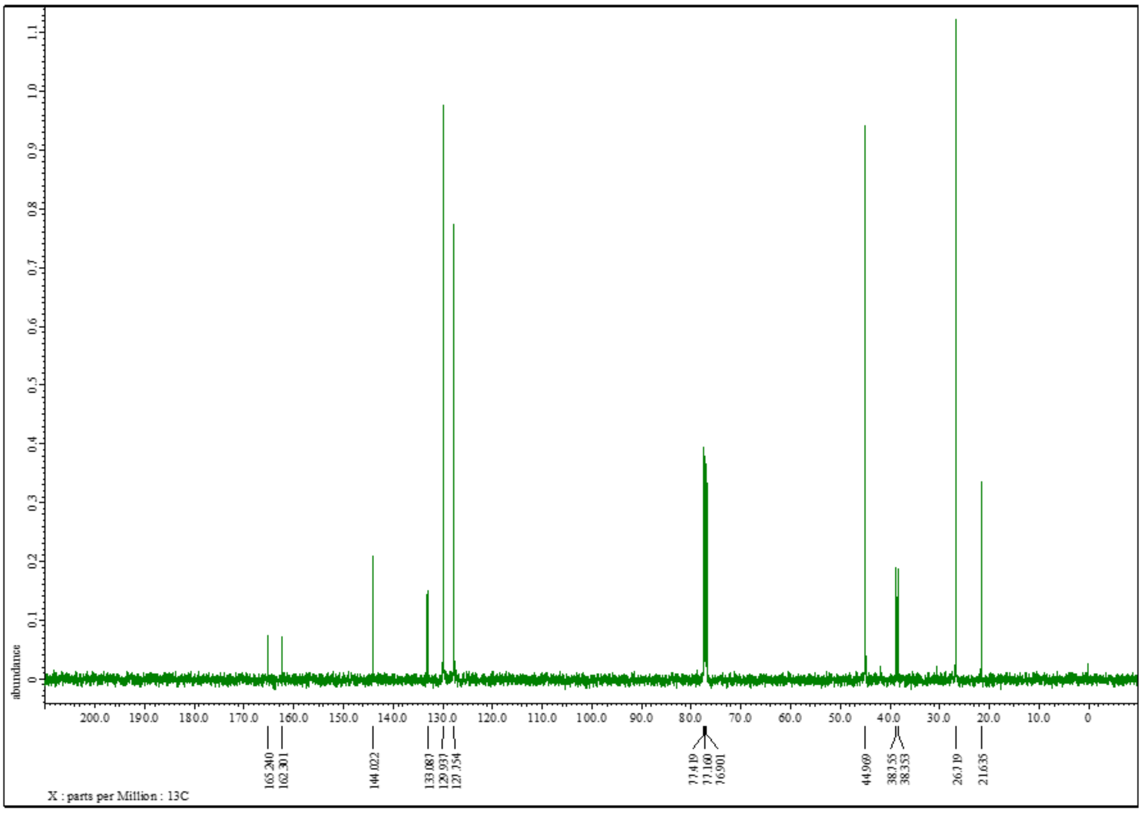

^{19}F NMR of spectrum of **6h** (470 MHz, $CDCl_3$)

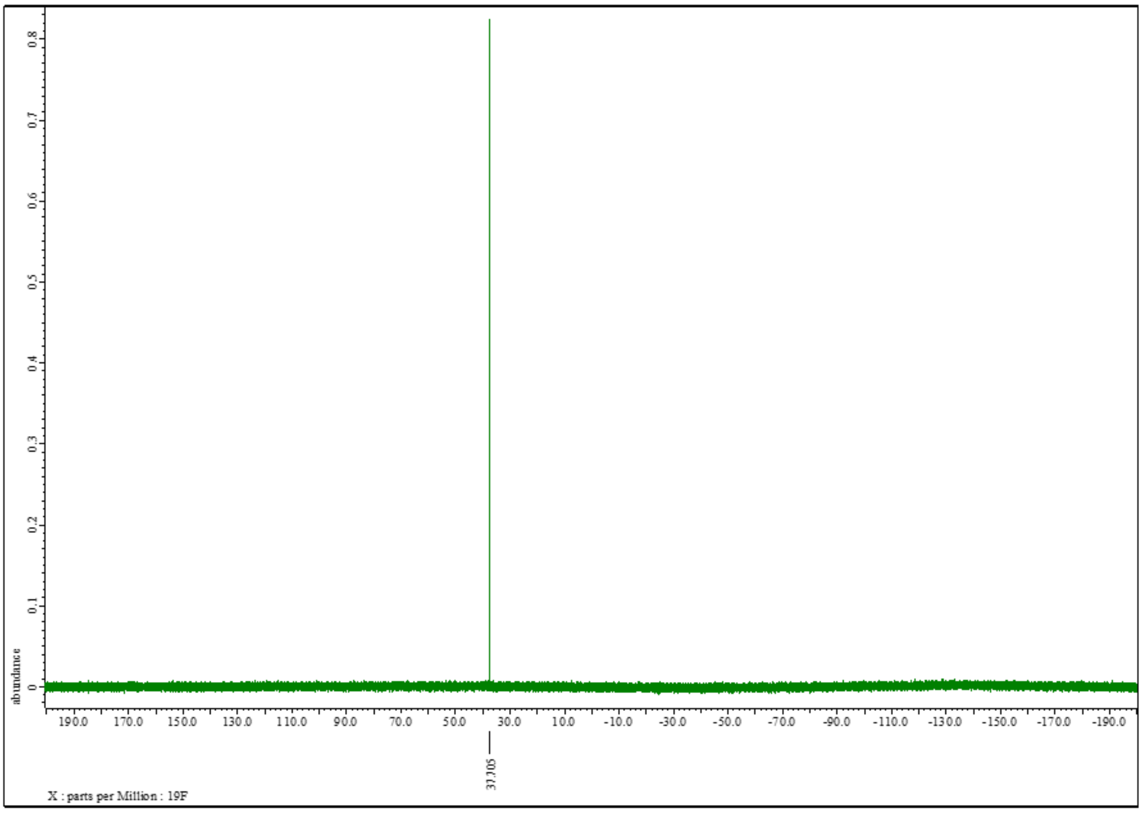

^{1}H NMR of spectrum of **6m** (500 MHz, $CDCl_3$)

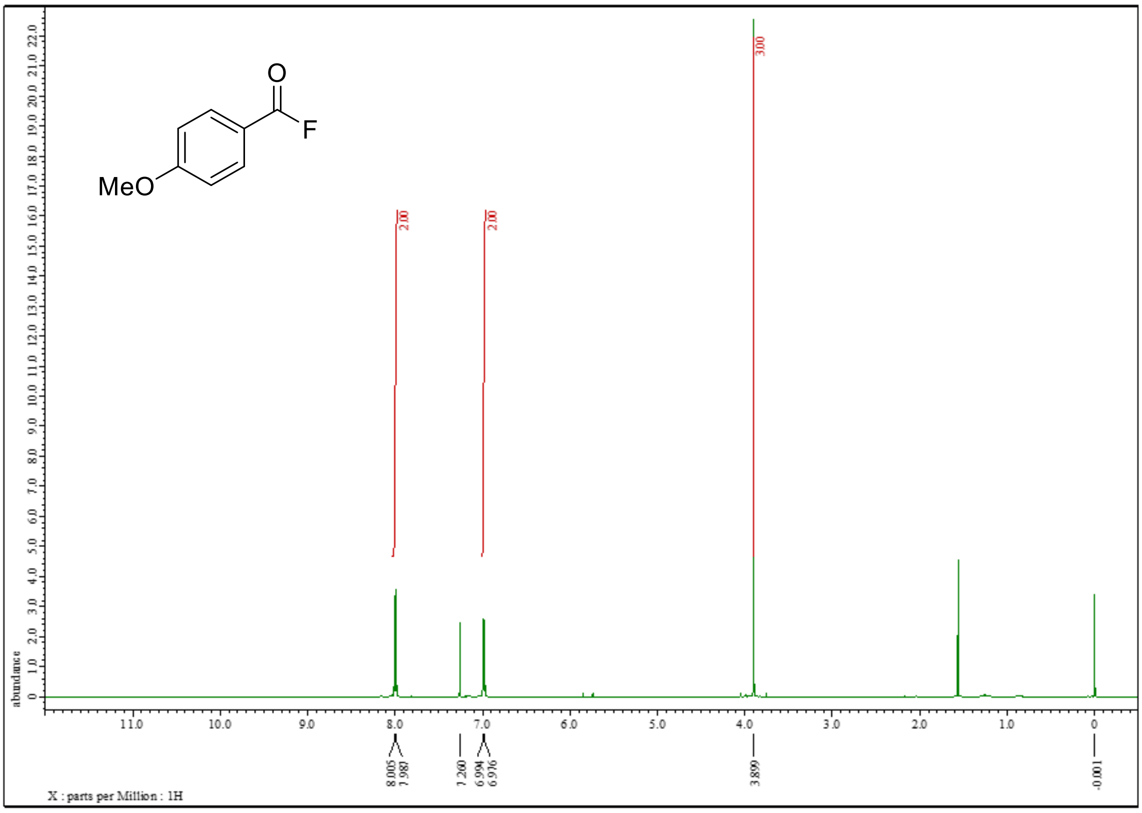

^{13}C NMR of spectrum of **6m** (125 MHz, $CDCl_3$)

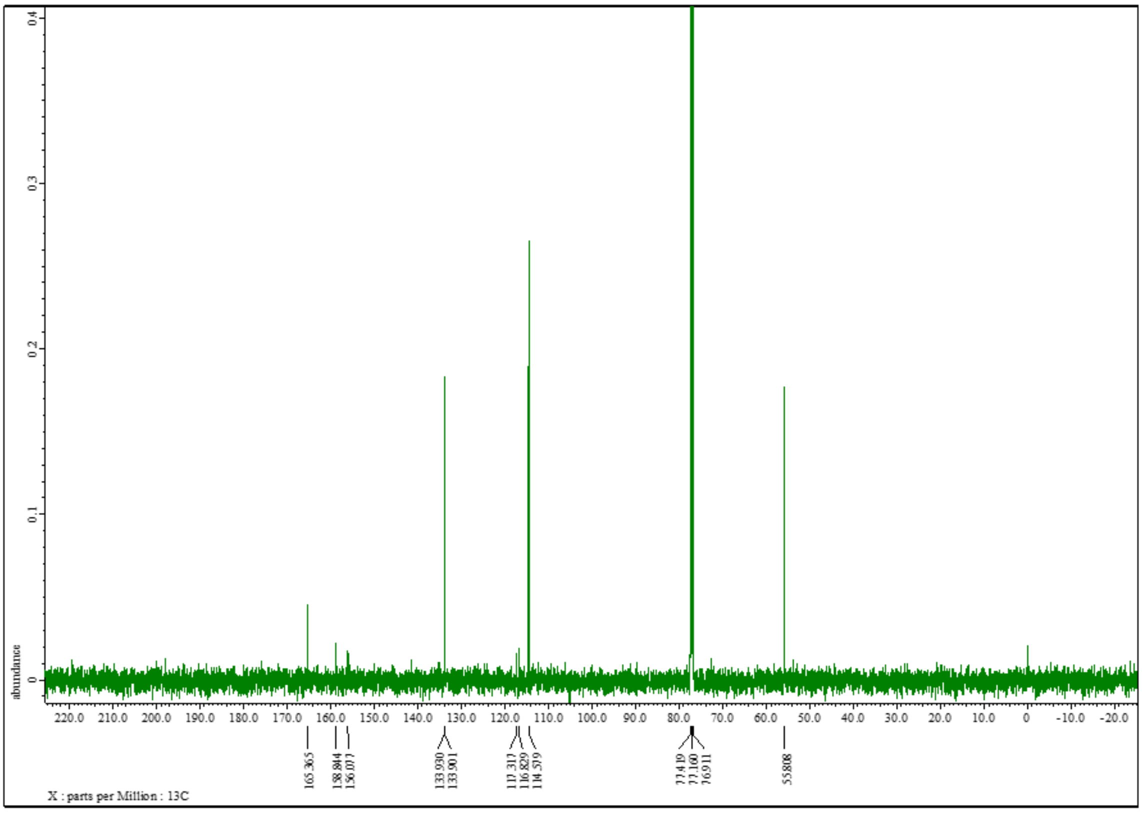

^{19}F NMR of spectrum of **6m** (470 MHz, $CDCl_3$)

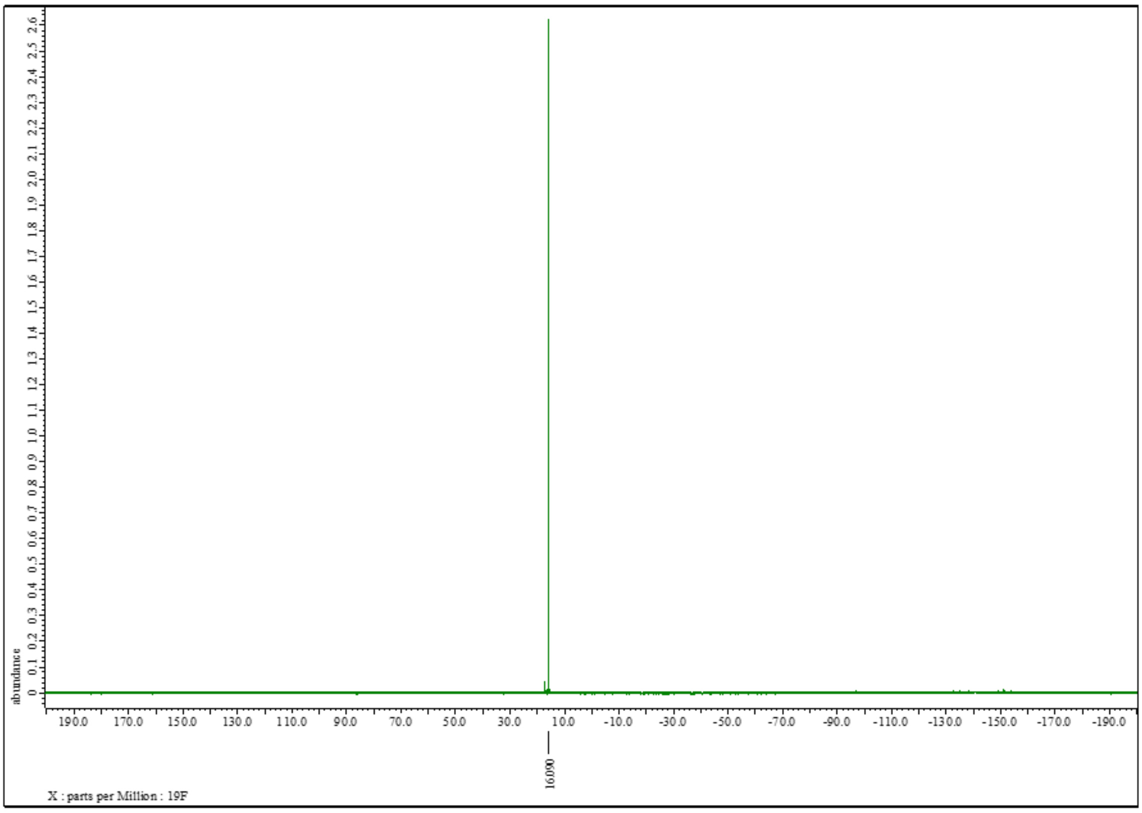

^{1}H NMR of spectrum of **6n** (500 MHz, $CDCl_3$)

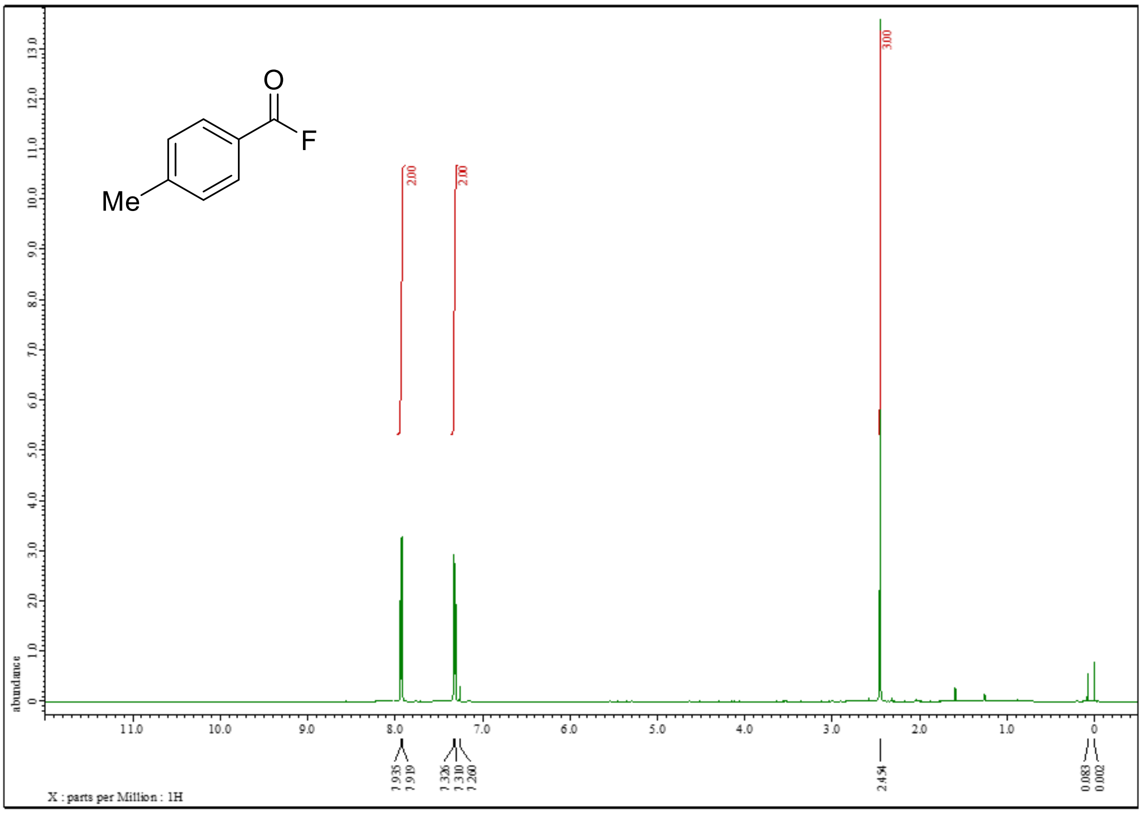

^{13}C NMR of spectrum of **6n** (125 MHz, $CDCl_3$)

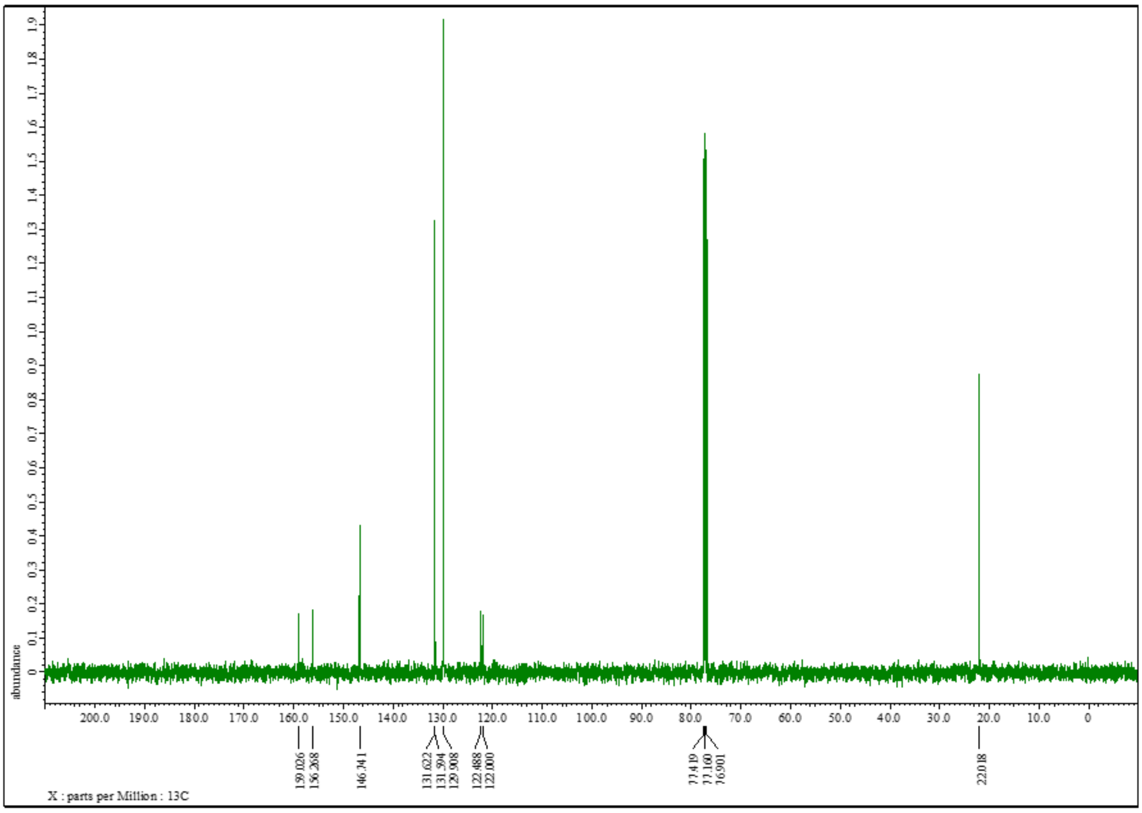

^{19}F NMR of spectrum of **6n** (470 MHz, $CDCl_3$)

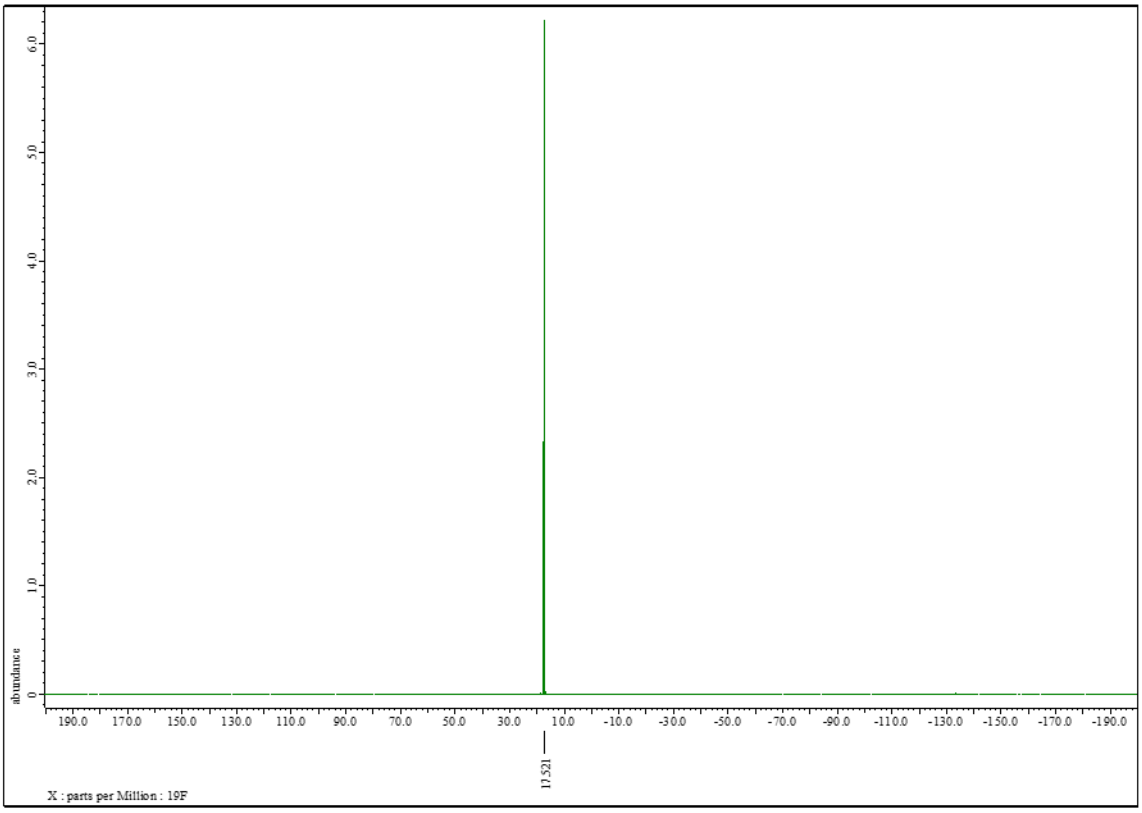

^{1}H NMR of spectrum of **6o** (500 MHz, $CDCl_3$)

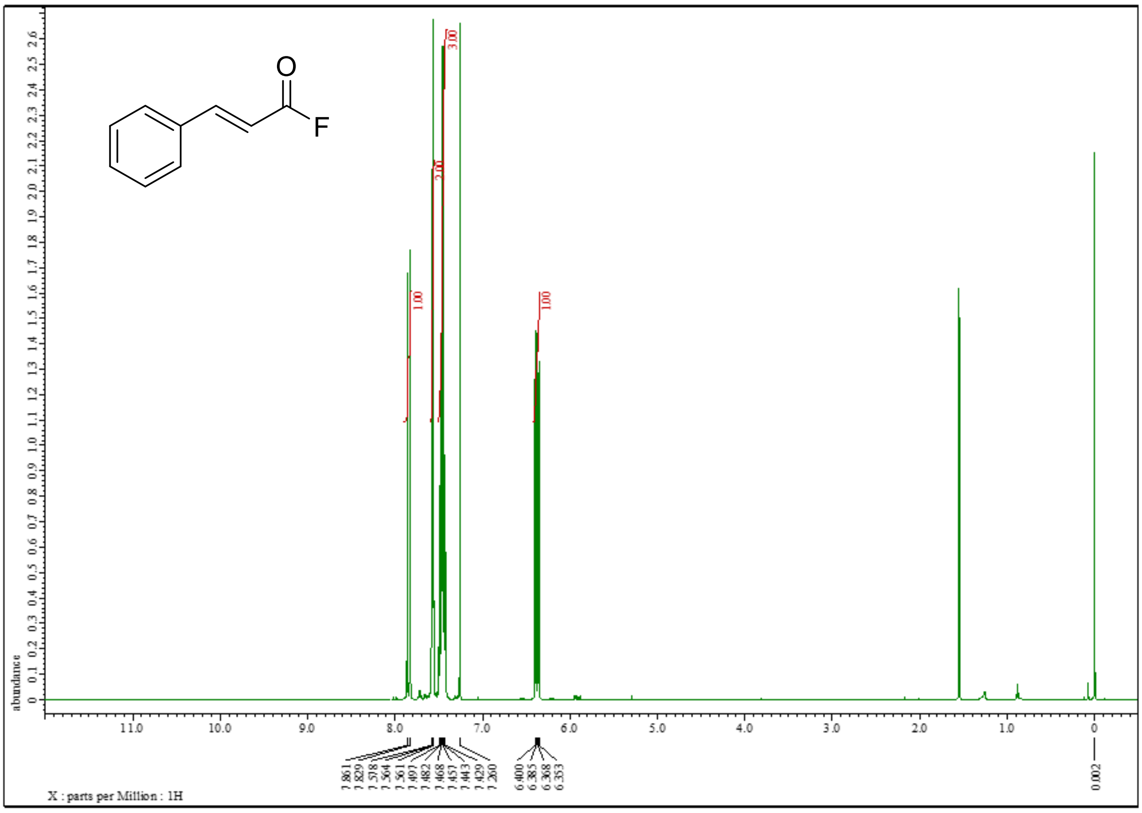

^{13}C NMR of spectrum of **6o** (125 MHz, $CDCl_3$)

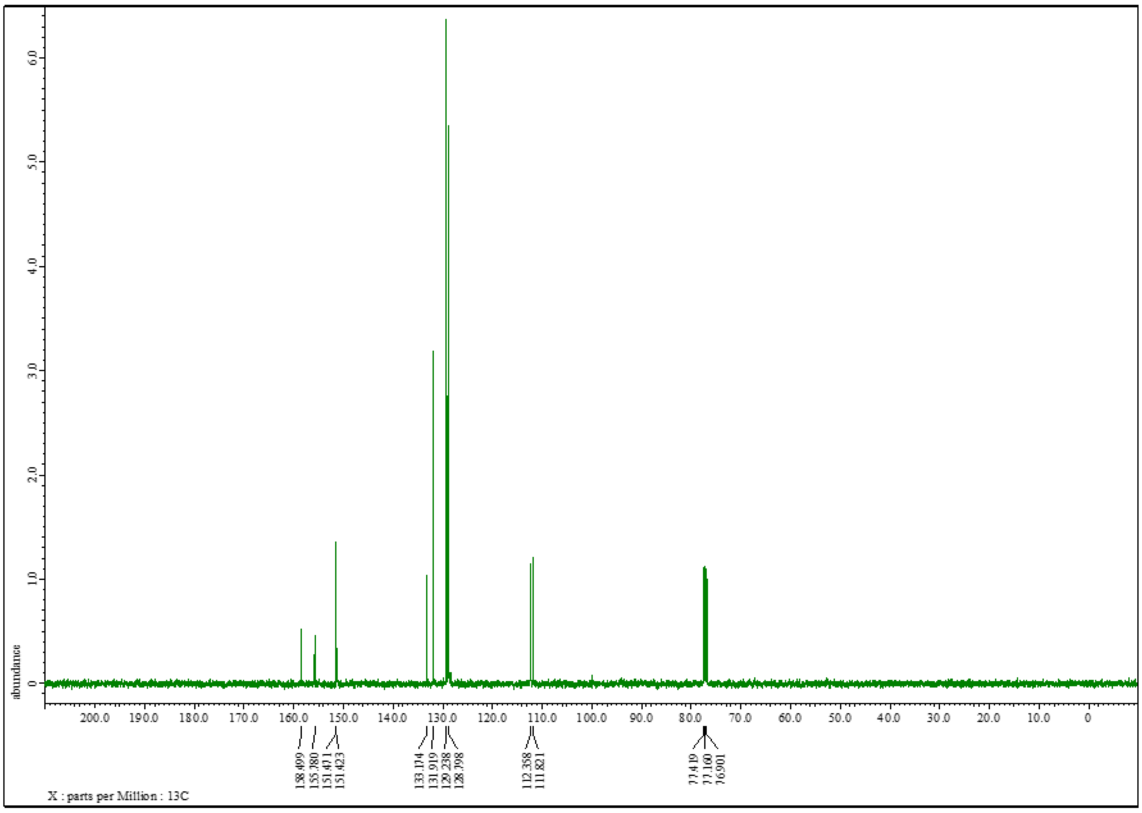

^{1}H NMR spectrum of **7a** (500 MHz, $CDCl_3$)

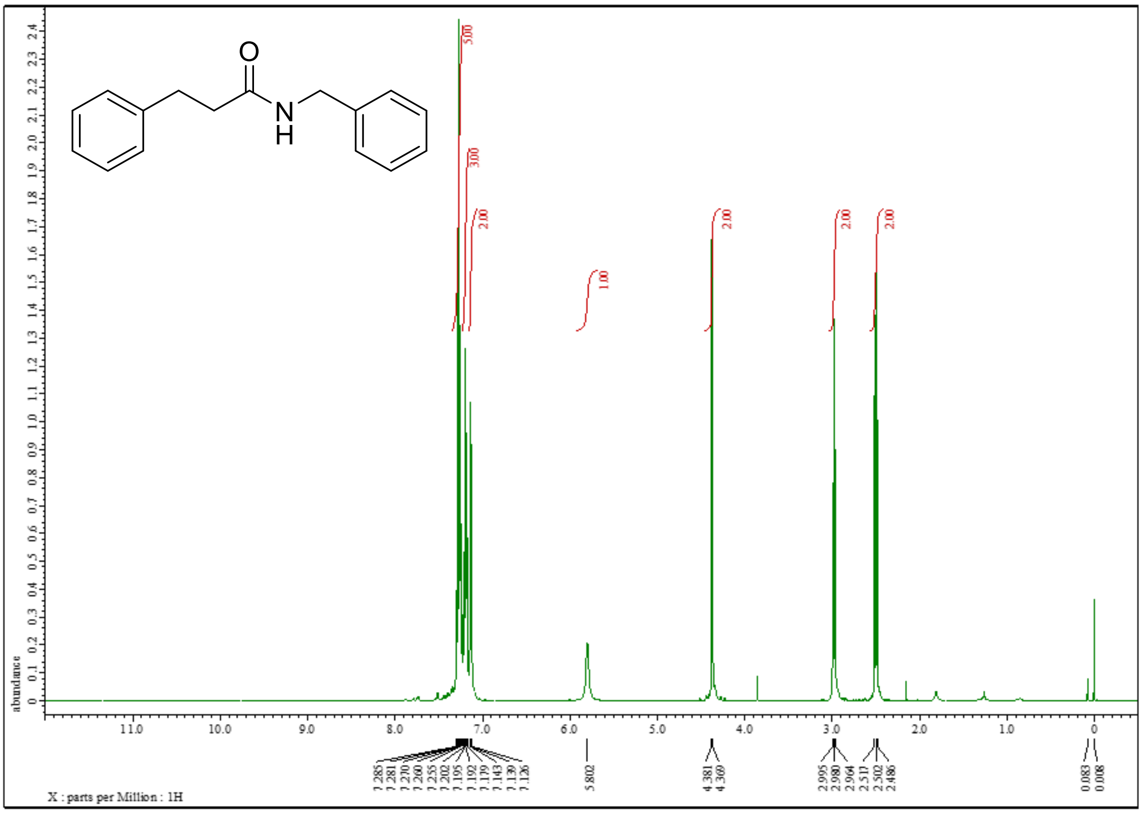

^{13}C NMR spectrum of **7a** (125 MHz, $CDCl_3$)

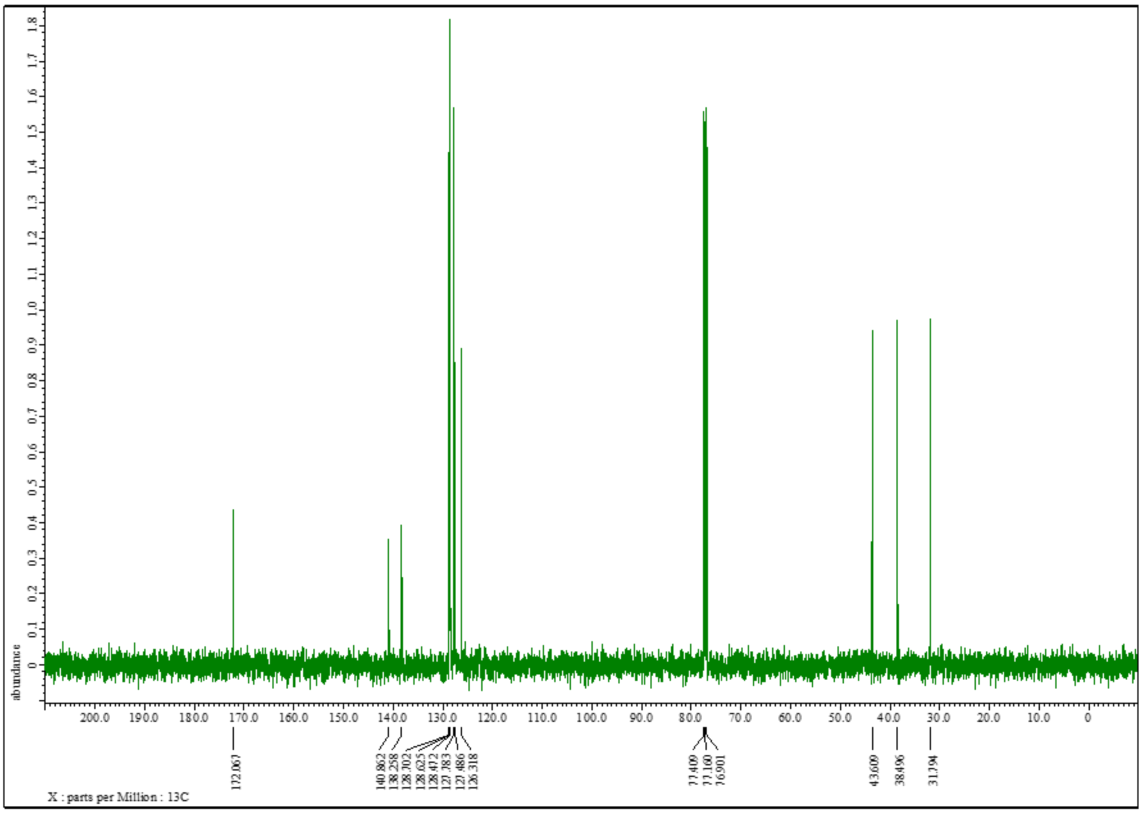

^{1}H NMR of spectrum of **7b** (500 MHz, $CDCl_3$)

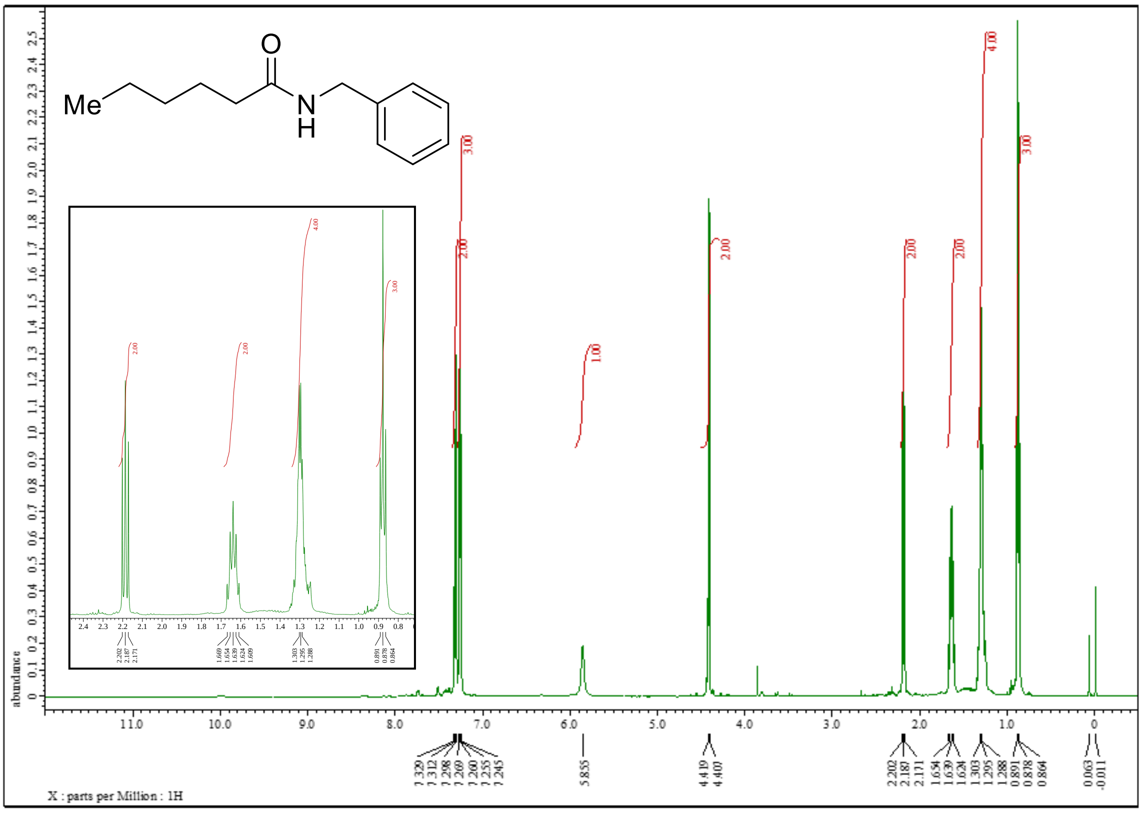

^{13}C NMR of spectrum of **7b** (125 MHz, $CDCl_3$)

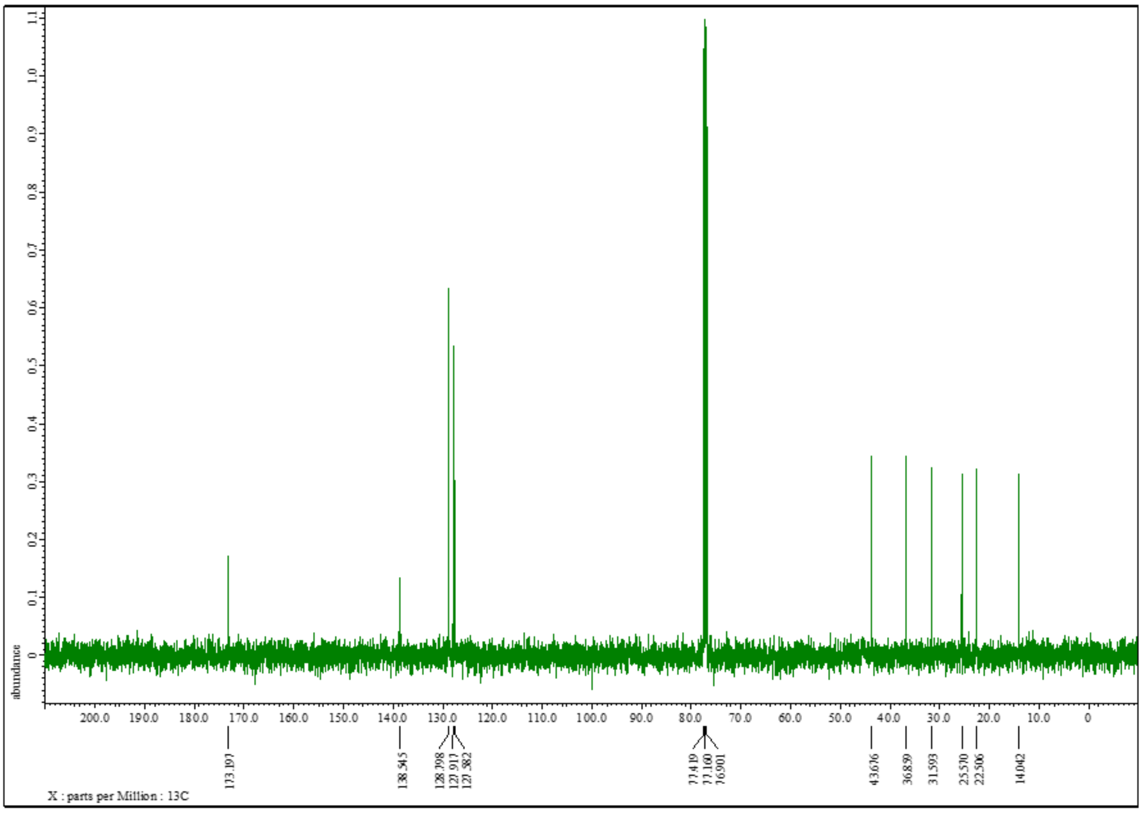

^{1}H NMR spectrum of **7c** (500 MHz, $CDCl_3$)

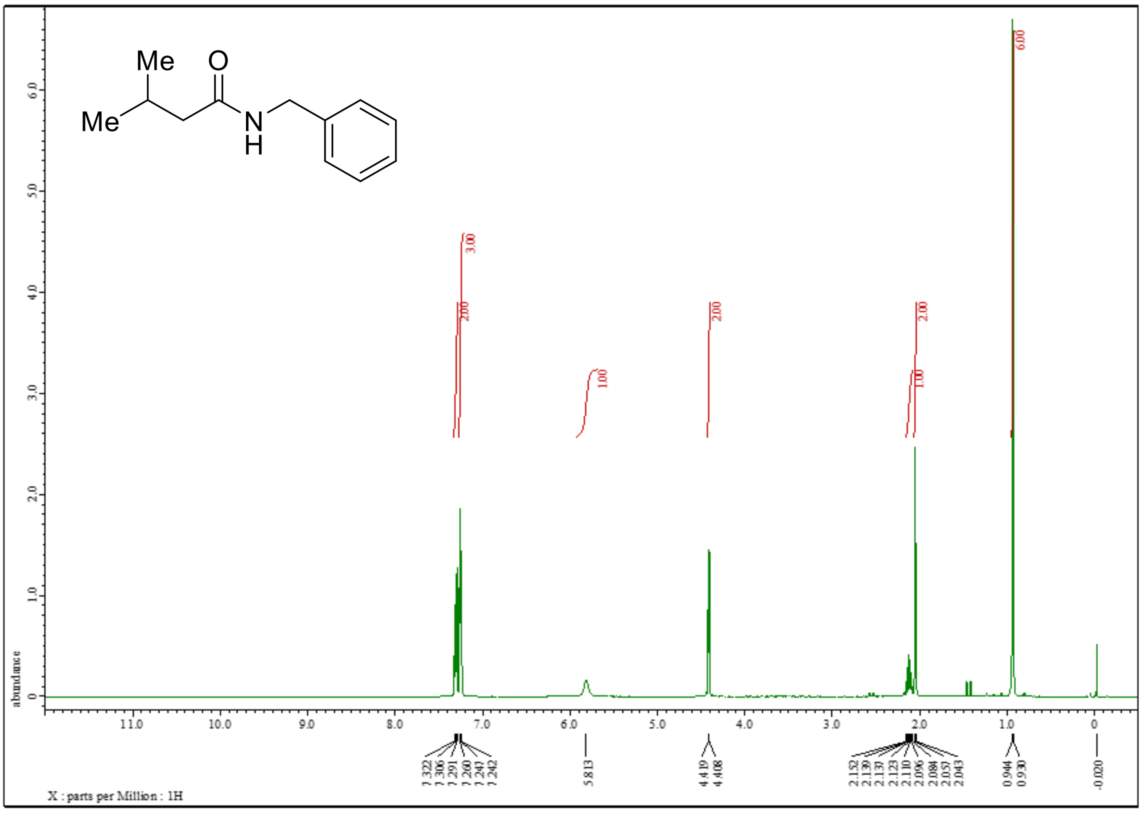

^{13}C NMR spectrum of **7c** (125 MHz, $CDCl_3$)

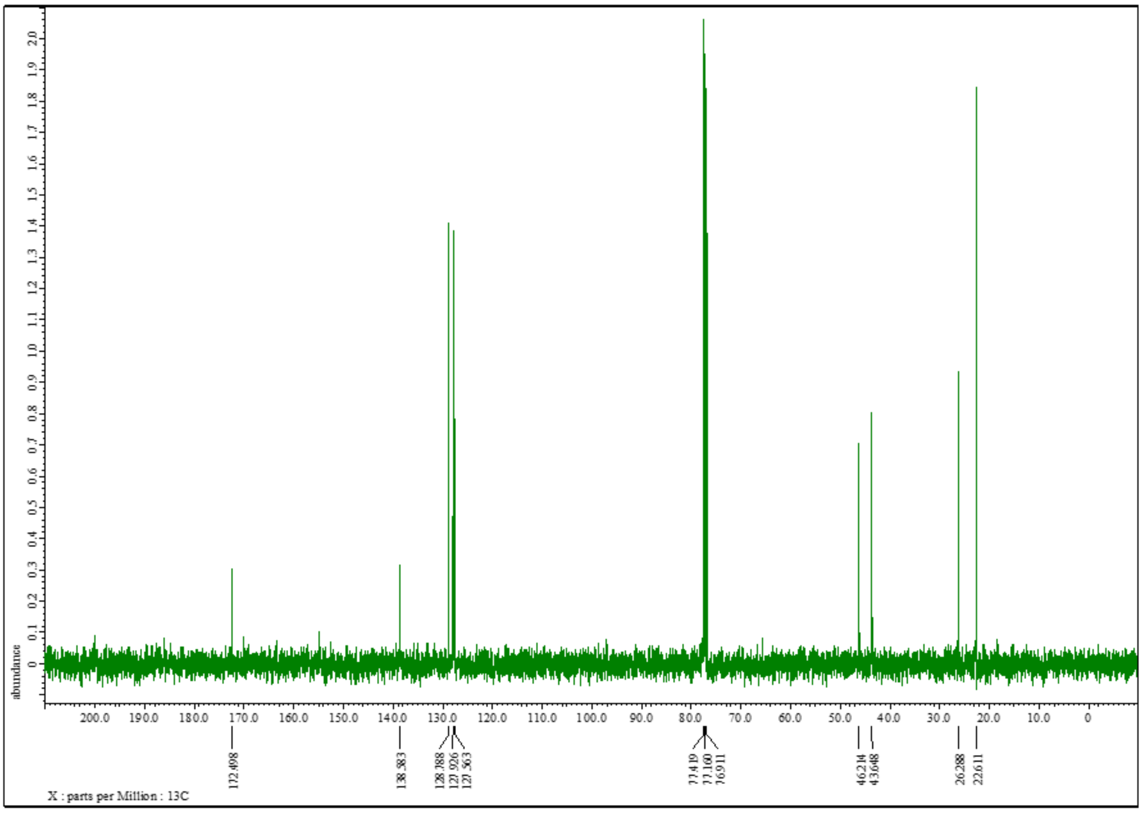

^{1}H NMR spectrum of **7d** (500 MHz, $CDCl_3$)

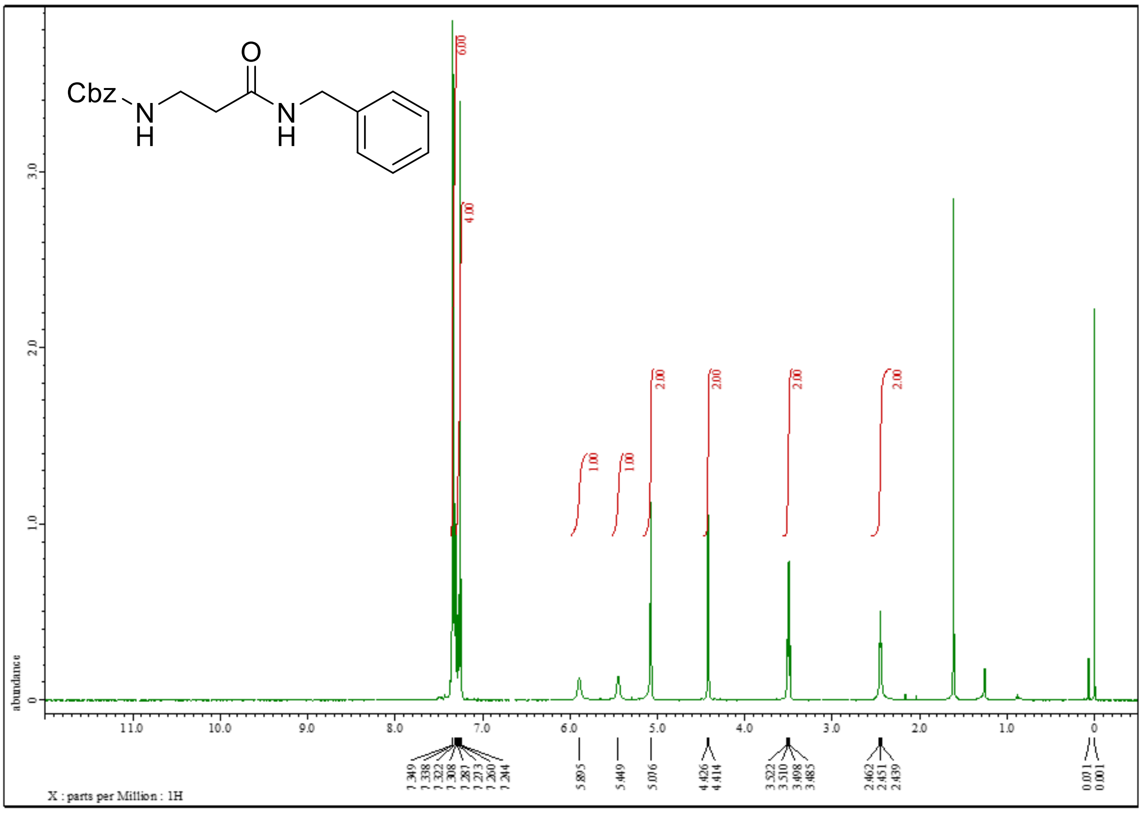

^{13}C NMR of spectrum of **7d** (125 MHz, $CDCl_3$)

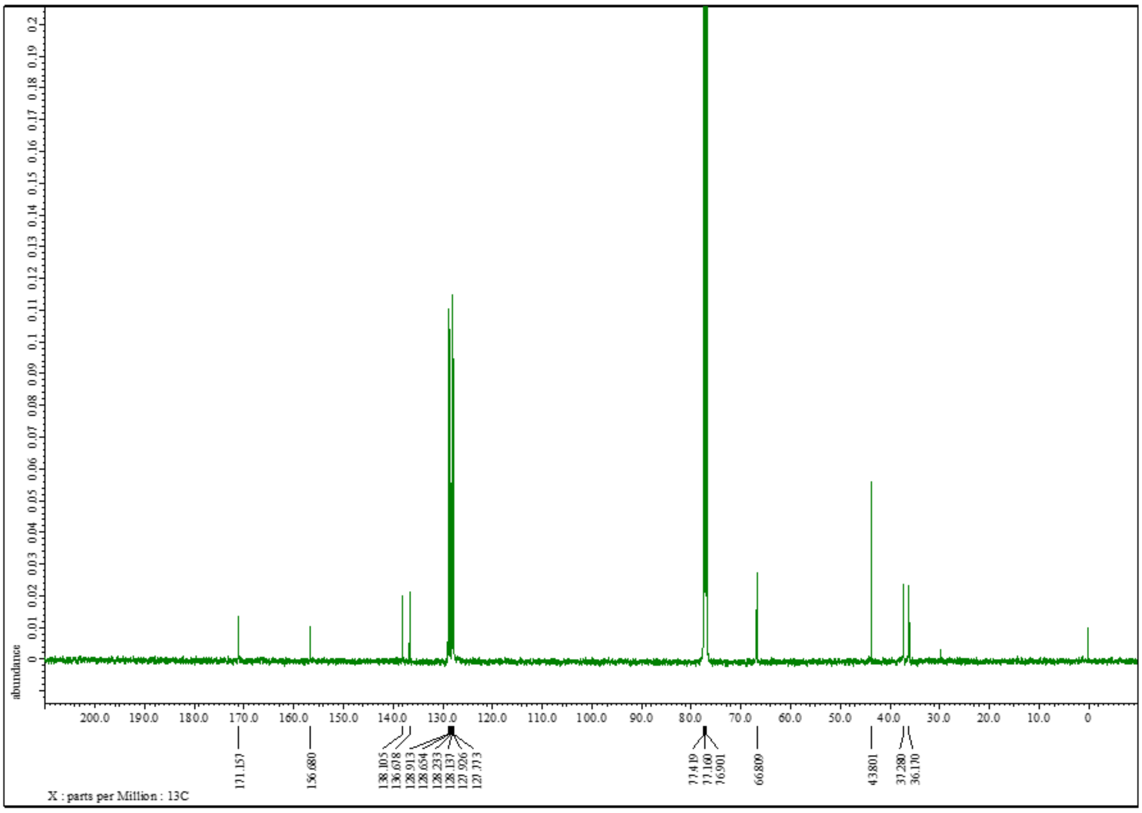

^{1}H NMR of spectrum of **7e** (500 MHz, $CDCl_3$)

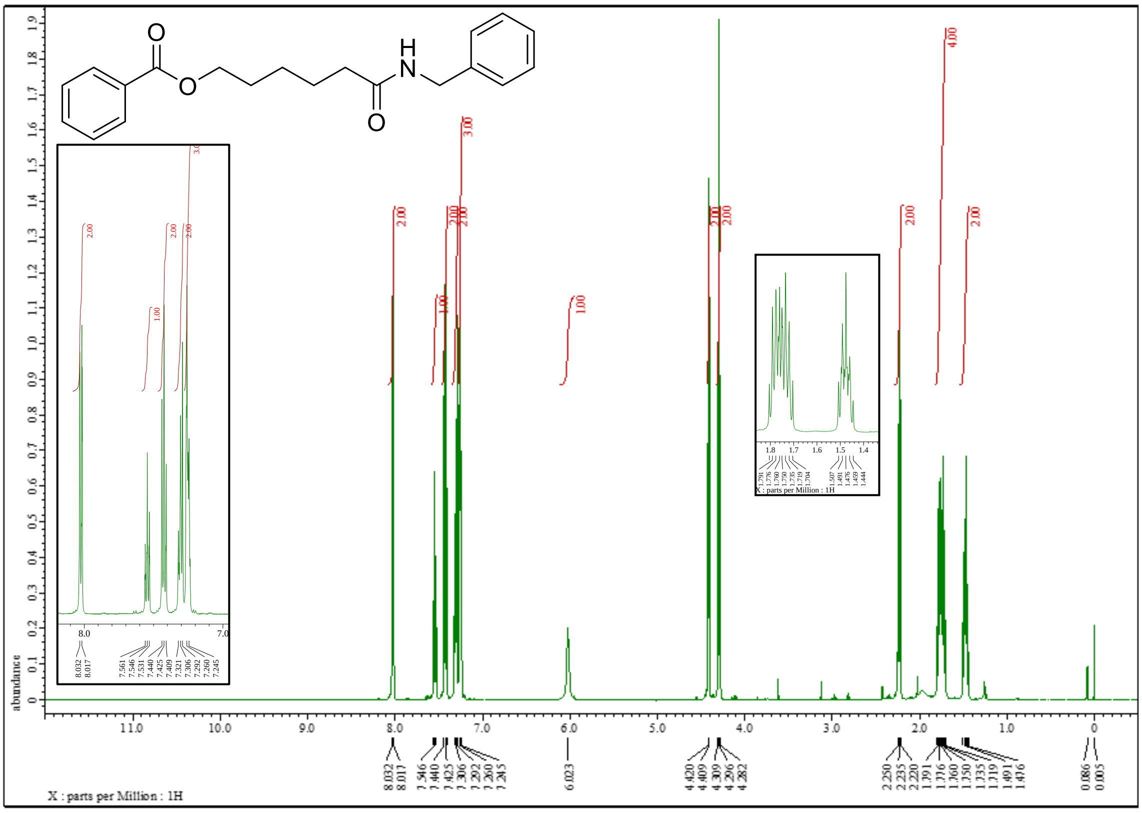

^{13}C NMR of spectrum of **7e** (500 MHz, $CDCl_3$)

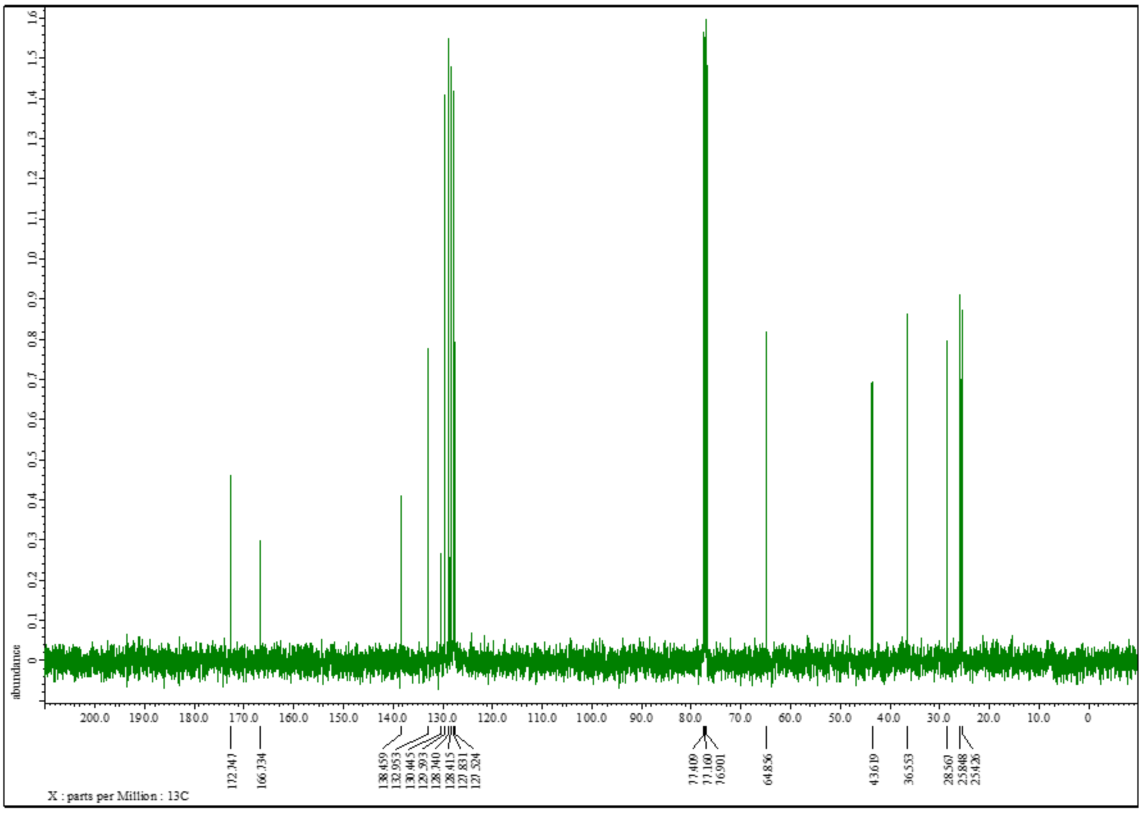

^{1}H NMR spectrum of **7f** (500 MHz, $CDCl_3$)

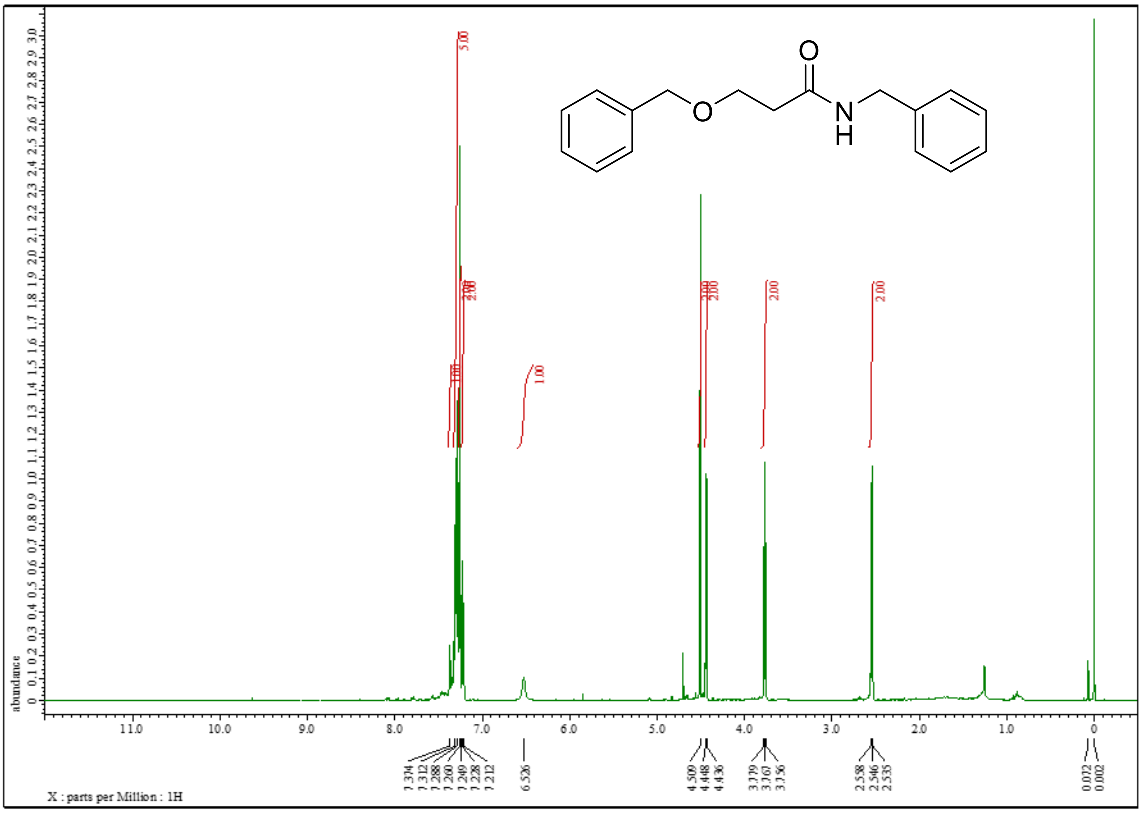

^{13}C NMR of spectrum of **7f** (125 MHz, $CDCl_3$)

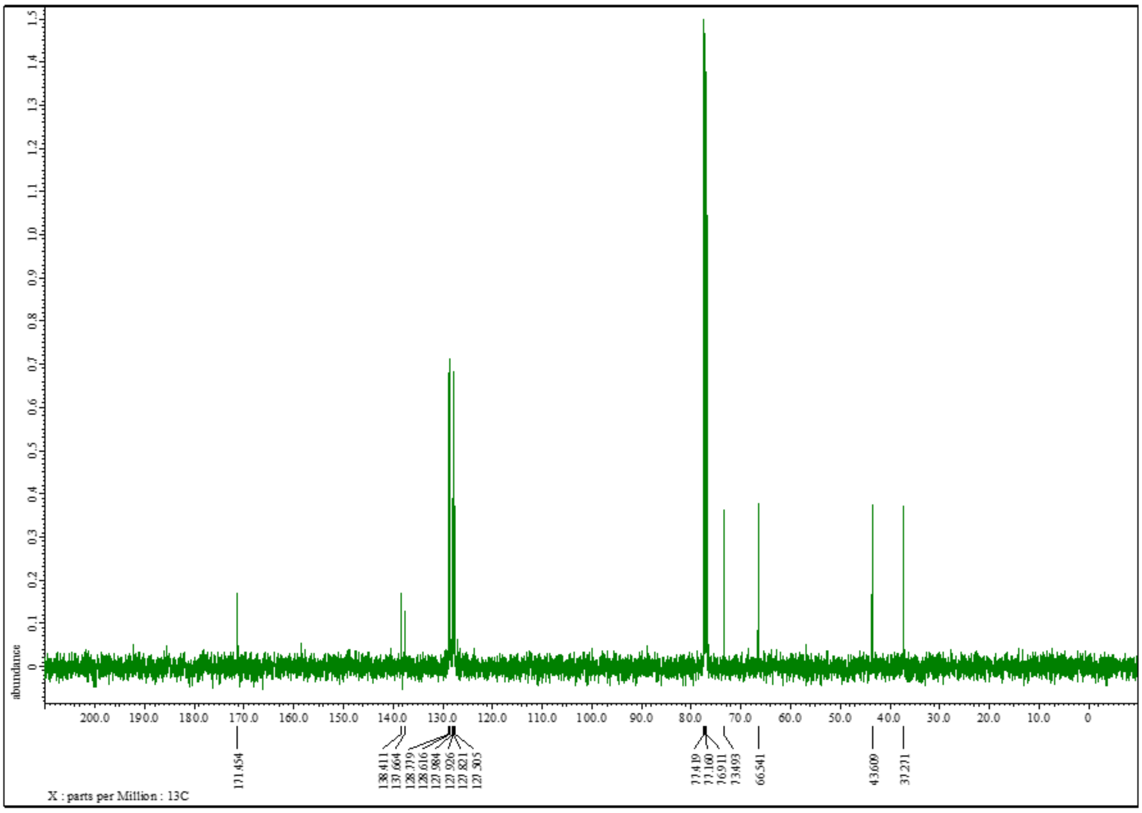

^{1}H NMR spectrum of **7g** (500 MHz, $CDCl_3$)

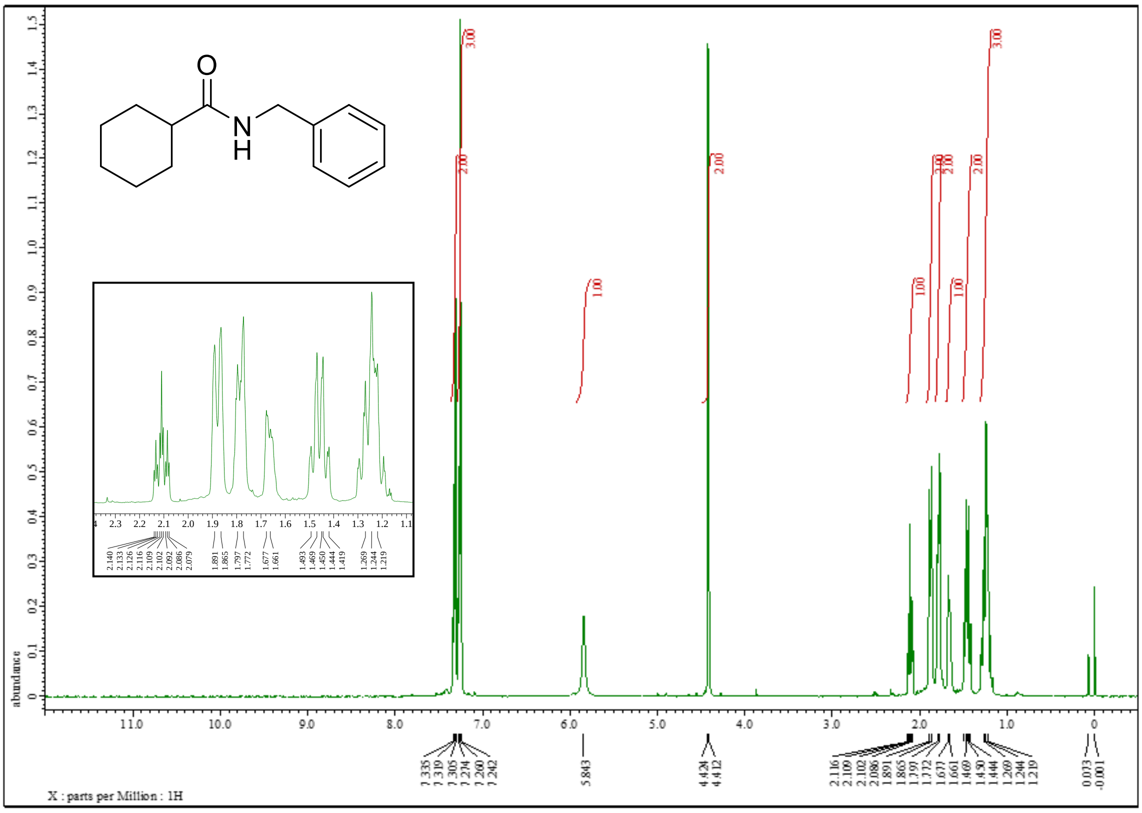

^{13}C NMR spectrum of **7g** (125 MHz, $CDCl_3$)

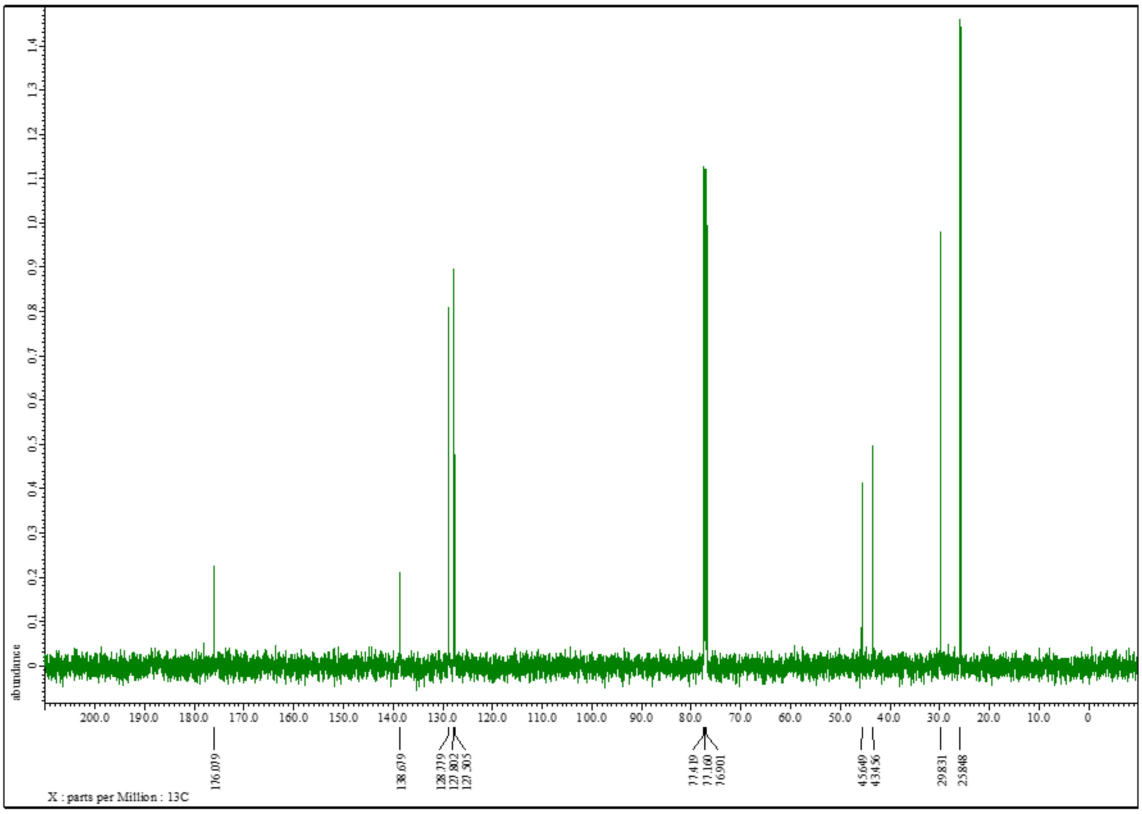

^{1}H NMR spectrum of **7h** (500 MHz, $CDCl_3$)

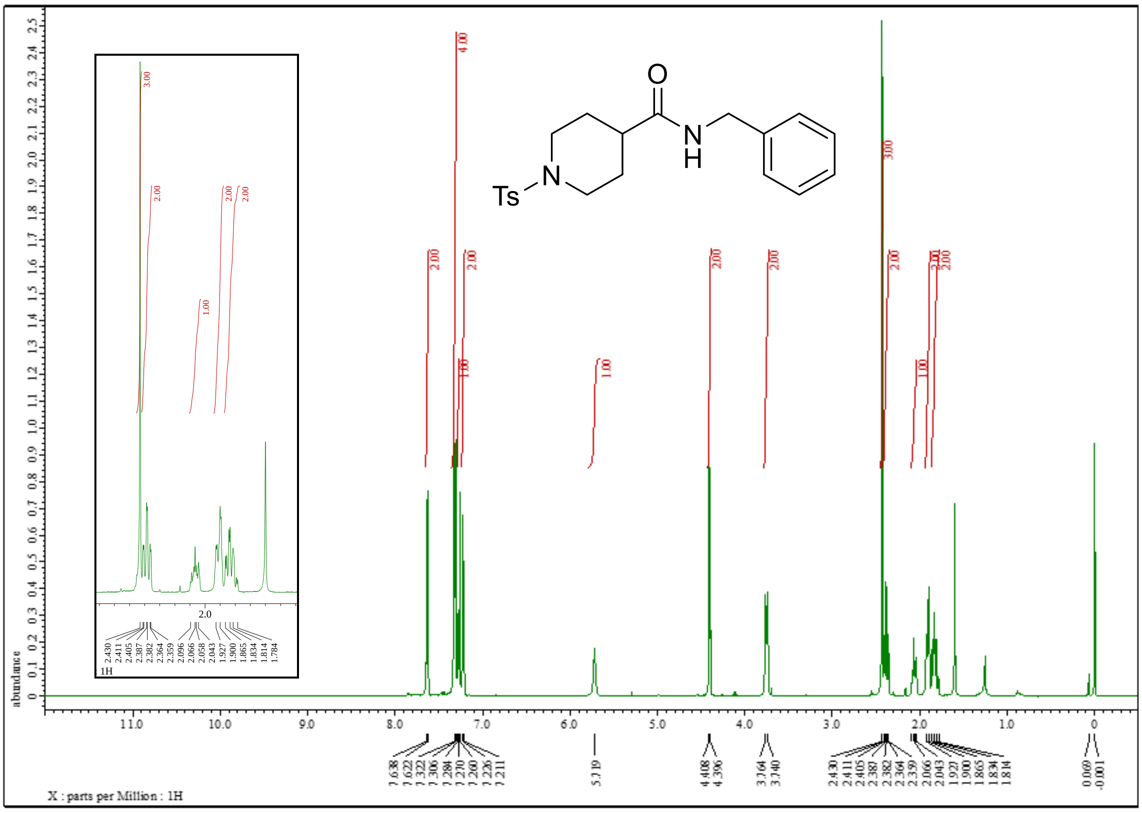

^{13}C NMR of spectrum of **7h** (125 MHz, $CDCl_3$)

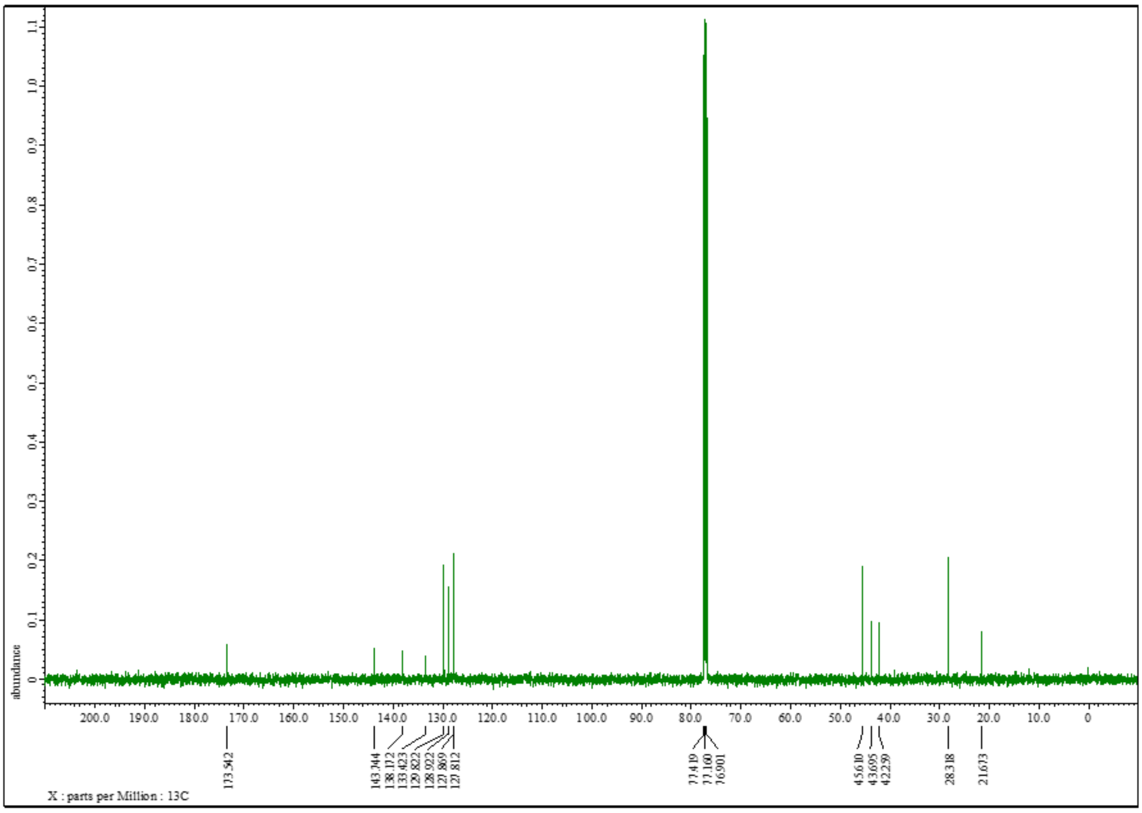

^{1}H NMR of spectrum of **7i** (500 MHz, $CDCl_3$)

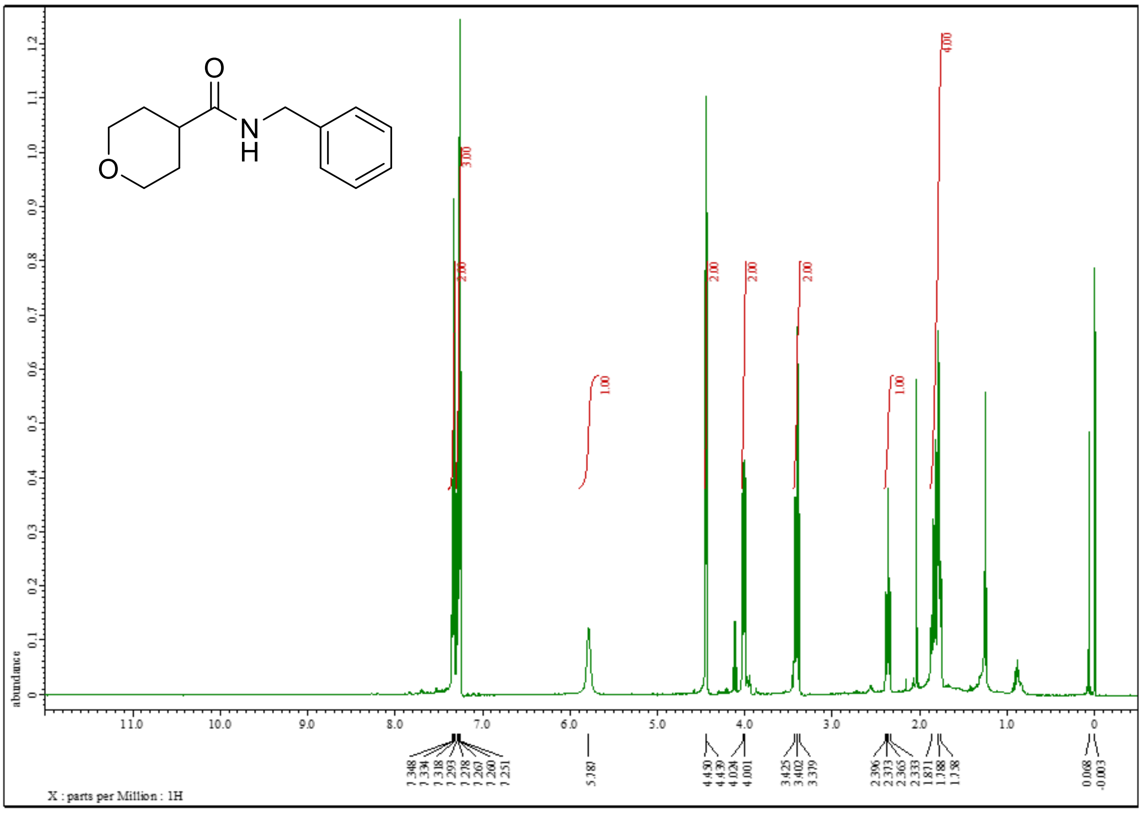

^{13}C NMR of spectrum of **7i** (125 MHz, $CDCl_3$)

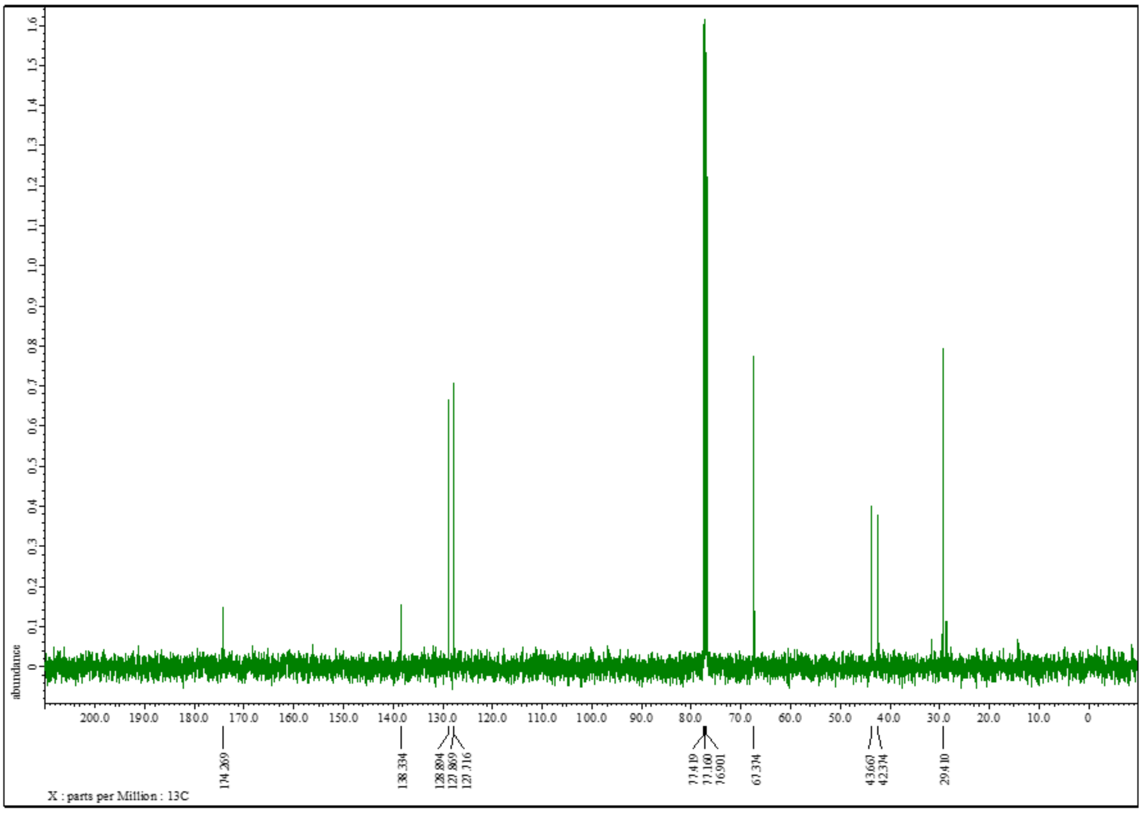

^{1}H NMR of spectrum of **7k** (500 MHz, $CDCl_3$)

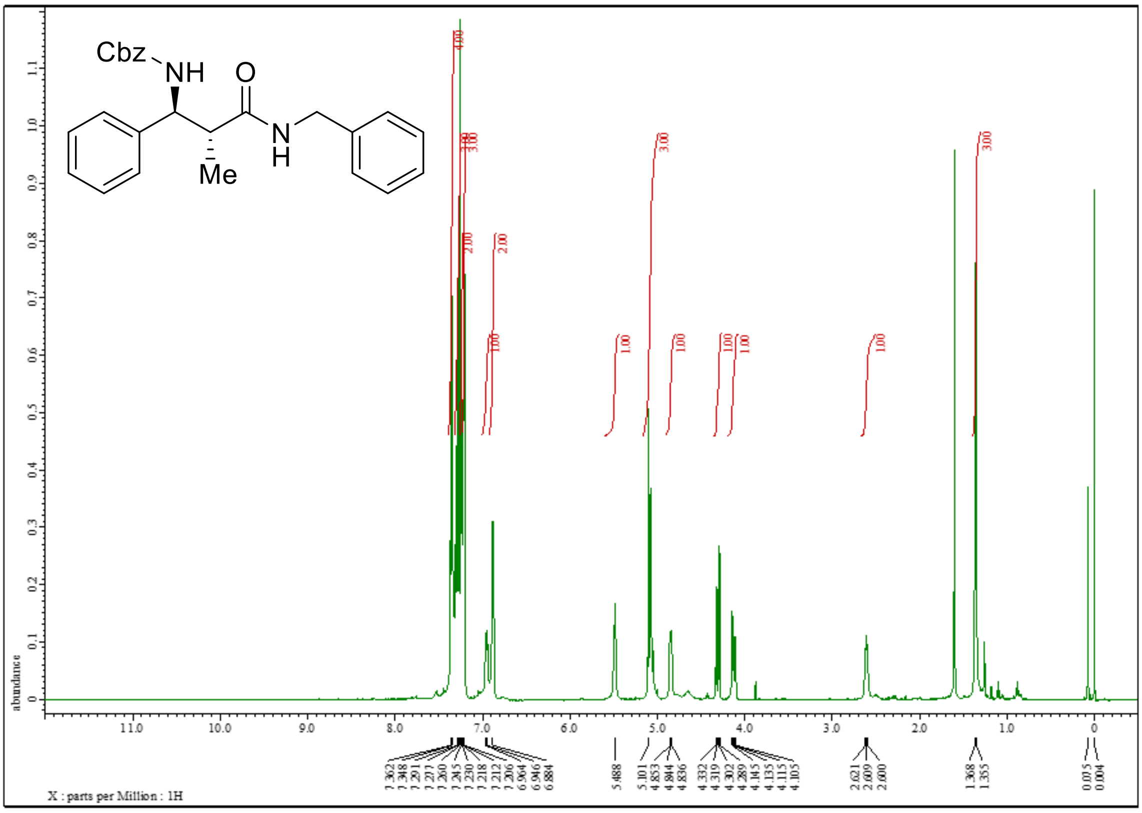

^{13}C NMR of spectrum of **7k** (125 MHz, $CDCl_3$)

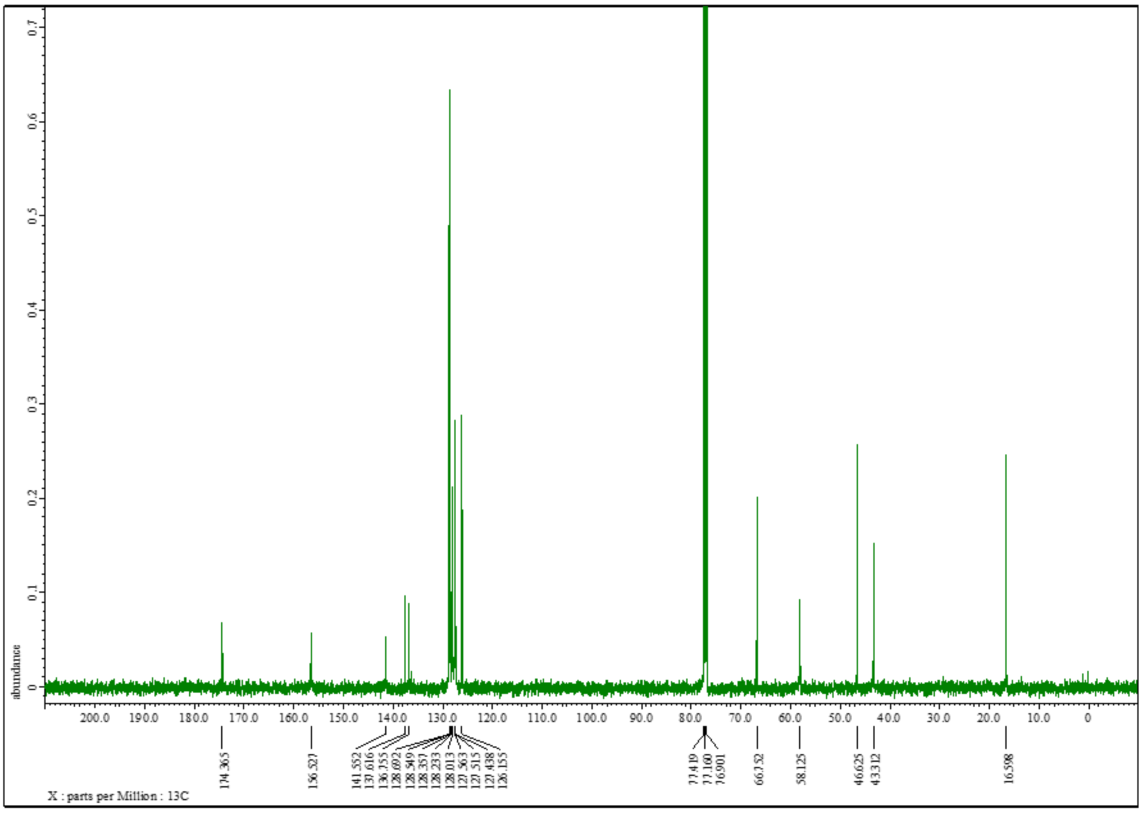

^{1}H NMR of spectrum of **7l** (500 MHz, $CDCl_3$)

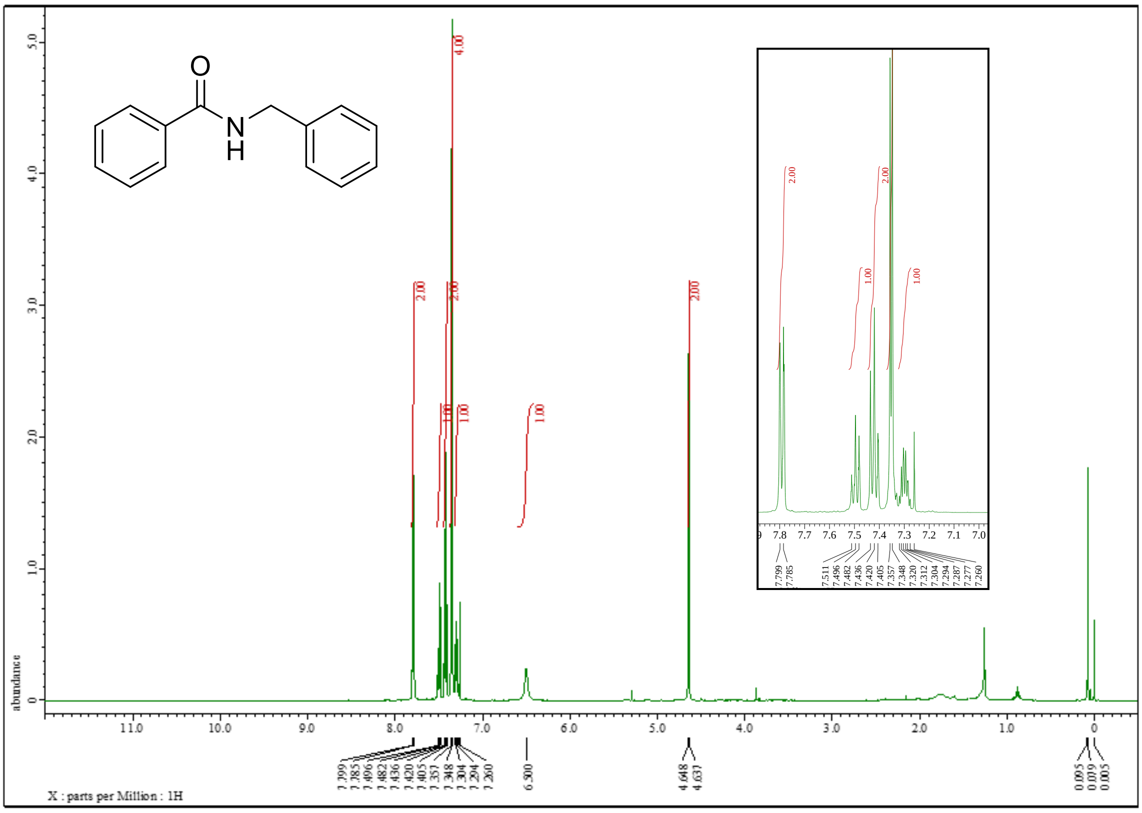

^{13}C NMR of spectrum of **7l** (125 MHz, $CDCl_3$)

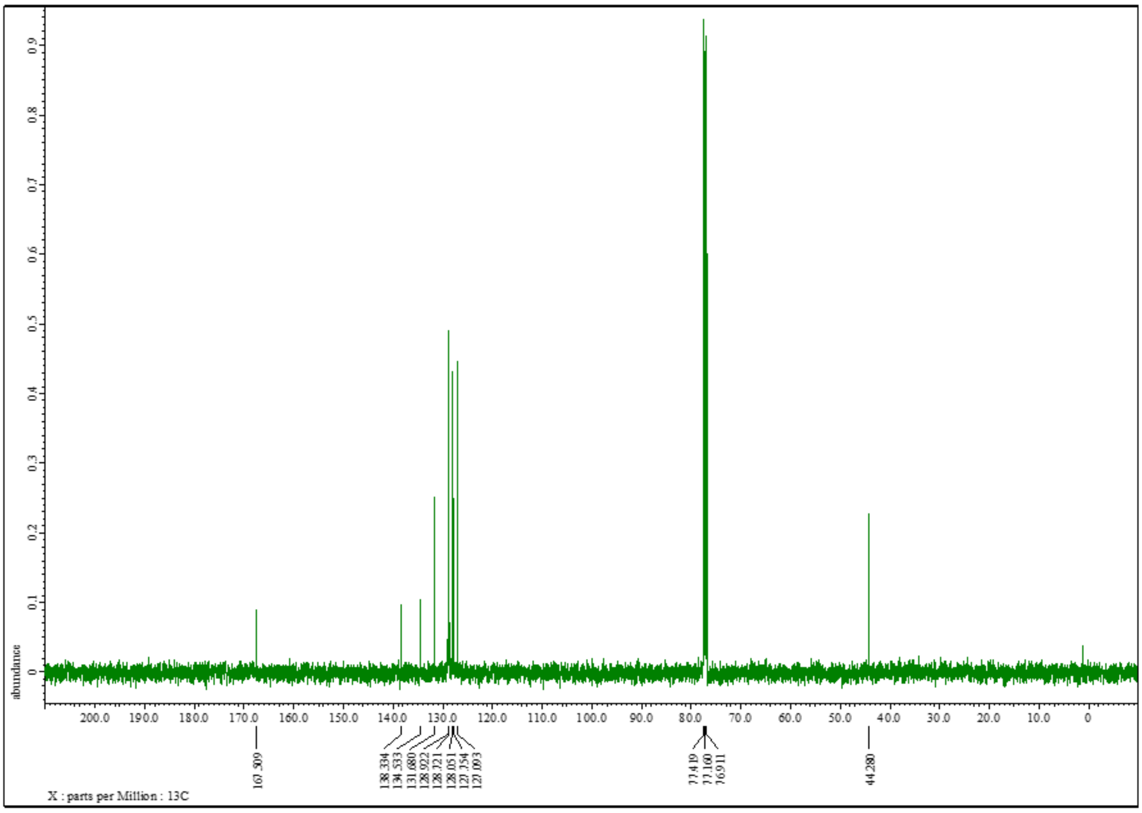

^{1}H NMR of spectrum of **7m** (500 MHz, $CDCl_3$)

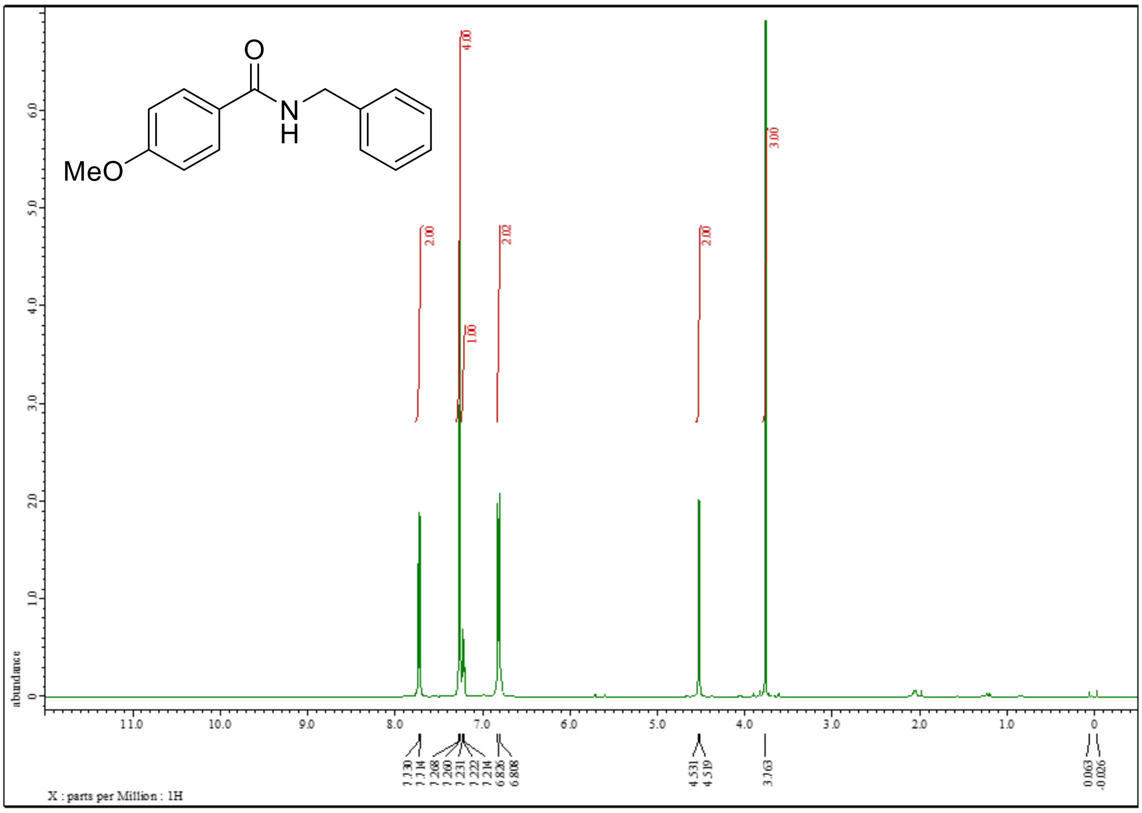

^{13}C NMR of spectrum of **7m** (125 MHz, $CDCl_3$)

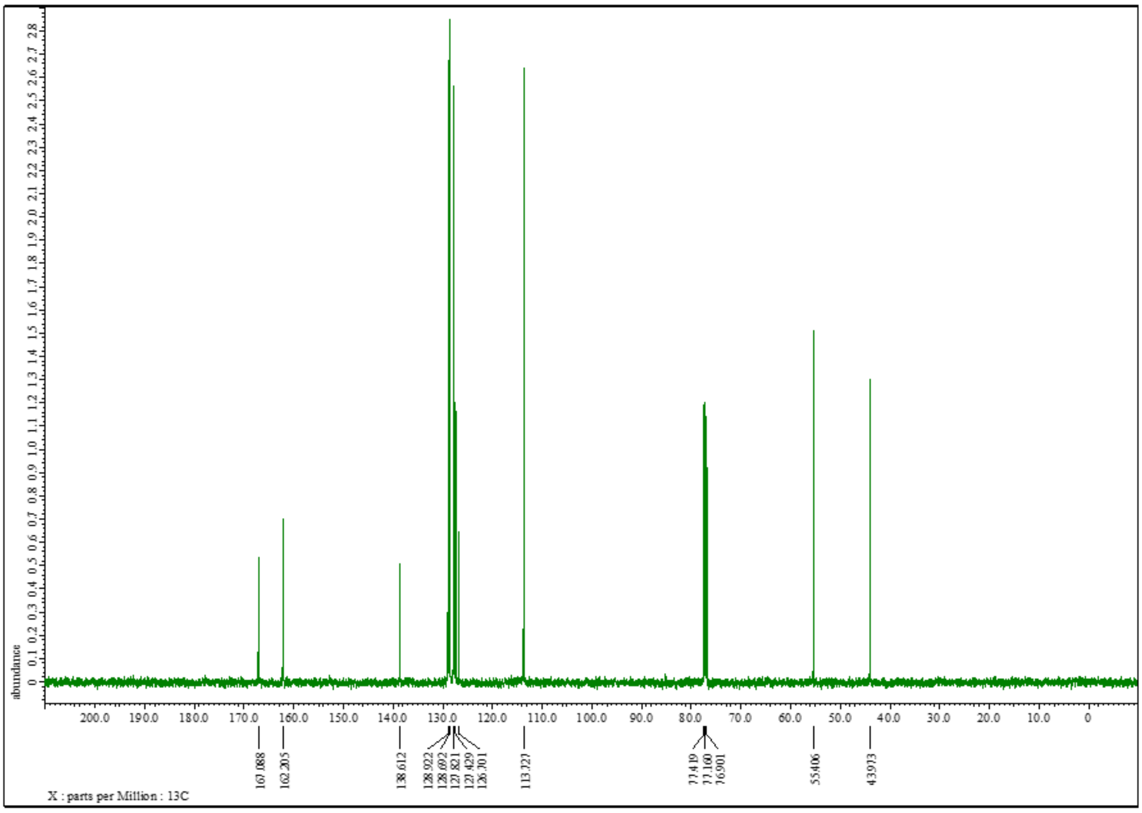

^{1}H NMR spectrum of **7n** (500 MHz, $CDCl_3$)

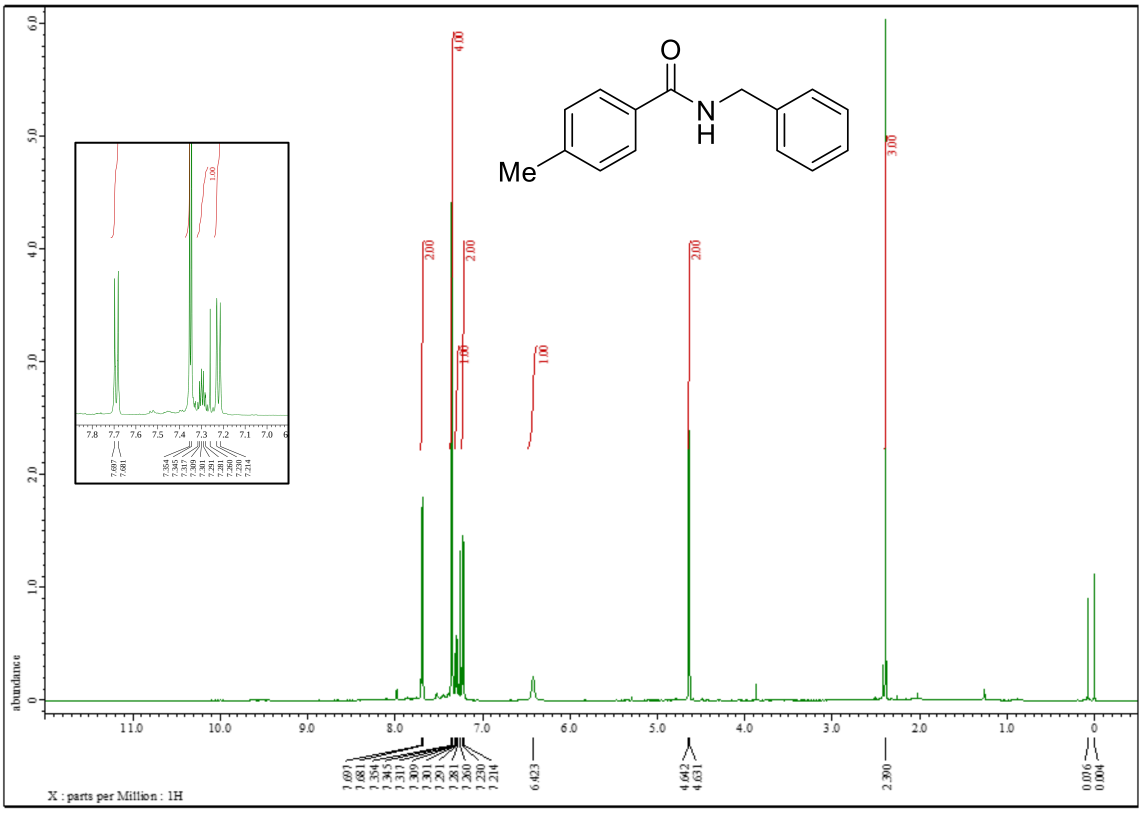

^{13}C NMR spectrum of **7n** (125 MHz, $CDCl_3$)

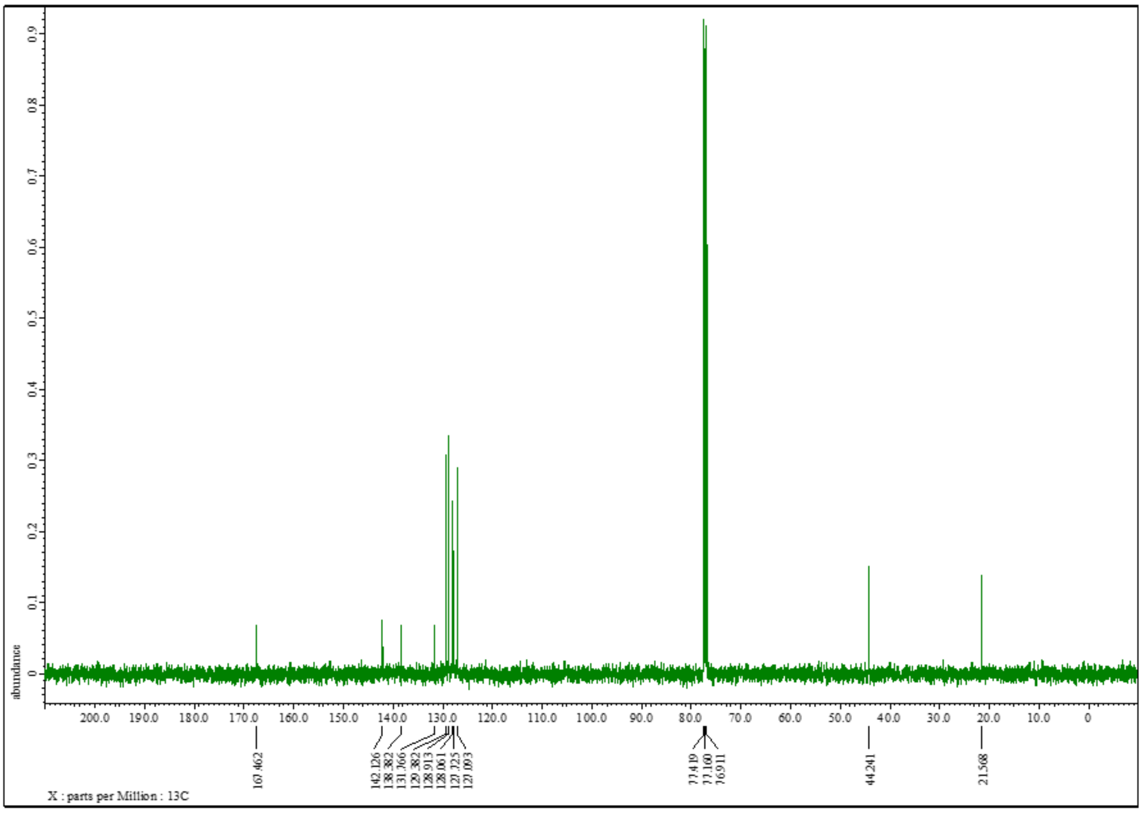

^{1}H NMR of spectrum of **7o** (500 MHz, $CDCl_3$)

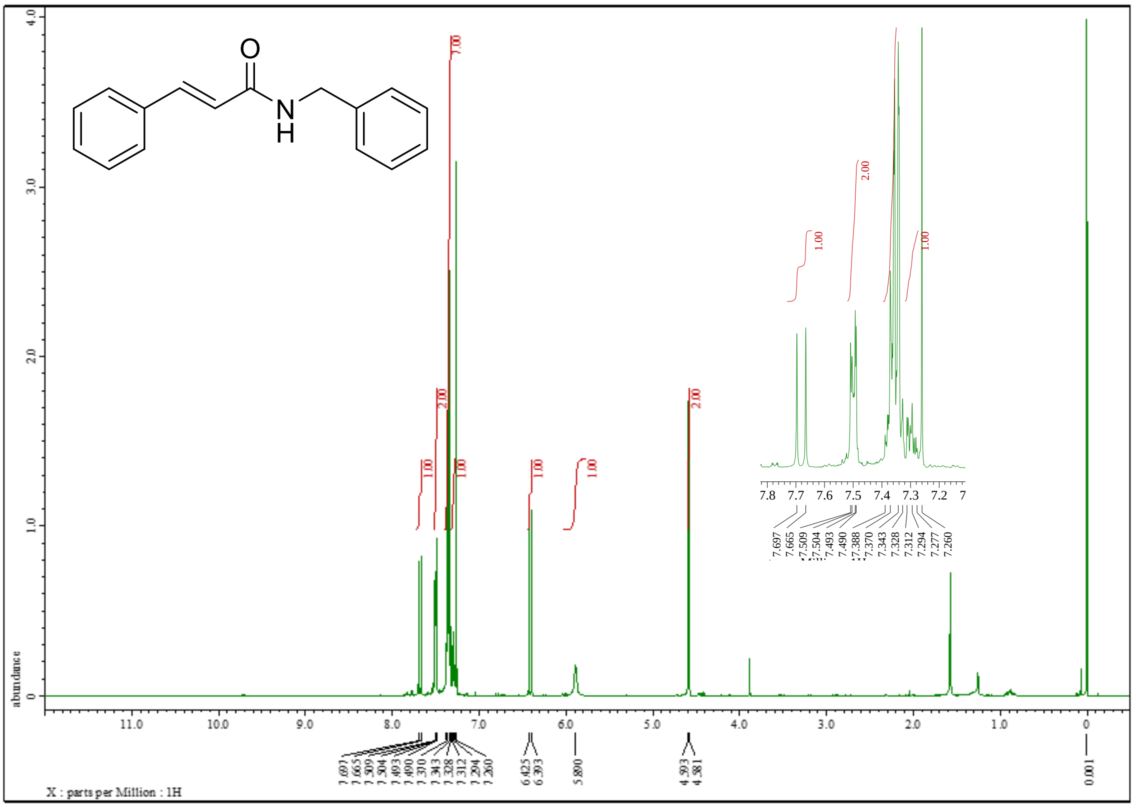

^{13}C NMR of spectrum of **7o** (125 MHz, $CDCl_3$)

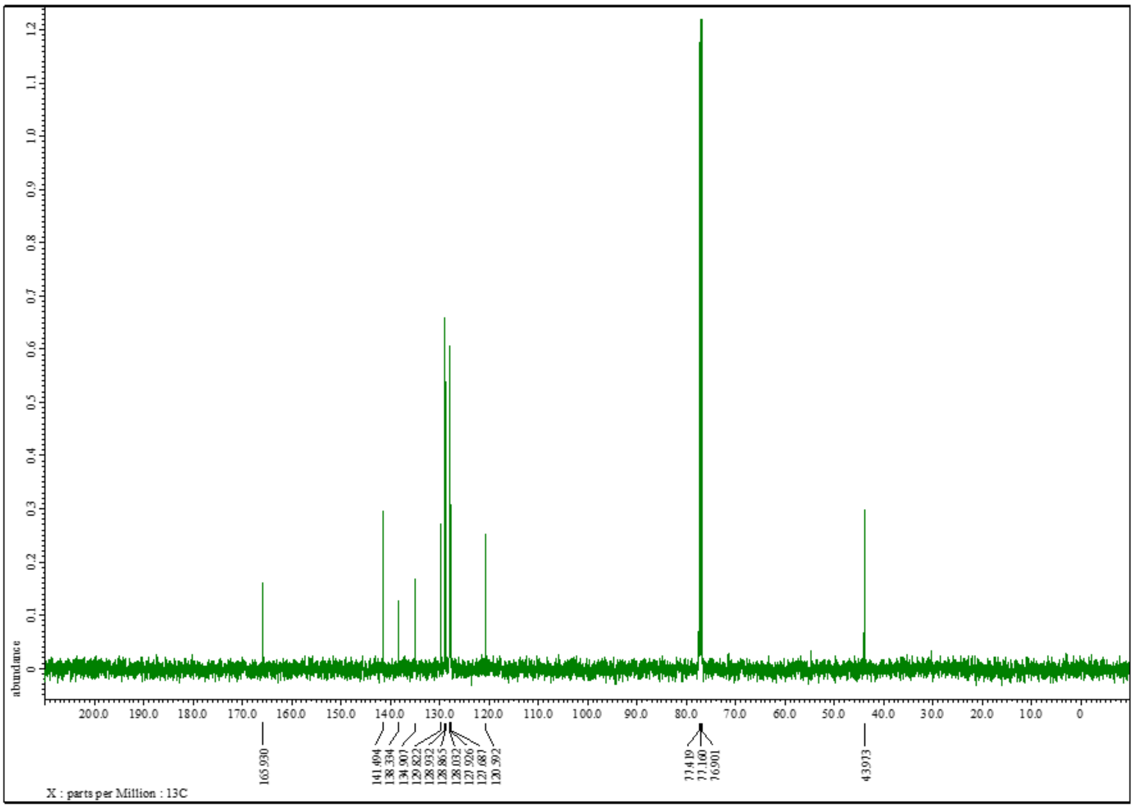

^{1}H NMR of spectrum of **8a** (500 MHz, $CDCl_3$)

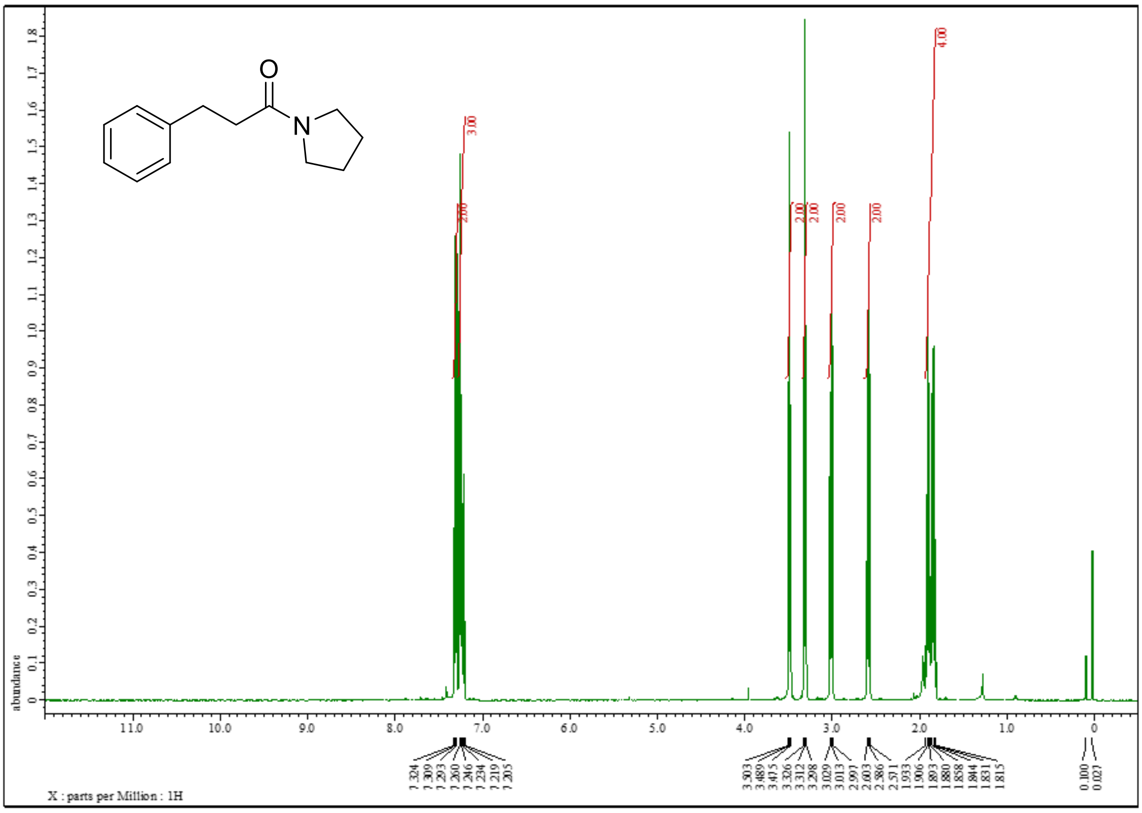

^{13}C NMR of spectrum of **8a** (125 MHz, $CDCl_3$)

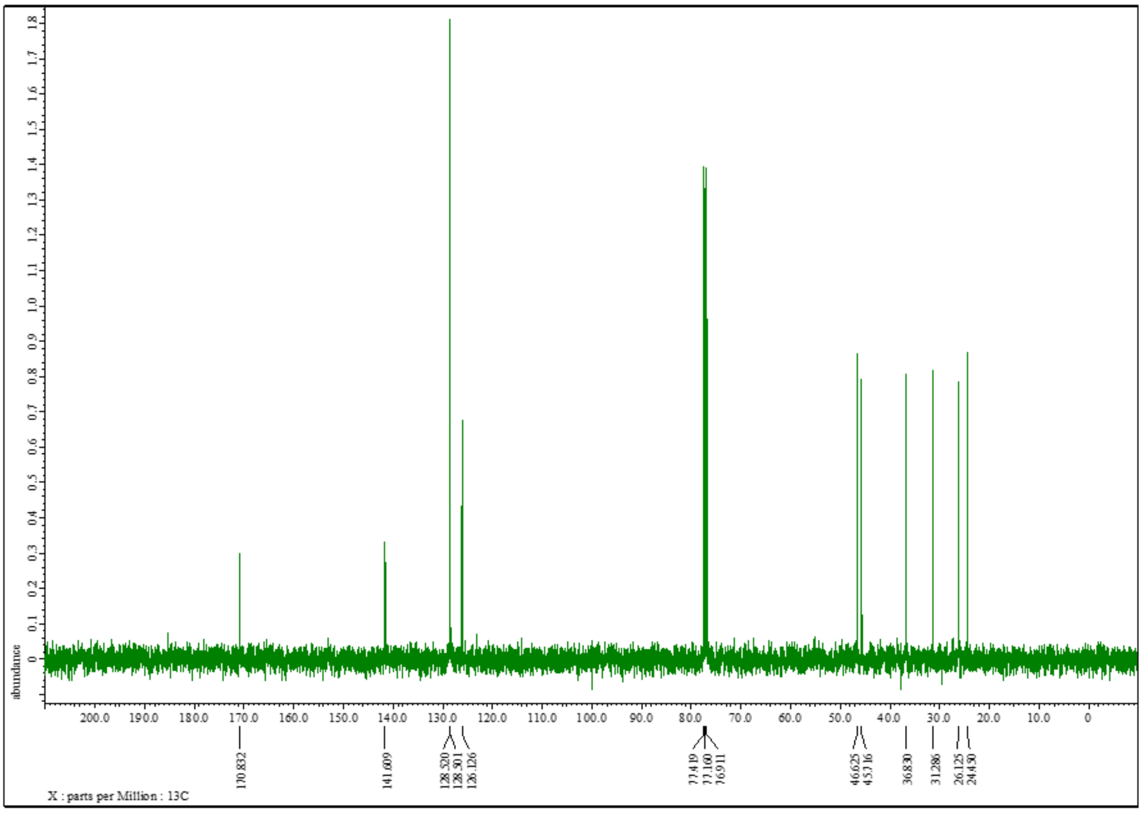

^{1}H NMR of spectrum of **8b** (500 MHz, $CDCl_3$)

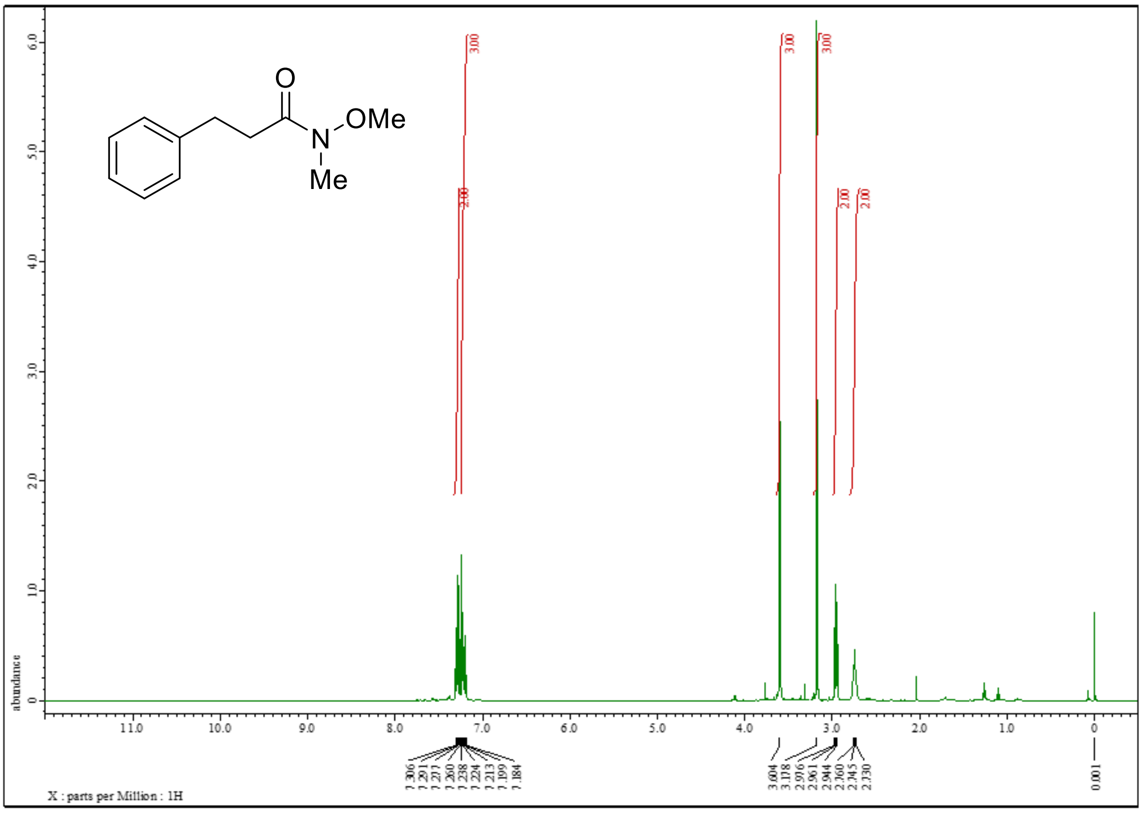

^{13}C NMR of spectrum of **8b** (125 MHz, $CDCl_3$)

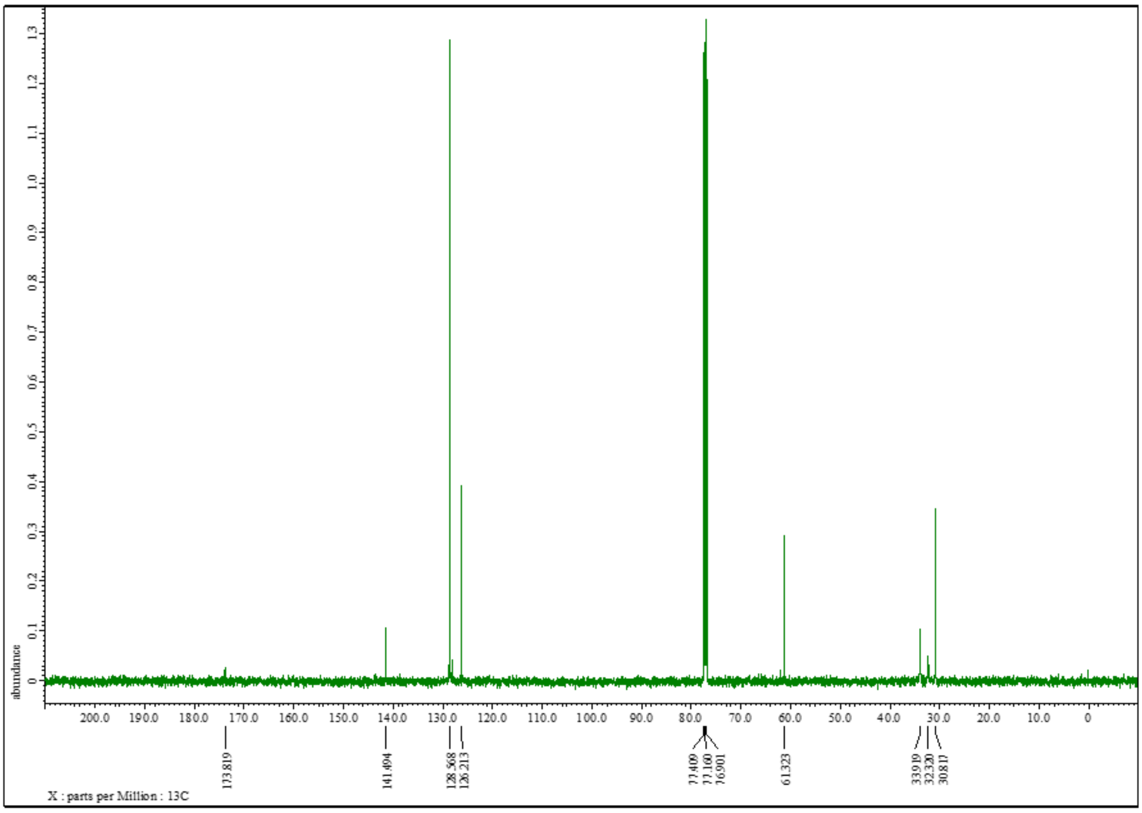

^{1}H NMR of spectrum of **8c** (500 MHz, $CDCl_3$)

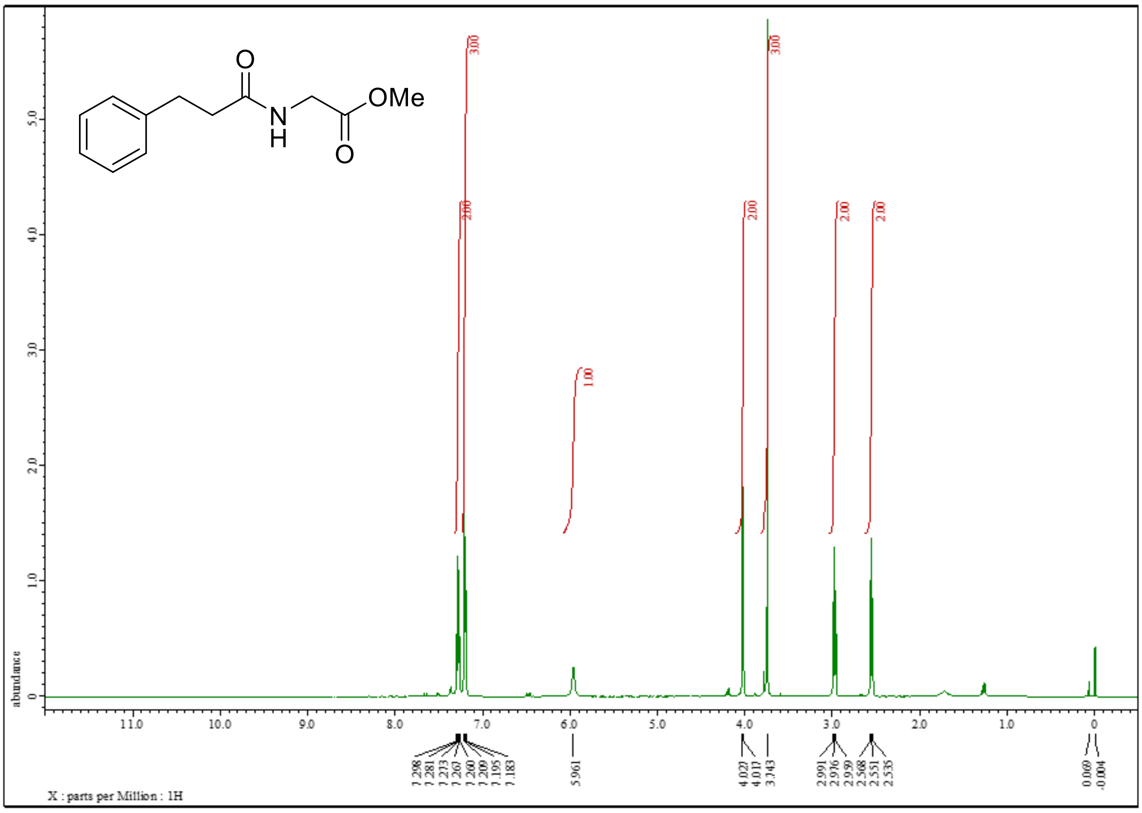

^{13}C NMR of spectrum of **8c** (125 MHz, $CDCl_3$)

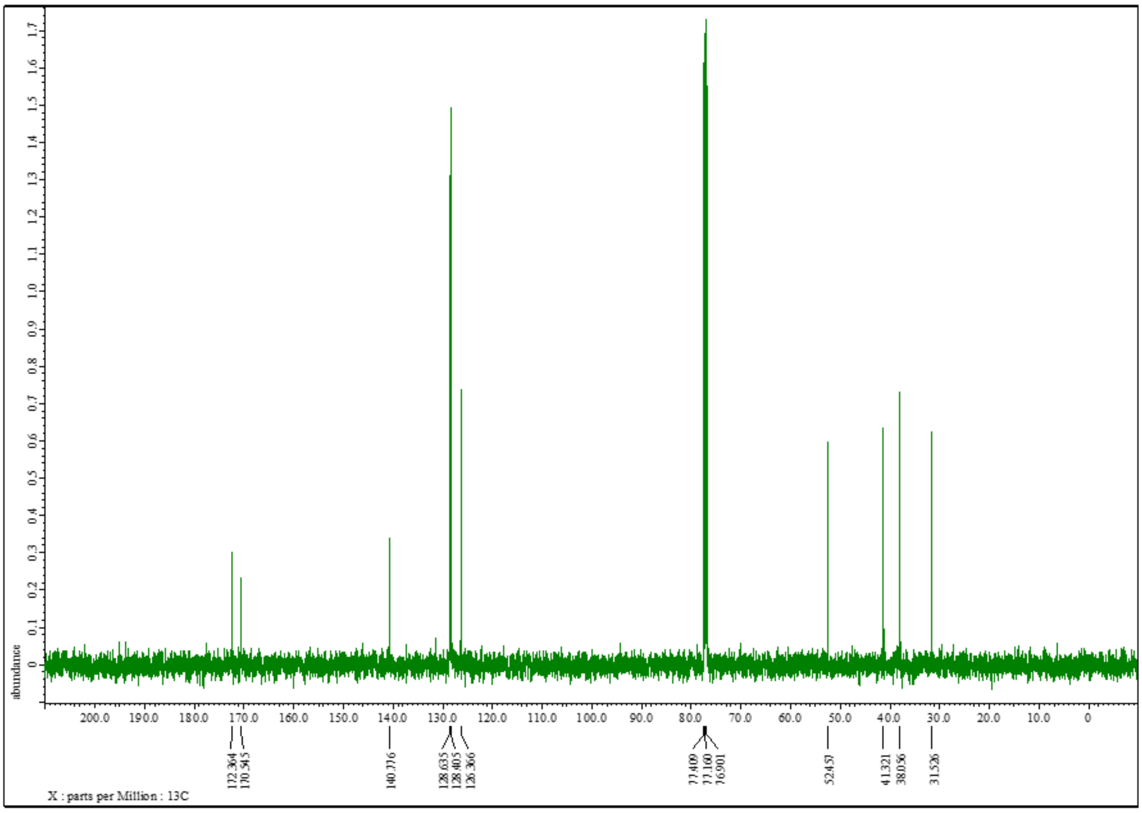

^{1}H NMR of spectrum of **8d** (500 MHz, $CDCl_3$)

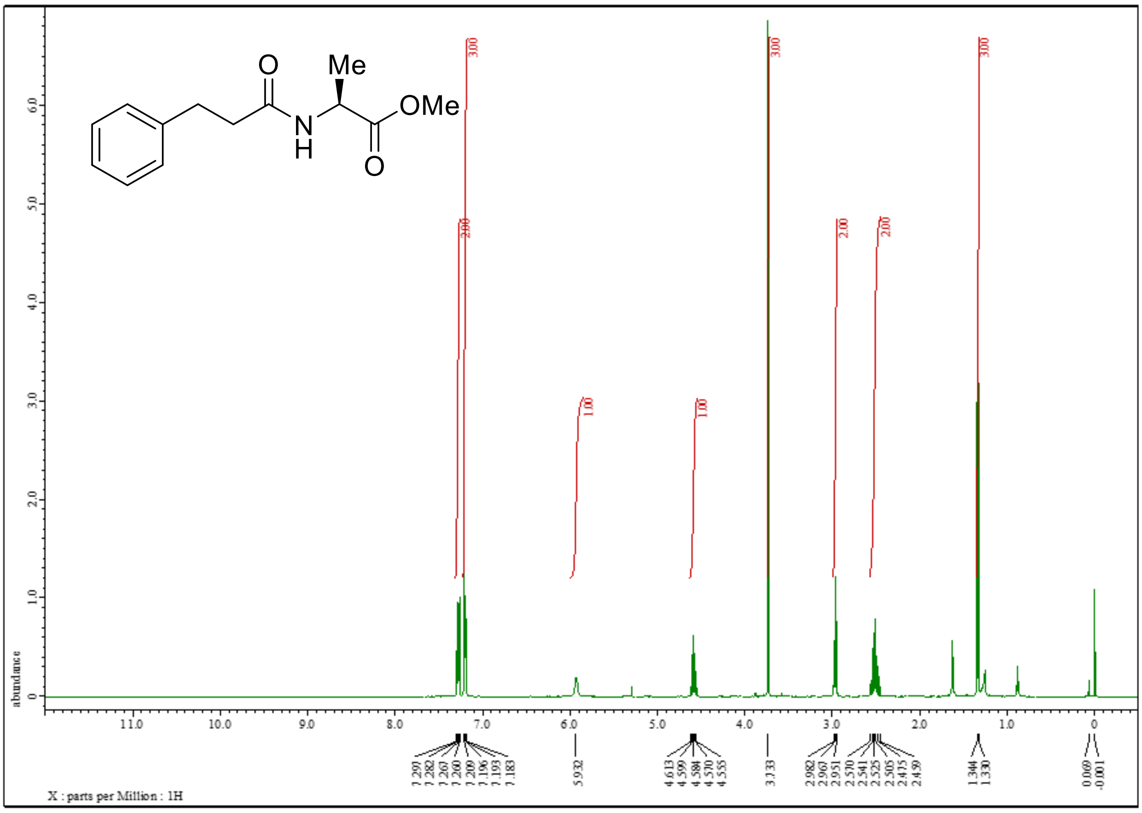

^{13}C NMR of spectrum of **8d** (125 MHz, $CDCl_3$)

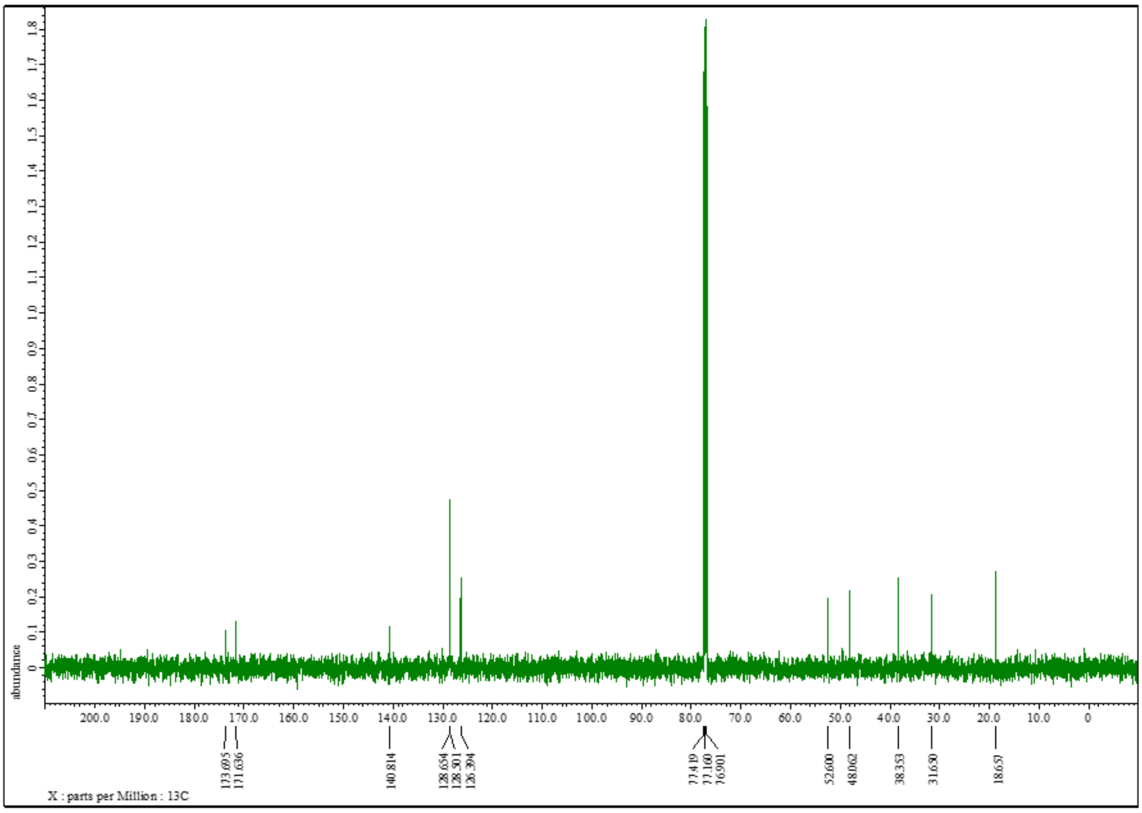

^{1}H NMR of spectrum of **8e** (500 MHz, $CDCl_3$)

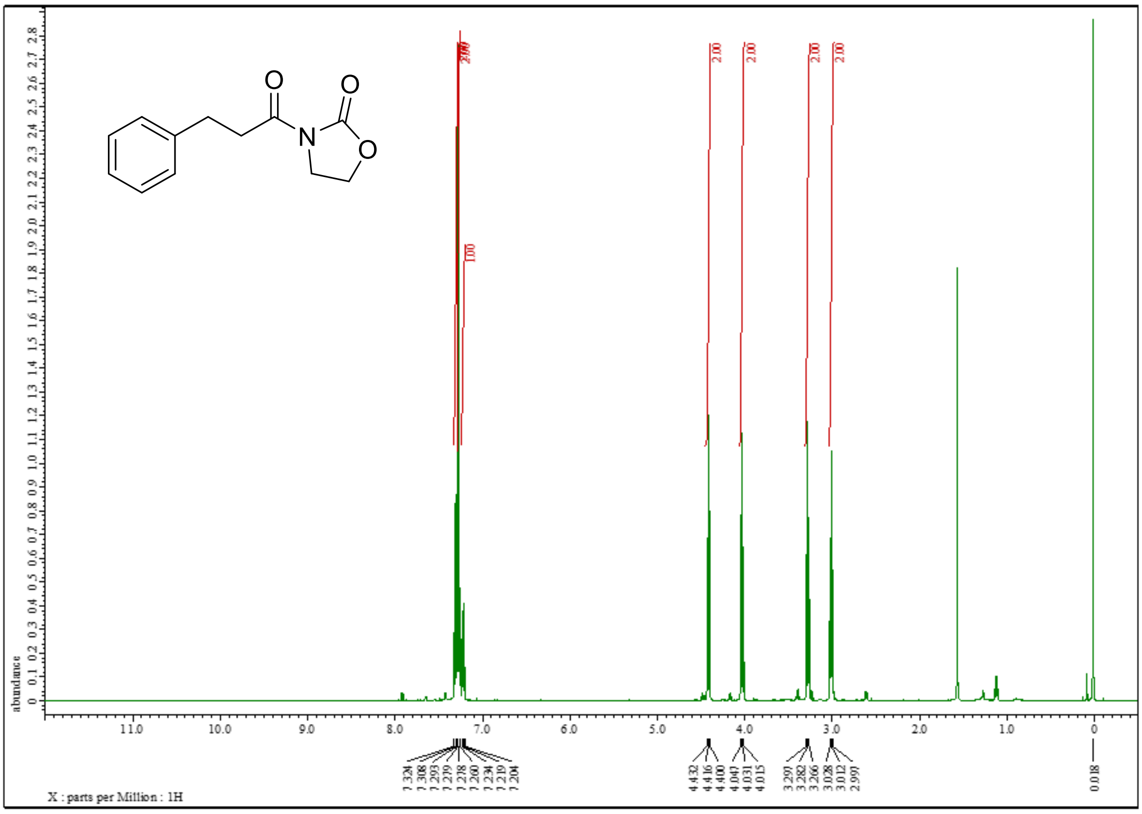

^{13}C NMR of spectrum of **8e** (125 MHz, $CDCl_3$)

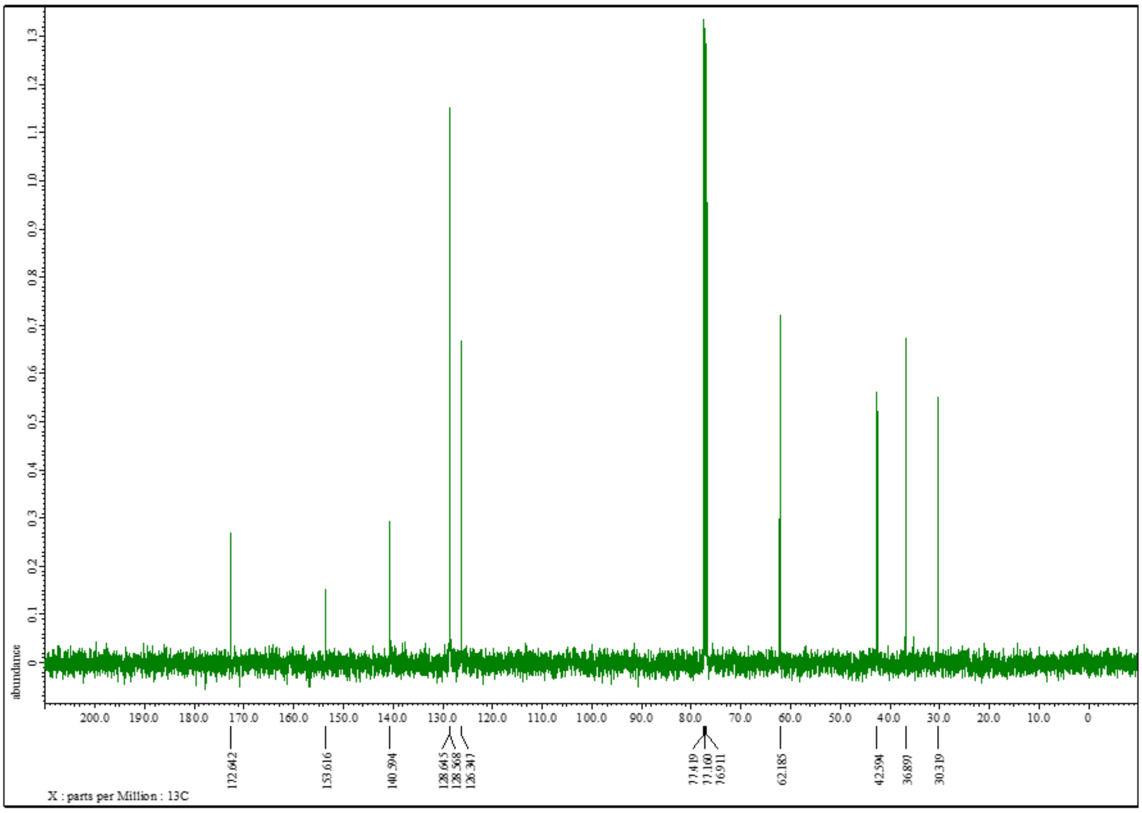

^{1}H NMR of spectrum of **8f** (500 MHz, $CDCl_3$)

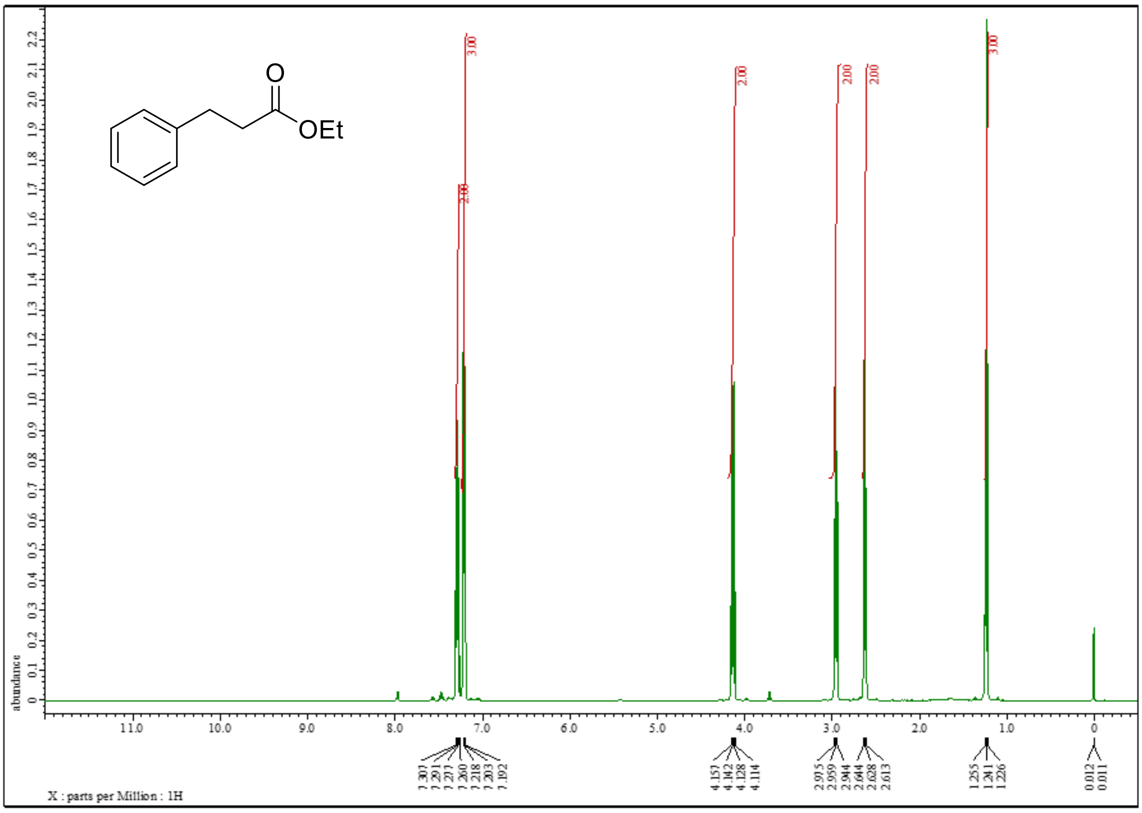

^{13}C NMR of spectrum of **8f** (125 MHz, $CDCl_3$)

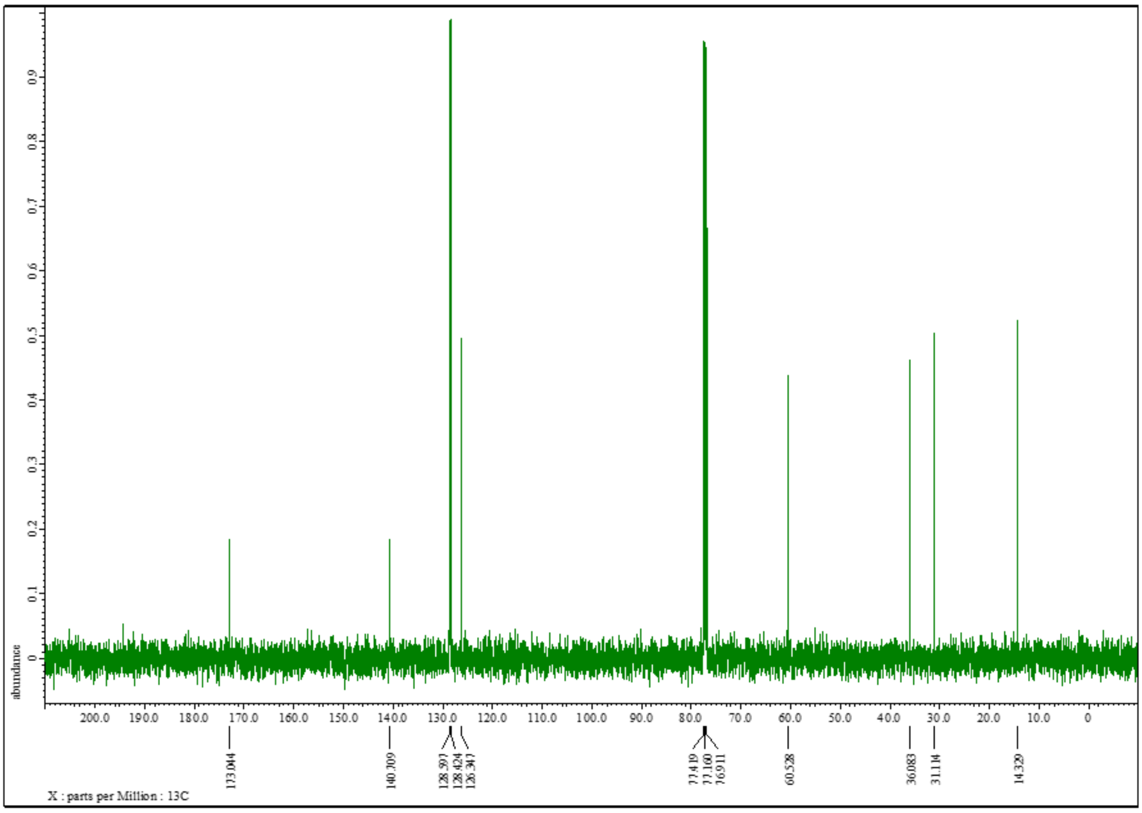

^{1}H NMR of spectrum of **8g** (500 MHz, $CDCl_3$)

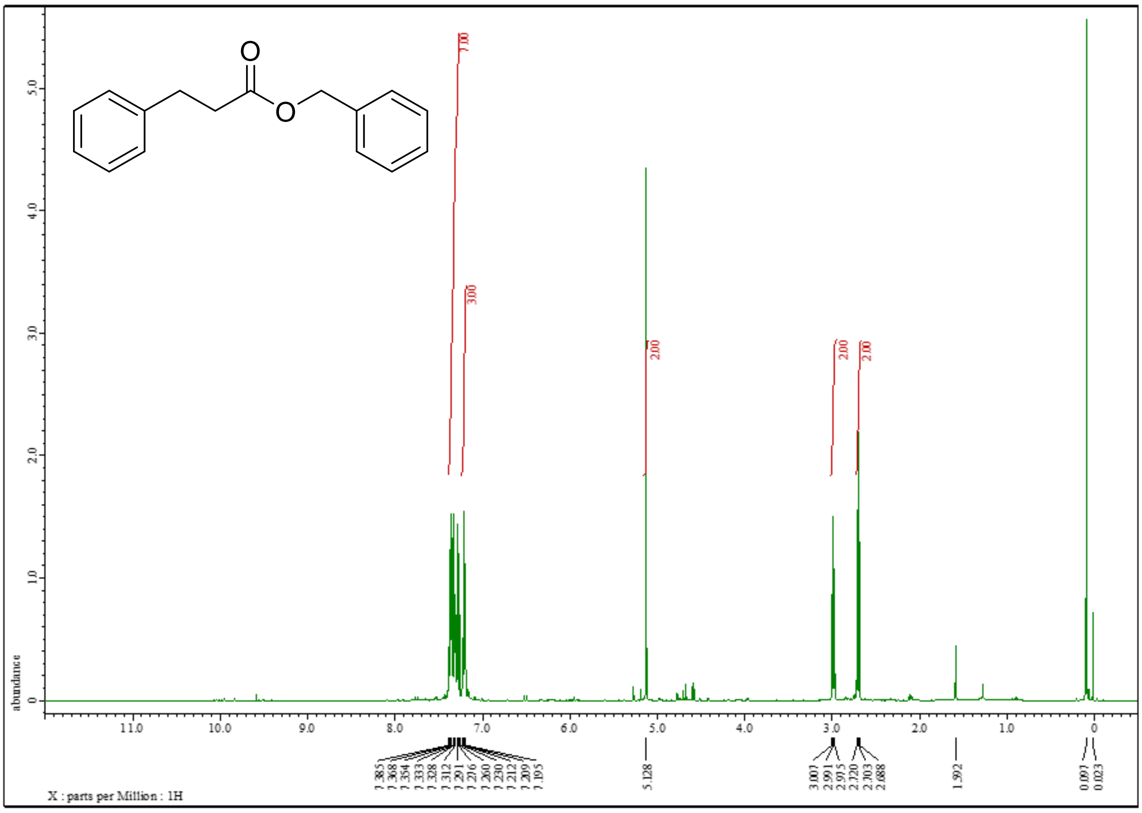

^{13}C NMR of spectrum of **8g** (125 MHz, $CDCl_3$)

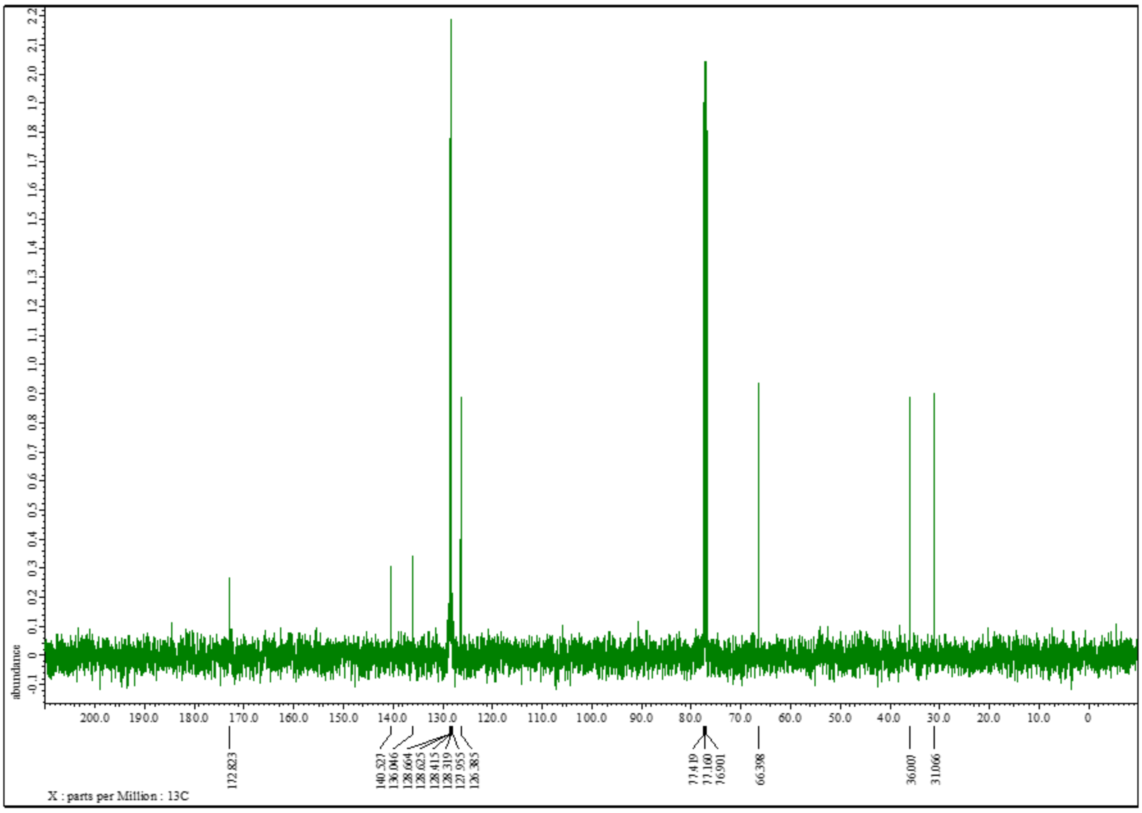

^{1}H NMR of spectrum of **8h** (500 MHz, $CDCl_3$)

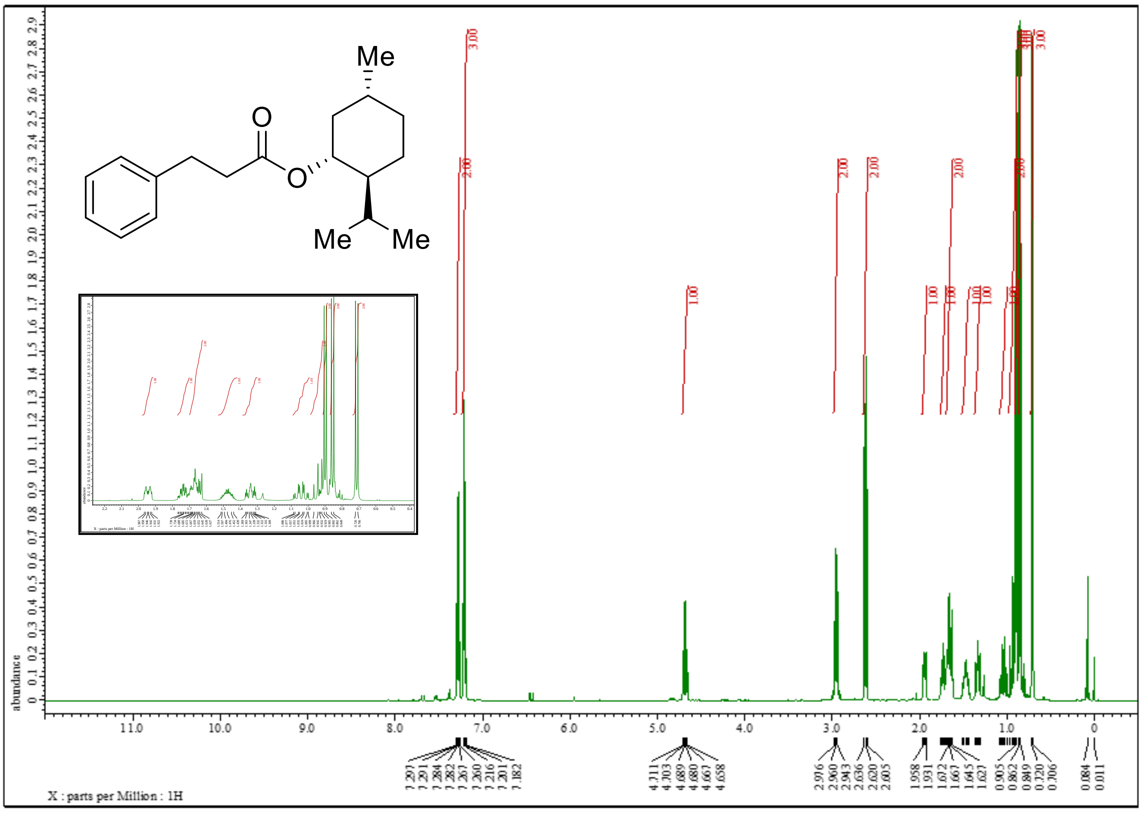

^{13}C NMR of spectrum of **8h** (125 MHz, $CDCl_3$)

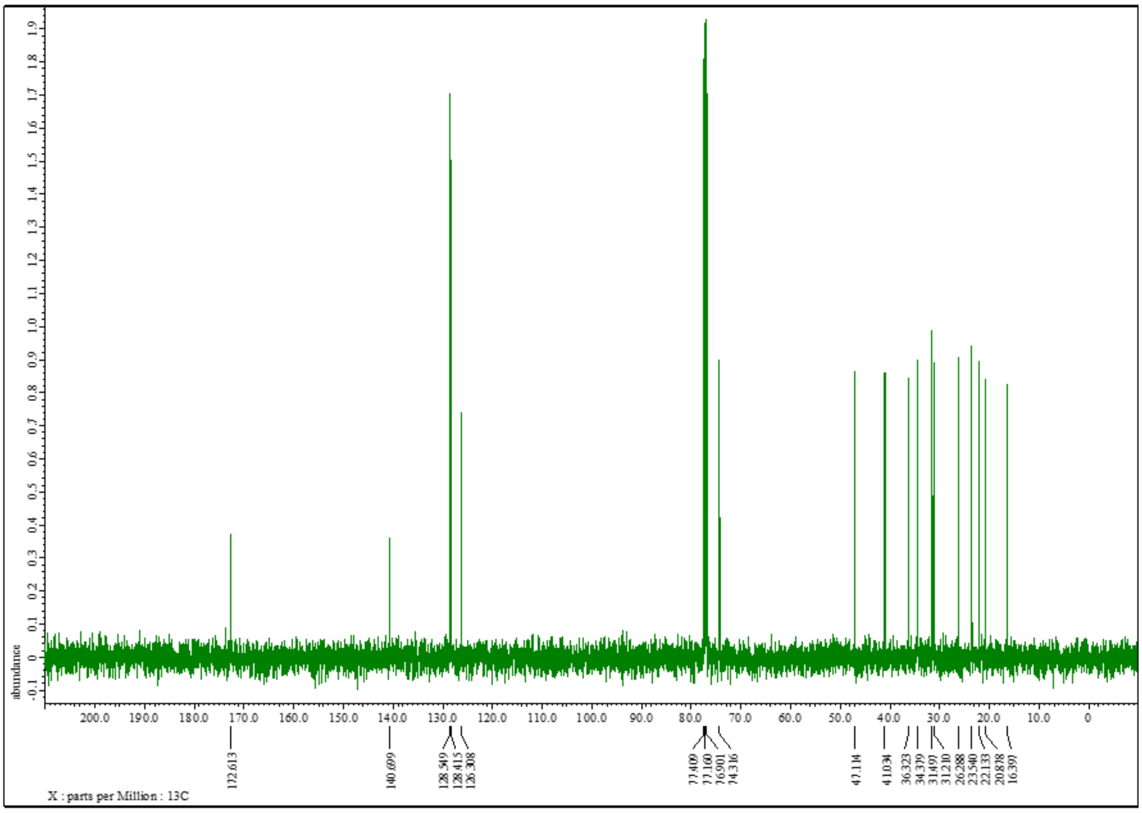

^{1}H NMR of spectrum of **8i** (500 MHz, $CDCl_3$)

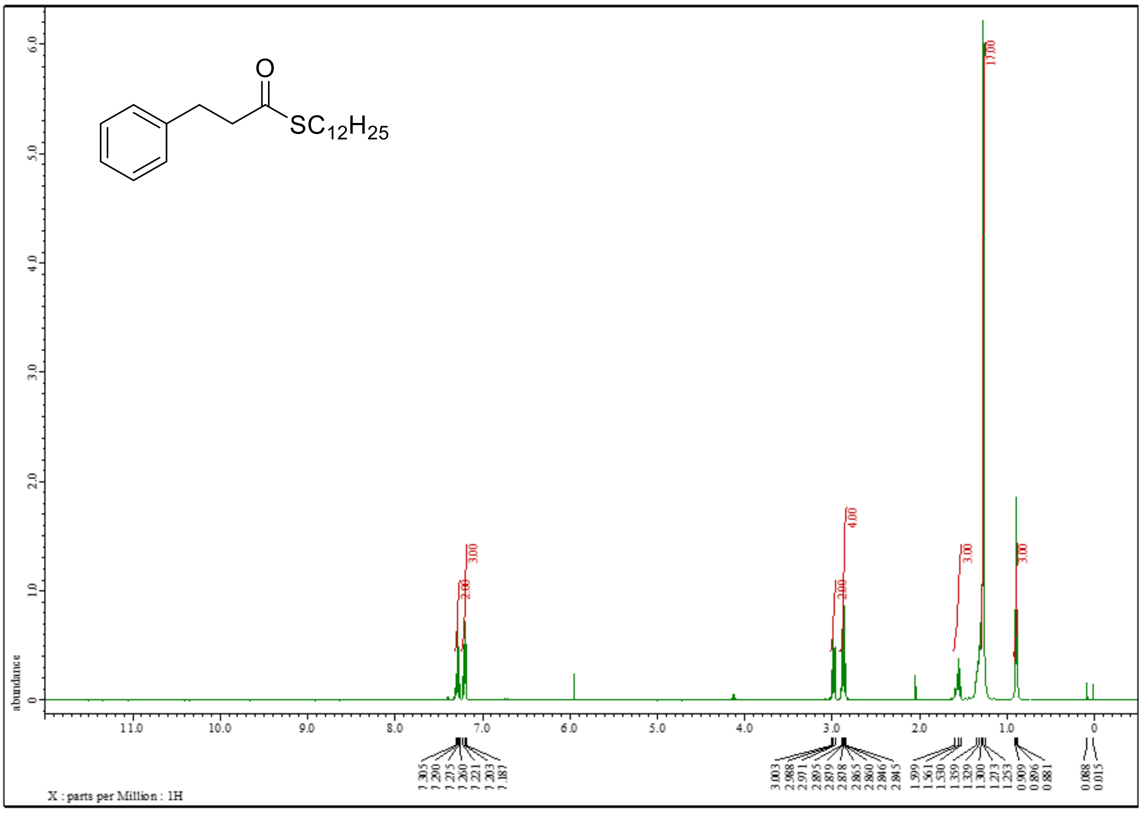

^{13}C NMR of spectrum of **8i** (125 MHz, $CDCl_3$)

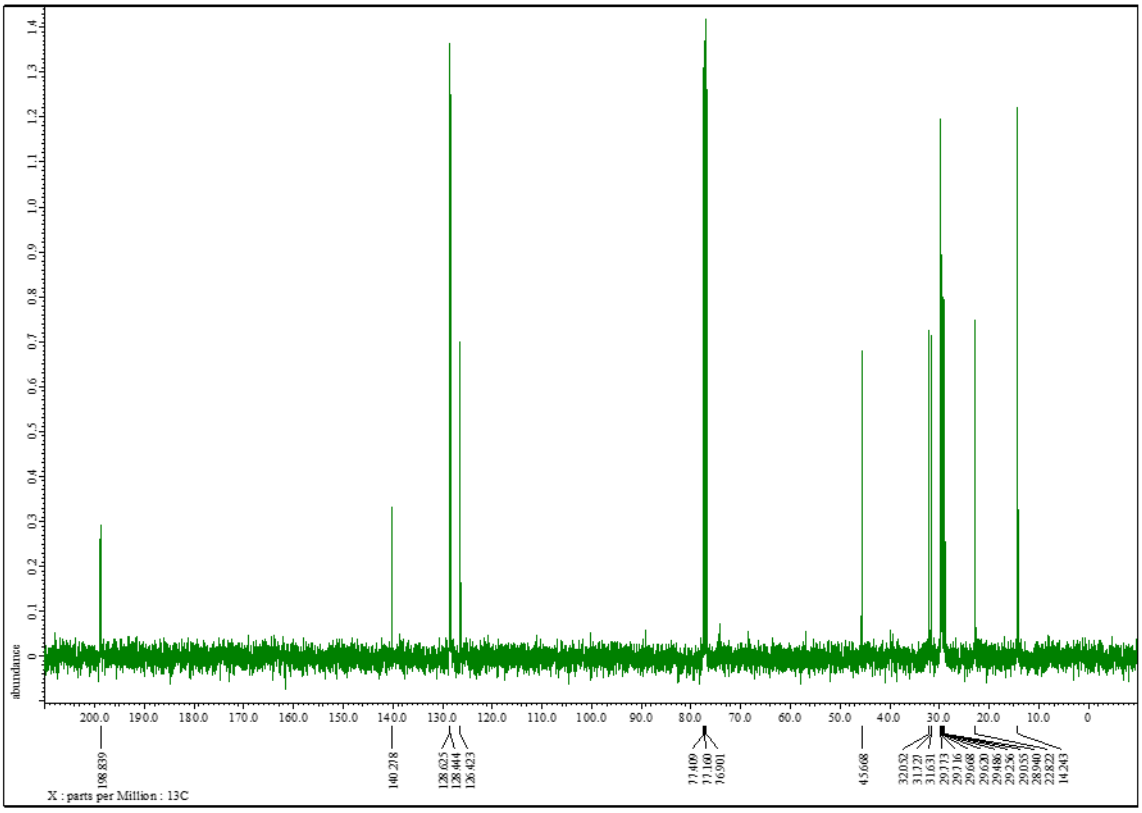

^{1}H NMR of spectrum of **10** (500 MHz, $CDCl_3$)

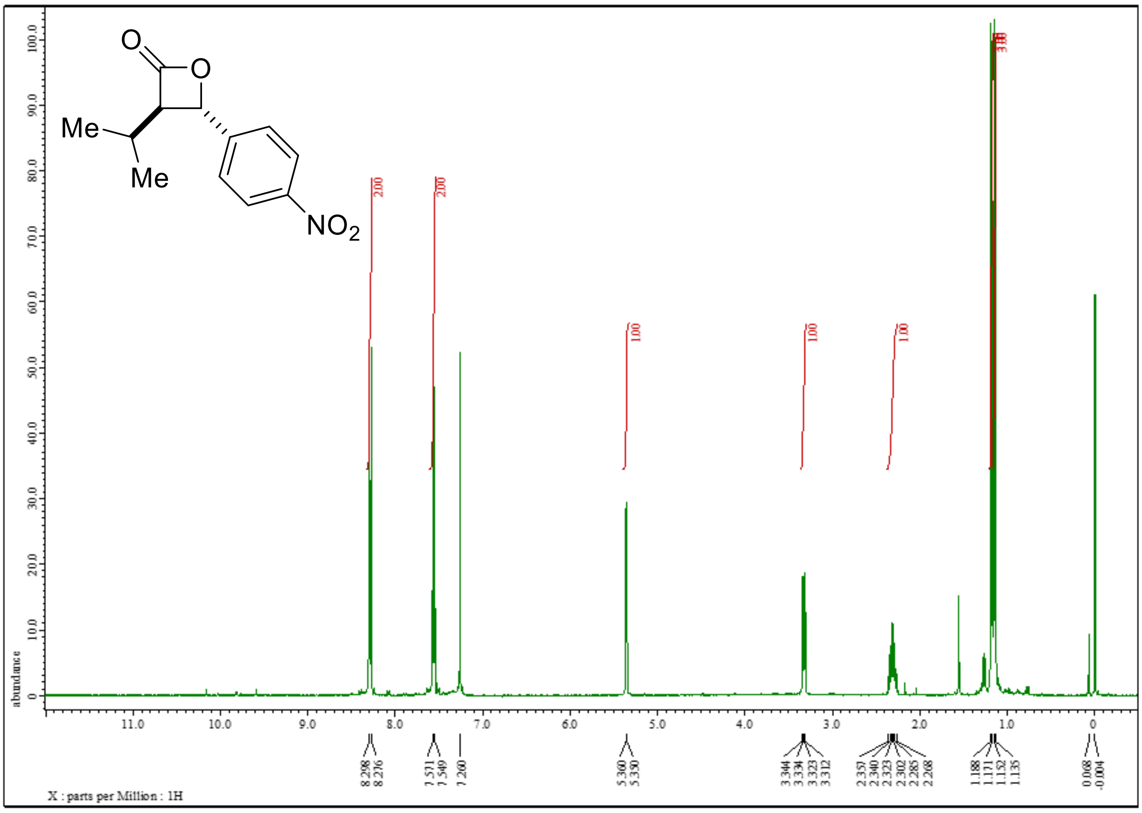

^{13}C NMR of spectrum of **10** (125 MHz, $CDCl_3$)

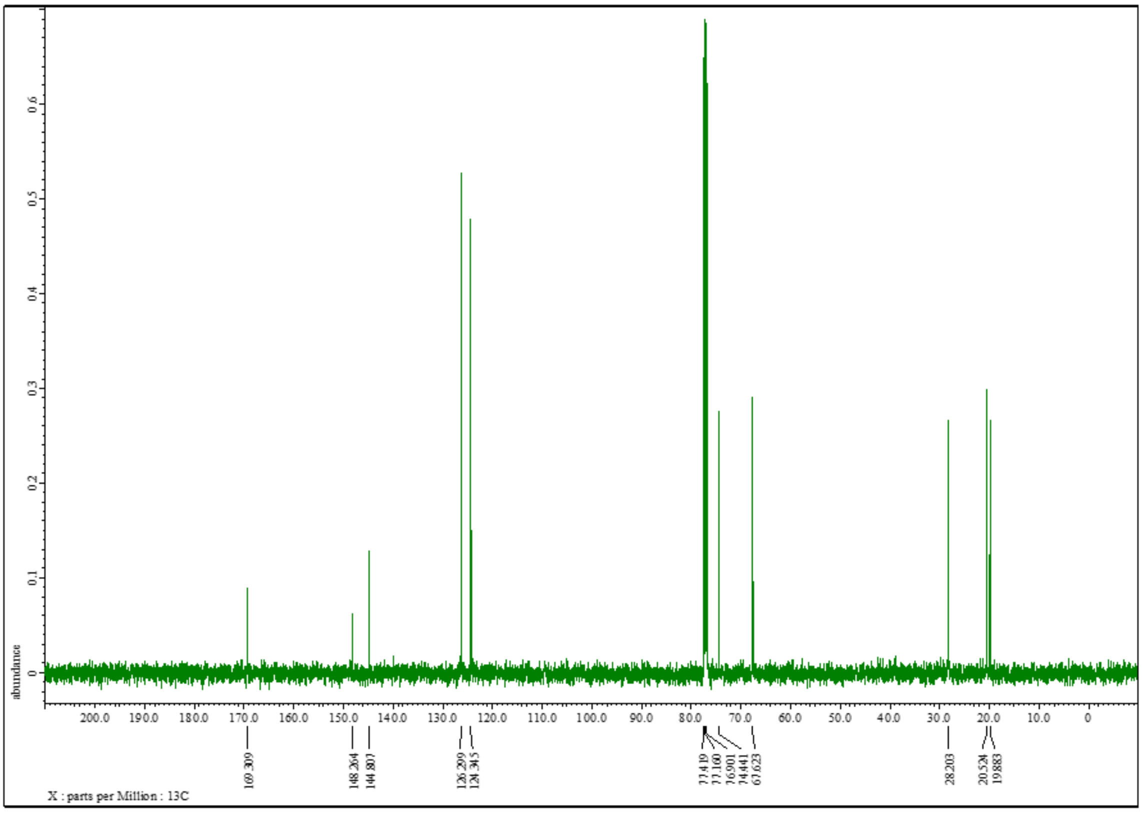

^{1}H NMR of spectrum of **11a** (500 MHz, $CDCl_3$)

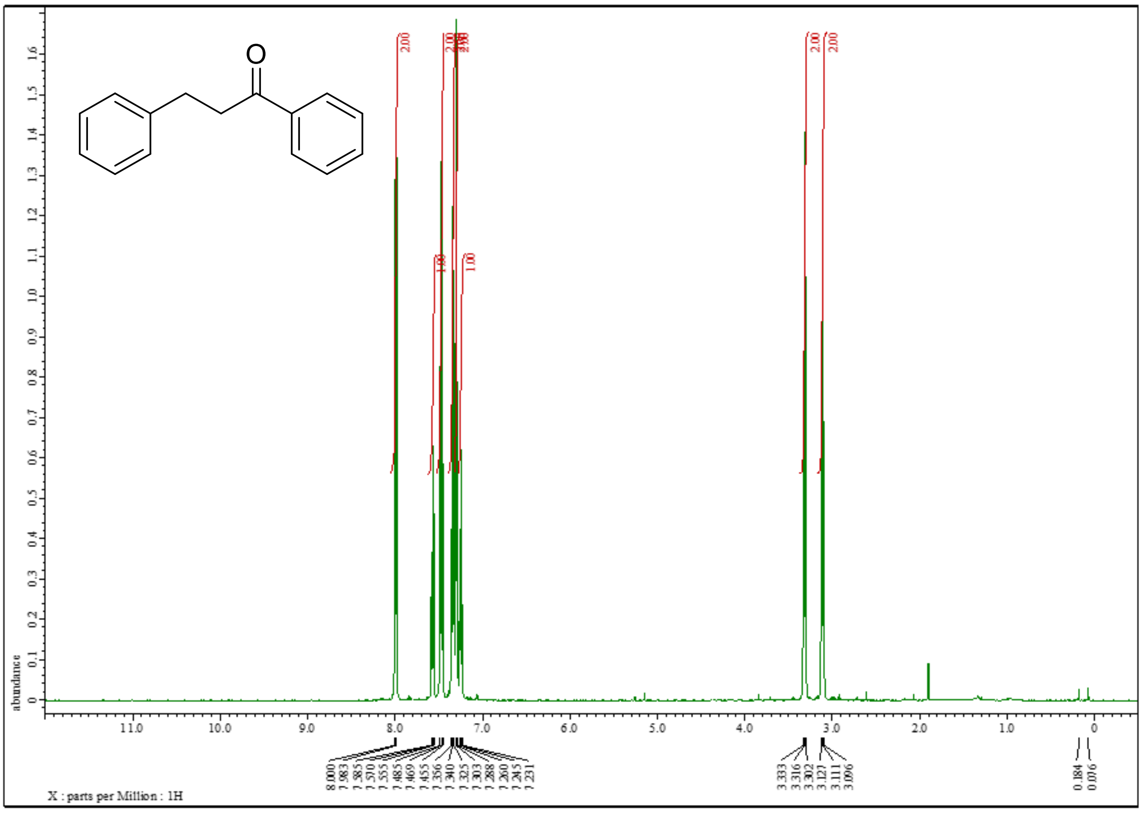

^{13}C NMR of spectrum of **11a** (125 MHz, $CDCl_3$)

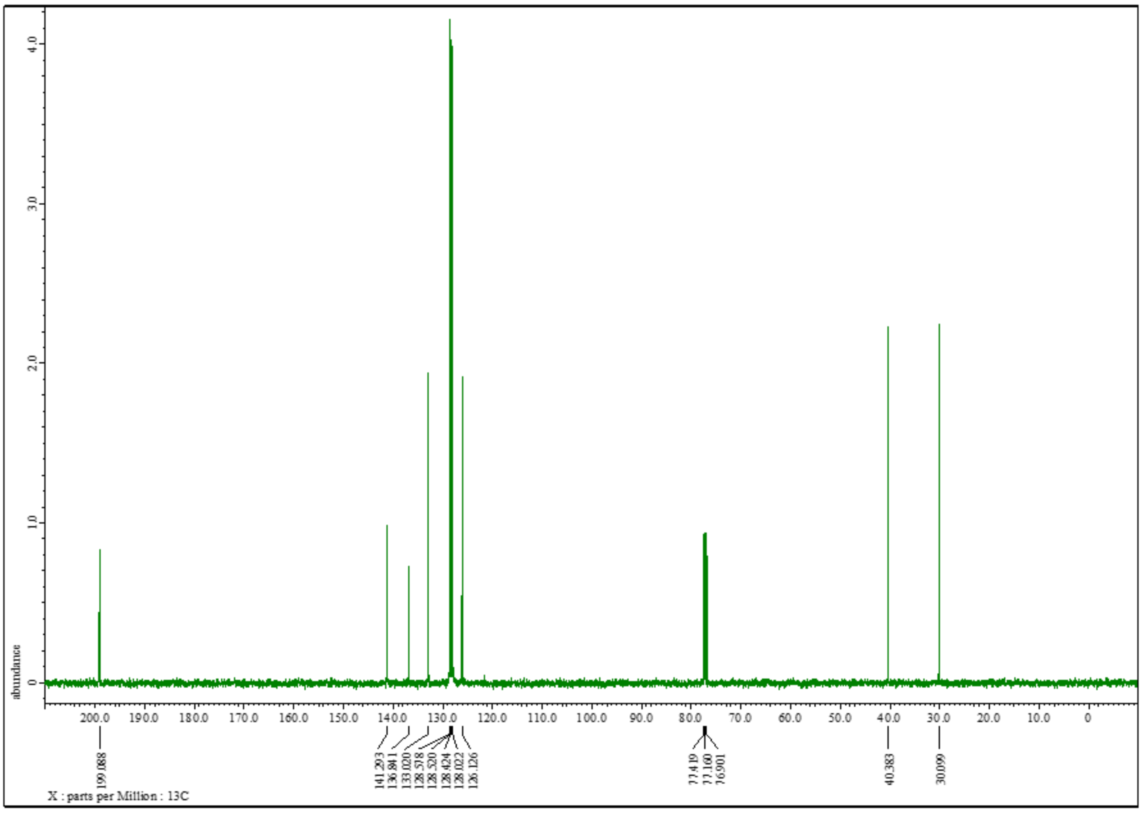

^{1}H NMR of spectrum of **11b** (500 MHz, $CDCl_3$)

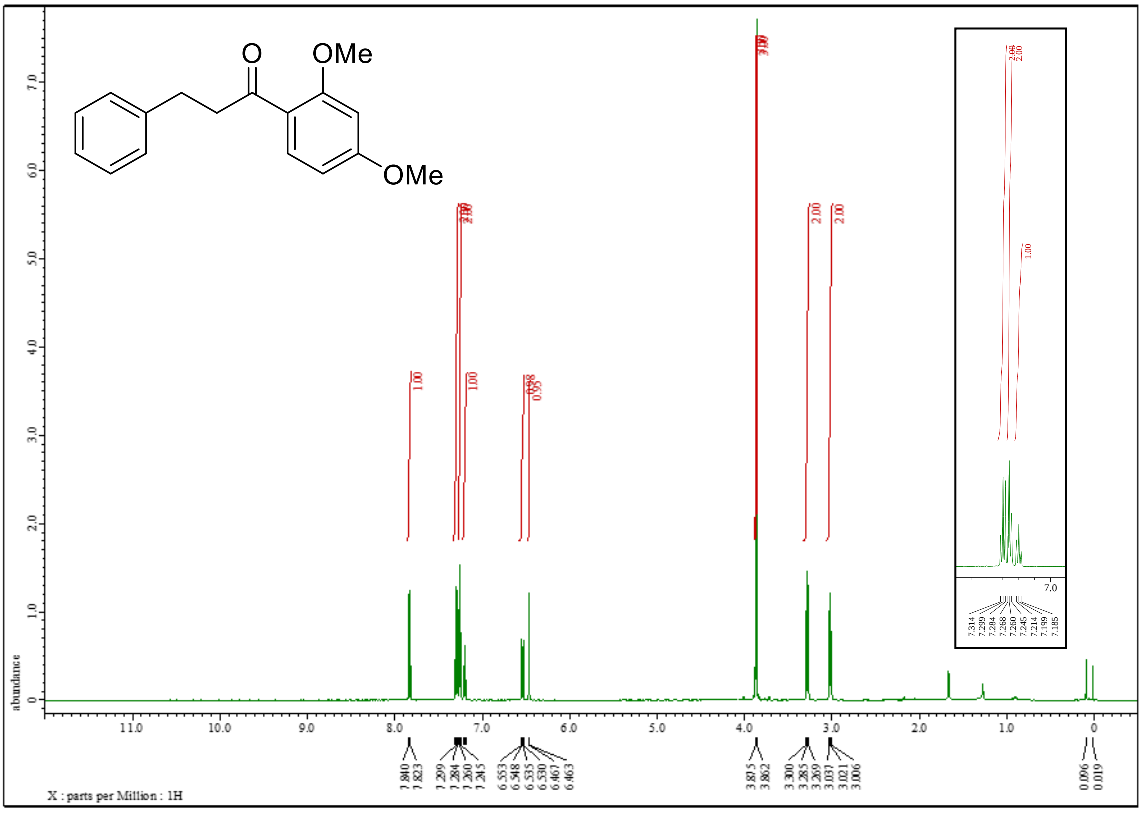

^{13}C NMR of spectrum of **11b** (125 MHz, $CDCl_3$)

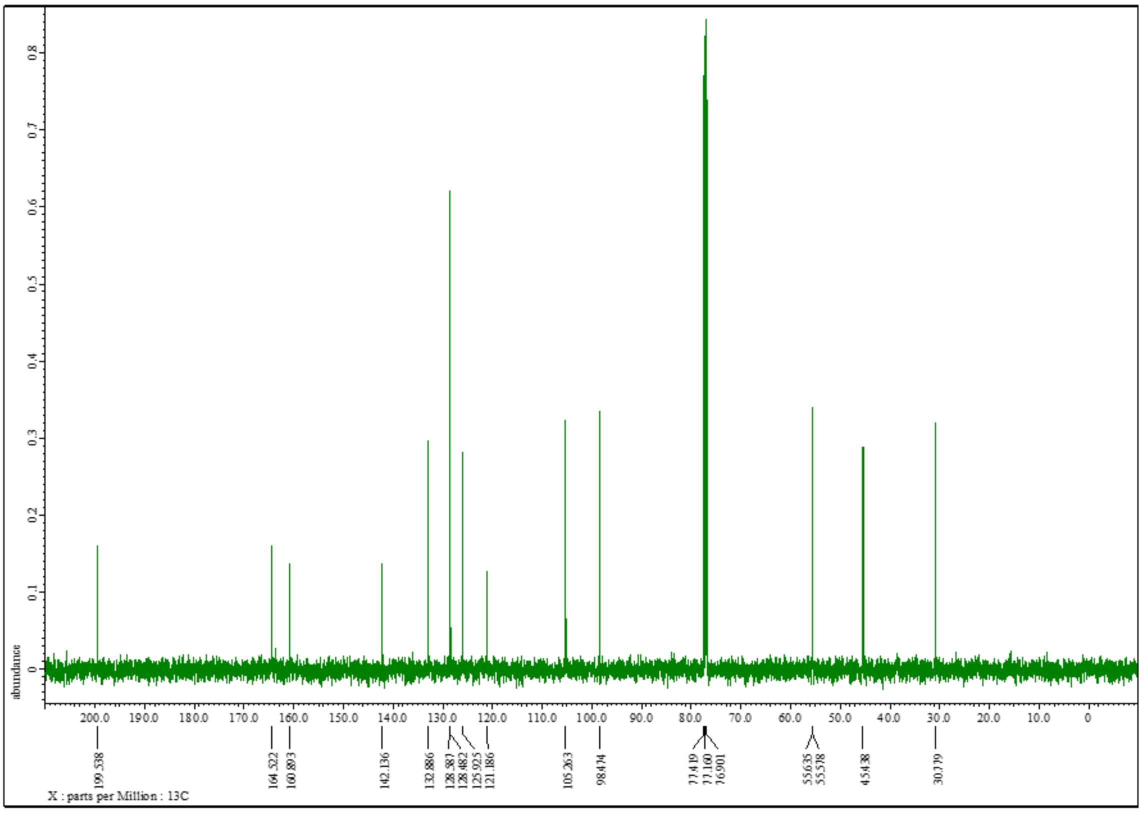

^{1}H NMR of spectrum of **11c** (500 MHz, $CDCl_3$)

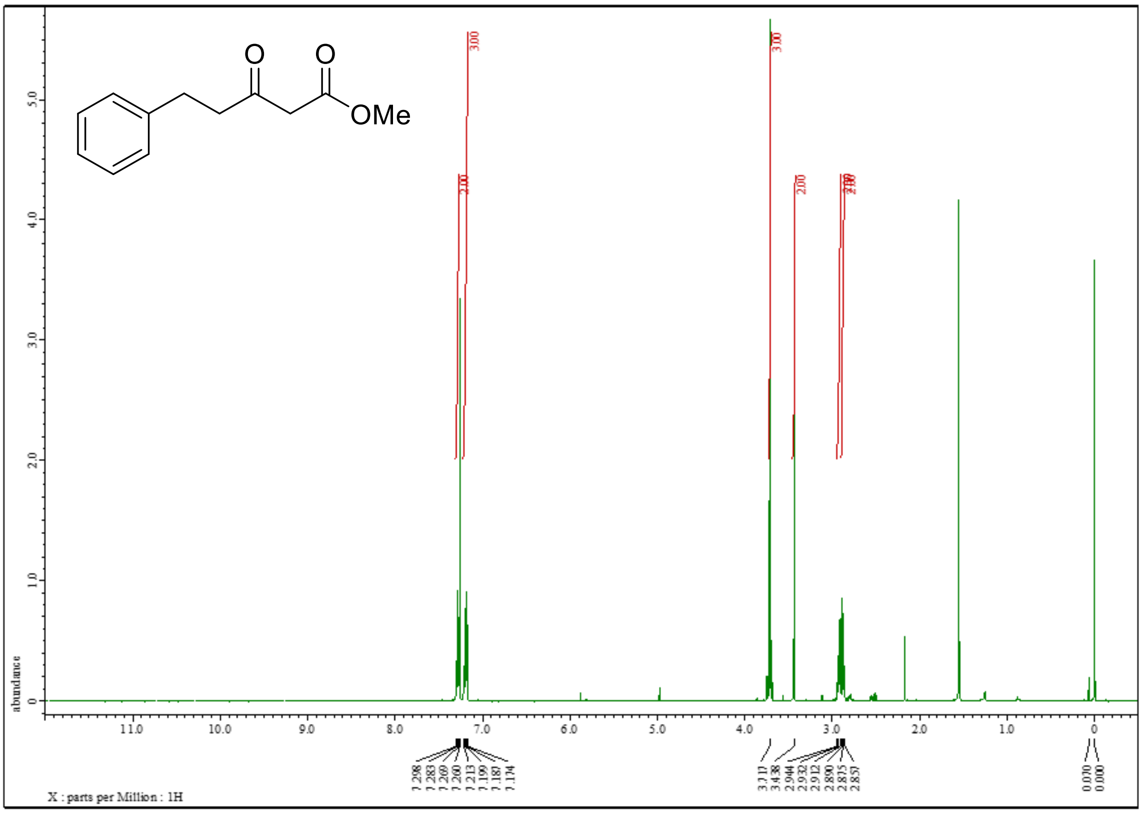

^{13}C NMR of spectrum of **11c (**125 MHz, $CDCl_3$)

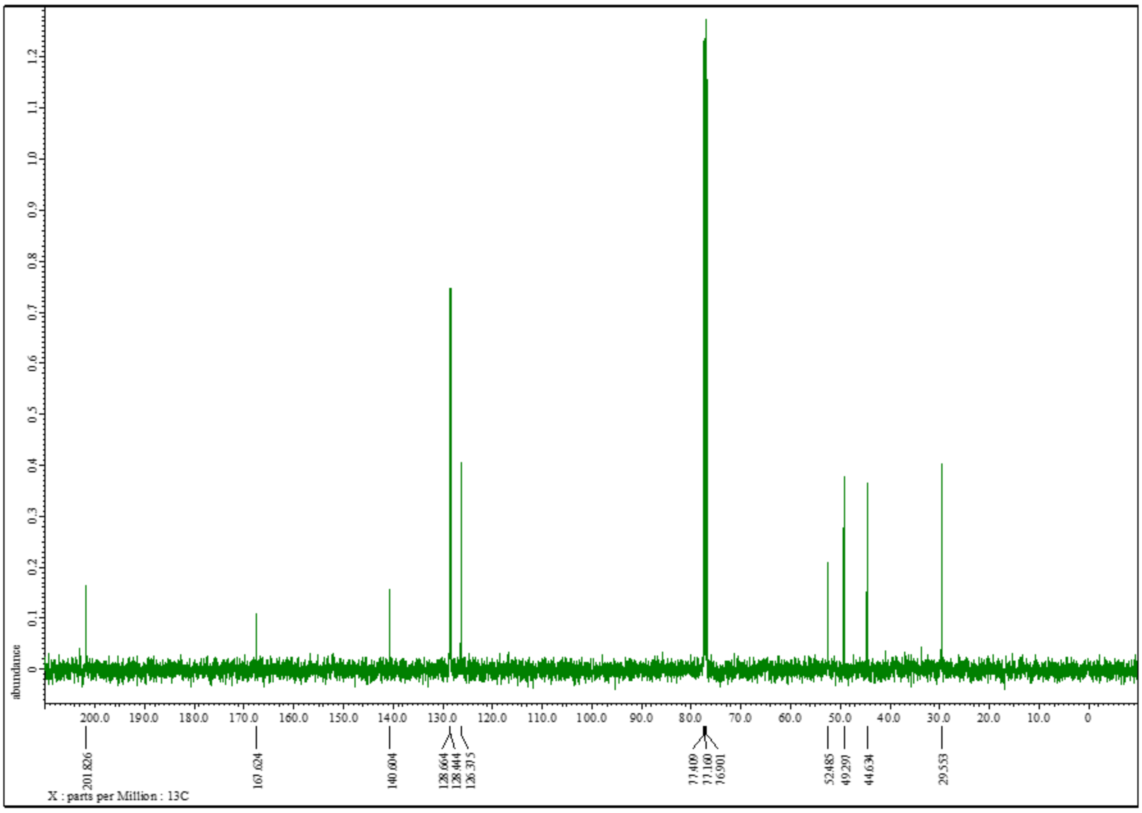

^{1}H NMR of spectrum of **11d** (500 MHz, $CDCl_3$)

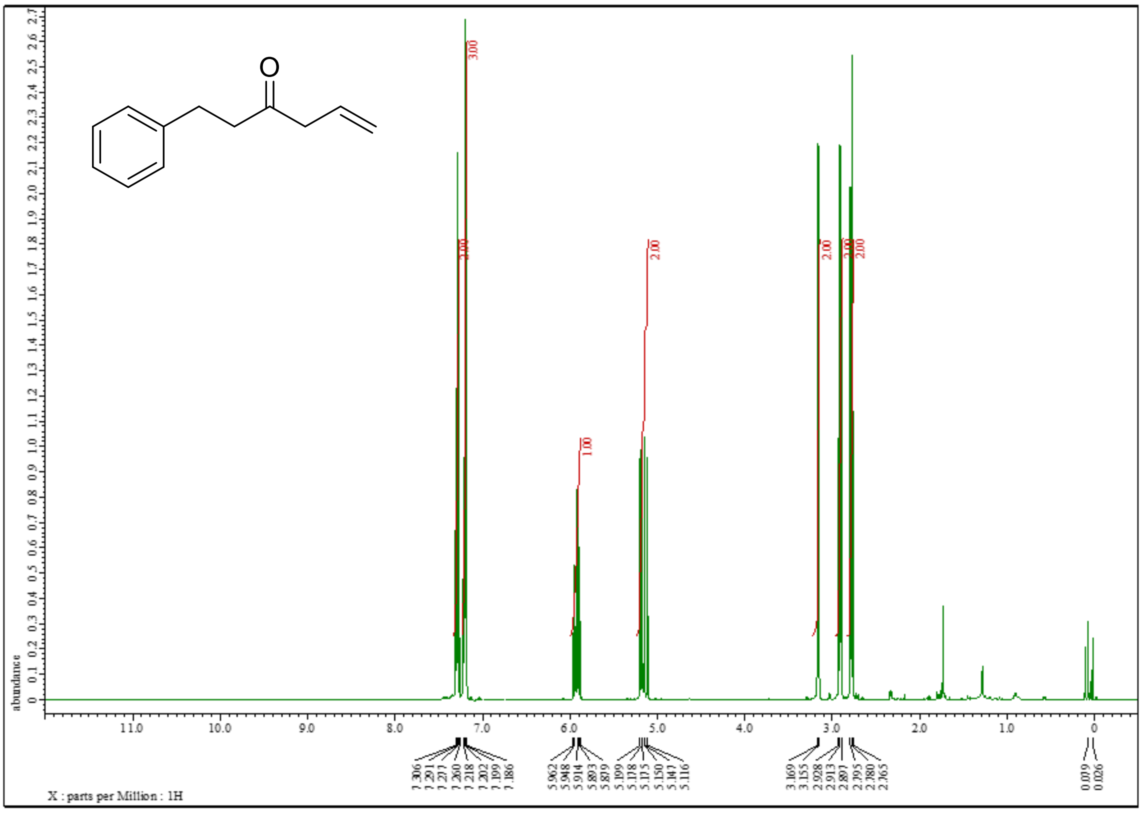

^{13}C NMR of spectrum of **11d** (125 MHz, $CDCl_3$)

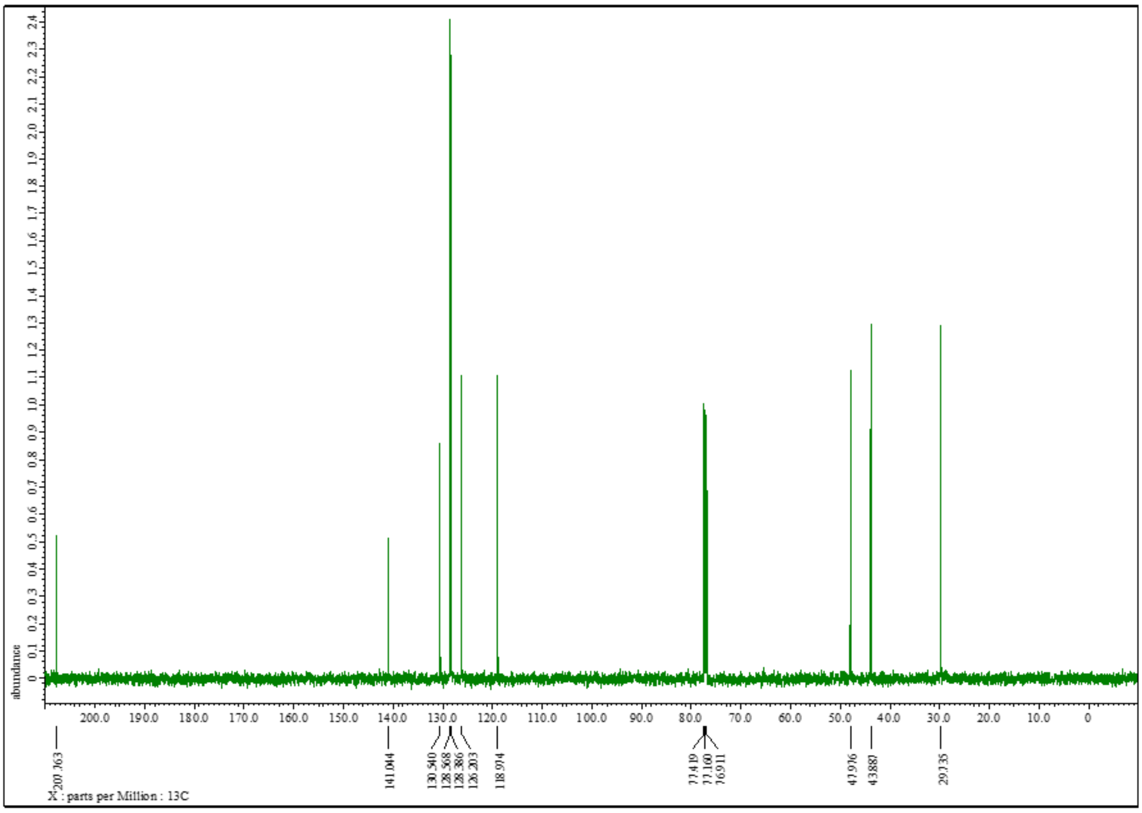

^{1}H NMR of spectrum of **11e** (500 MHz, $CDCl_3$)

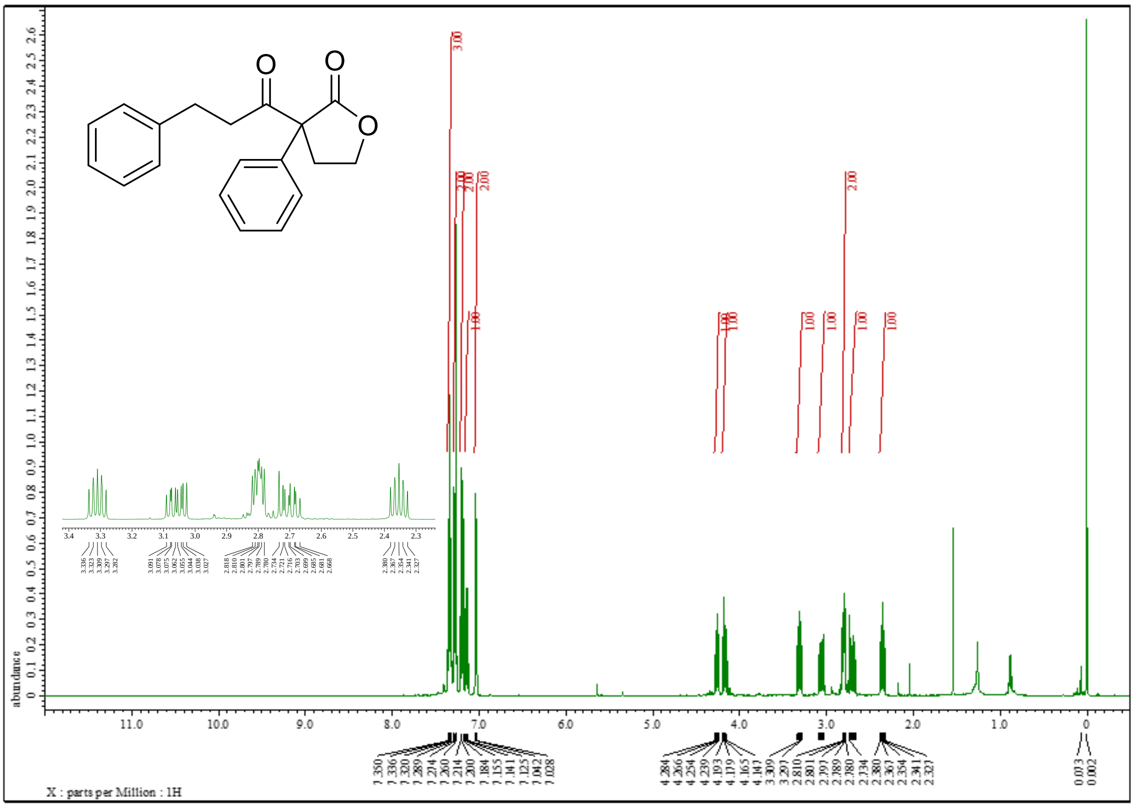

^{13}C NMR of spectrum of **11e** (125 MHz, $CDCl_3$)

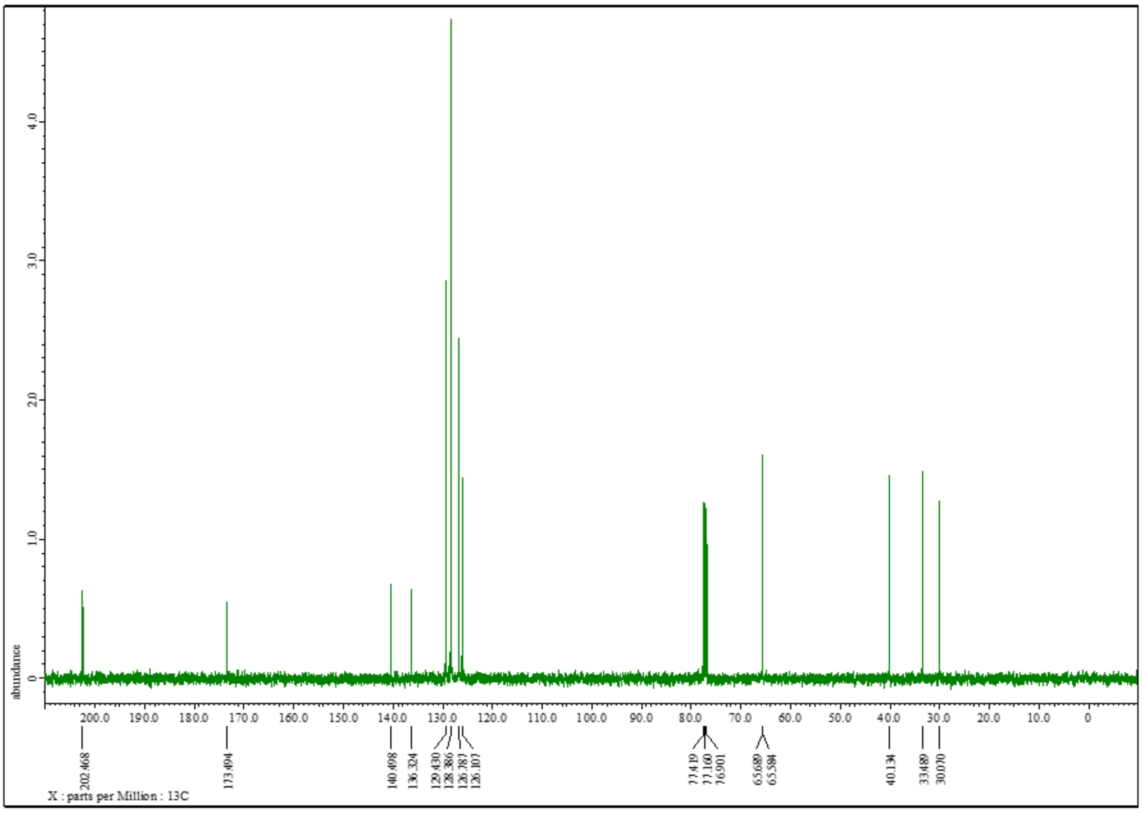

^{1}H NMR of spectrum of **13** (500 MHz, $CDCl_3$)

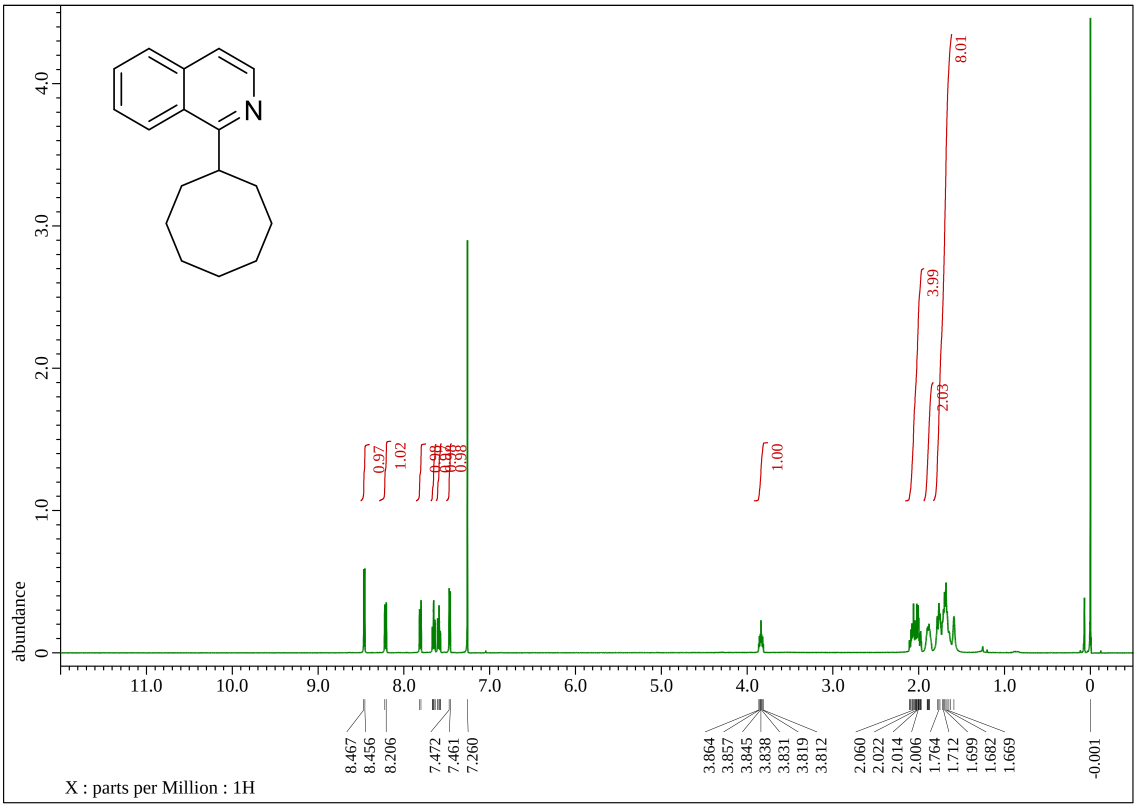

^{13}C NMR of spectrum of **13** (125 MHz, $CDCl_3$)

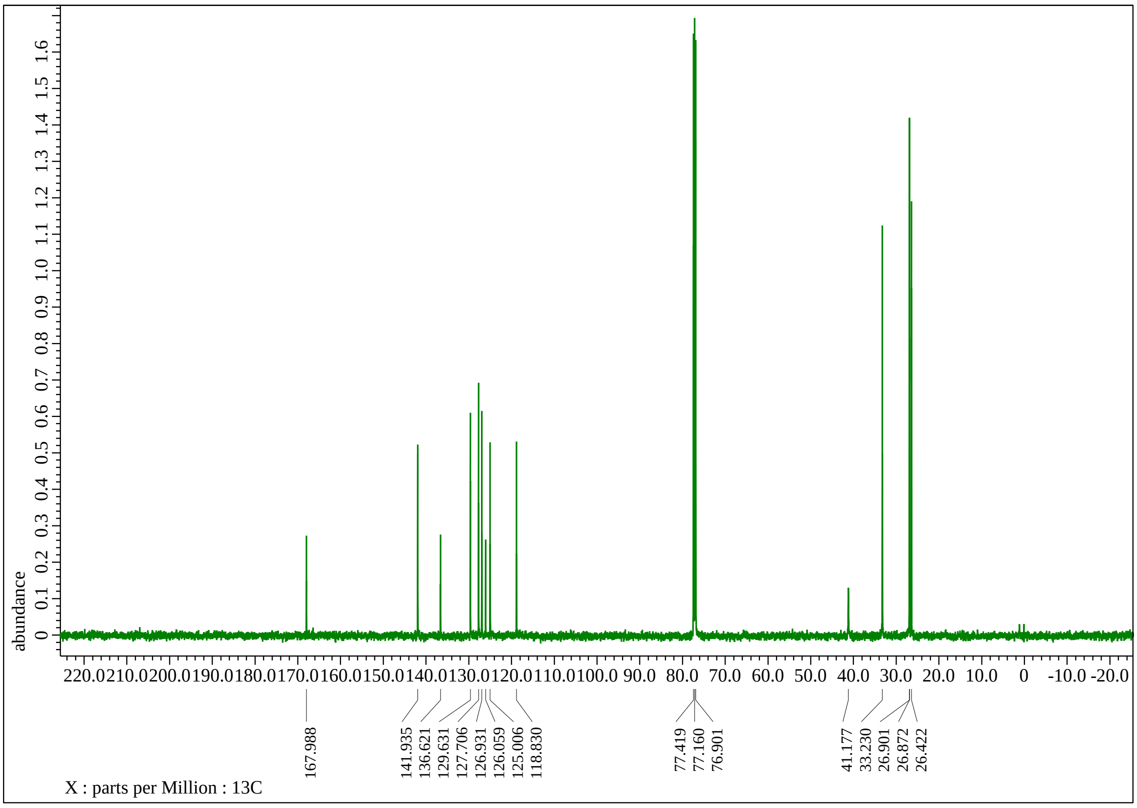

Chiral HPLC Chart

Benzyl ((1*S*, 2*R*)-3-(Benzylamino)-2-methyl-3-oxo-1-phenylpropyl)carbamate (7k)

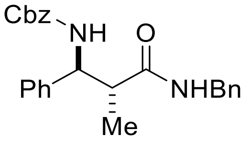

(*rac*)

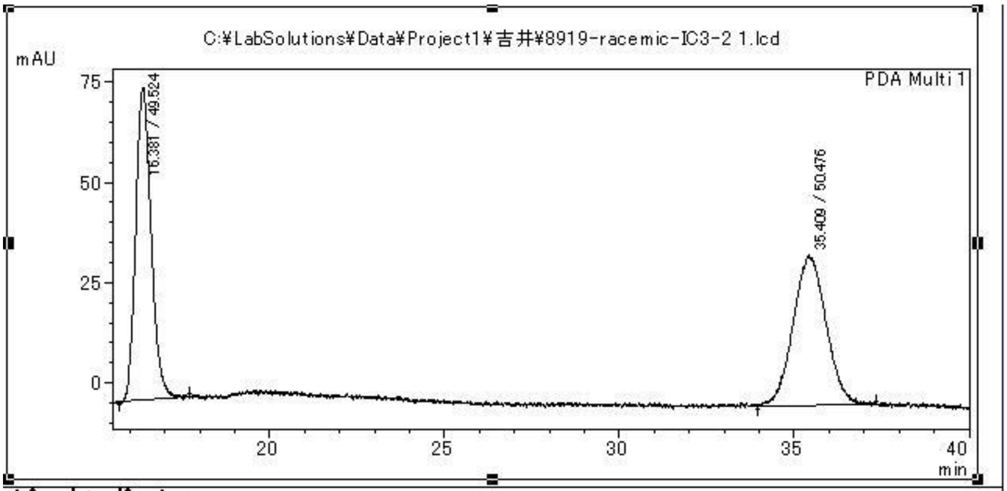

<ピークレポート>

C:¥LabSolutions¥Data¥Project1¥吉井¥8919-racemic-IC3-2 1.lcd

PDA Ch1 200nm 4nm

ピーク#	保持時間	面積	面積%	マーク
1	16.381	2390462	49.524	
2	35.409	2436415	50.476	
合計		4826878	100.000	

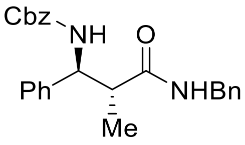

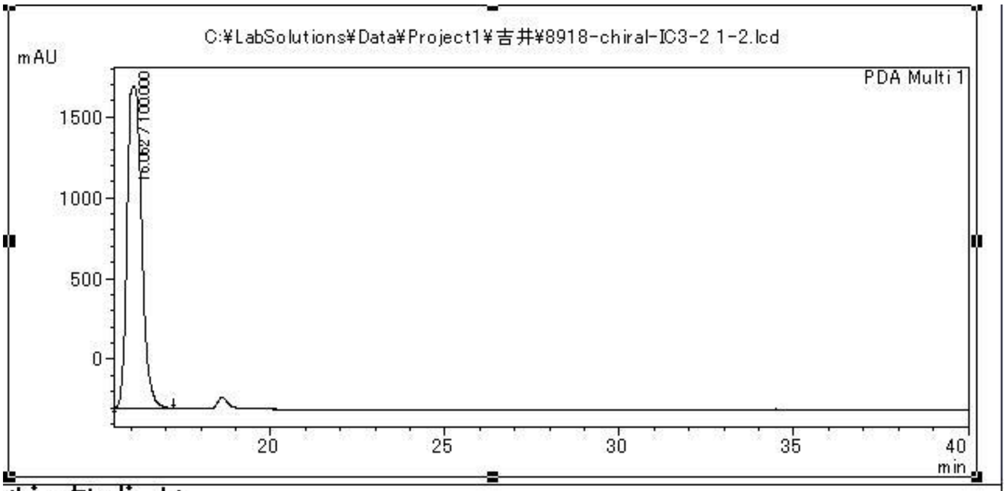

<ピークレポート>

C:¥LabSolutions¥Data¥Project1¥吉井¥8918-chiral-IC3-2 1-2.lcd

PDA Ch1 200nm 4nm

ピーク#	保持時間	面積	面積%	マーク
1	16.062	57916340	100.000	
合計		57916340	100.000	

Methyl (3-Phenylpropanoyl)-*L*-alaninate (8d)

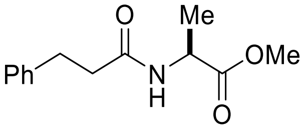

(*rac*)

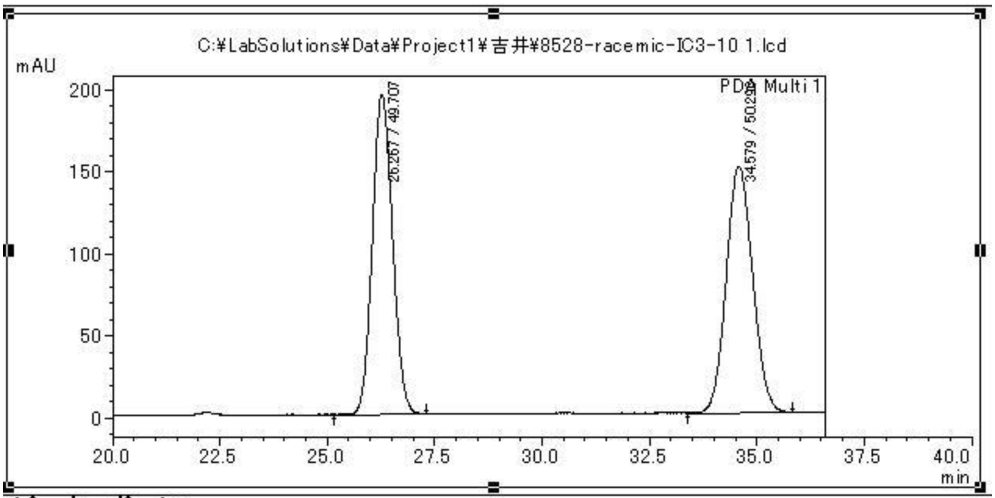

<ピークレポート>

C:¥LabSolutions¥Data¥Project1¥吉井¥8528-racemic-IC3-10 1.lcd

PDA Ch1 208nm 4nm

ピーク#	保持時間	面積	面積%	マーク
1	26.267	6351176	49.707	
2	34.579	6426072	50.293	
合計		12777249	100.000	

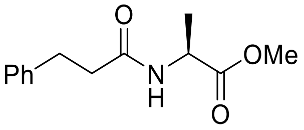

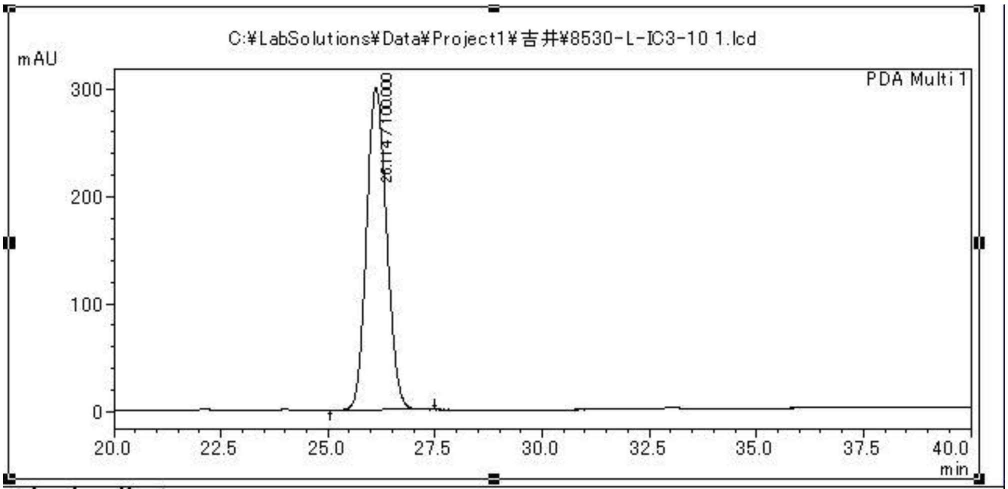

<ピークレポート>

C:¥LabSolutions¥Data¥Project1¥吉井¥8530-L-IC3-10 1.lcd

PDA Ch1 208nm 4nm

ピーク#	保持時間	面積	面積%	マーク
1	26.114	9793174	100.000	S
合計		9793174	100.000	
